# Molecular evolution of dietary shifts in ladybird beetles (Coleoptera: Coccinellidae): from fungivory to carnivory and herbivory

**DOI:** 10.1186/s12915-025-02174-2

**Published:** 2025-02-28

**Authors:** Yu-Hao Huang, Hermes E. Escalona, Yi-Fei Sun, Pei-Fang Zhang, Xue-Yong Du, Sen-Rui Gong, Xue-Fei Tang, Yuan-Sen Liang, Dan Yang, Pei-Tao Chen, Huan-Ying Yang, Mei-Lan Chen, Bruno Hüttel, Ondrej Hlinka, Xingmin Wang, Karen Meusemann, Adam Ślipiński, Andreas Zwick, Robert M. Waterhouse, Bernhard Misof, Oliver Niehuis, Hao-Sen Li, Hong Pang

**Affiliations:** 1 State Key Laboratory of Biocontrol, School of Ecology, Sun Yat-sen University, Shenzhen, 518107 China; 2 Australian National Insect Collection, CSIRO, GPO Box 1700, Canberra, ACT 2601 Australia; 3 College of Forestry, Henan Agricultural University, Zhengzhou, 450002 China; 4 School of Environmental and Life Sciences, Nanning Normal University, Nanning, 530001 China; 5 Max Planck Genome Centre Cologne, Max Planck Institute for Plant Breeding Research, Cologne, Germany; 6 CSIRO Information, Management and Technology, Pullenvale, QLD Australia; 7 College of Plant Protection, South China Agricultural University, Guangzhou, 510642 China; 8 Leibniz Institute for the Analysis of Biodiversity Change, Adenauerallee 127, Bonn, 53113 Germany; 9 Department of Ecology and Evolution, University of Lausanne and Swiss Institute of Bioinformatics, Lausanne, 1015 Switzerland; 10 Department of Evolutionary Biology and Ecology, Institute for Biology I (Zoology), University of Freiburg, Freiburg, 79104 Germany

**Keywords:** Coccinellidae, Ladybird beetle, Feeding habit, Evolution, Genome, Transcriptome, Chemosensation, Digestion, Detoxification, Immunity

## Abstract

**Background:**

Dietary shifts are major evolutionary steps that shape ecological niches and biodiversity. The beetle family Coccinellidae, commonly known as ladybirds, first transitioned from a fungivorous to an insectivorous and subsequently a plant diet. However, the molecular basis of this dietary diversification remained unexplored.

**Results:**

We investigated the molecular evolution of dietary shifts in ladybirds, focusing on the transitions from fungivory to carnivory (Coccinellidae) and from carnivory to herbivory (Epilachnini), by comparing 25 genomes and 62 transcriptomes of beetles. Our analysis shows that chemosensory gene families have undergone significant expansions at both nodes of diet change and were differentially expressed in feeding experiments, suggesting that they may be related to foraging. We found expansions of digestive and detoxifying gene families and losses of chitin-related digestive genes in the herbivorous ladybirds, and absence of most plant cell wall-degrading enzymes in the ladybirds dating from the transition to carnivory, likely indicating the effect of different digestion requirements on the gene repertoire. Immunity effector genes tend to emerge or have specific amino acid sequence compositions in carnivorous ladybirds and are downregulated under suboptimal dietary treatments, suggesting a potential function of these genes related to microbial symbionts in the sternorrhynchan prey.

**Conclusions:**

Our study provides a comprehensive comparative genomic analysis to address evolution of chemosensory, digestive, detoxifying, and immune genes associated with dietary shifts in ladybirds. Ladybirds can be considered a ubiquitous example of dietary shifts in insects, and thus a promising model system for evolutionary and applied biology.

**Supplementary Information:**

The online version contains supplementary material available at 10.1186/s12915-025-02174-2.

## Background

The acquisition and specialization of specific foods has been a major factor shaping the evolution of heterotrophic organisms, such as animals. Vertebrates, especially hominids and other mammals, have been the subject of several studies focusing on the anatomical and molecular basis of dietary shifts [1–5]. For invertebrates such as insects, the evolution of plant-based diets and its impact on their extraordinary diversification has been the subject of countless studies, spurred in part by the seminal work of Ehrlich and Raven [6] on the coevolution of butterflies and plants. While the evolutionary implications of a transition to herbivory are still much debated [7, 8], an adequate understanding of the molecular basis and mechanism of dietary shifts in insects remains challenging due to the lack of studies on taxa with appropriate dietary breath, phylogenetic diversity, and relatively recent age. Only *Drosophila*, with at least three herbivorous species, has emerged as a system to study insect diet shifts [9]. Here, we present genomic data and analyses to demonstrate that ladybird beetles may be a promising group to study the evolution of dietary shifts in insects.

Ladybird beetles (Coleoptera: Coccinellidae) comprise more than 6000 species worldwide [10]. They belong to the superfamily Coccinelloidea, a conglomerate of 15 currently recognized taxonomic families [11, 12]. Although largely known as voracious predators of aphids, the diets of the thousands of species of ladybirds are highly diverse. Approximately 36% of ladybirds feed primarily on coccids (including mealybugs and other scale insects), 20% feed on aphids, and 20% feed on plant leaves, while the remainder feed on fungi, pollen, or other insects [13–16]. In general, Coccinellidae are recognized as the largest group and the only family of beetles that prey extensively on sternorrhynchan insects (coccids, aphids, whiteflies, and psyllids), in contrast to other Coccinelloidea that feed primarily on fungi [13, 17].

Ladybird ancestors expanded their dietary preferences after angiosperm diversification in the Late Cretaceous [18, 19]. Host association and phylogenetic analyses on Coccinellidae and some Coccinelloidea indicated a putative dietary shift from fungi to Coccoidea [14], with further specialization in some major clades (e.g., Coccinellini mainly on aphids, Serangiini mainly on whiteflies) or a shift to other food sources such as plant leaves (e.g., Epilachnini, *Bulaea*), other arthropod lineages (e.g., preying on mites by representatives of Stethorini), and fungi (e.g., some genera in Coccinellini) [14, 20–22]. These dietary shifts make ladybirds a promising group to study the evolution of feeding habits. Comparative anatomical and physiological studies have revealed phenotypic differences between carnivorous and herbivorous ladybirds [23–28], for example, herbivorous ladybirds have a digestive system at least twice the length of carnivorous ones.

**Fig. 1:**
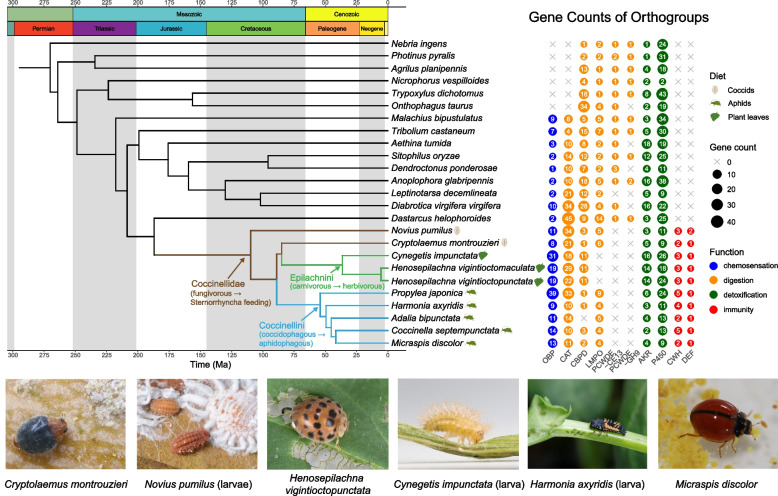
Phylogenetic tree and feeding-related genes of major lineages of ladybirds (Coccinellidae) and outgroup beetles. The time-calibrated maximum likelihood phylogeny was inferred from 770 near single-copy protein-coding genes using IQ-TREE and MCMCTREE. Candidate gene family abbreviations are listed in the Abbreviations section. PCWDE: plant cell wall degrading enzyme, GH9: glycosyl hydrolase family 9, CE13: carbohydrate esterase family 13. Photos by Hermes E. Escalona, Xue-Yong Du, Pei-Tao Chen, Xue-Fei Tang, and Hong Pang

In addition, because of their feeding habits, several ladybird species are important in agroecosystems and biosecurity. For example, *Cryptolaemus montrouzieri* is widely used for biocontrol of mealybugs [29], *Harmonia axyridis* is used for aphid control but it is also a major invasive species [30], the herbivorous *Henosepilachna vigintioctopunctata* is considered a major pest of Solanaceae [31], and *Micraspis discolor* can complete its life cycle by feeding on aphids or pollen; its impact on rice fields is still unclear [32] (Fig. 1). The prominent role of ladybirds in agricultural activities and natural ecosystems requires a deeper understanding of the mechanisms involved in their feeding habits.

Comparative genomics has become an essential tool for studying evolutionary questions, and genomic data combined with transcriptome expression profiles have been used to explain complex phenotypes in organisms, including their feeding habits. For example, such approaches have shed light on macroevolutionary signatures and genomic mechanisms of novel host plant shifts in butterflies [33], insect dietary adaptation in blind snakes [34], and herbivory and carnivory in mammals [1, 2, 35–37].

To investigate genomic changes associated with dietary shifts, one would need to comprehensively compare the sequenced genomes of multiple ladybirds that differ in their diets, as has been done in animal groups [2]. However, the currently available number of ladybird species with sequenced genomes precludes such a genome-based analysis, although the number of ladybird genomes and transcriptomes has increased significantly in recent years [38–47].

In other arthropods, various genome-based studies over the years have provided insights into genes related to feeding habits. These studies have shown that genes underlying dietary adaptations are often related to chemosensation, digestion, detoxification, and immunity. For example, a gustatory receptor encoded by a chemosensory gene was reported to determine the feeding preference for mulberry leaves in the silkworm [48]. Digestion-related genes encoding plant cell wall degrading enzymes (PCWDEs), acquired by horizontal gene transfer from bacteria and fungi, are used for lignocellulose degradation in at least two groups of herbivorous beetles, Buprestidae and Phytophaga [49–52]. The expansions of several detoxification-associated gene families are found to be potentially key to the polyphagous diet of spiders [53]. Overall, genes related to chemosensation, digestion, and detoxification appear to contribute to host adaptation and plant component metabolism in general in many herbivorous insects, such as beetles [54, 55], cactophilic flies [56–58], cotton bollworms [59], and plant hoppers [60], with a few studies on dietary adaptation in carnivorous insects (e.g., praying mantis [61], green lacewing [62], and hoverfly [63]). Furthermore, genomic and transcriptomic analyses have shown that diet affects insect immunity, and thus immune system responses may be relevant as evolutionary drivers of dietary shifts [64–66]. We have previously used transcriptomic data to study ladybird genes involved in adaptation to novel prey [67–70] and genomic data to study dietary adaptation to scale insects in *C. montrouzieri* and *Novius pumilus* [38, 39]. These studies also revealed the importance of genes associated with chemosensation, digestion, detoxification, and immunity.

In the present study, we report new insights into the molecular basis of ladybird dietary shifts from the analysis of a newly assembled dataset. Our dataset includes four newly sequenced and assembled genomes of ladybirds with different diets (i.e., the coccidophagous *C. montrouzieri* [CMONT, chromosome level], the aphidophagous *M. discolor* [MDISC], and the herbivorous *H. vigintioctopunctata* [HVIGI] and *Cynegetis impunctata* [CIMPU]) and transcriptomes of 25 species of Coccinelloidea. We complemented these data with six publicly available ladybird genomes, 37 species of additional Coccinelloidea transcriptomes, and the genomes of 15 beetle species as outgroups, providing the basis for comparative genomics at three diet-shift nodes: Coccinellidae, Coccinellini, and Epilachnini (Fig. 1). Given that previous research has found genes related to chemosensation, digestion, detoxification, and immunity to be of major importance in the evolutionary history of dietary shifts in animals, we hypothesized that protein-coding genes related to these functions should be relevant to dietary adaptations in the ladybirds, and that members of these gene families should be (1) involved in adaptive lineage-specific gene family dynamics or specific amino acid sequence compositions, (2) differentially expressed under different dietary treatments, and (3) highly expressed in correspondingly relevant tissues (i.e., gut for digestive and detoxifying genes, antenna and head for chemosensory genes). Focusing on these candidate gene families, we performed phylogenetic analyses coupled with diet- and tissue-specific transcriptome profiling to further explore their evolutionary history and expression patterns in relation to dietary shifts.

## Results

### Genomes, transcriptomes, and ortholog groups of 69 ladybird species (see Sect. 1 of the Additional file 1 for details)

To increase the number of ladybird species with available genomes, we first de novo sequenced and assembled the genomes of three species (i.e., *M. discolor*, *H. vigintioctopunctata*, *C. impunctata*) using a combination of short reads (Illumina) and long reads (Nanopore, Pacbio). For a fourth species, *C. montrouzieri*, we used data from the previously published contig-level assembly (Li, Huang [38]) to construct a chromosome-level assembly using Hi-C technology. The completeness of these new resources was assessed using the Benchmarking Universal Single-Copy Orthologs (BUSCO) tool (OrthoDB version 10, Insecta lineage dataset, *n* = 1367) [71, 72]. The gene space completeness scores and assembly N50 values were as follows: *C. montrouzieri*, 99.2%, 101.22 Mb; *M. discolor*, 94.3%, 2.63 Mb; *H. vigintioctopunctata*, 99.3%, 5.76 Mb; and *C. impunctata*, 98.2%, 562.46 kb (Table 1). We complemented this dataset by including published assemblies of an additional six species, resulting in a total of ten ingroup genomes for our analyses [38–42, 46, 47]. Note that the species whose genomes we have selected for our comparative analyses are predominantly carnivores and herbivores (Table 1, Fig. 1). Specifically, two of the species feed on scale insects (i.e., *C. montrouzieri* [CMONT] and *Novius pumilus* [NPUMI]), three feed on plant leaves (i.e., *H. vigintioctopunctata* [HVIGI], *C. impunctata* [CIMPU], and *Henosepilachna vigintioctomaculata* [HVIMA]), and five feed primarily on aphids (i.e., *M. discolor* [MDISC], *H. axyridis* [HAXYR], *Coccinella septempunctata* [CSEPT], *Adalia bipunctata* [ABIPU], and *Propylea japonica* [PJAPO]).

**Table 1:** Diet information and general genomic features of ten ladybirds with genomes

Species	Abbr	Optimal diet	Genome size	Assembly N50	Genome completeness	Genome ref
*Novius pumilus*	NPUMI	Coccids, mainly cottony cushion scales (*Icerya*)	182.42 Mb	7.58 Mb	97.8%	[39]
*Cryptolaemus montrouzieri*	CMONT	Optimal: coccids, mainly mealybugs (Pseudococcidae) suboptimal: other arthropods (e.g., aphids, whiteflies, psyllids)	988.13 Mb	101.22 Mb	99.2%	[38]; this study
*Harmonia axyridis*	HAXYR	Optimal: aphids suboptimal: other arthropods (e.g., coccids, whiteflies, psyllids, thrips, mites)	425.54 Mb	63.68 Mb	99.0%	[41]
*Coccinella septempunctata*	CSEPT	Optimal: aphids suboptimal: other arthropods (e.g., coccids, whiteflies, psyllids, mites)	398.87 Mb	41.44 Mb	99.2%	[42]
*Propylea japonica*	PJAPO	Optimal: aphids suboptimal: other arthropods (e.g., coccids, whiteflies, psyllids)	851.23 Mb	100.34 Mb	95.8%	[40]
*Adalia bipunctata*	ABIPU	Optimal: aphids suboptimal: other arthropods (e.g., coccids, whiteflies, psyllids)	475.29 Mb	45.87 Mb	98.8%	[46]
*Micraspis discolor*	MDISC	Optimal: aphids suboptimal: pollen, other arthropods (e.g., coccids, whiteflies, hoppers, thrips, mites)	523.75 Mb	2.63 Mb	94.3%	This study
*Henosepilachna vigintioctomaculata*	HVIMA	Plant leaves, mainly Solanaceae	581.63 Mb	56.17 Mb	99.1%	[47]
*Henosepilachna vigintioctopunctata*	HVIGI	Plant leaves, mainly Solanaceae	496.12 Mb	5.76 Mb	99.3%	This study
*Cynegetis impunctata*	CIMPU	Plant leaves, mainly Poaceae	796.00 Mb	562.46 kb	98.2%	This study

Diet information was collected from the previous researches and reviews [10, 13–15, 20, 21, 32, 73–78]. The size and N50 of the genomes were all calculated in this study. The completeness of the genomes was estimated by the completeness scores of BUSCO (OrthoDB version 10, Insecta lineage dataset)

To study the evolution of protein-coding genes in ladybird beetles, we annotated the complete predicted protein sets in all ten ladybird genomes and collected the annotated protein sets of fifteen published beetle genomes [79–94] belonging to different families as outgroups (Fig. 1, Additional file 2: Table SE1). The BUSCO completeness scores of the annotated protein-coding genes from the resulting 25 beetle genomes (hereafter referred to as the genome dataset) ranged from 90.2 to 99.6%.

We also inferred protein-coding gene sets from 62 Coccinelloidea transcriptomes, consisting of 37 published and 25 newly sequenced ones (BUSCO completeness scores: 82.0 to 97.4%). This resulted in a total of 87 gene sets (69 from ladybirds and 18 from outgroup beetles) for which we identified orthologous genes (ortholog groups; OGs). Protein-coding genes were assigned to a total of 148,089 OGs, including 1074 common to all 87 species, which formed the basis of our downstream phylogenomic analyses.

### Time-calibrated phylogeny and ancestral state reconstruction (see Sect. 2 of Additional file 1 for details)

We inferred the phylogeny of the 87 beetle species studied by analyzing 770 OGs of nearly single-copy protein-coding genes. Concatenation of the corresponding amino acid alignments resulted in a supermatrix of 295,332 amino acid (aa) positions. Phylogenetic analysis of this supermatrix using the maximum likelihood optimality criterion was performed with IQ-TREE [95], specifying a gene-partitioned matrix and selecting the most appropriate substitution model for each partition separately. The resulting reconstructed phylogenetic tree confirmed all major taxonomic lineages of interest (i.e., Coccinellidae, Coccinellini, Epilachnini) as monophyletic entities with high statistical support (ultrafast bootstrap values = 100%).

We estimated divergence times with a Bayesian approach using MCMCTREE [96] and eleven fossil calibration points. The time-calibrated phylogeny suggests that Coccinellidae originated in the Early Cretaceous, 142 million years ago (= Ma; 95% confidence intervals [CI]: 156–131 Ma). The crown groups Coccinellini and Epilachnini originated in the Paleogene, 54 Ma (95% CI 60–50 Ma) and 48 Ma (95% CI 53–44 Ma), respectively.

Using this time-calibrated phylogeny, we reconstructed the ancestral character states of feeding habits at all nodes within Coccinelloidea using published data on feeding habits across the lineage. Species for which no feeding information was available were discarded from the analysis, leaving a total of 61 species for stochastic character mapping using the phytools package [97]. Our results indicate that the diet of the most recent common ancestor of Coccinellidae was most likely scale insects (state probability (SP) = 81%) and that its diet was most likely derived from an ancestral fungivorous diet (Fig. 1), in agreement with previous analyses [14, 17, 22, 98]. Dietary shifts from coccidophagy to aphidophagy and to herbivory likely occurred in the lineages leading to Coccinellini and Epilachnini, respectively (SP ≥ 99%). The identified shifts provide the basis for our comparative genomic and transcriptomic analyses to explore the evolutionary history and expression patterns of candidate gene families in ladybirds.

### Evolutionary histories of gene families putatively associated with dietary shifts (see Sects. 3 and 4 of Additional file 1 for details)

Focusing on our genome dataset, we searched for signatures of selection in the subset of single-copy orthologs using PAML [96], and we quantified gene family dynamics (expansions and contractions) using CAFE [99] across all OGs. In addition, we quantified gene losses from phylogenetic lineages and de novo lineage-specific gene emergence by comparing gene counts across the phylogeny. The selection analyses identified a small number of genes with sites showing signatures of positive, relaxed, or enhanced selection along branches (Sect. 3 of Additional file 1). For convenience, we hereafter refer to OGs with significant expansions and contractions, or with clade-specific gene loss, or with lineage-specific de novo gene emergence, as lineage-specific evolving gene families (LEGFs). We focused our analysis of LEGFs on the three nodes of the ladybird phylogeny associated with major dietary shifts (Fig. 1), viz: Coccinellidae, Coccinellini, and Epilachnini.

**Fig. 2:**
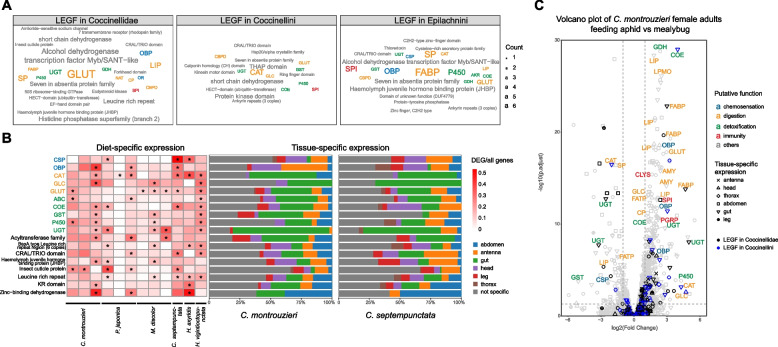
Functional analysis of candidate genes likely involved in chemosensation, detoxification, digestion, and immunity in ladybirds. **A** Functional enrichment of gene families that significantly expanded, significantly contracted, or were emergent or lost at the nodes (= lineage-specific evolving gene families: LEGF) of Coccinellidae, Coccinellini (switch to carnivory), and Epilachnini (switch to herbivory). **B** Functional enrichment and high expression tissues of genes differentially expressed between carnivorous and herbivorous ladybirds. The heatmap color indicates the ratio of differentially expressed genes (DEGs) in all genes with the target function. Asterisks indicate statistically significant (adjusted *p*-value < 0.05) enrichments. **C** Volcano plot showing log-fold expression differences, high expression tissues, and putative function of genes differentially expressed in female adults of *Cryptolaemus montrouzieri* when fed with aphids instead of mealybugs. Candidate gene family abbreviations are listed in the Abbreviations section

We identified overrepresented functional gene categories using clusterProfiler [100]. LEGFs at the Coccinellidae, Coccinellini, and Epilachnini nodes were enriched with gene families functionally related mainly to chemosensation (CSP, OBP, OR), digestion (CAT, CBPD, CP, FABP, GLC, GLUT, LIP, NAT, SP), detoxification (AKR, COE, GDH, GST, P450, UGT), and immunity (SPI) (Fig. 2A; candidate gene family abbreviations are listed in the Abbreviations section).

### Diet-specific differentially expressed genes and tissue-specific gene expression (see Sects. 5 and 6 of Additional file 1 for details)

Six ladybird species (five carnivorous, one herbivorous) were subjected to diet-specific experiments and transcriptome profiling (Additional file 1: Table S5.1). Transcriptome profiling of different tissues (i.e., abdomen without gut, antennae, gut, head, legs, thorax) was performed on *C. montrouzieri* and *C. septempunctata*, and transcriptome profiling of gut and body without gut was performed on *C. impunctata*. We found that gene families related to chemosensation (CSP, OBP), digestion (CAT, GLC, GLUT), and detoxification (ABC, COE, GST, P450, UGT) were significantly enriched in diet-specific comparisons in at least three ladybird species (e.g., aphid vs. mealybug diet treatments in female adults of *C. montrouzieri*) (Figs. 2B, C). Digestion- and detoxification-related genes were mainly highly expressed in the gut, while chemosensation-related genes were found to be highly expressed in the antennae or head (Fig. 2B). In addition, OG enrichment analysis of diet-specific differentially expressed genes (= DEGs) revealed that specific OGs containing large numbers of DEGs mostly belong to gene families related to chemosensation, digestion, detoxification, and immunity (Additional file 1: Tables S5.2 and S5.3). The LEGFs at the Coccinellidae, Coccinellini, and Epilachnini nodes also tended to be differentially expressed in the corresponding diet comparisons (Additional file 1: Figures S5.2, S5.3 and S5.7).

### Evolutionary dynamics of the candidate gene families

Our hypothesis that genes related to chemosensation, digestion, detoxification, and immunity are likely relevant to ladybird dietary adaptation are supported by our statistical enrichment analyses. We subsequently present additional information (e.g., OG size, clade-level gene dynamics and sequence composition, diet- and tissue-specific expression patterns) on gene families related to these functions, with the main results summarized in Table 2. The gene dynamics (e.g., expansion, de novo gene emergence and gene loss) were detected at not only OG level but also clade level (see methods for details).

**Table 2:** Ortholog groups of the candidate gene families evolving at the nodes with diet shifts

Orthogroup	Gene families	Putative function	Orthogroup size	Clade within orthogroup	Diet-specific expression	Highly expressing tissue
OG0000083	OR	Chemosensation	Contraction (Cd)	Nothing found	No DEG	Antenna
OG0000120	OBP	Chemosensation	Expansion (Cd, E)	Duplication (E)	Totally 32/61 DEGs	Antenna, head
OG0000042	CSP	Chemosensation	Nothing found	Nothing found	4/20 up (CMONT); 4/28 up (PJAPO); 11/17 up (CSEPT); 6/15 up (HAXYR)	Antenna, head, leg
OG0000509	SNMP	Chemosensation	Nothing found	Two duplications (Cd)	2/3 up (CMONT); 1/4 down (HVIGI)	Head, gut
OG0000196	CP	Digestion	Nothing found	Duplication (E)	2/2 down (HVIGI)	Gut
OG0000255	SP	Digestion	Expansion (Cd, E)	Nothing found	No DEG	Male abdomen
OG0000065	CAT	Digestion	Contraction (Cn), near Expansion (Cd, *p* = 0.084)	Duplication (Cn)	7/14 up (CMONT); 2/9 down (MDISC); 2/10 down (CSEPT); 3/10 down (HAXYR); 5/29 down (PJAPO); 6/20 down (HVIGI)	Gut
OG0000380	ASP	Digestion	Nothing found	Duplication (E)	2/4 down (HVIGI)	Gut
OG0003251	ASP	Digestion	Nothing found	Duplication (E)	2/2 down (HVIGI)	Gut
OG0000287	MMP	Digestion	Expansion (E), contraction (Cn)	Nothing found	No DEG	Nothing found
OG0000110	GLC	Digestion/detoxification	Contraction (Cn), near expansion (E, *p* = 0.117)	Tandem and segmental duplications (E)	6/13 down (HVIGI)	Gut
OG0000139	Lipase	Digestion	Expansion (E)	Loss (Cn)	2/17 down (HVIGI)	Nothing found
OG0000184	Chitinase	Digestion	Nothing found	Two losses (E)	2/2 and 1/2 down respectively (pollen-fed MDISC)	Gut
OG0000068	GLUT	Digestion	Expansion (Cd, E)	Duplications (Cd, E)	5/29 DEGs (CMONT); 4/31 DEGs (HVIGI)	Thorax, abdomen
OG0000088	GLUT	Digestion	Expansion (Cd, E)	Nothing found	8/12 DEGs (CMONT); 8/21 down (HVIGI)	Gut
OG0000283	FABP	Digestion	Expansion (Cd, E)	Duplication and de novo emergence (E)	3/12 DEG (CMONT); 9/27 down (HVIGI)	Gut
OG0000118	FATP	Digestion	Nothing found	Two duplications (E)	3/10 down (HVIGI)	Gut, abdomen
OG0000227	CBPD	Digestion	Expansion (E), contraction (Cn)	Two duplications (E)	9/11 down (HVIGI)	Gut
OG0000406	LPMO	Digestion	Loss (E)	Nothing found	5/6 DEGs (CMONT); 2/4 down (pollen-fed MDISC)	Gut
OG0000205	ABC	Detoxification	Nothing found	Three duplications (E)	1/5 down (HVIGI)	Gut
OG0000050	P450	Detoxification	Expansion (E)	Two duplications (E)	6/24 down (HVIGI); 6/7 DEGs (CMONT); 6/24 DEGs (PJAPO)	Gut
OG0000153	P450	Detoxification	Expansion (E)	Nothing found	3/11 down (HVIGI)	Nothing found
OG0000289	GST	Detoxification	Expansion (E), contraction (Cn)	Nothing found	2/7 down (HVIGI)	Gut
OG0000047	UGT	Detoxification	Nothing found	Two duplications (E), duplication (Cn)	3/13 down (HVIGI); 2/9 DEGs (CMONT); 4/19 up (PJAPO); 2/13 down (MDISC)	Gut
OG0000113	UGT	Detoxification	Expansion (E)	Duplication (E)	4/22 down (HVIGI); 10/15 DEGs (MDISC)	Gut
OG0000247	UGT	Detoxification	Expansion (Cd), contraction (Cn)	Nothing found	9/21 DEGs (CMONT); 5/10 down (MDISC)	Gut
OG0000439	UGT	Detoxification	Expansion (Cd)	Nothing found	4/10 down (CMONT); 4/14 DEGs (PJAPO)	Gut
OG0000084	COE	Detoxification	Expansion (E)	Two duplications (E), loss (Cn)	4/17 down (HVIGI); 6/7 DEGs (CMONT)	Gut, thorax and abdomen
OG0000276	AKR	Detoxification	Expansion (E)	Four duplications (E)	2/14 down (HVIGI)	Gut
OG0000136	GDH	Detoxification	Expansion (E)	Duplication (E), duplication (Cn)	1/20 down (HVIGI)	Gut
OG0000423	GDH	Detoxification	Nothing found	Two duplications (E)	2/9 down (HVIGI)	Gut (CIMPU)
OG0000273	Attacin	Immunity	Nothing found	Loss (Cn)	3/4 down (CMONT)	Nothing found
OG0009673	Defensin	Immunity	De novo emergence (Cd)	Loss of amino acid sequence composition (E)	1/1 DEG (MDISC); 1/1 down (HAXYR)	Nothing found
OG0000458	Coleoptericin	Immunity	Nothing found	Specific amino acid sequence compositions (Cd, Cn)	1/3 down (CMONT); 2/2 down (PJAPO); 7/11 down (MDISC)	Nothing found
OG0009100	ILYS	Immunity	De novo emergence (Cd)	Nothing found	2/2 down (CMONT); 1/1 down (CSEPT); 2/2 down (MDISC)	Nothing found
OG0009356	ILYS	Immunity	De novo emergence (Cd)	Nothing found	No DEG	Nothing found
OG0001441	CWH	Immunity	De novo emergence (Cd)	Nothing found	1/2 down (CMONT); 2/5 down (PJAPO); 1/4 up (HAXYR); 1/2 down (CSEPT); 2/5 up (MDISC); 1/3 down (HVIGI)	Nothing found
OG0000225	Serpin	Immunity	Expansion (Cd, E), contraction (Cn)	Duplication (E)	5/19 down (HVIGI); 5/9 up (CMONT); 1/2 down (PJAPO)	Gut, abdomen, head

Detailed results and discussion can be found in the Additional file 1: Sect. 7–10. Candidate gene family abbreviations are listed in the Abbreviations section. Cd: occurred at the node of Coccinellidae, Cn: occurred at the node of Coccinellini, E: occurred at the node of Epilachnini. DEGs: differentially expressed genes (containing both up and downregulated genes), up: upregulated DEGs under non-optimal diet treatments, down: downregulated DEGs under non-optimal diet treatments.

*CMONT* *Cryptolaemus montrouzieri*, *HVIGI* *Henosepilachna vigintioctopunctata*, *CSEPT* *Coccinella septempunctata*, *HAXYR* *Harmonia axyridis*, *PJAPO* *Propylea japonica*, *MDISC* *Micraspis discolor*, *CIMPU* *Cynegetis impunctata*

#### Chemosensory gene families (see Sect. 7 of Additional file 1 for details)

**Fig. 3:**
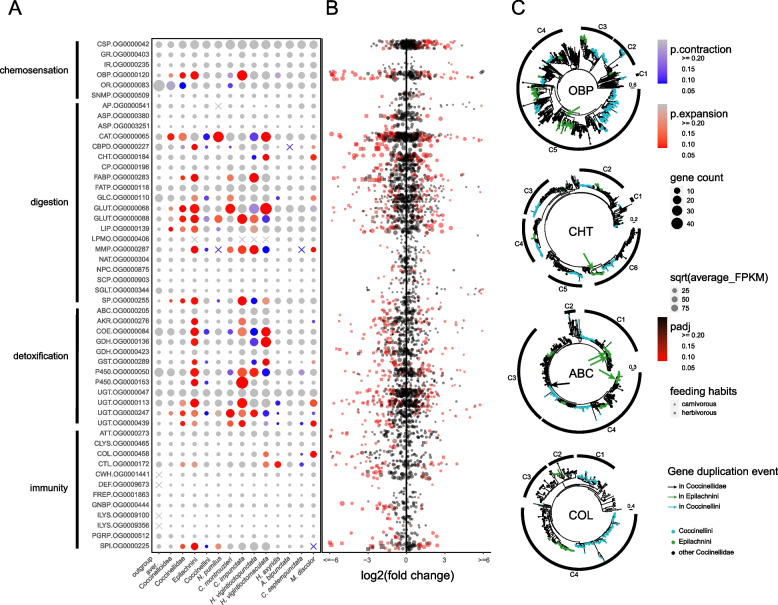
Family size and clade-level dynamics of candidate feeding-related genes in ladybirds (Coccinellidae). **A** Expansions, contractions, gene losses from entire subgroups, and de novo lineage-specific gene emergences in ortholog groups. Gene counts of the nodes of Coccinelloidea, Coccinellidae, Epilachnini, and Coccinellini are reconstructed by CAFE. Outgroup averages were calculated based on the gene counts from 15 outgroup species. **B** Diet-specific differentially expressed genes in candidate gene families. **C** Phylogenies of odorant-binding protein (OBP) OG0000120, chitinase (CHT) OG0000184, ATP-binding cassette transporter (ABC) OG0000205, and coleoptericin (COL) OG0000458 showing gene duplications or losses in Coccinellidae and Epilachnini. Candidate gene family abbreviations are listed in the Abbreviations section

We found evolutionary dynamics in the chemosensation-related OGs, with evidence of expansion (OBP, SNMP) and contraction (OR) events (Fig. 3A). The chemosensation-related OGs also contained diet-specific DEGs (mainly OBP, CSP and SNMP, Fig. 3B). Compared to other chemosensation-related OGs, the largest OG of OBP (OG0000120) was notably expanded in Coccinellidae and Epilachnini (Figs. 1 and 3A). Clade C5 of OG0000120 contributes the most to the gene repertoire expansion in Coccinellidae (Fig. 3C), with 3/5 genes being highly expressed in the antennae in *C. montrouzieri*, whereas half of genes in *C. montrouzieri* and *C. septempunctata* of clades C2 and C3 are clearly expressed in the head (Additional file 1: Figure S7.3). The gene expansion in OG0000120 of the tribe Epilachnini is largely due to a single gene duplication in clade C5 (Fig. 3C). Overall, approximately 50% of the genes in OG0000120 were found to have diet-specific expression in ladybirds (Fig. 3B).

#### Digestive gene families (see Sect. 8 of Additional file 1 for details)

We examined the presence of genes encoding PCWDEs of interest (i.e., cellulases and pectinases) in Coccinellidae and outgroup beetles. We found that Coccinellidae encode only GLC (glycosyl hydrolase family 1 (GH1) domain-contained) genes, while most outgroup beetles encode not only genes of this family but also glycosyl hydrolase family 9 (GH9) and carbohydrate esterase family 13 (CE13) genes (Figs. 1 and 3A). Among the candidate digestive OGs, those belonging to at least seven gene families exhibit expansions (ASP, CBPD, CP, FABP, FATP, GLC, GLUT) in Epilachnini (Figs. 1 and 3A). These OGs also contain a relatively high proportion (13–100%) of genes that are differentially expressed under different dietary treatments (Fig. 3B) as well as highly expressed in the gut (Additional file 1: Figures S8.3, S8.9 and S8.13). In Coccinellidae, only the OGs of GLUT and FABP were expanded. We found that genes of these two gene families are highly expressed in the gut and show large numbers of diet-specific DEGs (25–67%) in the carnivorous ladybirds. Among the candidate chitin-degrading enzymes, we found genes of the chitinase OG0000184 and of the lytic polysaccharide mono-oxygenase (LPMO) OG0000406 to be often highly expressed in the gut. Epilachnini lost genes of the OG0000184 clades C3 and C5 (Figs. 3C) as well as all genes of OG0000406 (Fig. 3A). In *M. discolor*, the genes of both clades (C3: 2/2, C5: 1/1) in OG0000184 and 2/4 genes of OG0000406 were downregulated when individuals were fed on pollen instead of insects. In Coccinellini, the CAT OG0000065 is significantly contracted in size (Figs. 1 and 3A). We found that most genes in this OG are highly expressed in the gut, and we found that expression of many genes in this OG was upregulated in *C. montrouzieri* (50%) and downregulated in Coccinellini (17–30%) when individuals of each taxon were fed on suboptimal diets (e.g., aphids for coccidophagous *C. montrouzieri*, and mealybugs for aphidophagous Coccinellini species).

#### Detoxifying gene families (see Sect. 9 in Additional file 1 for details)

We found two UGT OGs that were significantly expanded in Coccinellidae (Fig. 3A). In the carnivorous Coccinellidae, most of the underlying genes were highly expressed in the gut (50–100%; Additional file 1: Figure S9.7) and exhibited diet-specific DEGs (29–50%; Fig. 3B). OGs of the following families are significantly expanded in Epilachnini (Fig. 3A): two of GDH, two of P450, two of UGT, and one each of ABC, AKR, COE, and GST. Genes from these OGs were highly expressed in the gut (Additional file 1: Figures S9.3, S9.7, S9.11 and S9.15). Experiments on *H. vigintioctopunctata* showed that the expression of many genes from these OGs is diet-dependent (5–29%; Fig. 3B). Such diet-dependent gene expression differences were also found in the carnivorous Coccinellidae (15–85%; Fig. 3B).

#### Immune gene families (see Sect. 10 of Additional file 1 for details)

We found that OG0000225 of a SPI was significantly expanded at the Coccinellidae and Epilachnini nodes and to be contracted at the Coccinellini node (Fig. 3A). Most immune genes (i.e., ATTs, COLs, CLYSs, CWHs DEFs, GNBPs, ILYSs, and PGRPs) were downregulated when the carnivorous ladybirds were fed a suboptimal diet (e.g., moth eggs) (Fig. 3B). Five of 19 genes of the SPI OG0000225 were downregulated in *H. vigintioctopunctata* when fed with a suboptimal diet (sugar water) and five out of nine genes were upregulated in *C. montrouzieri* when fed with a suboptimal diet (i.e., moth eggs, and aphids). The following gene families are unique to ladybirds (Fig. 3A) and show a diet-specific DEG in the carnivorous species (Fig. 3B): CWH (OG0001441), DEF (OG0009673), and ILYS (OG0009100). Among the antimicrobial peptides, the clade C5 of the ATT OG0000273 lost genes in Coccinellini but included genes in most other Coccinellidae (Additional file 1: Figure S10.7). In our experiments with *C. montrouzieri*, we found that three of the four genes of OG0000273 were downregulated when the beetles were fed with a suboptimal diet. Clade C4 of the COL OG0000458 (Fig. 3C) shows unique arrangements at the motif level, with relatively extensive lengths in Coccinellini and other Coccinellidae (33–40 aa, totally 136 aa; Additional file 1: Figure S10.8). Another specific motif was found in genes of carnivorous ladybirds in the DEF OG0009673, which is absent in Epilachnini, resulting in the loss of the functional domain of the defensin (Additional file 1: Figure S10.6).

## Discussion

**Fig. 4:**
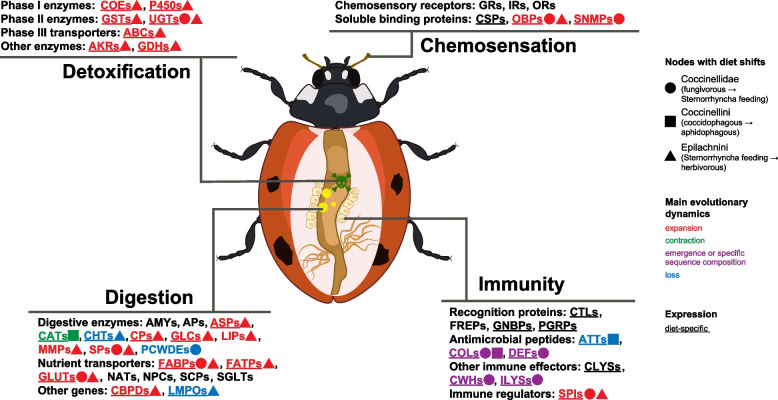
Summary of gene family dynamics associated with dietary shifts in ladybirds at specific nodes. PCWDEs: plant cell wall-degrading enzymes. Candidate gene family abbreviations are listed in the Abbreviations section

Comparison of the genomes and transcriptomes of ladybirds sampled from across their phylogeny and of outgroup beetles, combined with gene expression experiments, provided us with a list of promising candidate genes that are likely to enable the use of different diets by different species. These candidate genes are predicted to be functionally involved in chemosensation, digestion, detoxification, and immunity (Fig. 4) and are discussed in detail below.

Using genomes and transcriptomes across major ladybird lineages, our comparative analysis revealed several genomic signatures of genes related to chemosensation, digestion, detoxification, and immunity, consistent with the molecular adaptation to diet shifts at the nodes of Coccinellidae (fungivorous to an insectivorous diet), Coccinellini (aphidophagous diet) or Epilachnini (herbivorous diet) (Fig. 4).

### Evolution of chemosensory genes in ladybirds possibly related to food searching

We analyzed the gene evolutionary dynamics and transcriptome profiling of the chemosensory genes, including those encoding the chemosensory receptors and soluble binding proteins. OBPs and SNMPs expanded at the node of Coccinellidae, thus possibly in the context of a dietary shift from fungivory to carnivory. OBPs also expanded at the node of Epilachnini and thus possibly in the context with a dietary shift from carnivory to herbivory. In insects, OBPs play a key role in the perception of sex and alarm pheromones as well as of host plant volatiles [101, 102]. SNMPs have been reported to be essential for the perception of fatty acid-derived odorants in *Drosophila* [103]. Carnivorous ladybirds are known to use such chemical cues, for example the aphid alarm pheromone (E)-β-farnesene [104], the mealybug sex pheromone chrysanthemyl 2-acetoxy-3-methylbutanoate [105], and methyl salicylate, which indicates plant damage [106]. OBPs have also been associated with the perception of functionally similar volatiles in carnivorous ladybirds [107–109]. Herbivorous ladybirds are able to locate their host plants through plant volatiles [110]. These adaptative requirements in ladybirds may drive the expansions of the chemosensory gene families, especially OBPs. Interestingly, some of the volatiles can be used by ladybirds to locate both sternorrhynchan insect prey and host plants, and even host fungi, such as (E)-β-farnesene, methyl salicylate, (-)-α pinene, and 1-octen-3-ol [106, 110–113]. It provides a reasonable explanation how diet shift could have evolved without temporary fitness reduction and why expansions at Coccinellidae and Epilachnini nodes could occur in the same OG.

We found that the expression of genes of the soluble binding protein family (i.e., CSPs, OBPs, and SNMPs) was affected by the type of food the beetles were fed. A possible explanation could be an autoregulation of protein abundance in response to the semiochemical environment. Such a mechanism could increase the sensitivity not only to detect volatiles of the preferred diet—and in consequence a better ability to track it [114]—but also to avoid suboptimal diets [114–116].

### Evolution of digestion and detoxification genes in ladybirds likely reflects adaptation to plant components

In the herbivorous tribe Epilachnini, digestion-related OGs (i.e., ASPs, CBPDs, CPs, FABPs, FATPs, GLCs, and GLUTs) have expanded. These genes tend to be highly expressed in the gut and are downregulated in dietary experiments when *H. vigintioctopunctata* is fed sugar water instead of plant leaves. We also found that the digestion-related OGs GLUTs and FABPs are expanded at the node of Coccinellidae, with diet-specific DEGs and high expression in gut in the carnivorous ladybirds. The observed expansions and gene expression likely reflect adaptation to metabolize plant components. For example, the expansion of a GLC has been hypothesized to have been critical for the evolution of herbivory by enabling digestion, or detoxification, of plant cell components [49, 117]. We found that GLUTs and FABPs are expanded at both the Coccinellidae and Epilachnini nodes, which is associated with the switches from fungivory to carnivory and from carnivory to herbivory. This at first glance counterintuitive result may be explained by the specific prey of these ladybirds: sternorrhynchan insects. Their gut may contain plant components (e.g., phytoene, carotenoids) that ladybirds with specific GLUTs and FABPs may be able to digest or detoxify [118]. CBPD, whose expansion we found to be associated with a switch to herbivory, is thought to be required to ensure the structural and functional integrity of the peritrophic membrane in the gut and to influence the digestibility of plant tissues [119–121]. CBPD could thus help herbivorous ladybirds, which have a longer gut than carnivorous ladybirds [27, 28, 73], to digest plant components.

Several of the OGs related to detoxification (i.e., ABC, AKR, COE, GDH, GST, P450, and UGT) have expanded in the tribe Epilachnini. We found that the genes of these OGs were predominantly upregulated in dietary experiments on *H. vigintioctopunctata*, in which individuals were fed plant leaves instead of sugar water, and highly expressed in the gut of the ladybirds. These genes cover all the detoxifying gene families we considered and all the three phases in the insect detoxification enzyme system [122]. Similar results have been reported in studies of other herbivorous beetles [55, 81]. It has been reported that *H. vigintioctomaculata* is not deterred by host plant alkaloid toxins such as α-solanine, α-chaconine, and tomatine [123, 124]. Thus, it appears that Epilachnini can neutralize plant secondary compounds, probably with the help of proteins from the detoxification-related protein families listed above, as has been reported in other herbivorous insects [125, 126].

Although typically associated with benefits for herbivorous species, detoxification-related genes are also important for carnivorous species. Previous studies on ladybirds have already reported major changes in the expression of genes encoding proteins related to detoxification in carnivorous ladybirds in response to different dietary treatments [38, 67, 69]. In our study, we found two UGT OGs to have significantly expanded in Coccinellidae. These two protein families are likely involved in dietary toxin process, as we found them to be expressed primarily in the gut and their expression to respond to dietary treatments. Some toxins (e.g., glycosides, glucosinolates, isothiocyanates, and alkaloids) have been reported from the prey of carnivorous ladybirds, such as aphids and coccids, which are originally acquired from the plant or synthesized using plant components [13, 104, 127], suggesting a need to cope with toxins also in carnivorous ladybirds. Expansion of detoxification-related genes has been previously reported in other carnivorous insects, such as praying mantises [61] and green lacewings [62], indicating a convergent molecular adaptation to toxic prey.

The large numbers of digestive and detoxifying OGs exhibiting expansions in Epilachnini is similar to the pattern found in other herbivorous beetles and insects [48, 54, 56–60, 81, 128–130]. Compared with the carnivorous ladybirds, we found more genes associated with digestion or detoxification in herbivorous ladybirds, suggesting the need for a more sophisticated system of digestion and detoxification to digest plant leaves.

### Loss and contraction of genes related to carnivory and herbivory in ladybirds

We found that most of the PCWDE gene families known from beetles [49, 131, 132], especially the widely existing endocellulase GH9 and pectinase CE13, are absent in Coccinellidae, with the exception of GLC (GH1 domain-contained). The absence of GH9 in Coccinellidae has been reported previously [49]. The different enzymatic requirements for digestion of fungi versus arthropods may account for this widespread loss of PCWDE genes at the base of Coccinellidae.

Two genes related to chitin metabolism (a CHT and an LMPO) are highly expressed in the gut of carnivorous species of the Coccinellidae and are partially lost in Epilachnini. Although LMPOs have been reported to be possibly involved in the digestion of cellulose [133], in the ladybirds it seems possible that these genes play roles in chitin degradation [134–137] based on the above results. Consistent with this idea, we found an upregulation of these two genes in *M. discolor* when fed with an insect diet instead of a pollen diet. Woodring [138] reported the absence of PCWDEs in carnivorous praying mantids and the absence of chitinases in herbivorous stick insects, a pattern reminiscent of that found in ladybirds. This suggests that carnivory and herbivory are associated with predictive enzyme requirements in different insects. However, the lack of PCWDEs, particularly endocellulases and pectinases, has been passed on to the Epilachnini lineage, resulting in an abnormal condition. The PCWDEs in other herbivorous beetles, such as weevils and leaf beetles, could be acquired from fungi through horizontal gene transfer [49]. This suggests that reduced opportunities to horizontally acquire the fungi PCWDEs due to their carnivorous ancestry may be one of the reasons of the absence of most PCWDEs in Epilachnini. The only PCWDE present in Epilachnini, GH1, is thought to act as a hemicellulose. This raises the question of how Epilachnini cope with the lack of PCWDEs, such as the endocellulase GH9, which has synergism with GH1 and is found in most other beetles [49, 139]. These findings suggest that Epilachnini may have adopted alternative strategies to deal with plant cell wall components in their diet compared to other herbivorous beetles. For example, some Epilachnini are known to primarily scrape the soft tissues of plant leaves, chew them, and suck the exposed sap. Thus, Epilachnini leave most of the cellulose on the leaves compared to other leaf-feeding beetles that swallow leaf fragments [16, 140, 141]. In addition, the omnivorous ladybird *M. discolor *was found to harbor putative cellulolytic bacteria [70], and some isolated bacteria from Epilachnini species have the ability to hydrolyze the cellulose, though they are relatively low in abundance [142]. These support a potential role of symbiotic microbes to digest plant cell walls in the ladybirds. We hypothesize that similar mechanical and/or microbial strategies exist in all Epilachnini species.

We found that genes of the family CAT are highly expressed in the gut and exhibit diet-specific expression patterns. These results are in line with the idea that proteins of this family serve critical digestive functions in both carnivorous and herbivorous ladybirds [143–147]. However, we found that this family is significantly contracted in the tribe Coccinellini, whose species feed on aphids instead of coccids. Since research on the coccidophagous ladybird *C. montrouzieri* has shown that the enzymatic activity of CAT is reduced when individuals of this species are fed with aphids [144], we hypothesize that CAT is required to digest a specific component present in coccids or mealybugs.

### Evolution of immune effector genes in carnivorous ladybirds in relation to prey symbionts

Reports linking immunity-related genes to insect feeding habits are rare [64–66]. We have previously found that immune effector genes are downregulated when ladybirds are fed with a suboptimal diet, and that immunity-related gene families are expanded in size in the mealybug predator *C. montrouzieri* [38, 67]. In this study, we also found that several immune effector OGs, such as CWHs, COLs, DEFs, and ILYSs, appear to have evolved de novo in Coccinellidae. In at least one case, CWHs, the genes were obtained by ladybird ancestors from bacteria via horizontal gene transfer [19]. We found that many genes of immunity-related families (i.e., ATTs, CLYSs, COLs, CWHs DEFs, GNBPs, ILYSs, and PGRPs) are downregulated when the ladybirds are fed on diets other than sternorrhynchan insects (e.g., moth eggs). We also found that the SPI immunoregulator gene family is expanded at the Coccinellidae and Epilachnini nodes and includes several genes whose expression is diet-specific in the ladybirds. However, whether serpins play a role in immunity or have other functions (e.g., regulation of secretion, digestion [148]) in the ladybirds remains to be explored.

Ladybirds primarily prey on sternorrhynchan insects, which are known to harbor a diverse set of symbiont bacteria in their bacteriome or in their tissues (e.g., *Tremblaya* and *Moranella* in mealybugs, *Buchnera* and *Serratia* in aphids [149, 150]). These symbionts can protect their hosts from predators [151, 152] or colonize and thereby harm predators [150]. It is therefore reasonable to assume that ladybirds have evolved strategies to cope with these bacteria. We interpret the higher expression of genes from several immunity-related families when ladybirds are fed sternorrhynchan insects compared to alternative diets as a possible indication of an immune response against bacteria.

Compared to genes in families related to chemosensation, digestion, and detoxification, we found few lineage-specific changes in family size via expansion or loss and diet-specific changes in expression of genes related to immunity (Fig. 2). We found that dietary shifts were associated primarily by de novo emergence and amino acid changes in immunity effector genes. This is similar to the evolutionary mechanisms of antimicrobial peptides in *Drosophila* that are driven by the bacteria in the flies’ diet [66].

## Conclusions

Ladybirds serve as a promising model group to study of the evolution of dietary shifts in insects, with relevance for agricultural activities and ecosystems in general. In this study, we shed light on the molecular mechanisms underlying dietary shifts in ladybirds, in particular the transitions from fungivory to carnivory and from carnivory to herbivory. This was achieved by applying a combination of comparative genomic analyses using the new genomes of four ladybird species and 21 published ladybird and outgroup beetle genomes, as well as the transcriptomes of 62 related species, together with diet- and tissue-specific transcriptome profiling representing the major feeding guilds: carnivores versus herbivores. Our study revealed diet-shift-specific patterns in the evolution of multigene families related to chemosensation, digestion, detoxification, and immunity. Specifically, we found notable changes in the gene repertoires at phylogenetic nodes with diet shifts from fungi to sternorrhynchan insects (Coccinellidae), from sternorrhynchan insects to plants (Epilachinini), and from coccids to aphids (Coccinellini).

In Coccinellidae, the evolutionary dynamics of gene families related to chemosensation (expansion of OBPs and SNMPs), digestion (loss of PCWDEs and expansion of GLUTs and FABPs), detoxification (expansion of UGTs), and immunity (emergence or specific sequence composition of immune effector genes) can be seen as possible adaptations to foraging, digestion, and the management of toxins and symbiotic bacteria during the dietary transition from fungi to sternorrhynchan insects. In Coccinellini, the reduction in size of the CAT digestive gene family could be a consequence of significant differences in protein components between aphids and coccids, while the loss of a specific clade of attacins and the specific sequence composition of coleoptericin could have been driven by symbiotic bacteria in aphids serving as prey. In Epilachnini, changes in gene families related to chemosensation (expansion of OBPs), digestion (loss of chitin-related genes, expansion of ASPs, CBPDs, CPs, FABPs, FATPs, GLCs, GLUTs), and detoxification (expansion of almost all gene families) could represent adaptations to perceive volatiles of, digest, and cope with toxins in plants.

However, to make the model system of dietary shifts in the ladybirds more complete and precise, our hypotheses on the function of specific gene families should be further supported by experimental verifications. In addition, the genomes of the ladybirds with other feeding habits not covered in our research are worthy to be studied in the future. For example, the genomes of the fungivorous ladybirds (e.g., *Illeis* and *Halyzia*) would provide insights into the dynamics during the back transition from carnivory to fungivory, and the genomes of non-sternorrhyncha carnivores (e.g., *Stethorus*) could be used to investigate the adaptive mechanisms to sternorrhynchan prey in the ladybirds in comparison with those of sternorrhyncha-feeding carnivores. Molecular evolution of metabolizing specific diet components with different contents (e.g., different chitins in insects and fungi, cellulose and toxins in different host plants, wax covered on prey and used as camouflage by ladybird larvae) during the dietary shifts of ladybirds could be explored more deeply, considering the global gene pathways and even the symbiotic microbiomes.

## Methods

We generated a chromosome-level assembly from an already existing *C. montrouzieri* contig-level genome assembly [38] using Hi-C technology. We de novo sequenced the genomes of *M. discolor* and *H. vigintioctopunctata* using a combination of Oxford Nanopore long read and Illumina short-read DNA sequencing technologies and the *C. impunctata* genome using a combination of PacBio, 10X Genomics, and Illumina DNA sequencing technologies. We used the following software packages to assemble the read data from these genomes: ARCS v. 1.2.4 [153], Canu v. 1.5 [154], LACHESIS [155], NextDenovo v. 2.5.0 [156], NextPolish v. 1.4.1 [157], Pilon v. 1.21 [158], Racon v. 1.32 [159], and wtdbg v. 2.5 [160]. Other published genomes of the ladybirds before our analyses were used in our research (Table 1). Our research included the genome of *H. vigintioctomaculata* close to *H. vigintioctopunctata*, because it has higher assembly level and potentially more complete gene contents related to herbivory. The completeness of the protein-coding gene space of the resulting assemblies was assessed using the BUSCO v. 5.2.2 pipeline [71] and specifying the Insecta ortholog set of OrthoDB v. 10 [72]. Structural and functional annotation of protein-coding genes in the genome assemblies was performed using the FunAnnotate v. 1.8.1 pipeline [161] and applying the procedures described by Tang, Huang [39]. Coccinelloidea transcriptomes of whole individuals were sequenced using Illumina short-read DNA sequencing technology, and non-redundant transcriptomic protein sets were inferred using EvidentialGene v. 2018.06.18 [162]. Ortholog group assignment and annotation were performed by OrthoFinder v2.5.4 [163] and KinFin v1.0.3 [164].

We inferred a species tree of the investigated taxa from the phylogenetic signal contained in 770 near single-copy protein-coding genes (including the single-copy OGs in the genome dataset and the OGs selected for phylogenetic reconstruction by OrthoFinder in 87 species) using IQ-TREE v. 2.1.4-beta [95]. The best models and partitions were selected using ModelFinder [165] in IQ-TREE with minimum Bayesian information criterion (BIC) score. The adephagan beetle *Nebria ingens* was selected to root the tree. Divergence times were estimated using MCMCTREE in PAML v. 4.8a [96] and the age of eleven fossils for calibration (Additional file 1: Table S2.1). Dietary evolution was traced on the inferred phylogeny using the fitMk function in the R package phytools [97].

CAFE v. 5.0 [99] was used to identify contractions and expansions of OGs of species with sequenced genomes, using the genome dataset except *P. japonica* due to its species-specific high gene duplication level of 20.0%, which directly affects gene count data. The lineage-specific OGs and linage-absent OGs were also identified as de novo emergences and losses from the whole subgroups. For the OGs related to chemosensation, digestion, detoxification, and immunity of interest, we also considered clade-level dynamics, and thought clade-specific gene duplications, de novo gene emergence and loss as supplementary of expansion, and de novo gene emergence and loss in OG size. Phylogenies of OGs were inferred using IQ-TREE [95]. The inferred phylogenies served as the basis for identifying ladybird clades, as well as taxonomic clade-specific gene duplications and de novo gene emergence or loss using the OG tree reconciliation implemented in OrthoFinder [163]. Motif analysis of the amino acid sequences of the 25 beetle genomes was performed using MEME v. 5.4.1 [166]. Pfam domains reported in previous studies were used to identify PCWDEs [49, 131, 132].

Ladybird samples of different diet treatments or different tissues were prepared and sequenced using Illumina platform. Abundance estimation and DEG detection were performed by HISAT2 v2.2.0 [167], StringTie v2.1.4 [168], and DESeq2 [169]. For tissue-specific expression, an upregulated DEG detected in a specific tissue group compared with other tissue groups were considered as a gene highly expressed in this tissue. Enrichment was performed using clusterProfiler package [100] and a customized annotation based on our gene family identification. In order to avoid biases of the enrichment results, we also added the Pfam annotations of other OGs or genes into the enrichments.

More detailed information on the procedures used, as well as additional results and discussion, can be found in Additional file 1.

## Supplementary Information

Additional file 1. Supplementary Text and Supplementary Figures: Section 1: Detailed methods, results, discussions, tables S1.1-1.2, and figures S1.1-1.4 of taxon selection, data preparation, and genome description. Section 2: Detailed methods, results, discussions, table S2.1, and figures S2.1-2.6 of species phylogeny. Section 3: Detailed methods, results, discussions, table S3.1, and figures S3.1-3.4 of selection pressure of single-copy genes. Section 4: Detailed methods, results, discussions, and figures S4.1-4.5 of gene count evolution of ortholog groups. Section 5: Detailed methods, results, discussions, tables S5.1-5.4, and figures S5.1-5.7 of diet-specific transcriptome comparison. Section 6: Detailed methods, results, discussions, table S6.1, and figures S6.1-6.7 of tissue-specific transcriptome comparison. Section 7: Detailed methods, results, discussions, tables S7.1-7.3 and figures S7.1-7.4 of evolution of candidate chemosensory genes. Section 8: Detailed methods, results, discussions, tables S8.1-8.2, and figures S8.1-8.16 of evolution of candidate genes related to nutrient digestion. Section 9: Detailed methods, results, discussions, tables S9.1-9.2, and figures S9.1-9.16 of evolution of candidate genes related to detoxification. Section 10: Detailed methods, results, discussions, tables S10.1-10.2 and figures S10.1-10.8 of evolution of candidate genes related to immunity.

Additional file 2. Table SE1. Information on Coccinellidae and other Coleoptera taxa analyzed in this study. Table SE2: Information on the transcriptomes analyzed in this study. Table SE3: Ortholog groups (OGs) under selection, significant expansions and contractions, clade-specific gene loss, or lineage-specific *de novo* gene emergence based on our genomic and transcriptomic data. Table SE4: Results of ortholog group (OG) enrichment analysis of differentially expressed genes (DEGs) in six carnivorous ladybird species when treated with different diets. Table SE5: Gene expression in diet- and tissue-specific transcriptomes. Table SE6: Candidate ortholog groups (OG) associated with chemosensation, detoxification, digestion, and immunity.

## Data Availability

Raw reads from the genome and transcriptome sequencing were deposited at NCBI (BioProject accessions: PRJNA626074, PRJNA509782, PRJNA549114, PRJNA776094, PRJNA956151, PRJNA956140, PRJNA956138, PRJNA956078, PRJNA955835, and PRJNA967842).

## Abbreviations

ABC: ATP-binding cassette transporter (detoxification)
AKR: Aldo-keto reductase (detoxification)
AMY: α-Amylase (digestion)
AP: Aminopeptidase (digestion)
ASP: Aspartic proteinases (digestion)
ATT: Attacin (immunity)
CAT: Cysteine proteinase (digestion)
CBPD: Chitin binding Peritrophin-A domain containing protein (digestion)
CLYS: C-type lysozyme (immunity)
COE: Carboxylesterase (detoxification)
COL: Coleoptericin (immunity)
CP: Carboxypeptidase (digestion)
CSP: Chemosensory protein (chemosensation)
CTL: C-type lectin (immunity)
CWH: Cell wall hydrolase (immunity)
DEF: Defensin (immunity)
FABP: Fatty acid binding protein (digestion)
FATP: Fatty acid transport protein (digestion)
FREP: Fibrinogen-related protein (immunity)
GDH: Glucose dehydrogenase (detoxification)
GLC: α/β-Glucosidase (digestion)
GLUT: Glucose transporter protein (digestion)
GNBP: Gram-negative binding protein (immunity)
GR: Gustatory receptor (chemosensation)
GST: Glutathione S-transferase (detoxification)
ILYS: I-type lysozyme (immunity)
IR: Ionotropic receptor (chemosensation)
LIP: Lipase (digestion)
LPMO: Lytic polysaccharide mono-oxygenase (digestion)
MMP: Metalloproteinase (digestion)
NAT: Nutrient amino acid transporter (digestion)
NPC: Niemann-Pick C1 (digestion)
OBP: Odorant-binding protein (chemosensation)
OR: Odorant receptor (chemosensation)
P450: Cytochrome P450 (detoxification)
PGRP: Peptidoglycan recognition protein (immunity)
SCP: Sterol carrier protein (digestion)
SGLT: Sodium-driven glucose symporter (digestion)
SNMP: Sensory neuron membrane protein (chemosensation)
SP: Serine proteinase (digestion)
SPI: Serine protease inhibitor, serpin (immunity)
UGT: UDP-glucuronosyltransferase (detoxification)
