# Supplementary material for: Molecular evolution of dietary shifts in ladybird beetles (Coleoptera: Coccinellidae): from fungivory to carnivory and herbivory
Source: BMC Biol. 2025 Feb 28;23:67. doi: 10.1186/s12915-025-02174-2 (PMC11871716; doi:10.1186/s12915-025-02174-2)
Supplement: Supplementary file 1 — Additional file 1. Supplementary Text and Supplementary Figures: Section 1: Detailed methods, results, discussions, tables S1.1-1.2, and figures S1.1-1.4 of taxon selection, data preparation, and genome description. Section 2: Detailed methods, results, discussions, table S2.1, and figures S2.1-2.6 of species phylogeny. Section 3: Detailed methods, results, discussions, table S3.1, and figures S3.1-3.4 of selection pressure of single-copy genes. Section 4: Detailed methods, results, discussions, and figures S4.1-4.5 of gene count evolution of ortholog groups. Section 5: Detailed methods, results, discussions, tables S5.1-5.4, and figures S5.1-5.7 of diet-specific transcriptome comparison. Section 6: Detailed methods, results, discussions, table S6.1, and figures S6.1-6.7 of tissue-specific transcriptome comparison. Section 7: Detailed methods, results, discussions, tables S7.1-7.3 and figures S7.1-7.4 of evolution of candidate chemosensory genes. Section 8: Detailed methods, results, discussions, tables S8.1-8.2, and figures S8.1-8.16 of evolution of candidate genes related to nutrient digestion. Section 9: Detailed methods, results, discussions, tables S9.1-9.2, and figures S9.1-9.16 of evolution of candidate genes related to detoxification. Section 10: Detailed methods, results, discussions, tables S10.1-10.2 and figures S10.1-10.8 of evolution of candidate genes related to immunity. [file 12915_2025_2174_MOESM1_ESM.docx]

Additional file 1

Molecular evolution of dietary shifts in ladybird beetles (Coleoptera, Coccinellidae): from fungivorous to carnivorous and herbivorous

Yu-Hao Huang^1^, Hermes E. Escalona^2^, Yi-Fei Sun^1^, Pei-Fang Zhang^1^, Xue-Yong Du^1^, Sen-Rui Gong^1^, Xue-Fei Tang^1,3^, Yuan-Sen Liang^1^, Dan Yang^1^, Pei-Tao Chen^1^, Huan-Ying Yang^1^, Mei-Lan Chen^4^, Bruno Hüttel^5^, Ondrej Hlinka^6^, Xingmin Wang^7^, Karen Meusemann^8^, Adam Ślipiński^2^, Andreas Zwick^2^, Robert M. Waterhouse^9^, Bernhard Misof^8^, Oliver Niehuis^10^, Hao-Sen Li^1*^, Hong Pang^1*^

1. State Key Laboratory of Biocontrol, School of Ecology, Sun Yat-sen University, Shenzhen 518107, China

2. Australian National Insect Collection, CSIRO, GPO Box 1700, Canberra, ACT 2601, Australia

3. College of Forestry, Henan Agricultural University, Zhengzhou 450002, China

4. School of Environment and Life Science, Nanning Normal University, Nanning 530001, China

5. Max Planck Genome Centre Cologne, Max Planck Institute for Plant Breeding Research, Cologne, Germany

6. CSIRO Information, Management and Technology, Pullenvale, Queensland, Australia

7. College of Plant Protection, South China Agricultural University. Guangzhou 510642, China

8. Leibniz Institute for the Analysis of Biodiversity Change, Adenauerallee 127, 53113 Bonn, Germany

9. Department of Ecology and Evolution, University of Lausanne and Swiss Institute of Bioinformatics, 1015 Lausanne, Switzerland

10. Department of Evolutionary Biology and Ecology, Institute for Biology I (Zoology), University of Freiburg, 79104 Freiburg, Germany

* Corresponding author: lihaosen3@mail.sysu.edu.cn; lsshpang@mail.sysu.edu.cn

Content

[1 Taxon selection, data preparation and genome description 4](#_Toc148913141)

[1.1 Materials and methods 4](#_Toc148913142)

[1.1.1 Taxon selection, data collection and dataset setting 4](#_Toc148913143)

[1.1.2 DNA extraction, genome sequencing and assembly of *M. discolor* and *H. vigintioctopunctata* 4](#_Toc148913144)

[1.1.3 DNA extraction, genome sequencing and assembly of *C. impunctata* 5](#_Toc148913145)

[1.1.4 Hi‐C library construction and chromosome assembly of *C. montrouzieri* 6](#_Toc148913146)

[1.1.5 Genome refinement, gene prediction and functional annotation 7](#_Toc148913147)

[1.1.6 Transcriptome sequencing and transcriptomic protein set construction 8](#_Toc148913148)

[1.1.7 Ortholog group assignment and annotation 9](#_Toc148913149)

[1.1.8 Genome synteny analysis 9](#_Toc148913150)

[1.2 Results 10](#_Toc148913151)

[1.2.1 General genomic features 10](#_Toc148913152)

[1.2.2 General features of ortholog groups and datasets 10](#_Toc148913153)

[1.2.3 Genome synteny 11](#_Toc148913154)

[1.3 Summary 13](#_Toc148913155)

[2 Species phylogeny 23](#_Toc148913156)

[2.1 Materials and methods 23](#_Toc148913157)

[2.1.1 Phylogeny inference 23](#_Toc148913158)

[2.1.2 Divergence time estimation 23](#_Toc148913159)

[2.1.3 Ancestral character reconstruction 24](#_Toc148913160)

[2.2 Results 24](#_Toc148913161)

[2.2.1 Phylogenetic tree of 69 ladybird species and 18 outgroup species 24](#_Toc148913162)

[2.2.2 Divergence time of the ladybirds 26](#_Toc148913163)

[2.2.3 Ancestral character reconstruction of the ladybird diets 26](#_Toc148913164)

[2.3 Summary 27](#_Toc148913165)

[3 Selection pressure of single-copy genes 39](#_Toc148913166)

[3.1 Materials and methods 39](#_Toc148913167)

[3.2 Results 40](#_Toc148913168)

[3.3 Summary 41](#_Toc148913169)

[4 Gene count evolution of ortholog groups 50](#_Toc148913170)

[4.1 Materials and methods 50](#_Toc148913171)

[4.2 Results 50](#_Toc148913172)

[4.3 Summary 52](#_Toc148913173)

[5 Diet-specific transcriptome comparison 60](#_Toc148913174)

[5.1 Materials and methods 60](#_Toc148913175)

[5.1.1 Insect rearing and sample collection 60](#_Toc148913176)

[5.1.2 Transcriptome sequencing and analysis 60](#_Toc148913177)

[5.2 Results 61](#_Toc148913178)

[5.2.1 Diet-specific differentially expressed genes in six carnivorous ladybirds 61](#_Toc148913179)

[5.2.2 Diet-specific differentially expressed genes in the herbivorous ladybird *Henosepilachna vigintioctopunctata* 63](#_Toc148913180)

[5.3 Summary 64](#_Toc148913181)

[6 Tissue-specific transcriptome comparison 78](#_Toc148913182)

[6.1 Materials and methods 78](#_Toc148913183)

[6.2 Results 78](#_Toc148913184)

[6.3 Summary 80](#_Toc148913185)

[7 Evolution of candidate chemosensory genes 90](#_Toc148913186)

[7.1 Materials and methods 90](#_Toc148913187)

[7.2 Results 91](#_Toc148913188)

[7.2.1 Chemosensory receptors: GR, IR, OR 91](#_Toc148913189)

[7.2.2 Soluble binding protein: OBP, CSP, and SNMP 92](#_Toc148913190)

[7.3 Summary 93](#_Toc148913191)

[8 Evolution of candidate genes related to nutrient digestion 103](#_Toc148913192)

[8.1 Materials and methods 103](#_Toc148913193)

[8.2 Results 104](#_Toc148913194)

[8.2.1 Candidate genes encoding digestive enzymes 104](#_Toc148913195)

[8.2.2 Candidate genes encoding nutrient transporters 107](#_Toc148913196)

[8.2.3 Other candidate genes related to digestion 108](#_Toc148913197)

[8.3 Summary 109](#_Toc148913198)

[9 Evolution of candidate genes related to detoxification 139](#_Toc148913199)

[9.1 Materials and methods 139](#_Toc148913200)

[9.2 Results 139](#_Toc148913201)

[9.2.1 Phase I: P450 and COE 139](#_Toc148913202)

[9.2.2 Phase II: GST and UGT 141](#_Toc148913203)

[9.2.3 Phase III: ABC 141](#_Toc148913204)

[9.2.4 Other candidate detoxification enzymes: AKR and GDH 142](#_Toc148913205)

[9.3 Summary 143](#_Toc148913206)

[10 Evolution of candidate genes related to immunity 163](#_Toc148913207)

[10.1 Materials and methods 163](#_Toc148913208)

[10.2 Results 163](#_Toc148913209)

[10.2.1 Recognition protein: PGRP, GNBP, FREP, CTL 163](#_Toc148913210)

[10.2.2 Antimicrobial peptide: Attacin, Defensin, Coleoptericin 164](#_Toc148913211)

[10.2.3 Lysozyme and Cell Wall Hydrolase 164](#_Toc148913212)

[10.2.4 Serpin 165](#_Toc148913213)

[10.3 Summary 165](#_Toc148913214)

[Excel table information 179](#_Toc148913215)

[Reference 180](#_Toc148913216)

# 1 Taxon selection, data preparation and genome description

## 1.1 Materials and methods

### 1.1.1 Taxon selection, data collection and dataset setting

We selected six published ladybird genomes, including *Harmonia axyridis* (Boyes et al., 2021), *Coccinella septempunctata* (Crowley et al., 2021b), *Adalia bipunctata* (Wellcome Sanger Institute Tree of Life programme et al., 2022), *Propylea japonica* (Zhang et al., 2020b), *Novius pumilus* (Tang et al., 2022b) and *Henosepilachna vigintioctomaculata* (Zhu et al., 2023), from the National Center for Biotechnology Information (NCBI) Reference Sequence (RefSeq) (Li et al., 2021c) or Genome database or self-provided datasets. For *H. axyridis* and *C. septempunctata* with several genomes published (Ando et al., 2018; Boyes et al., 2021; Chen et al., 2021; Crowley et al., 2021b; Gautier et al., 2018; Shen and Zhou, 2023), we only used the chromosome-level genomes with RefSeq annotations and highest completeness and continuity (Boyes et al., 2021; Crowley et al., 2021b). In addition to these published genomes, we assembled a previously published contig-level genome of *Cryptolaemus montrouzieri* (Li et al., 2021a) into chromosome-level. We also newly sequenced the genomes of an omnivorous ladybird beetle (*Micraspis discolor*) and two herbivorous ladybirds (*Henosepilachna vigintioctopunctata* and *Cynegetis impunctata*). We also selected 15 published outgroup Coleoptera genomes (Crowley et al., 2021a; Cunningham et al., 2015; Evans et al., 2018; Fallon et al., 2018; Herndon et al., 2020; Keeling et al., 2022; Keeling et al., 2013; King et al., 2023; McKenna et al., 2016; Parisot et al., 2021; Richards et al., 2008; Schoville et al., 2018; Thomas et al., 2020; Wang et al., 2022; Weng et al., 2021; Zhang et al., 2023) from NCBI RefSeq/Genome database, InsectBase 2.0 (Mei et al., 2022) or self-provided datasets (Additional file 2: Table SE1). To extend the knowledge to more ladybird species, we included the transcriptome data from 37 published Coccinelloidea transcriptomes (Allen, 2015; Li et al., 2021b; McKenna et al., 2019; Nadeau et al., 2022; Vizán-Rico et al., 2019; Yuan et al., 2023) in NCBI Sequence Read Archive (SRA) database and 25 newly sequenced Coccinelloidea species (including seven from 1KITE project (http://1kite.org/)) in the extended dataset (Additional file 2: Table SE1, Figure S1.1).

### 1.1.2 DNA extraction, genome sequencing and assembly of *M. discolor* and *H. vigintioctopunctata*

DNA was extracted from the whole body of ~20 female adults of *M. discolor* and ~10 male adults of *H. vigintioctopunctata*, separately, using the CTAB method (Milligan, 1988). The *M. discolor* individuals were from a population that was originally collected in 2018 from Hainan Province, China, and have been maintained with a mixed diet consisting of eggs of the flour moth *Ephestia kuehniella* and pollen of rape, *Brassica campestris*. The *H. vigintioctopunctata* individuals were from a population that was originally collected in 2020 from Guangzhou, China, and have been maintained with the leaves of *Solanum melongena*. Both these two populations were reared under laboratory conditions (27 ± 1 °C, 80 ± 1% relative humidity (RH) and a 14:10 (L:D) h photoperiod) at Sun Yat-sen University. The quality and concentration of the extracted genomic DNA were checked using 1% agarose gel electrophoresis and a Qubit fluorimeter (Invitrogen, Carlsbad, CA, USA) to ensure high-quality DNA was used for subsequent sequencing. Approximately 15 μg genomic DNA was used to generate Oxford Nanopore long reads via PromethION DNA sequencer (Oxford Nanopore, Oxford, UK).

For *M. discolor*, we filtered raw data to remove short sequence reads (< 5kb) and low-quality reads (Q30 < 90%) using Nanofilt v2.3.0 (De Coster et al., 2018). We then used Canu v1.5 (Koren et al., 2017) to generate more accurate self-corrected reads with a corrected error rate of 0.05 for assembly of Nanopore sequencing data. Assembly was then performed by wtdbg v2.2 (Ruan and Li, 2020) with default settings, followed by Racon v1.32 (Vaser et al., 2017) correction with Nanopore reads through two rounds with default settings. To further correct errors in the genome assembly, genomic DNA was also sequenced on the Illumina HiSeq X Ten platform (Illumina, San Diego, CA, USA). The Illumina-sequenced data were filtered to remove low-quality bases and adapters using Trimmomatic v0.36 (Bolger et al., 2014) with default settings. Finally, Pilon v1.21 (Walker et al., 2014) was implemented to correct the assembled genome from Nanopore sequencing data with Illumina reads through three rounds with default settings for additional error correction purposes.

For *H. vigintioctopunctata*, we called and filtered raw Nanopore reads to remove low-quality reads (Qmean < 7) using Guppy v4.0.2 (Oxford Nanopore Technologies). NextDenovo v2.5.0 (Hu et al., 2023) was then used to correct and assemble the reads. To further improve assembly accuracy, Racon v1.4.11 (Vaser et al., 2017) was implemented for two rounds to correct the assembly with Nanopore reads with default settings. Additionally, genomic DNA was also sequenced on the Illumina NovaSeq 6000 platform (Illumina, San Diego, CA, USA). The Illumina-sequenced data were filtered to remove low-quality bases (percent of Ns > 5% or Q5 < 50%) and adapters using FastQC v0.20.1 (https://www.bioinformatics.babraham.ac.uk/projects/ fastqc/). Finally, Pilon v1.23 (Walker et al., 2014) was implemented for two rounds with default settings to correct errors in the genome assembly from Nanopore-sequencing data with Illumina reads for additional error correction purpose.

To prevent potential contamination in the primary genome assembly, such as sequences from symbiotic bacteria, we used BlobTools v1.1.1 (Laetsch and Blaxter, 2017a) to identify foreign sequences based on GC content and short-read coverage information obtained from the BWA-MEM2 v2.2.1 (Vasimuddin et al., 2019) alignments. We also searched for matches in the NCBI nucleotide sequence (NT) database using BLAST v2.8.1+ (Camacho et al., 2009) with an E-value cutoff of 10^−5^ to manually checked matched hits of contigs, removing any identified bacterial-derived contigs from the assemblies. The completeness of the assembly was evaluated using Benchmarking Universal Single-Copy Orthologs (BUSCO) v5.2.2 pipeline (Manni et al., 2021) with the Insecta set of OrthoDB v10 database (Kriventseva et al., 2019). The completeness after contamination removal was compared with that before removal to ensure that there was no substantial decrease in the completeness level achieved by filtering out bacterial-derived contigs during this process.

### 1.1.3 DNA extraction, genome sequencing and assembly of *C. impunctata*

For *C. impunctata*, we used a different sequencing and assembly due to differences in laboratory resources. DNA was extracted from 2-3 pupae of *C. impunctata* for each library of different sequencing method. Libraries were prepared using a standard PacBio library preparation protocol (Pacific Biosciences) and the genome sequencing was performed on the PacBio RS II sequencing platform. The initial filtered PacBio subreads (< 1kb) were self-corrected by NextDenovo v2.5.0 (Hu et al., 2023). In addition, Pacific Biosciences HiFi circular consensus sequencing (CCS) and 10X Genomics DNA sequencing libraries were constructed according to the manufacturers’ instructions with Pacific Biosciences SEQUEL II and Illumina HiSeq X instruments, respectively. The corrected PacBio sequences along with CCS sequences were assembled using wtdbg v2.5 (Ruan and Li, 2020). We then processed the 10X Genomics linked reads using Long Ranger pipeline v2.2.2 (Marks et al., 2019) for genome scaffolding through ARCS v1.2.4 (Yeo et al., 2018). To polish the assembly, we mapped Illumina reads sequenced on HiSeq X Ten platform to scaffolds and corrected the assembly mistakes through three iterations followed by two more iterations alongside both PacBio CCS reads as well as Illumina reads together via NextPolish v1.4.1 (Hu et al., 2020) with default parameters. We applied similar contamination removal methods along with completeness estimation techniques using in *M. discolor* and *H. vigintioctopunctata* genomes analysis for this particular genome assembly project.

### 1.1.4 Hi‐C library construction and chromosome assembly of *C. montrouzieri*

To prepare Hi-C libraries for *C. montrouzieri*, we used newly hatched first instar larvae and constructed Hi-C fragment libraries with 300-700 bp insert size as described by Rao et al. (2014). The libraries were then sequenced using Illumina platform. Briefly, adapter sequences and low-quality paired-end raw reads were trimmed and removed to obtain clean data. These clean Hi-C reads were truncated at putative Hi-C junctions before aligning them to the primary assembly presented previously (Li et al., 2021a) with BWA v0.7.10-r789 (Li and Durbin, 2009). We retained only uniquely alignable paired-end reads whose mapping quality was more than 20 for further analysis. Invalid read pairs such as dangling-end and self-cycle, re-ligation and dumped products were filtered out using HiC-Pro v2.10.0 pipeline (Servant et al., 2015).

Before chromosomes assembly, we performed preassembly error correction of scaffolds by splitting them into segments averaging around 50 kb, followed by mapping the Hi-C data onto these segments using BWA aligner. The uniquely mapped data was then retained to perform assembly. Any two inconsistent connected segments from raw scaffold information were manually checked for corrections. These corrected scaffolds were assembled with LACHESIS (Burton et al., 2013) , with parameters set at CLUSTER_MIN_RE_SITES, 76; CLUSTER_MAX_LINK_DENSITY, 2; ORDER_MIN_N_RES_IN_TRUNK, 67; ORDER_MIN_N_RES_IN_SHREDS, 66. Afterword, the placement and orientation errors exhibiting obvious discrete chromatin interaction patterns were manually adjusted.

The resulting chromosome-level scaffolds were equally divided into bins measuring approximately 300,000-bp bins. A heatmap was generated based on interaction signals between bins, which were revealed by valid mapped read pairs. Finally, the chromosome-level assembly was evaluated using BUSCO as described above.

### 1.1.5 Genome refinement, gene prediction and functional annotation

Genome refinement, gene prediction and functional annotation were conducted in all the ten ladybird genomes and the genome of *D. helophoroides*, using a modified FunAnnotate v1.8.1 (https://github.com/nextgenusfs/funannotate) pipeline with the same procedures as Tang et al. (2022b). For *H. axyridis* and *C. septempunctata*, our annotations were only used for evaluation and comparison in this section. Instead, in the downstream analyses of these two species, RefSeq annotations downloaded from NCBI were used for downstream analyses, with more transcriptomic data involved and wide usage in the researches.

First, duplicated contigs were removed by the “clean” module in the FunAnnotate pipeline using Minimap2 v2.17 (Li, 2018) to align the shorter contigs to the longer contigs one by one and removed the shorter contigs if identity and coverage of alignments were both above 95%. Contigs with less than 500 bases were also excluded. The rest contigs were then sorted by length and renamed.

The repetitive elements of the prepared genome were identified and masked through the FunAnnotate “mask” module. Repetitive elements were predicted by RepeatModeler v1.0.11 (http://www.repeatmasker.org/RepeatModeler/), followed by soft masking with RepeatMasker v4.0.9 (http://www.repeatmasker.org/RepeatMasker/).

RNA-Seq data from each species at different life stages and treatments (Table S1.1) were mapped to genomes using HISAT2 v2.2.0 (Kim et al., 2019), then used to generate a genome-guided assembly through Trinity v2.8.5 (Grabherr et al., 2011) and StringTie v2.1.4 (Kovaka et al., 2019). Subsequently, PASA v2.4.1 (Haas et al., 2003) assembly and prediction were performed with Minimap2, GMAP v2019-03-15 (Wu and Watanabe, 2005) and BLAT v36x2 (Kent, 2002) as aligners and TransDecoder v5.5.0 (https://github.com/TransDecoder/TransDecoder) as coding sequence (CDS) region predictor through the FunAnnotate “train” module, setting the maximum intron length to 20,000 bp.

Gene models were generated by the consensus of various prediction methods through the FunAnnotate “predict” module. *ab initio* gene predictions were conducted by SNAP v2006-07-28 (Korf, 2004), AUGUSTUS v3.3.3 (Stanke et al., 2008), GlimmerHMM v3.0.4 (Majoros et al., 2004), GeneMark-ET v4.46 (Lomsadze et al., 2014) and the ET mode of BRAKER v2.1.1 (Bruna et al., 2021). HISAT2 RNA-seq alignments generated above were used to train GeneMark-ET and AUGUSTUS, followed by AUGUSTUS optimization. Minimap2, GMAP and BLAT spliced alignments generated in the PASA assembly above were used as transcript evidence. Protein evidence was obtained via Exonerate v2.4.0 (Slater and Birney, 2005) alignment after a DIAMOND v2.0.4 (Buchfink et al., 2015) search against a customized protein database, which was comprised of all the proteins in UniProtKB/Swiss-Prot (Boutet et al., 2016) and the proteins of the species under Arthropoda in the NCBI protein non-redundant (NR) database. Additionally, a high-quality GMAP “gff3_gene” prediction was conducted using complete CDSs from TransDecoder prediction of the genome-guided transcript assembly obtained above in the “train” module, with identity >= 95%, coverage = 100% and completely consistent CDS regions. Together with the PASA/TransDecoder prediction, all these predictions were submitted to EVidenceModeler (EVM) v1.1.1 (Haas et al., 2008) to obtain final gene models. Repeats were used in EVM consensus model building. The maximum intron length was set as 20,000, and the long introns with more than 500 bp were searched again to find the nested genes.

PASA was used to capture untranslated regions (UTRs) and refine gene models through the FunAnnotate “update” module. Only alternative transcripts with more than 10% expression in kallisto v0.46.0 (Bray et al., 2016) pseudoalignments compared to the highest transcripts were retained. Problematic gene models were fixed in the FunAnnotate “fix” module.

Newly predicted proteins from the ladybird genomes and those extracted from studied outgroup genomes were annotated using the FunAnnotate “annotate” module. Domains within the proteins were searched against Pfam v33.1 (Mistry et al., 2021) database using HMMER v3.3 (Mistry et al., 2013) and InterPro v71.0 (Mitchell et al., 2019) database using InterProScan v5.32 (Jones et al., 2014). EggNOG and COG annotations were obtained through EggNOG-mapper v2.0.0 (EggNOG v5.0) (Cantalapiedra et al., 2021; Huerta-Cepas et al., 2019). Gene Ontology (GO) (Carbon et al., 2021) and Kyoto Encyclopedia of Genes and Genomes (KEGG) (Kanehisa et al., 2021) pathway annotations were obtained by combining the results from InterProScan and EggNOG-mapper. UniProtKB/Swiss-Prot v2020_05 database was searched with DIAMOND. Protein names were then identified by combining UniProtKB/Swiss-Prot and EggNOG-mapper results. In addition, BUSCO (Simao et al., 2015) annotations with the Endopterygota set of OrthoDB v9 database (Zdobnov et al., 2017), CAZy/dbCAN v8.0 (Drula et al., 2022; Yin et al., 2012) annotations via HMMER and MEROPS v12.0 (Rawlings et al., 2018) annotations via DIAMOND were generated. Secreted signal peptides and transmembrane structures were predicted by SignalP v5.0 (Armenteros et al., 2019b) and Phobius v1.01 (Kall et al., 2004). All E-value cutoffs above are default values used in FunAnnotate. In addition to the above FunAnnotate annotations, mitochondrial transit peptides were predicted by TargetP v2.0 (Armenteros et al., 2019a).

### 1.1.6 Transcriptome sequencing and transcriptomic protein set construction

Gene sets of 62 Coccinelloidea species in the extended dataset were predicted from the 37 published and 25 new transcriptomes. Adult individuals of the eighteen newly sequenced Coccinelloidea species (Additional file 2: Table SE1) were collected from the field and immediately frozen in liquid nitrogen or at −80 °C prior to RNA extraction. RNA was extracted from one individual per species, and libraries were constructed and sequenced on an Illumina HiSeq 2500 platform with at least 6-Gb sequencing depth for each species.

For the seven ladybird transcriptomes through the 1KITE project, we used methods of RNA extraction, library construction and transcriptome sequencing that were consistent with Misof et al. (2014). Samples were preserved in RNAlater before total RNA isolation using TRIzol reagent according to the manufacturer’s instructions (Invitrogen, Grand Island, NY, USA). Libraries were then constructed and sequenced on an Illumina HiSeq 2000 platform according to the manufacturer’s protocols, generating about 2.5 Gb raw data per specimen with each library being sequenced for 150 bp paired-end reads.

Transcriptomic Illumina data from different species, whether newly sequenced or obtained from NCBI SRA database, was initially cleaned using Trimmomatic v0.39 (Bolger et al., 2014) to remove adaptors and low-quality sequences with default parameters. The clean data was then *de novo* assembled separately using Trinity v2.8.5 (Grabherr et al., 2011), SOAPdenovo-Trans v1.04 (Xie et al., 2014), Oases v0.2.09 (Schulz et al., 2012), Trans-ABySS v2.0.1 (Robertson et al., 2010) and IDBA-Tran v1.1.3 (Peng et al., 2013), as proposed in EvidentialGene (Gilbert, 2013). To prevent cross contamination between transcriptomes during sequencing, CroCo v1.1 (Simion et al., 2018) was used with default parameters to detect and remove contamination sequences. We created a customized nucleotide sequence database for Coccinelloidea containing all the ladybird genome sequences we used alongside previously published versions (Ando et al., 2018; Chen et al., 2021; Gautier et al., 2018) and the sequences of species under Coccinellidae, Endomychidae, Corylophidae, Latridiidae, Alexiidae, Cerylonidae, Discolomatidae and Bothrideridae in the NCBI NT database. Then, transcriptomic assemblies were searched against both this customized database and the NCBI NT database by BLAST v2.8.1+ (Camacho et al., 2009). Transcripts with percent identity < 90% compared to those within our customized database and percent identity >= 98% compared those in the NCBI NT database were considered foreign and removed from assemblies. Non-redundant coding gene set for each species were constructed by EvidentialGene v2018.06.18 (Gilbert, 2013). Protein from this gene set were annotated with method for those from the genomes as described above.

### 1.1.7 Ortholog group assignment and annotation

We extracted the longest isoform of each gene in the genomes using Generic Feature Format (GFF) prediction files to generate genome protein sets. We then evaluated these protein sets along with those from transcriptomes using BUSCO v5.2.2 pipeline with the Insecta set of OrthoDB v10 database to ensure that complete (C) proteins made up larger than 80% of protein set of each species. Next, we assigned consistent OGs between the genome dataset and extended dataset by analyzing protein sets of all 87 species through OrthoFinder v2.5.4 (Emms and Kelly, 2019). According to the annotations of the Pfam v32 database (El-Gebali et al., 2019) to the protein sets by InterProScan v5.32 (Jones et al., 2014) as described above, KinFin v1.0.3 (Laetsch and Blaxter, 2017b) was used to infer representative Pfam, InterPro and GO annotations for the OGs with default settings.

### 1.1.8 Genome synteny analysis

Whole genome synteny among the chromosomes of CMONT, HVIMA, CSEPT, HAXYR, ABIPU, PJAPO and the outgroups DHELO, TCAST, MBIPU and TDICH was detected. The information of the chromosomes of CSEPT, HAXYR, ABIPU, PJAPO, DHELO and TCAST was obtained from the NCBI Genome database, while those of HVIMA, MBIPU and TDICH were obtained from the downloaded assemblies. Additionally, we also took all the genome scaffolds or contigs with length >= 1 Mb in other four ladybirds without chromosome-level genomes into the analysis, in order to extend the results. The hits between the gene members in the same OGs were extracted from each species-to-species DIAMOND search result generated in the OrthoFinder OG assignment in Section 1.1.7, and subsequently submitted to MCScanX (Wang et al., 2012) to analyze the synteny. Circos v0.69-9 (Krzywinski et al., 2009) was used to plot the links of the syntenic gene pairs among different chromosomes in a species, and chromosomes between two species, respectively.

## 1.2 Results

### 1.2.1 General genomic features

In this study, we used Hi-C technology to assemble a chromosome-level genome of *C. montrouzieri* based on our previous contig-level assembly (Li et al., 2021a). We anchored, ordered and oriented 398 scaffolds to ten autosomes and one X chromosome using Hi-C scaffolding (Figure S1.2), based on the published karyotype of *C. montrouzieri* (2n = 22, 20A + XY) (Blackmon and Demuth, 2015). Over 99.85% of the assembled bases were anchored. This resulted in an improved continuity compared with the contig-level assembly: our new chromosome-level genome assembly has a scaffold N50 of 101.22 Mb (Table S1.2) (but N50 = 9.22 Mb in the contig-level assembly, making a large improvement of continuity compared with the contig-level assembly with a contig N50 of 9.22 Mb (Li et al., 2021a)). BUSCO pipeline (Manni et al., 2021) showed that this chromosome-level genome and its gene set contain 99.2% and 94.7% complete genes, respectively (Table S1.2).

To assemble the genomes of *M. discolor* and *H. vigintioctopunctata*, we generated 60.80 Gb and 41.16 Gb of high-quality clean reads using PromethION DNA sequencing (Oxford Nanopore, UK) and 33.35 Gb and 47.99 Gb using Illumina data for each species respectively. After assembling and polishing the genomes, we identified and removed 64 bacterial sequences comprising approximately 7.36 Mb in the genome of *M. discolor* and 1 bacterial sequence comprising approximately 0.17 Mb in the genome of *H. vigintioctopunctata* combining BlobTools (Laetsch and Blaxter, 2017a) with manual checks to BLAST (Camacho et al., 2009) searches against NT database. The genome assembly of *M. discolor* yielded 2,609 contigs with a total size of 523.75 Mb and a contig N50 of 2.63 Mb, while the genome assembly of *H. vigintioctopunctata* yielded 151 contigs with a total size of 496.12 Mb and a contig N50 of 5.76 M. The results of BUSCO pipeline found that both genome assemblies had an impressively high level of completeness with regard to complete genes at 94.3% and 99.3% (Table S1.2). Similarly, the gene sets of both species had a high level of completeness with regard to complete genes at 92.4% and 97.5%.

To assemble the genome of *C. impunctata*, we used PacBio long reads (18.70 Gb), PacBio CCS reads (10.86 Gb), 10X Genomics linked reads (52.66 Gb) and Illumina reads (41.14 Gb). After removing 9 foreign sequences (1.38 Mb), the final assembly consisted of 5,777 scaffolds with a total size of 796.00 Mb bases and a scaffold N50 of 562.46 Kb. Using the BUSCO pipeline, 98.2% and 96.0% complete genes were found, respectively for the genome and gene set of *C. impunctata* (Table S1.2), revealing a high completeness of this assembly.

### 1.2.2 General features of ortholog groups and datasets

To study the evolution of feeding habits in the ladybirds, we predicted and collected the protein sets of the genomes of ten ladybirds and 15 representative outgroup beetle genomes, which made up the genome dataset, with BUSCO completeness estimates ranging from 90.2% to 99.6% (Additional file 2: Table SE1). On the basis of the genome dataset, we additionally constructed 59 ladybird and three outgroup Coccinelloidea transcriptomic protein sets, with BUSCO completeness estimates ranging from 82.0% to 97.4%, to make an extended dataset.

All these protein sets of totally 87 species (the extended dataset) were analyzed by OrthoFinder (Emms and Kelly, 2019). 1,869,610 of 3,224,726 (57.98%) genes were assigned into 148,089 OGs, including 1,074 OGs with all species present. Using KinFin (Laetsch and Blaxter, 2017b) to infer representative annotations, 14,601 OGs were annotated in Pfam database and 6,810 OGs were annotated in GO database. Among 20,862 OGs from only the genome dataset with at least two genes, 4,423 were present in all protein sets of the genomes, while 5,897 were only present in one or more ladybird genomes, containing 33,333 of 226,906 (14.69%) genes from the ladybird genomes.

### 1.2.3 Genome synteny

Synteny analysis by MCScanX (Wang et al., 2012) was performed among all the genomes of ladybirds, especially the six chromosome-level genomes, including CMONT, HVIMA, CSEPT, ABIPU, HAXYR and PJAPO, and chromosome level genomes of four outgroup beetles, DHELO, TCAST, MBIPU, TDICH (Figure S1.3, S1.4). In the four ladybirds without chromosome-level genomes, 17 contigs of NPUMI, 97 contigs of HVIGI, 89 contigs of MDISC and 132 scaffolds of CIMPU with the length >= 1Mb were also analyzed.

Compared with the genomes of the outgroup beetles and other ladybirds, CMONT has more chromosomes (10A + X) than most species with only ten chromosomes (9A + X), including TDICH, MBIPU, TCAST, PJAPO, ABIPU and CSEPT. In the genome of CMONT, chromosome 8 and 9 are mainly derived from the fission of chromosome 1 of TDICH, chromosome 1 of MBIPU, chromosome 3 of TCAST, the chromosome 1 and 7 of DHELO, chromosome 10 and 11 of HVIMA, chromosome 1 of PJAPO, chromosome 2 of HAXYR, chromosome 1 of ABIPU or chromosome 1 of CSEPT, leading to increase of the number of the chromosomes. We thus consider that it is a species-specific chromosomal fission event, mostly derived from the longest chromosomes in other species.

HVIMA also has eleven chromosomes, with its chromosome 6 identified as the candidate X chromosome (10A + X) (Zhu et al., 2023). But it seems to go through different chromosomal events. Chromosome 10 and 11 of HVIGI are mainly derived from chromosome 1 of TDICH, chromosome 1 of MBIPU, chromosome 3 of TCAST, the chromosome 1 and 7 of DHELO, chromosome 8 and 9 of CMONT, chromosome 1 of PJAPO, chromosome 2 of HAXYR, chromosome 1 of ABIPU or chromosome 1 of CSEPT. Although the source of these two chromosomes is the same as that of chromosome 8 and 9 of CMONT, their distribution in the two chromosomes is different. Additionally, chromosome 1 and 7 of HVIMA are recombined from chromosome 2 and 9 of TDICH, chromosome 3 and 9 of MBIPU, chromosome 6 and 7 of TCAST, chromosome 6, 10 and 13 of DHELO, chromosome 1 and 10 of CMONT, part of chromosome 2, 3, 4 and 5 of PJAPO, part of chromosome 1 and 3 of HAXYR, chromosome 2 and 9 of ABIPU or chromosome 2 and 9 of CSEPT. These two species-specific events have also been shown in its previous research (Zhu et al., 2023).

On the contrary, fewer chromosomes are found in HAXYR (7A + X) (Boyes et al., 2021), which has undergone two fusion events, including chromosome 1 from chromosome 2 and 7 of TCAST, chromosome 1 and 7 of CMONT, chromosome 1, 4 and 7 of HVIMA, chromosome 2, 3 and 4 of PJAPO, chromosome 2 and 7 of ABIPU or chromosome 2 and 8 of CSEPT, and chromosome 3 from chromosome 5 and 10 of CMONT, chromosome 1, 7 and 9 of HVIMA, chromosome 4, 5 and 6 of PJAPO, chromosome 8 and 9 of ABIPU or chromosome 6 and 9 of CSEPT (Figure S1.4). These two fusions have been also confirmed in other version of chromosome-level genome of HAXYR (Chen et al., 2021), which also indicates a species-specific event.

PJAPO, ABIPU and CSEPT all have ten chromosomes (9A + X) (Crowley et al., 2021b; Wellcome Sanger Institute Tree of Life programme et al., 2022; Zhang et al., 2020b). Y chromosome was also assembled in the genome of ABIPU (Wellcome Sanger Institute Tree of Life programme et al., 2022), but no synteny is found compared with other species. For PJAPO, the chromosome 10 reveals a congruent relationship to chromosome X in other genomes, which can be identified as its X chromosome. In addition, the chromosomes of PJAPO seem to have experience relatively complicated recombination events. Chromosome 2 of PJAPO was mainly derived from part of chromosome 1 and 2 of CMONT, chromosome 1 and 5 of HVIMA, chromosome 1 and 4 of HAXYR, chromosome 2 and 3 of ABIPU or chromosome 2 and 4 of CSEPT. Chromosome 3 of PJAPO was mainly derived from part of chromosome 1 and 4 of CMONT, chromosome 1, 2 and 7 of HVIMA, chromosome 1 and 7 of HAXYR, chromosome 2 and 5 of ABIPU or chromosome 2 and 3 of CSEPT. Chromosome 4 of PJAPO was mainly derived from part of chromosome 4, 7 and 10 of CMONT, chromosome 1, 2, 4 and 7 of HVIMA, chromosome 1, 3 and 7 of HAXYR, chromosome 5, 7 and 9 of ABIPU or chromosome 3, 8 and 9 of CSEPT. Chromosome 5 of PJAPO was mainly derived from part of chromosome 3 and 10 of CMONT, chromosome 1 and 8 of HVIMA, chromosome 3 and 5 of HAXYR, chromosome 4 and 9 of ABIPU or chromosome 5 and 9 of CSEPT. Chromosome 7 of PJAPO was mainly derived from part of chromosome 3 of CMONT, chromosome 8 of HVIMA, chromosome 5 of HAXYR, chromosome 4 of ABIPU or chromosome 5 of CSEPT. Chromosome 8 of PJAPO was mainly derived from part of chromosome 2 of CMONT, chromosome 5 of HVIMA, chromosome 4 of HAXYR, chromosome 3 of ABIPU or chromosome 4 of CSEPT.

Additionally, we can find that two chromosomes are frequently recombined among TDICH (chromosome 6 and 8), MBIPU (chromosome 4 and 8), TCAST (chromosome 2 and 10), DHELO (chromosome 3 and 5), and the ladybirds (chromosome 5 and 7 of CMONT, chromosome 4 and 9 of HVIMA, part of chromosome 4 and 6 of PJAPO, part of chromosome 1 and 3 of HAXYR, chromosome 7 and 8 of ABIPU, chromosome 6 and 8 of CSEPT), but they keep relatively stable within the six ladybirds. However, it seems that contig scaff1 (part), scaff4 and scaff11 of NPUMI also reveal recombination to those two chromosomes in all the outgroups and the ladybirds, which indicates that the stabilization of these two chromosomes occurred later than the ancestor of the ladybirds and even Coccinellinae.

Furthermore, a large amount of syntenic gene pairs appear within each chromosome of PJAPO, which shows agreement on the large duplications by BUSCO analysis (Figure S1.3), while relatively few syntenic gene pairs were found within the chromosomes in CMONT or HVIMA. In HAXYR, ABIPU and CSEPT, only some highly similar syntenic blocks repeatedly exist in each chromosome. As described above, frequent and complicated recombination always occurred in PJAPO in the syntenic comparison to other genomes. Indeed, chromosomes of PJAPO are likely to have more translocation events than other beetle genomes (Van Dam et al., 2021), and we found this a species-specific condition of PJAPO compared with other ladybirds or outgroup beetles. Clear chromosomal evolution events in PJAPO are hard to described and explained.

High continuity of the contig-level genomes of NPUMI also shows at least one fusion event of the longest contig scaff1 from chromosome 2, X and part of recombination of 5, 7 of CMONT, chromosome 5, 6 (X) and part of recombination of 4, 9 of HVIMA, chromosome 2, 10 (X) and part of recombination of 4, 6 of PJAPO, chromosome 4, X and part of recombination of 1, 3 of HAXYR, chromosome 3, X and part of recombination of 7, 8 of ABIPU, and chromosome 4, X and part of recombination of 6, 8 of HAXYR. The contig scaff1, scaff3, scaff4 of HVIGI are also derived from two different chromosomes respectively, compared to most of the six chromosome-level genomes of the ladybirds, but the contig scaff3 is completely derived from part of chromosome 1 of HVIMA, which supports the possibility that recombination of chromosome 1 and 7 in HVIMA occurred not later than MRCA of HVIMA and HVIGI. However, these results of the contig-level genomes may be caused by contig misassembly and are still ambiguous.

## 1.3 Summary

We newly sequenced the high-quality genomes of three ladybirds, assembled the chromosome-level genome of one ladybird and the transcriptomes of 23 new ladybirds and two Coccinelloidea species, combined with the previously published six ladybird genomes, 36 ladybird transcriptomes and 16 outgroup beetle genomes or transcriptomes, to construct a core genome dataset and an extended dataset of ladybird OGs for the downstream evolution analysis.

Additionally, we conducted a syntenic analysis among the genomes of the ladybirds and four outgroup beetles. The results indicate the fusion, fission and recombination events in the chromosomes of the ladybirds. However, the chromosomes of the beetles are highly changed, leading to variable chromosome number. Most these events seem to be only species-specific and not related to change of the feeding habits. We also found large amounts of gene duplications in the genome of PJAPO, which leads to large gene copies in the OGs. To avoid the impact on the analysis because of the large gene copies, we excluded PJAPO from the downstream OG contraction and expansion analysis and selection pressure detection of single-copy genes.

Table S1.1 Transcriptomes for gene prediction of genomes of ten ladybirds and *Dastarcus helophoroides*

| Species | Treatment | RNA-seq SRA accession |
| --- | --- | --- |
| *Cryptolaemus montrouzieri* | Female adult feeds on mealybugs | SRR2971116 |
| *Cryptolaemus montrouzieri* | 4th instar larva feeds on mealybugs | SRR2971112 |
| *Cryptolaemus montrouzieri* | Female adult feeds on aphids | SRR6981477 |
| *Cryptolaemus montrouzieri* | 4th instar larva feeds on pollen | SRR8325176 |
| *Cryptolaemus montrouzieri* | 4th instar larva feeds on pork meat | SRR8325159 |
| *Micraspis discolor* | 4th instar larva feeds on moth eggs | SRR16629587 |
| *Micraspis discolor* | 4th instar larva feeds on pollen | SRR16629580 |
| *Micraspis discolor* | 4th instar larva feeds on aphids | SRR16629579 |
| *Micraspis discolor* | 4th instar larva feeds on mealybugs | SRR16629584 |
| *Micraspis discolor* | Wild caught adult | SRR11576236 |
| *Henosepilachna vigintioctopunctata* | Wild caught adult | SRR11576235 |
| *Henosepilachna vigintioctopunctata* | Eggs | SRR6221652 |
| *Henosepilachna vigintioctopunctata* | 1-4th instar larvae | SRR6221653 |
| *Henosepilachna vigintioctopunctata* | Pupae | SRR6221654 |
| *Henosepilachna vigintioctopunctata* | Adult injected with *Bacillus subtilis* | Our unpublished data |
| *Cynegetis impunctata* | Wild caught 1-4th instar larvae | SRR24194748 |
| *Cynegetis impunctata* | Gut of adult | SRR24194747 |
| *Cynegetis impunctata* | Adult without gut | SRR24194745 |
| *Cynegetis impunctata* | Gut of adult | SRR24194746 |
| *Cynegetis impunctata* | Adult without gut | SRR24194744 |
| *Cynegetis impunctata* | Adult | SRR24194743 |
| *Harmonia axyridis* | 3rd instar larva | SRR8380388 |
| *Harmonia axyridis* | 1st instar larva | SRR8380389 |
| *Harmonia axyridis* | Pupa of population with red wings | DRR092254 |
| *Harmonia axyridis* | Male adult of 4th day after emergence | SRR5891405 |
| *Harmonia axyridis* | Female adult of 5th day after spawning | SRR5451334 |
| *Coccinella septempunctata* | Male adult feeds on artificial diet | SRR9208146 |
| *Coccinella septempunctata* | Female adult feeds on aphids | SRR9208152 |
| *Coccinella septempunctata* | Adult injected with bacteria | ERR1145729 |
| *Coccinella septempunctata* | Adult of diapause for 30 days | SRR2971094 |
| *Coccinella septempunctata* | 4th instar larva feeds on mealybugs | SRR9649803 |
| *Novius pumilus* | Wild caught adult | SRR11576223 |
| *Novius pumilus* | 4th instar larva feeds on *Icerya aegyptiaca* | SRR15420509 |
| *Novius pumilus* | 4th instar larva of starvation | SRR15420493 |
| *Novius pumilus* | Female adult feeds on *I. aegyptiaca* | SRR15420505 |
| *Novius pumilus* | Female adult of starvation | SRR15420504 |
| *Propylea japonica* | all stages of susceptible strain | SRR1299012 |
| *Propylea japonica* | 4th instar larva feeds on aphids | SRR9649793 |
| *Propylea japonica* | Female adult feeds on aphids | SRR24183502 |
| *Propylea japonica* | Female adult feeds on mealybugs | SRR24183500 |
| *Propylea japonica* | Adult injected with *B. subtilis* | Our unpublished data |
| *Adalia bipunctata* | Wild caught adult | SRR11576219 |
| *Adalia bipunctata* | Sham-injected adult | ERR1145718 |
| *Adalia bipunctata* | Sham-injected adult | ERR1145719 |
| *Adalia bipunctata* | Sham-injected adult | ERR1145720 |
| *Adalia bipunctata* | Bacteria-injected adult | ERR1145721 |
| *Adalia bipunctata* | Bacteria-injected adult | ERR1145722 |
| *Adalia bipunctata* | Bacteria-injected adult | ERR1145723 |
| *Henosepilachna vigintioctomaculata* | Adult | SRR18847866 |
| *Dastarcus helophoroides* | newly emerged adults with both sexes | SRR1201401 |
| *Dastarcus helophoroides* | Wild caught adult | SRR13089473 |
| *Dastarcus helophoroides* | antennae of adults with both sexes | SRR1044007 |
| *Dastarcus helophoroides* | newly hatched larvae | SRR10201368 |
| *Dastarcus helophoroides* | newly hatched larvae | SRR10201370 |

Table S1.2 Genomic features of ten ladybird genomes

| Features | *Cryptolaemus montrouzieri* | *Micraspis discolor* | *Henosepilachna vigintioctopunctata* | *Cynegetis impunctata* | *Novius pumilus* * |
| --- | --- | --- | --- | --- | --- |
| Reference | this study | this study | this study | this study | Tang et al. (2022b) |
| Level | chromosome,  10A + X | contig | contig | scaffold | contig |
| Genome size  (Mb) | 988.13 | 523.75 | 496.12 | 796.00 | 182.42 |
| Numbers of  scaffolds/contigs | 206 scaffolds | 2,609 contigs | 151 contigs | 5,777 scaffolds | 942 contigs |
| N50 (Mb) | 101.22 | 2.63 | 5.76 | 562.46 Kb | 7.58 |
| BUSCO estimates  n=1367 | C: 99.2%  [S:98.0%,D:1.2%]  F: 0.1%  M: 0.7% | C: 94.3%  [S:90.4%,D:3.9%]  F: 0.3%  M: 5.4% | C: 99.3%  [S:96.9%,D:2.4%]  F: 0.1%  M: 0.6% | C: 98.2%  [S:96.3%,D:1.9%]  F: 0.5%  M: 1.3% | C: 97.8%  [S:95.5%,D:2.3%]  F: 0.5%  M: 1.7% |
| G + C (%) | 34.88 | 35.04 | 31.61 | 31.56 | 37.12 |
| Number of genes | 24,180 | 20,056 | 21,898 | 32,621 | 15,772 |
| Repeat (%) | 68.25 | 64.86 | 48.92 | 61.48 | 44.58 |
| Numbers of Ns | 20,400 | 0 | 0 | 0 | 0 |

Table S1.2 (continue)

| Features | *Harmonia*  *axyridis* * | *Coccinella septempunctata* * | *Adalia*  *bipunctata* * | *Propylea*  *japonica* * | *Henosepilachna vigintioctomaculata* * |
| --- | --- | --- | --- | --- | --- |
| Reference | Boyes et al. (2021) | Crowley et al. (2021b) | Wellcome Sanger Institute Tree of Life programme et al. (2022) | Zhang et al. (2020b) | Zhu et al. (2023) |
| Level | chromosome,  7A + X | chromosome,  9A + X | chromosome,  9A + XY | chromosome,  9A + X | Chromosome,  10A + X |
| Genome size  (Mb) | 425.54 | 398.87 | 475.29 | 851.23 | 581.63 |
| Numbers of  scaffolds/contigs | 14 scaffolds | 25 scaffolds | 119 scaffolds | 1074 scaffolds | 52 scaffolds |
| N50 (Mb) | 63.68 | 41.44 | 45.87 | 100.34 | 56.17 |
| BUSCO estimates  n=1367 | C: 99.0%  [S:96.0%,D:3.0%]  F: 0.1%  M: 0.9% | C: 99.2%  [S:98.0%,D:1.2%]  F: 0.1%  M: 0.7% | C:98.8%  [S:97.4%,D:1.4%]  F:0.3%  M:0.9% | C: 95.8%  [S:66.5%,D:29.3%]  F: 0.4%  M: 3.8% | C:99.1%  [S:94.6%,D:4.5%]  F:0.1%  M:0.8% |
| G + C (%) | 35.14 | 36.42 | 35.90 | 35.12 | 31.83 |
| Number of genes | 18,877 | 18,364 | 19,636 | 39,730 | 24,404 |
| Repeat (%) | 56.58 | 55.52 | 62.84 | 59.18 | 52.56 |
| Numbers of Ns | 37,100 | 23,593 | 21,619 | 370,201 | 45,900 |

Abbreviations in BUSCO: C: complete, S: single, D: duplicate, F: fragment, M: missing

* The previously published genomes were additionally refined through our pipeline and all the parameters were newly estimated in this study. It should be noted that, though the parameters of the genome of *H. axyridis* and *C. septempunctata* in this table were obtained through annotation by our pipeline, we selected the NCBI RefSeq annotations of these two species for the downstream analyses.


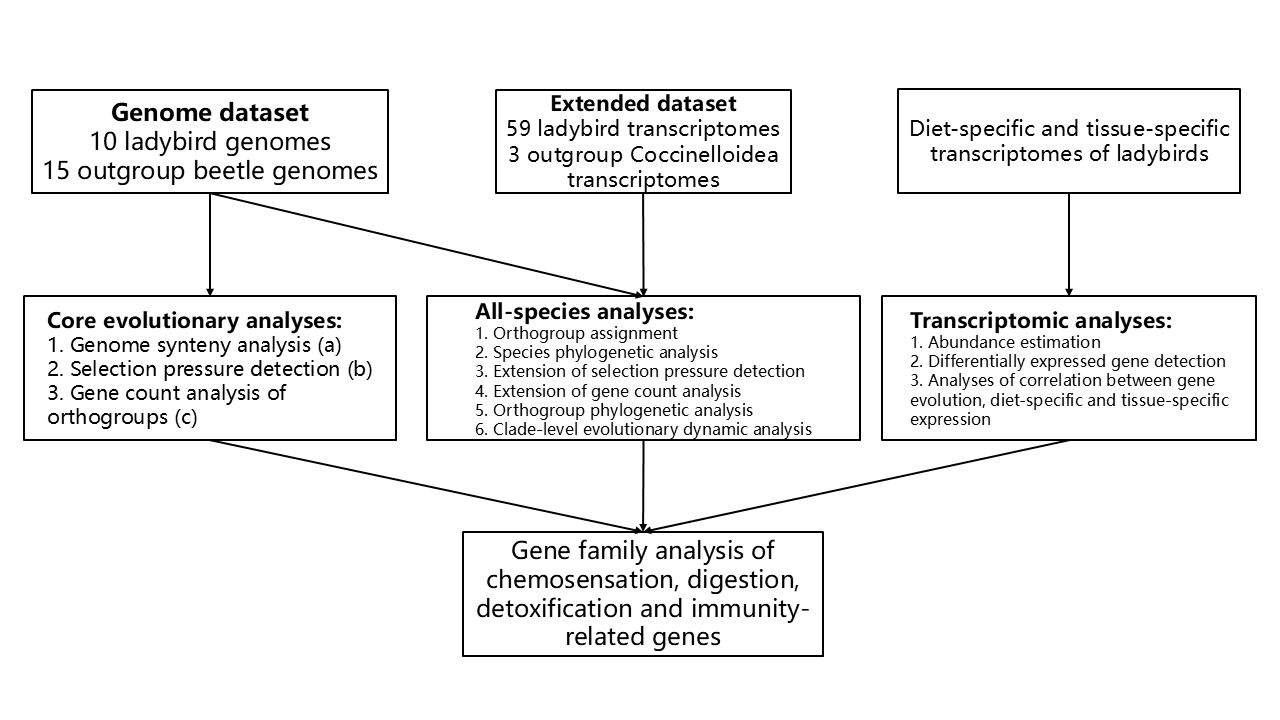


Figure S1.1 Design of the datasets for different analyses. a: genomes of 10 ladybirds and chromosome-level genomes of 4 outgroup beetles were involved; b: PJAPO was excluded from detection of single-copy ortholog groups (OGs) due to large amount of gene duplications; c: PJAPO was excluded from CAFE analysis of expansions and contractions due to large amount of gene duplications. A high-quality figure can be downloaded from https://github.com/huangyh45/ladybird-genomes-supplementary-figures.


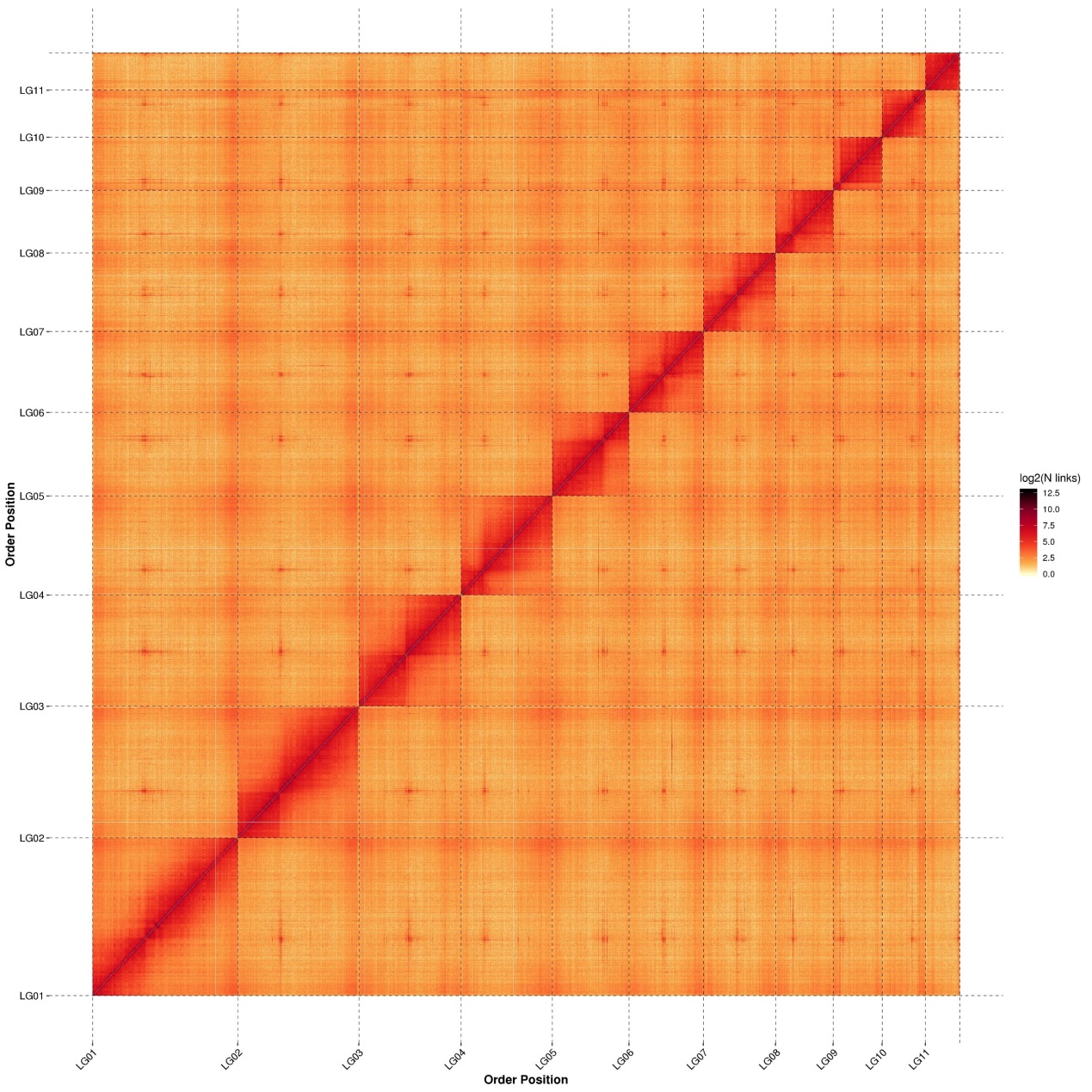


Figure S1.2 Hi-C contact heatmap of the chromosome-level genome of *C. montrouzieri*. A high-quality figure can be downloaded from https://github.com/huangyh45/ladybird-genomes-supplementary-figures.


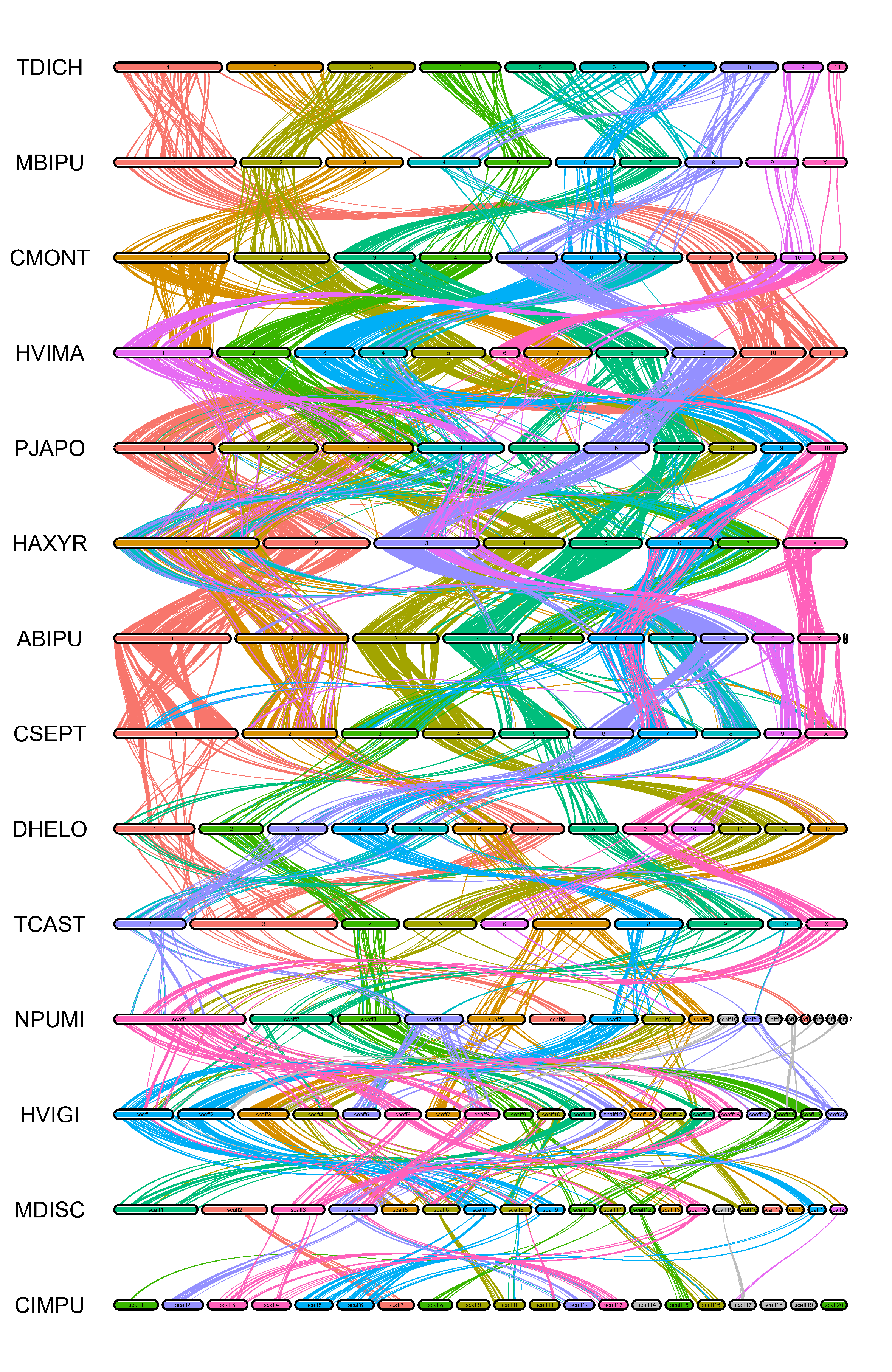


Figure S1.3 Whole-genome synteny among multiple species of ladybirds or outgroup beetles. The color of the chromosomes, scaffold or contigs and the links are mainly based on the homology with first species TDICH and then the last species above. For the four species on the bottom with contig-level (NPUMI, HVIGI, MDISC) or scaffold-level (CIMPU) genomes, only the longest 20 sequences >= 1 Mb are visualized. A high-quality figure can be downloaded from https://github.com/huangyh45/ladybird-genomes-supplementary-figures.


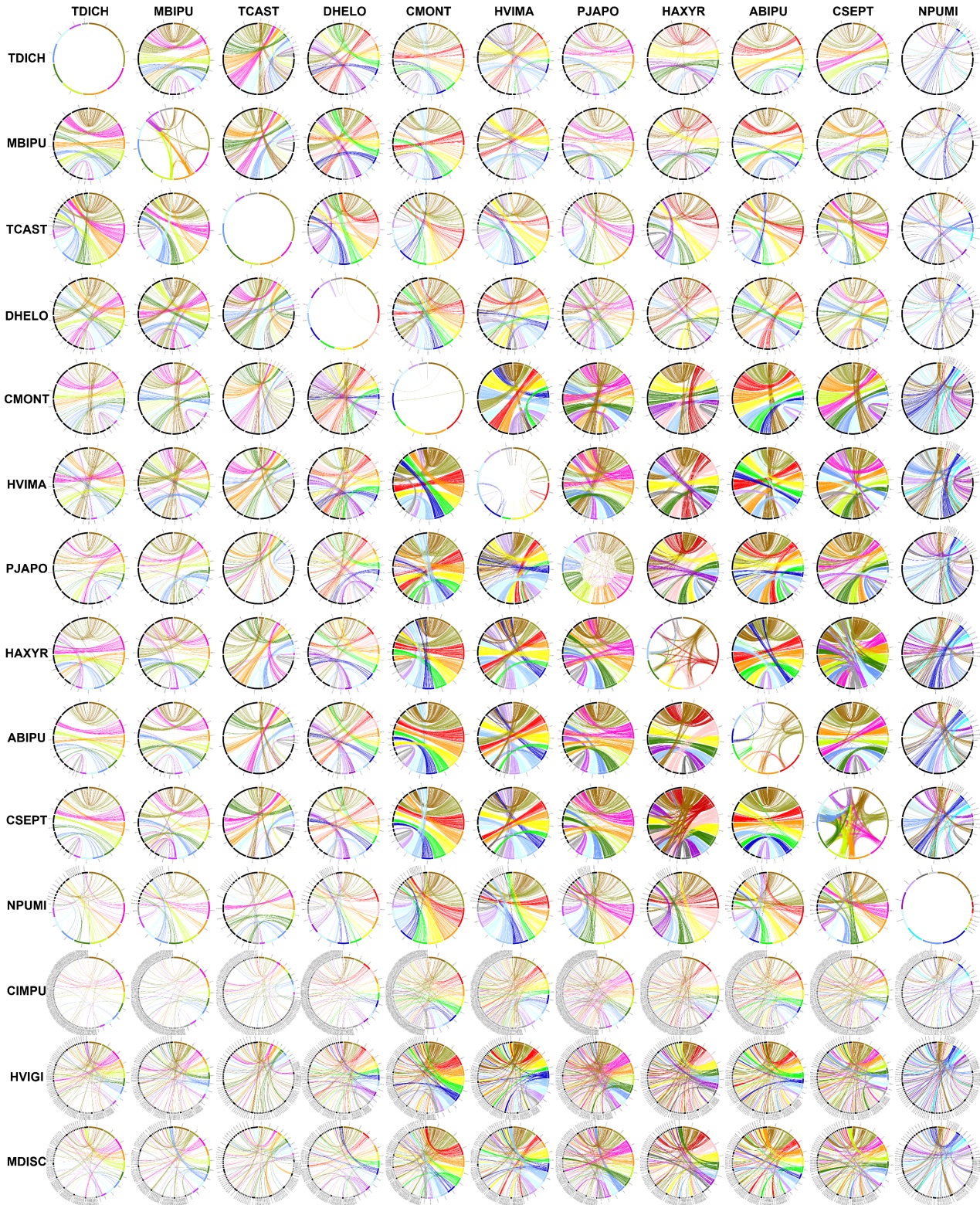


Figure S1.4 Whole-genome synteny between each pair of two species of ladybirds or outgroup beetles. Genomes of NPUMI, CIMPU, HVIGI and MDISC are scaffold-level or contig-level, in which only sequences >= 1 Mb are analyzed. The sequences of CIMPU, HVIGI and MDISC are too fragmented and their genome synteny results are only visualized by coloring another genome. A high-quality figure can be downloaded from https://github.com/huangyh45/ladybird-genomes-supplementary-figures.

# 2 Species phylogeny

## 2.1 Materials and methods

### 2.1.1 Phylogeny inference

308 OGs with minimum of 74 (85.06%) species having single-copy genes in the extended dataset were selected by OrthoFinder for species tree. Combining these OGs with those OGs with single-copy genes among all the 24 beetle genomes except PJAPO (due to large amount of gene duplications displayed in Additional file 2: Table SE1 and detailed in Section 1.2.3), which are likely to be indeed single-copy genes among the beetles, we used single-copy genes from totally 770 OGs, to concatenate into a protein sequence supermatrix and infer the species phylogeny. Sequences of these 770 OGs containing single-copy genes were aligned using L-INS-i mode of MAFFT v7.480 (Katoh and Standley, 2013) and the alignments were trimmed by trimAl v1.4 (Capella-Gutierrez et al., 2009) with the “automated1” option, separately, which aimed to remove the poorly aligned regions. Then the concatenated supermatrix of 770 alignments was imported into the “MFP+MERGE” mode of IQ-TREE v2.1.4-beta (Minh et al., 2020) to search for the best partition scheme and the best models and reconstruct the phylogeny of the 87 species with 1,000 ultrafast bootstrap (UFBoot) replicates. Finally, the species tree was rooted with *Nebria ingens* (NINGE) belonging to Adephaga according to the tree of Zhang et al. (2018), using the Python ETE 3 Toolkit (Huerta-Cepas et al., 2016). Additionally, those 770 trimmed alignments of the single-copy genes were also used to build the trees by IQ-TREE respectively. Then all the 770 gene trees were input to ASTRAL-Pro v1.1.6 (Zhang et al., 2020a) to generate a species tree under the coalescent model, which was subsequently rooted using the same method as described above.

### 2.1.2 Divergence time estimation

Divergence time estimation was conducted by using MCMCTREE in PAML v4.8a (Yang, 2007) based on the maximum likelihood species tree built by IQ-TREE. The uncorrelated rate model (clock=2) was used, and eleven fossils were chosen to calibrate the clock (Table S2.1). We constrained the maximum bound for the root (crown Coleoptera) to 323.2 million years ago (Ma), according to the maximum age of the Coleoptera-Neuropterida split set by Zhang et al. (2018), because no holometabolous insect was found before the Pennsylvanian, which began at this time.

Referring to the oldest beetle fossil *Coleopsis archaica* (Kirejtshuk et al., 2014) and the time tree estimated by Zhang et al. (2018), 295 Ma was set as the root age (crown Coleoptera) to calculate the prior on the overall substitution rate with the CODEML program in the PAML package under the JTT + F + GAMMA model with four rate categories. The ML estimates of the branch lengths were also calculated by CODEML using 66 partitions from the protein supermatrix divided by IQ-TREE. The gamma-Dirichlet prior for overall rate for genes (rgene gamma) was set at G (1, 4.59), and the gamma-Dirichlet prior for sigma^2 (sigma2 gamma) was set at G (1, 4.5). Then, the MCMCTREE program in PAML estimated the divergence times by using a Monte Carlo algorithm. Burn-in was set as 50,000, meaning the first 50,000 iterations were discarded. Next, the samples were recorded every 50 iterations until 10,000 samples were obtained. Another run was started from different seeds following the same steps. Finally, to check the convergence of the result, we used Tracer v1.7.2 (Rambaut et al., 2018) to ensure that all parameters were similar between two runs and that their effective sample sizes (ESSs) were all larger than 200.

### 2.1.3 Ancestral character reconstruction

We reconstructed the ancestral character state of feeding habits at the internal nodes of the ladybirds in the species time tree, using the part of 73 Coccinelloidea species. Information of diet was obtained from several descriptive books (Hodek, 1973; Hodek et al., 2012; Lundgren, 2009; Ren et al., 2009; Ślipiński, 2007; Ślipiński et al., 2020) and some articles concentrating on feeding habits (Biddinger et al., 2009; Escalona and Ślipiński, 2012; Escalona et al., 2017; Giorgi et al., 2009; Hodek and Honěk, 2009; Magro et al., 2010; Nattier et al., 2021; Seago et al., 2011; Song et al., 2020; Sutherland and Parrella, 2009). For those ladybirds with unknown diets, we also tried to infer the information based on observation of surrounding environments (Additional file 2: Table SE1), but these species were not involved in the ancestral character reconstruction and pruned from the species tree, remaining 61 species available. Some species have polymorphic diets in our data, such as aphids + pollen, aphids + whiteflies and aphids + coccids. Therefore, we designed five index matrices of the models to fit the data. In our models, those polymorphic states could only be obtained from diet expansion with the single states, for example, aphids + coccids could only be obtained from ‘aphids’ or ‘coccids’. The five models included: equal rates for all permitted transitions (ER), two different rates between diet shift of single states and diet expansion (DR), three different rates between diet shift of single states, acquisition and loss of polymorphism in diet expansion (TR), symmetric backward & forward rates for all permitted transitions (SYM) and all-rates-different for permitted transitions (ARD). We first use fitMk function in the R package phytools (Revell, 2012) to fit these five models. Then the customized anova function of fitMk was used to compare the five models. The model with lowest Akaike information criterion (AIC) value (DR model, detail in Figure S2.5) was input to make.simmap function in phytools, in order to perform stochastic mapping on the species tree. 100 simulations were conducted and summarized to obtained the state probabilities (SPs) of each state at the internal nodes.

## 2.2 Results

### 2.2.1 Phylogenetic tree of 69 ladybird species and 18 outgroup species

To reconstruct the phylogeny of 87 species of the extended data, 770 OGs containing available single-copy genes were concatenated into a protein sequence supermatrix with the length of 295,332 amino acids. IQ-TREE (Minh et al., 2020) divided the supermatrix into 66 partitions and infer a best tree of the 69 ladybird (Coccinellidae) species and 18 outgroup species (Figure S2.1). 73 of 84 internal nodes of the species tree have a UFBoot support value of 100, while other one node has a UFBoot support value larger than 95. Additionally, the relationships between the outgroup species conform to the former researches of coleopteran phylogeny except the debatable position of Cleroidea (represented by *Malachius bipustulatus* (MBIPU)) (Li et al., 2021b; McKenna et al., 2019; Zhang et al., 2018). Both these reveal relatively reliable topology of the species tree. Similarly, ASTRAL-Pro constructed a coalescent species tree based on gene trees of these 770 OGs, with 76/85 internal nodes having a local posterior probability (LPP) larger than 0.95 and the same topology of the outgroups (Figure S2.2).

The monophyly of Coccinelloidea, Coccinellidae, Coccinellinae, Microweiseinae, Serangiini, Coccinellini, Epilachnini, Stethorini, Scymnini and Chilocorini was confirmed and highly supported (UFBoot = 100% and LPP = 1) in our data, as same as the results of most phylogenetic researches of the ladybirds (Che et al., 2021; Escalona et al., 2017; Giorgi et al., 2009; Nattier et al., 2021; Robertson et al., 2015; Seago et al., 2011; Szawaryn et al., 2015; Tomaszewska et al., 2021). The monophyly of ABDHP and CSPS clade reported in Che et al. (2021) were also highly supported in our tree (UFBoot = 100% and LPP = 1), though not all the tribes in these two clades were included in our data. Additionally, the three species of Coccidulini were clustered into a monophyletic clade in both concatenated and coalescent species tree, but with relatively low support values (UFBoot = 56% and LPP = 0.75) and completely different topology within the clade. However, different from the monophyletic clade in our analysis, Scymnini and Coccidulini were sometimes found to be paraphyletic (Che et al., 2021), possibly due to limited species in our study.

Within Coccinellinae, *N. pumilus* (NPUMI) is the sister clade of other species. This first basal clade Noviini (represented by NPUMI) within Coccinellinae in our result, were also placed in the basal position of Coccinellinae in some researches (Che et al., 2021; Robertson et al., 2015). Coccinellini is the sister clade of CSPS clade (Che et al., 2021) containing Chilocorini and Sticholotidini. This topology, which is contrasted with the main opinion that Chilocorini is the sister group of Coccinellini, always with species in Sticholotidini placed in a close clade (Escalona et al., 2017; Nattier et al., 2021; Robertson et al., 2015). But the contradiction occurred in other phylogenetic researches with different positions (Che et al., 2021; Giorgi et al., 2009; Seago et al., 2011; Tomaszewska et al., 2021).

Among the species in Coccinellini, the current phylogenetic researches of Coccinellini identified four clades within the tribe (Nattier et al., 2021; Tomaszewska et al., 2021), which can also be found in our tree based on their genera. Our result supports the topology of (((A,B),C),D) reported by Nattier et al. (2021) but not (A,(B,(C,D))) reported by Tomaszewska et al. (2021). The four fungivorous ladybirds (Group A, former Psylloborini) and the four omnivorous ladybirds (Group B) are confirmed as monophyletic clade (UFBoot = 100% and LPP = 1), separately (Figure S2.1, S2.2).

However, the two species tree from different methods are partly different, mainly including the position of *M. bipustulatus* (MBIPU), Stethorini, Scymnini, *Diomus ementitor* (Tran_DEMEN) and the fungivorous ladybirds (Figure S2.3). In addition, although the cluster of ABDHP clade, two Azyini species, Coccidulini, *Chaetolotis amy* (Tran_CHAMY) and Epilachnini is highly supported in both species trees (UFBoot = 100% and LPP = 0.96), the relationship and position among these members are still unclear because of contradiction between the two trees. But these differences do not have impact on the relative position of the ladybird species with genomes and most outgroup species (except MBIPU) and the main phylogeny of the genome dataset, we thus intuitively used the maximum likelihood species tree built by IQ-TREE for the downstream analyses according to easier explainability of the branch lengths.

### 2.2.2 Divergence time of the ladybirds

Through MCMCTREE (Yang, 2007) divergence time estimation with eleven fossil calibrations (Table S2.1), the result suggests that Coccinellidae originated in the Early Cretaceous at 141.71 Ma (95% credibility interval (CI): 130.59-156.07 Ma) (Figure S2.4). It is similar with most estimated times ranging from 118-143 Ma, mostly in the Early Cretaceous (Che et al., 2021; McKenna et al., 2019; Mckenna et al., 2015; Nattier et al., 2021; Tomaszewska et al., 2021; Zhang et al., 2018), except 168.46 Ma estimated by Li et al. (2020) and 196 Ma estimated by Toussaint et al. (2017).

The origin of Coccinellinae occurred in the Early Cretaceous at 109.82 Ma (95% CI: 100.95-120.93 Ma), while that of Microweiseinae took place in the Late Cretaceous at 92.70 Ma (95% CI: 84.84-102.35) in our data. The divergence time of Coccinellinae, 109.82 Ma, is a little earlier than the reported ~100 Ma (Che et al., 2021; McKenna et al., 2019; Mckenna et al., 2015). After that, a rapid radiation within Coccinellinae followed at about 70-120 Ma, parallel with the angiosperm explosion at the same time (Bell et al., 2010; Foster et al., 2017), which supports the current conclusions (Che et al., 2021; Li et al., 2021b). In the meantime, the main prey of several clades in Coccinellinae, the aphids (90-106 Ma) (Johnson et al., 2018; Vea and Grimaldi, 2016), and the guard of prey of Coccinellinae, the ants (~120 Ma) (Branstetter et al., 2017), originated and diversified, which reveals possible co-evolution driving the change of the feeding habits.

The crown ages of Serangiini, Coccinellini, Epilachnini, Stethorini, Chilocorini, Scymnini and Coccidulini are 59.00 Ma (95%CI: 53.71-65.54 Ma), 54.22 Ma (95% CI: 49.78-59.68 Ma), 48.17 Ma (95% CI: 44.03-53.22 Ma), 49.86 Ma (95% CI: 45.33-55.31 Ma), 58.53 Ma (95% CI: 53.59-64.75 Ma), 81.12 Ma (95% CI: 74.43-89.35 Ma) and 79.83 Ma (95% CI: 73.32-88.01 Ma) respectively, in which those of Serangiini, Coccinellini, Epilachnini, Stethorini, Chilocorini are in the Paleogene. However, our crown age of Coccinellini (54.22 Ma) is later than reported time of 68-84 Ma in the Late Cretaceous (Che et al., 2021; Li et al., 2020; Nattier et al., 2021; Tomaszewska et al., 2021). According to our time, Coccinellini, in which the most species is mainly aphidophagous, originated later than Aphidoidea that was estimated to took place in the Late Cretaceous (Johnson et al., 2018; Vea and Grimaldi, 2016). A delay can be also observed comparing our time of mite feeding Stethorini at 48.86 Ma with the reported ~65 Ma, while the time of plant feeding Epilachnini at 48.17 Ma is similar with the reported time of ~50 Ma (Che et al., 2021).

The ancestor of four fungivorous ladybirds took place at 19.20 Ma (95% CI: 17.36-21.37 Ma), while the divergence among the four omnivorous ladybirds occurs at 31.49 Ma (95%CI: 28.83-34.71 Ma). But the limited species may cause a later time, compare with the time at ~38 Ma of origin of the fungivorous ladybirds with abundant species (Nattier et al., 2021; Tomaszewska et al., 2021).

### 2.2.3 Ancestral character reconstruction of the ladybird diets

According to the stochastic character mapping by phytools package (Figure S2.5) (Revell, 2012), the diet of Coccinellidae ancestors tends to be coccidophagy (state probability (SP) = 81%), which is obtained from fungivory of their Coccinelloidea ancestors (Figure S2.6). This result is same to the conclusion of previous researches (Escalona et al., 2017; Giorgi et al., 2009; Leschen, 2000; Seago et al., 2011). Furthermore, the ancestral states of Coccinellinae and Microweiseinae are also likely to be coccidophagy, with the SPs of 100% and 73%, respectively. And the trunk of our ladybird species tree is estimated as coccid feeding, shifting to other diets sporadically, which is similar to most researches with ancestral character reconstruction in ladybirds (Escalona et al., 2017; Giorgi et al., 2009; Magro et al., 2010). Actually, about 36% of the ladybirds prefer coccids and only 20% of ladybirds mainly prey on aphids (Hodek and Honěk, 2009), and these results thus seem quite reasonable. Although the ancestors of the two subfamilies in our study are both estimated to be coccidophagous, it is reported that, compared with Microweiseinae, coccidophagous species in Coccinellinae prefer those prey with ant guard and thus obtain some ant-specific larval defense mechanisms through the drive of co-evolution, such as waxy exudates and dorsal defensive glands (Seago et al., 2011).

Most primary diet shifts occur at the ancestors of some tribes based on our data, including whitefly feeding of Serangiini (SP = 96%), mite feeding of Stethorini (SP = 99%), herbivory of Epilachnini (SP = 99%) and aphidophagy of Coccinellini (SP = 100%). Specially, some species seems to obtain the preference of aphids from coccidophagy, including *Platynaspis maculosa* (Tran_PMACU), *P. hainanensis* (Tran_PHAIN), *Cryptogonus orbiculus* (Tran_CORBI), *C. hainanensis* (Tran_CHAIN) and *Apolinus lividigaster* (Tran_ALIVI). Che et al. (2021) reported the monophyletic ABDHP clade containing the genera of Tran_PMACU, Tran_PHAIN, Tran_DEMEN, Tran_CORBI and Tran_CHAIN and thought it as an aphidophagous clade. However, the node of this shift in our study is unclear, because the ancestor of ABDHP clade are probably coccidophagous, aphidophagous or both (SP: aphids + coccids = 51%, coccids = 42%, aphids = 7%) with the reason that information of several key species is missed, which is similar to the result of Giorgi et al. (2009). To solve this problem, more species and more information of this group are needed to considered in the future.

Within Coccinellini, two clear diet shifts or expansions are found. Preference on fungi, exactly, powdery mildew, is shifted from aphidophagy at the ancestor of Group A (Figure S2.6) (former Psylloborini, SP = 100%). This shift is supported in all previous researches (Escalona et al., 2017; Giorgi et al., 2009; Magro et al., 2010; Nattier et al., 2021; Song et al., 2020). Additionally, omnivory (aphidophagy and pollinivory) is successfully reconstructed at the ancestor Group B (SP = 94%) (Figure S2.6). This diet expansion is also found in the research by Giorgi et al. (2009), but results from other researches (Escalona et al., 2017; Magro et al., 2010; Nattier et al., 2021; Song et al., 2020) do not support it, which seem to be due to lack of species or diet information in this group. The genera of these four species, *M. discolor* (MDISC), *M. frenata* (Tran_MFREN), *C. quatuordecimpustulata* (Tran_CQUAT) and *Coleomegilla maculata* (Tran_CMACU), along with *Bulaea*, and *Tytthaspis*, are known as pollen feeders and form a group (Escalona et al., 2017; Huang et al., 2022; Nattier et al., 2021; Tomaszewska et al., 2021), which also supports our result.

## 2.3 Summary

We reconstructed a highly supported phylogeny of the ladybirds using the genomic and transcriptomic data, which covered two subfamilies and sixteen tribes in Coccinellidae. Combined with the ancestral character reconstruction, we confirmed several monophyletic clades with diet shift or expansion, including Coccinellidae from fungivory to coccidophagy, Coccinellinae from prey without ant guard to ant-guarded prey, Coccinellini from coccidophagy to aphidophagy, Epilachnini from carnivory to herbivory, Stethorini from coccid feeding to mite feeding, Serangiini from coccidophagy to whitefly feeding, and the nodes from accidophagy to fungivory or omnivory within Coccinellini.

Furthermore, the divergence time estimation based on the omic data may locate the time of evolution of feeding habits, and may provide us an aspect to explain the reasons leading to those evolution, for example, the possible relation of the angiosperm explosion, diversification of prey and enemies, and evolution of the ladybirds. Unfortunately, most fossils of the ladybirds were found from Oise (~48.6-56 Ma) and Baltic (~33.9-47.8 Ma) amber deposits and no fossils have been found with the time before these periods (Kirejtshuk and Nel, 2012; Szawaryn, 2019; Szawaryn, 2021; Szawaryn and Szwedo, 2018; Szawaryn and Tomaszewska, 2020a; Szawaryn and Tomaszewska, 2020b), leading to scarce evidence for further exploration to the time of evolution in the ladybirds.

Overall, our phylo-omic analysis brings a new insight into the Coccinellidae phylogeny and evolution, especially the trace of diet shifts. And the species tree and the divergence time can be used for the downstream evolutionary analyses.

**Table S2.1** Information of fossils used for calibration points in the species phylogenetic analysis. MRCA: most recent common ancestor.

| No. | Calibrated node | Note | Fossil taxa | Min. age | Reference |
| --- | --- | --- | --- | --- | --- |
| 1 | MRCA of *Harmonia* + *Hippodamia* | *Hippodamia olbia* | Coccinellidae - Coccinellini | 11.63 | Zhang et al. (1994) |
| 2 | MRCA of Sticholotidini + Chilocorini | *Electrolotis hoffeinsorum* | Coccinellidae - Sticholotidini | 33.9 | Szawaryn and Tomaszewska (2020a) |
| 3 | Crown Serangiini | *Serangium kalandyki* | Coccinellidae - Serangiini | 33.9 | Szawaryn (2019) |
| 4 | Crown Coccinellinae | *Nephus subcircularis* | Coccinellidae - Scymnini | 48.6 | Kirejtshuk and Nel (2012) |
| 5 | MRCA of Coccinellidae + Corylophidae + Endomychidae | *Burmalestes albertalleni* | Coccinelloidea - Endomychidae | 93.5 | Tomaszewska et al. (2018) |
| 6 | Crown Coccinelloidea | *Archelatrius marinae* | Coccinelloidea - Latridiidae | 125.5 | Kirejtshuk and Azar (2009) |
| 7 | Crown Chrysomeloidea | *Cretoprionus liutiaogouensis* | Chrysomeloidea - Cerambycidae | 122.5 | Wang et al. (2014) |
| 8 | Crown Curculionidae | *Cylindrobrotus pectinatus* | Curculionidae - Scolytinae | 122.5 | Kirejtshuk et al. (2009) |
| 9 | MRCA of Curculionoidea + Chrysomeloidea | *Nanophydes ovatus* | Curculionoidea - Brentidae | 155.7 | Arnoldi (1977) |
| 10 | MRCA of  Staphylinoidea +  Scarabaeoidea | *Juraesalus atavus* | Scarabaeoidea - Lucanidae | 155.7 | Nikolajev et al. (2011) |
| 11 | MRCA of Buprestoidea + Elateroidea | *Ancestrimorpha volgensis* | Buprestoidea - Buprestidae | 164.7 | Alekseev (1993) |


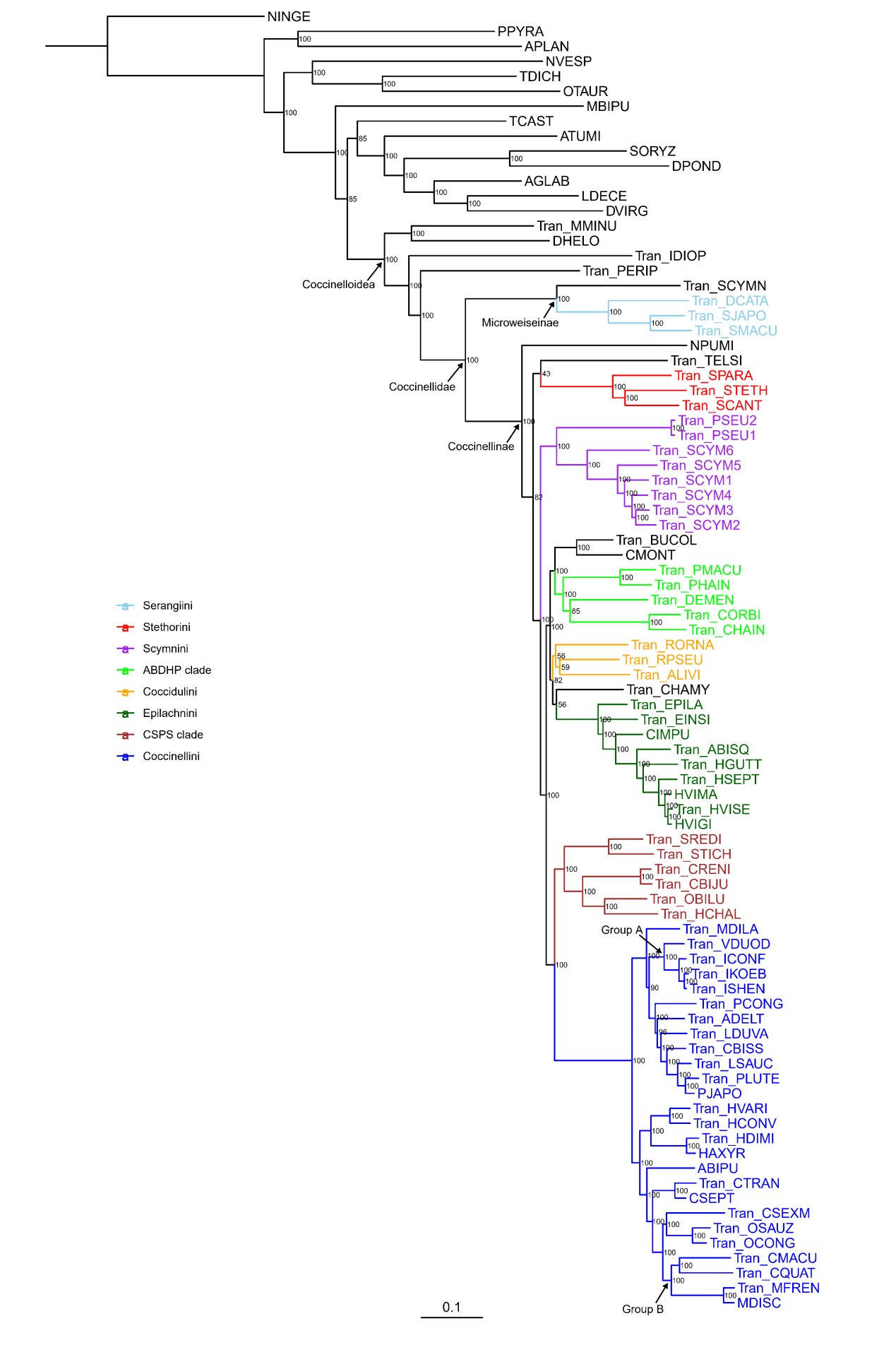


Figure S2.1 Phylogenetic tree of 69 ladybird (Coccinellidae) species and 18 outgroup species inferred by IQ-TREE. Numbers beside nodes are IQ-TREE ultrafast bootstrap (UFBoot) values. The colored clades represent the six main tribes and two clades with a monophyletic origin containing at least three species in our data. Group A: the fungivorous ladybirds (former Psylloborini), Group B: the omnivorous ladybirds. A high-quality figure can be downloaded from https://github.com/huangyh45/ladybird-genomes-supplementary-figures.


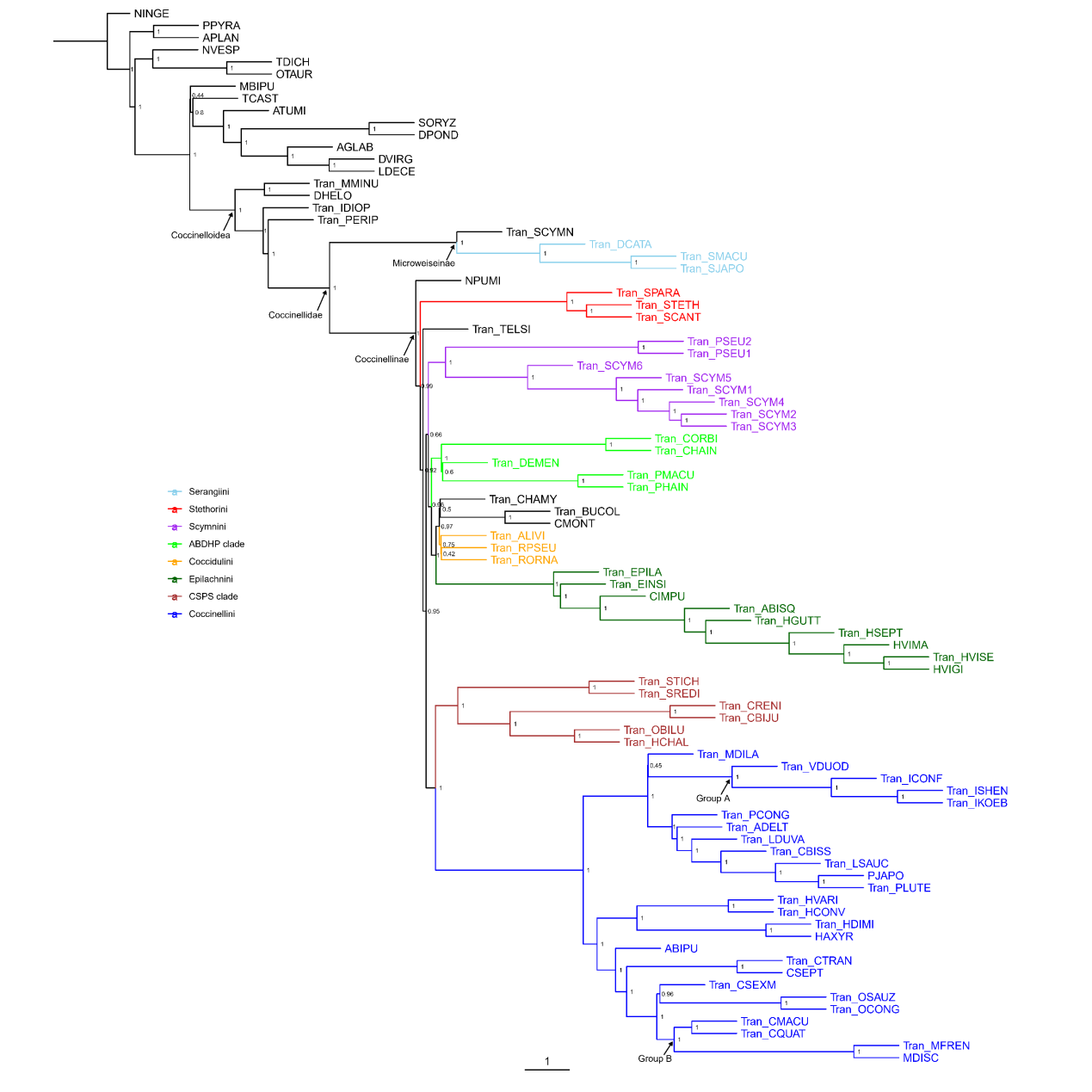


Figure S2.2 Phylogenetic tree of 69 ladybird (Coccinellidae) species and 18 outgroup species inferred by ASTRAL-Pro. Numbers beside nodes are local posterior probabilities. Internal branch lengths are in coalescent units, which represent a direct measure of the amount of discordance in the gene trees, while terminal branch lengths have no sense. The colored clades represent the six main tribes and two clades with a monophyletic origin containing at least three species in our data. Group A: the fungivorous ladybirds (former Psylloborini), Group B: the omnivorous ladybirds. A high-quality figure can be downloaded from https://github.com/huangyh45/ladybird-genomes-supplementary-figures.


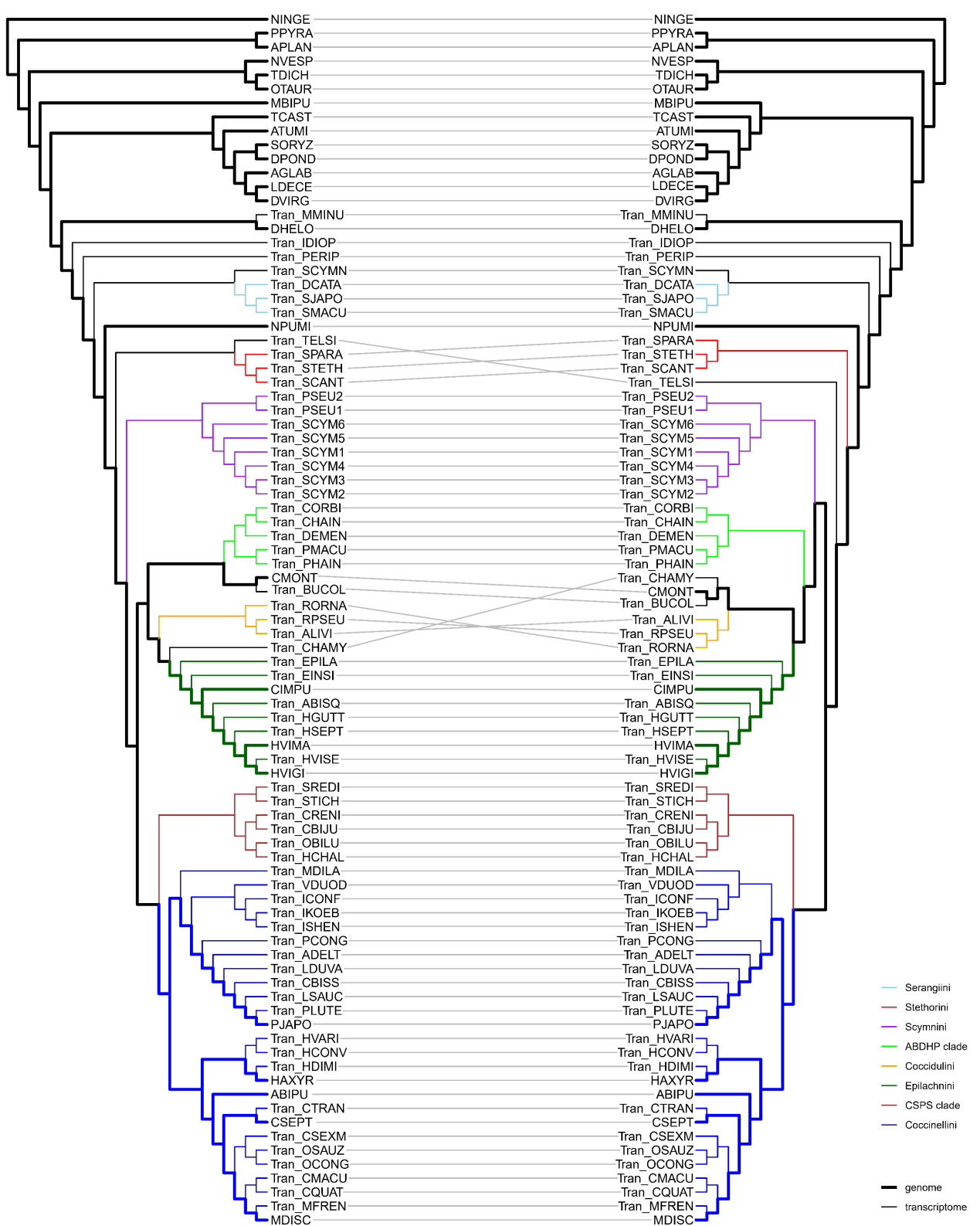


Figure S2.3 Comparison of the topologies of phylogenetic trees inferred by IQ-TREE (left) and ASTRAL-Pro (right). The colored clades represent the six main tribes and two clades with a monophyletic origin containing at least three species in our data. The bold branches represent the topologies of the species with the genomes. A high-quality figure can be downloaded from https://github.com/huangyh45/ladybird-genomes-supplementary-figures.


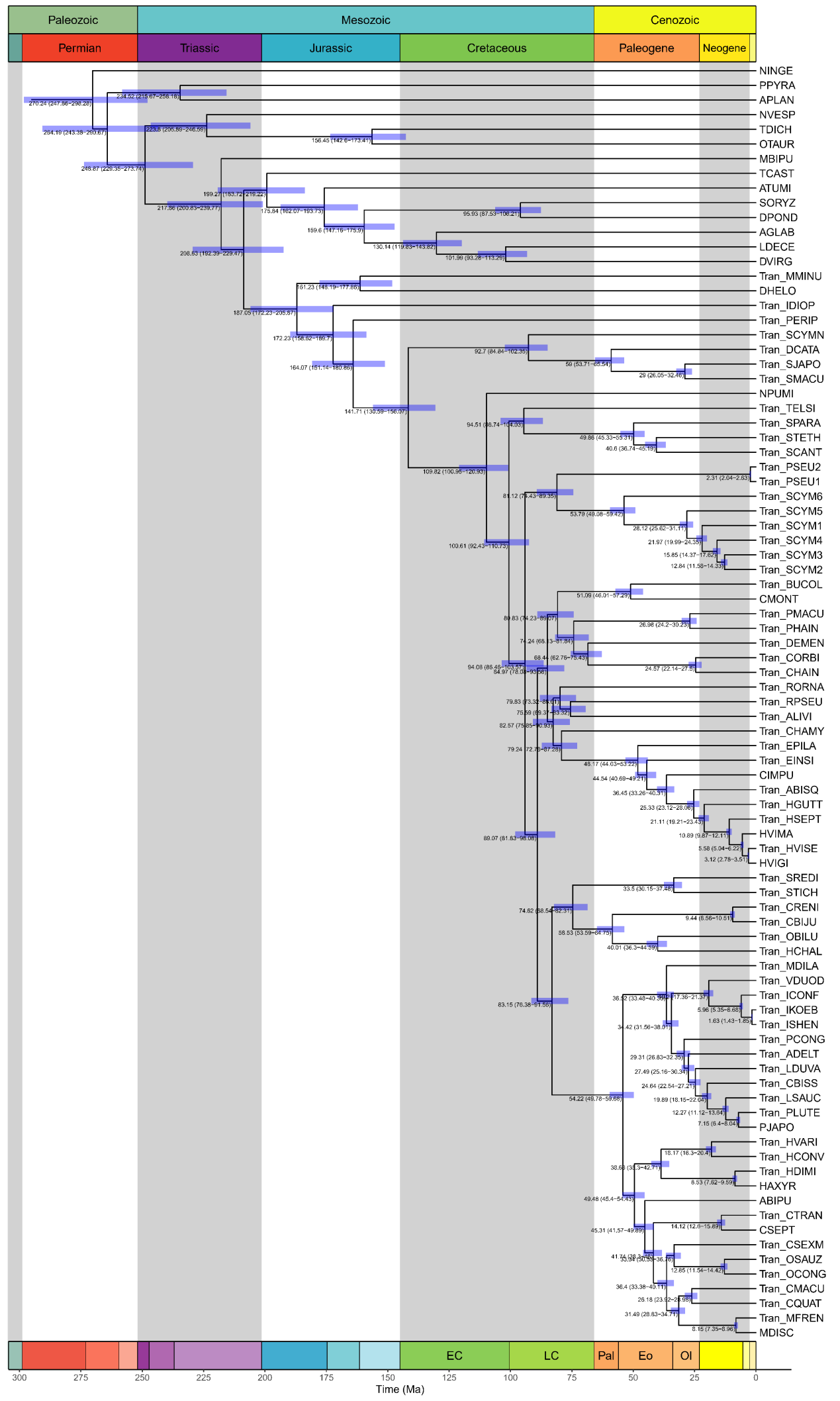


Figure S2.4 Divergence time tree of 69 ladybird (Coccinellidae) species and 18 outgroup species estimated by MCMCTREE. The blue bars on the nodes are the ranges of 95% credibility interval (CI). The text beside the nodes are the exact estimated time and the 95% CI. The first row of the blocks on the top means the eras, while the second row of the blocks on the top means the periods. The row of the blocks on the bottom represents the epochs, in which only the epochs in Cretaceous and Paleogene are differentiated and labeled with abbreviations. EC: Early Cretaceous, LC: Late Cretaceous, Pal: Paleocene, Eo: Eocene, Ol: Oligocene. A high-quality figure can be downloaded from https://github.com/huangyh45/ladybird-genomes-supplementary-figures.


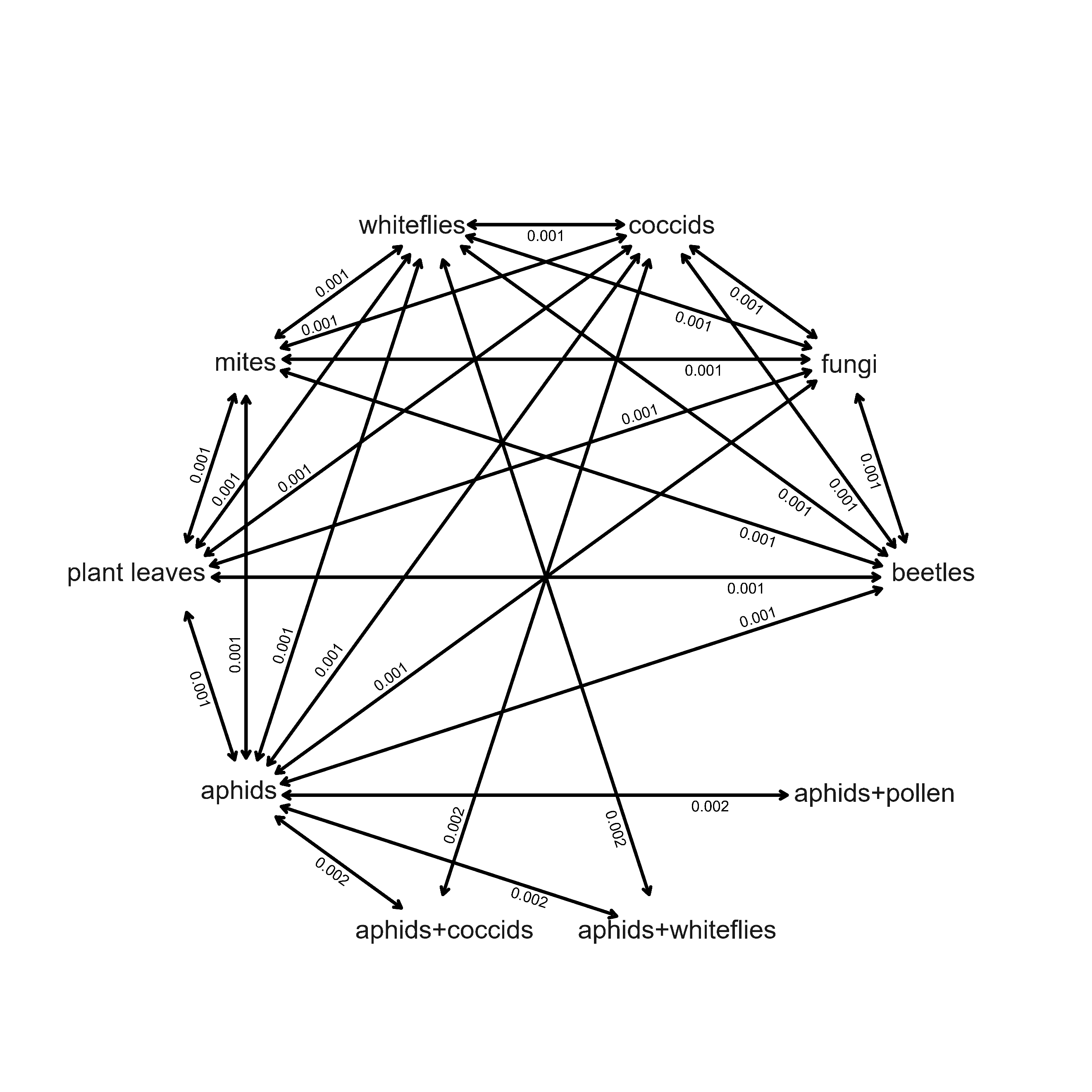


Figure S2.5 Best model of ancestral character reconstruction of diets within Coccinelloidea. The numbers represent transition rates between character states. A high-quality figure can be downloaded from https://github.com/huangyh45/ladybird-genomes-supplementary-figures.


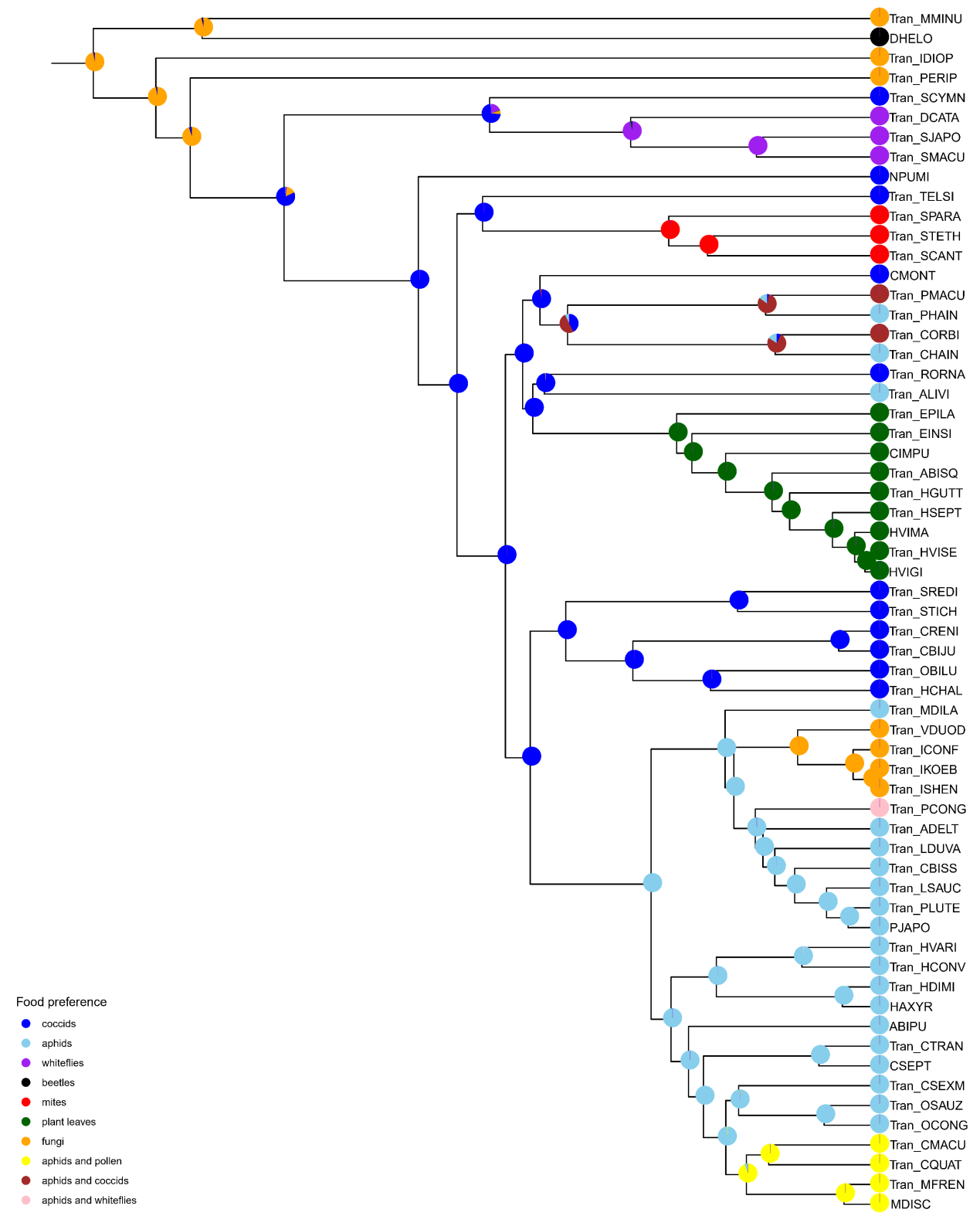


Figure S2.6 Stochastic character mapping of diets within Coccinelloidea using 61 species with known information. The area in the pie charts on the nodes represent the state probabilities of each character. A high-quality figure can be downloaded from https://github.com/huangyh45/ladybird-genomes-supplementary-figures.

# 3 Selection pressure of single-copy genes

## 3.1 Materials and methods

Selection pressure detection was conducted with the single-copy OGs in the genome dataset, from which PJAPO was excluded as mentioned in Section 1.3. The species tree used in the analysis was obtained through pruning the species tree reconstructed for the extended dataset in Section 2.2. Protein sequences of the single-copy OGs were first aligned by L-INS-i mode of MAFFT v7.480 (Katoh and Standley, 2013), and then back translated to codon alignments and trimmed by trimAl v1.4 (Capella-Gutierrez et al., 2009) for selection pressure detection.

We used the branch-site model (parameters: null hypothesis: model = 2, NSsites = 2, fix_omega = 1, omega = 1; alternative hypothesis: model = 2, NSsites = 2, fix_omega = 0, omega = 1) in CODEML of PAML v4.8a (Yang, 2007) to identify the genes with positively selected sites at specific node within the clade of ladybirds for single-copy ortholog sequences. The nodes of Coccinellidae (Node15 in Figure S3.1), Coccinellini (Node20), Epilachnini (Node18) and MDISC, along with diet shift or expansion, were labeled as the foreground branch respectively and then likelihood ratio tests (LRTs) were performed to detect positive selection on the foreground branch. LTR *P*-values were further adjusted for multiple testing using Benjamini-Hochberg Procedure. Only those genes with adjusted *P*-values < 0.05 were inferred as positively selected genes (genes with positively selected sites) (PSGs).

In addition, detection of selection relaxation or intensification was performed between branches of ladybirds with different feeding habits with the branch model (model = 2, NSsites = 0) in CODEML. The branches are divided into four sets, including coccidophagous ladybirds (C), aphidophagous ladybirds (Coccinellini) (A), herbivorous ladybirds (Epilachnini) (P) and outgroups (O) (Figure S3.1). Because MDISC is omnivorous, feeding on both aphids and pollen, we additionally compared branch of this omnivorous ladybird (AO) and other branches of aphidophagous ladybirds (Coccinellini) (AA). For alternative hypothesis, the branches of each two sets were labeled with #1 and #2, respectively, and other branches were considered as background branches. For null hypothesis, the branches of two sets in the target pair were both labeled with #1. LTR *P*-values were adjusted using Benjamini-Hochberg Procedure and adjusted *P*-values < 0.05 represent significant relaxation or intensification of selection of the set pairs. When dN/dS value of the former set was larger than that of the latter set in alternative hypothesis calculation, it was considered as selection relaxation, while dN/dS of the former set was smaller, it was considered as selection intensification. Furthermore, to avoid impact from extreme values of individual branches in the sets, we calculated the root-to-tip dN/dS value to check the difference between species in different sets. In detail, dN/dS value of each branch in the species tree was calculated by the free-ratio model (model = 1, NSsites = 0) in CODEML. Then root-to-tip dN/dS value of each species was obtained by averaging those dN/dS values of the branches according to the method described in Montgomery et al. (2011). If all the root-to-tip dN/dS values of the species in one set are all larger than those of the species in another set, it was considered as selection relaxation in this set pair, and if all the root-to-tip dN/dS values are smaller, it was considered as selection intensification. Only when the result of the branch model test and root-to-tip dN/dS values were detected, it was considered as exact event of selection relaxation or intensification. When the set or sets, in which all the ladybird species were contained (ladybird: Set C + A + P vs Set O) or all the species feed on a specific diet (coccid: Set C vs Set A + P + O; aphid: Set A vs Set C + P + O; Sternorrhyncha: Set C + A vs Set P + O), were detected experiencing significant relaxation or intensification compared with all other sets, this OG was considered to experience selection relaxation or intensification related to the ladybird clade or specific diet. Since several outgroup beetles feed on plant tissue, OGs with significant selection relaxation/intensification between pair of Set C vs P and pair of Set A vs P (plant: Set P vs Set C + A) were also considered to experience selection relaxation or intensification related to plant diet in ladybirds. The omnivorous ladybird MDISC were only compared with other Coccinellini species feeding on the aphids but not pollen (pollen: Set AO vs AA).

Functional enrichment was conducted by hypergeometric distribution testing using clusterProfiler package (Yu et al., 2012), with all single-copy OGs as background genes and the detected OGs as foreground genes. On the basis of the GO and Pfam annotations of OGs inferred by KinFin above in Section 1.1.7, GO or Pfam enrichment was performed. *P*-values were further adjusted for multiple testing using Benjamini-Hochberg Procedure without the cutoffs in order to observe all the enriched terms.

To explore another three nodes with diet shift, we fully utilized the extended dataset, and additionally constructed three small datasets to detect the selection pressure of other three clades with diet shift: Serangiini (whitefly feeding), Stethorini (mite feeding) and fungivorous ladybirds, respectively. For each clade, all species within the clade, a closest outgroup species and a closest outgroup species with genome were selected without causing different topology between the species trees built by IQ-TREE and ASTRAL-Pro (Figure S2.3), and then the small datasets were constructed using these species. That is, ((Tran_SCYMN, ((Tran_SMACU,Tran_SJAPO),Tran_DCATA)),NPUMI), (NPUMI,(((Tran_SCANT, Tran_STETH),Tran_SPARA),Tran_TELSI)) and (((((Tran_ISHEN,Tran_IKOEB),Tran_ICONF), Tran_VDUOD),Tran_MDILA),HAXYR), respectively. The PSGs at the nodes of ancestors of these three clades were detected using the same method mentioned above, and selection relaxation or intensification were also detected through comparison with the outgroups using the above method. The enrichment analyses were performed by clusterProfiler with all single-copy OGs of each small dataset as background genes.

## 3.2 Results

Totally 554 available single-copy OGs (three OGs were excluded due to no overlapped regions in the alignments) of the genome dataset without PJAPO were used to detect the selection pressure by CODEML (Yang, 2007). Details of results of selection pressure detection of the single-copy OGs can be found in Additional file 2: Table SE3 and the corresponding node numbers are shown in Figure S3.1.

Through branch-site model, 45 OGs are PSGs at the ancestor of ladybirds, 7 OGs are PSGs at the ancestor of Coccinellini, 6 OGs are PSGs at the ancestor of Epilachnini and 8 OGs are PSGs at the node of MDISC. These detected PSGs at four ladybird nodes are enriched in GO terms of protein binding, ATP binding, nucleic acid binding and protein kinase activity based on the annotations of OGs (Figure S3.2). Among the single-copy OGs possibly related to chemosensation, detoxification, digestion and immunity, two digestive genes, OG0006870 (lipase) and OG0006992 (fatty acid transport protein), are positively selected at the ancestor of Coccinellidae, while OG0007827 (chitin binding Peritrophin-A domain) is detected as PSG at the ancestor of Epilachnini (Additional file 2: Table SE3). OG0004069 (C-type lectin, possibly related to immunity) is detected as PSG at both the ancestors of Coccinellidae and Coccinellini.

Additionally, combining branch model test in CODEML and root-to-tip dN/dS comparison, we found 3 OGs under selection relaxation in the whole ladybird clade compared with outgroup beetles, 21 OGs under selection relaxation and 1 OG under selection intensification in the herbivorous ladybirds compared with other ladybirds, 7 OGs under selection relaxation and 4 OGs under selection intensification in MDISC compared with other Coccinellini species that cannot feed on pollen to complete the life circle (Table S3.1, Figure S3.3). But no selection relaxation or intensification related to aphid, coccid or Sternorrhyncha diet is found. In those plant-related relaxations, herbivorous outgroup beetles (mainly weevils, leaf beetles and buprestid beetles) also show high root-to-tip dN/dS values, which sometimes leads to close or even higher values than herbivorous ladybirds (e.g. OG0003982, OG0005460, OG0005463, OG0005474, OG0005722 and OG0007369) (Figure S3.3), supporting that these relaxations may be related to plant diet. Similarly, OG0006005 (C-terminal-binding protein) goes through selection relaxation in MDISC and also has highest root-to-tip dN/dS values in ATUMI, the outgroup beetle also eating pollen, which supports the relation to ability to complete life on pollen. However, all these relaxations or intensifications seem be more associated with general cellular process than adaptation to specific diet of ladybirds, such as transcription (OG0005722, OG0007369), translation (OG0007878), cell cycle (OG0007206), mitochondrial process (OG0001558, OG0006242, OG0007036) and ubiquitin-related process (OG0003486, OG0005474, OG0005550, OG0005563, OG0005619, OG0005096). Therefore, it is possible that these OGs are related to important change of other character like group-specific structure or speciation and thus accumulate or decrease relative amounts of harmful nonsynonymous mutations in the whole clade.

Using the extended dataset, we also constructed three small datasets to detect the selection pressure in another three nodes with diet shift, including Serangiini, Stethorini and fungivorous ladybirds (details in Additional file 2: Table SE3). Among 3454 available single-copy OGs in the dataset of Serangiini, 86 OGs are identified as PSGs at the ancestor, and 212 and 42 OGs are detected under selection relaxation and intensification respectively. In Stethorini feeding on mites, 18/3832 OGs are detected as PSGs at the ancestor, while 828 and 8 OGs are under relaxation and intensification in the whole clade. In fungivorous ladybirds, 3913 single-copy OGs are available, among which 2, 120 and 86 OGs are detected as PSGs, relaxation and intensification respectively. These OGs also include ABC transporters, major facilitator superfamily, serine-type endopeptidases, trypsins and sugar transporters (Figure S3.4), which are potentially connected to digestion and detoxification.

## 3.3 Summary

We detected positive selection and selection relaxation/intensification of 554 nuclear-encoded single-copy OGs. And we also explore the selection pressure in Serangiini, Stethorini and fungivorous ladybirds using the extended dataset. Among the single-copy OGs, the PSGs and genes with relaxation or intensification related to clades with the specific diet in ladybird seem to be mainly with function of general cellular process, which is hard to be explained about the evolution of feeding habits and is also possibly related to other characters like unique structures and group-specific pheromones. Actually, previous research found similar results that the genes under selection within the ladybird lineage were likely to be development-related (Zhu et al., 2023). Only few genes are putatively related to feeding habits with connection of detoxification or digestion, and detected in selection analyses in our study.

Table S3.1 Candidate single-copy orthogroups (OGs) undergoing selection relaxation or intensification related to whole ladybird clade or feeding habits in ladybirds.

| Orthogroup | Related, R/I | AvsC | PvsC | CvsO | PvsA | AvsO | PvsO | AOvsAA | Annotation |
| --- | --- | --- | --- | --- | --- | --- | --- | --- | --- |
| OG0003592 | ladybird, R | A>C | stable | C>O* | stable | A>O, *** | P>O, *** | AO<AA | probable inactive tRNA-specific adenosine deaminase-like protein 3 |
| OG0004919 | ladybird, R | stable | stable | C>O, *** | stable | A>O*** | P>O*** | AO<AA | VPS35 endosomal protein-sorting factor-like |
| OG0007206 | ladybird, R | stable | P<C | C>O** | P<A | A>O, *** | P>O, *** | AO<AA | cell cycle checkpoint control protein RAD9A-like |
| OG0001558 | plant, R | A>C | P>C*** | stable | P>A** | stable | P>O*** | stable | DNA polymerase subunit gamma-1, mitochondrial |
| OG0003486 | plant, R | stable | P>C*** | stable | P>A*** | stable | stable | AO>AA | ubiquitin-conjugating enzyme E2-22 kDa |
| OG0003982 | plant, R | stable | P>C*** | stable | P>A** | stable | stable | stable | serine/threonine-protein kinase Tor |
| OG0005460 | plant, R | stable | P>C*** | stable | P>A* | stable | stable | AO<AA | kinesin-like protein KIF21A |
| OG0005463 | plant, R | stable | P>C*** | stable | P>A*** | stable | stable | AO<AA | AN1-type zinc finger protein 2A |
| OG0005474 | plant, R | stable | P>C*** | stable | P>A** | stable | stable | AO<AA | E3 ubiquitin-protein ligase CHIP |
| OG0005487 | plant, R | A>C | P>C*** | stable | P>A*** | stable | stable | AO>AA | uncharacterized protein |
| OG0005550 | plant, R | stable | P>C** | stable | P>A** | stable | stable | AO<AA | ubiquitin carboxyl-terminal hydrolase |
| OG0005722 | plant, R | A>C*** | P>C*** | stable | P>A* | stable | stable | stable | general transcription factor IIE subunit 1 |
| OG0006045 | plant, R | stable | P>C* | stable | P>A** | stable | stable | stable | nucleoporin SEH1 |
| OG0006242 | plant, R | stable | P>C*** | stable | P>A** | stable | stable | AO<AA | probable 39S ribosomal protein L24, mitochondrial |
| OG0006589 | plant, R | stable | P>C*** | stable | P>A*** | stable | stable | stable | immunoglobulin domain and leucine-rich repeat-containing protein 2 |
| OG0007036 | plant, R | stable | P>C** | stable | P>A** | stable | stable | stable | mitochondrial import inner membrane translocase subunit Tim9 |
| OG0007230 | plant, R | stable | P>C* | stable | P>A* | stable | P>O*** | AO<AA | zinc finger protein 277 |
| OG0007369 | plant, R | A>C | P>C*** | stable | P>A*** | stable | stable | AO<AA | putative mediator of RNA polymerase II transcription subunit 26 |
| OG0007507 | plant, R | stable | P>C* | stable | P>A* | stable | stable | AO<AA | uncharacterized protein |
| OG0007526 | plant, R | stable | P>C* | stable | P>A* | stable | P>O*** | AO<AA | uncharacterized protein |
| OG0007702 | plant, R | stable | P>C** | stable | P>A** | stable | stable | AO>AA | tRNA-uridine aminocarboxypropyltransferase 1 |
| OG0007748 | plant, R | stable | P>C*** | stable | P>A*** | stable | P>O*** | AO<AA | protein TEX261 |
| OG0007956 | plant, R | stable | P>C** | stable | P>A* | stable | stable | stable | translocation protein SEC62 |
| OG0008503 | plant, R | stable | P>C** | stable | P>A* | stable | stable | AO<AA | calcium and integrin-binding protein 1-like |
| OG0005563 | plant, I | A>C | P<C* | stable | P<A** | A>O*** | stable | AO<AA | ubiquitin-conjugating enzyme E2 Q2 |
| OG0004541 | pollen, R | A>C | stable | stable | P<A | stable | stable | AO>AA* | interferon-related developmental regulator |
| OG0005408 | pollen, R | A>C*** | P>C** | stable | stable | stable | stable | AO>AA*** | cullin-1 |
| OG0005619 | pollen, R | stable | P>C** | stable | P>A | stable | stable | AO>AA** | (E3-independent) E2 ubiquitin-conjugating enzyme |
| OG0006005 | pollen, R | stable | P>C* | stable | stable | stable | stable | AO>AA* | C-terminal-binding protein |
| OG0006503 | pollen, R | stable | stable | stable | stable | stable | stable | AO>AA* | coatomer subunit beta |
| OG0007040 | pollen, R | stable | stable | C>O | stable | stable | P>O*** | AO>AA* | polypeptide N-acetylgalactosaminyltransferase 1-like |
| OG0007878 | pollen, R | stable | stable | stable | stable | stable | stable | AO>AA* | eukaryotic translation initiation factor 2 subunit 2 |
| OG0003622 | pollen, I | stable | stable | stable | stable | stable | stable | AO<AA** | myosin heavy chain, muscle |
| OG0004690 | pollen, I | stable | P>C** | stable | P>A | stable | stable | AO<AA* | YLP motif-containing protein 1-like |
| OG0005096 | pollen, I | A>C | P>C | stable | stable | stable | stable | AO<AA* | E3 ubiquitin-protein ligase Su(dx) |
| OG0005616 | pollen, I | A>C* | P>C | stable | P<A | stable | stable | AO<AA* | prefoldin subunit 6 |

‘>’ or ‘<’ of w (dN/dS) indicates both the whole clade w calculated by the branch model and all root-to-tip w of the species in this set are larger or smaller than those of the latter set respectively, otherwise it is ‘stable’

C: coccidophagous ladybirds

A: aphidophagous ladybirds

P: phytophagous ladybirds

O: outgroups

AO: omnivorous ladybirds

AA: aphidophagous and not omnivorous ladybirds

R: selection relaxation

I: indicates selection intensification

*: adjusted P-value < 0.05 in branch test

**: adjusted P-value < 0.01 in branch test

***: adjusted P-value < 0.001 in branch test


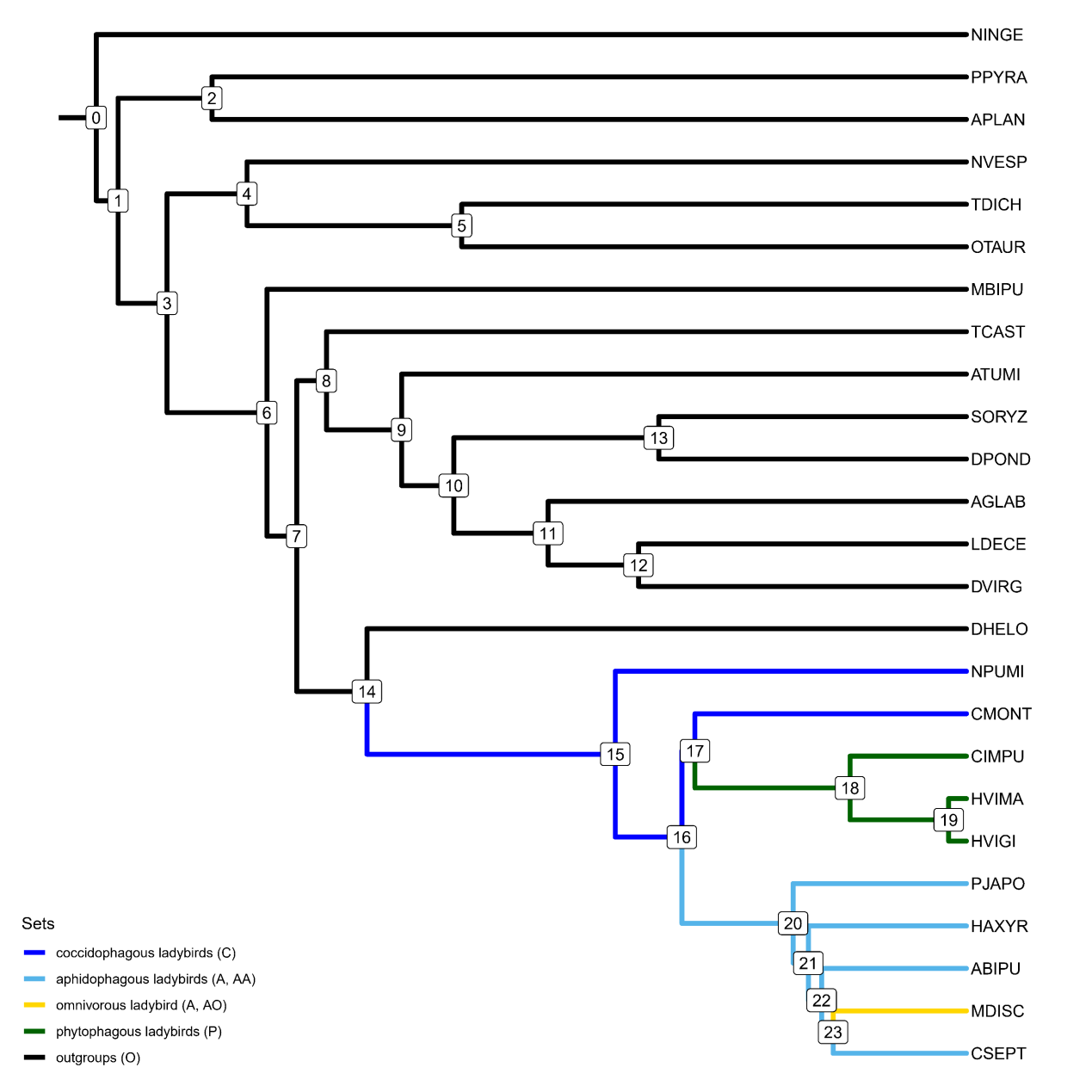


Figure S3.1 Node numbers of the species tree and different branch sets of ladybirds with different feeding habits for the genome dataset. The node numbers are used for selection pressure detection, orthogroup (OG) evolution analysis and domain rearrangement analysis. The branch sets will be used for detection of selection relaxation or intensification. A high-quality figure can be downloaded from https://github.com/huangyh45/ladybird-genomes-supplementary-figures.


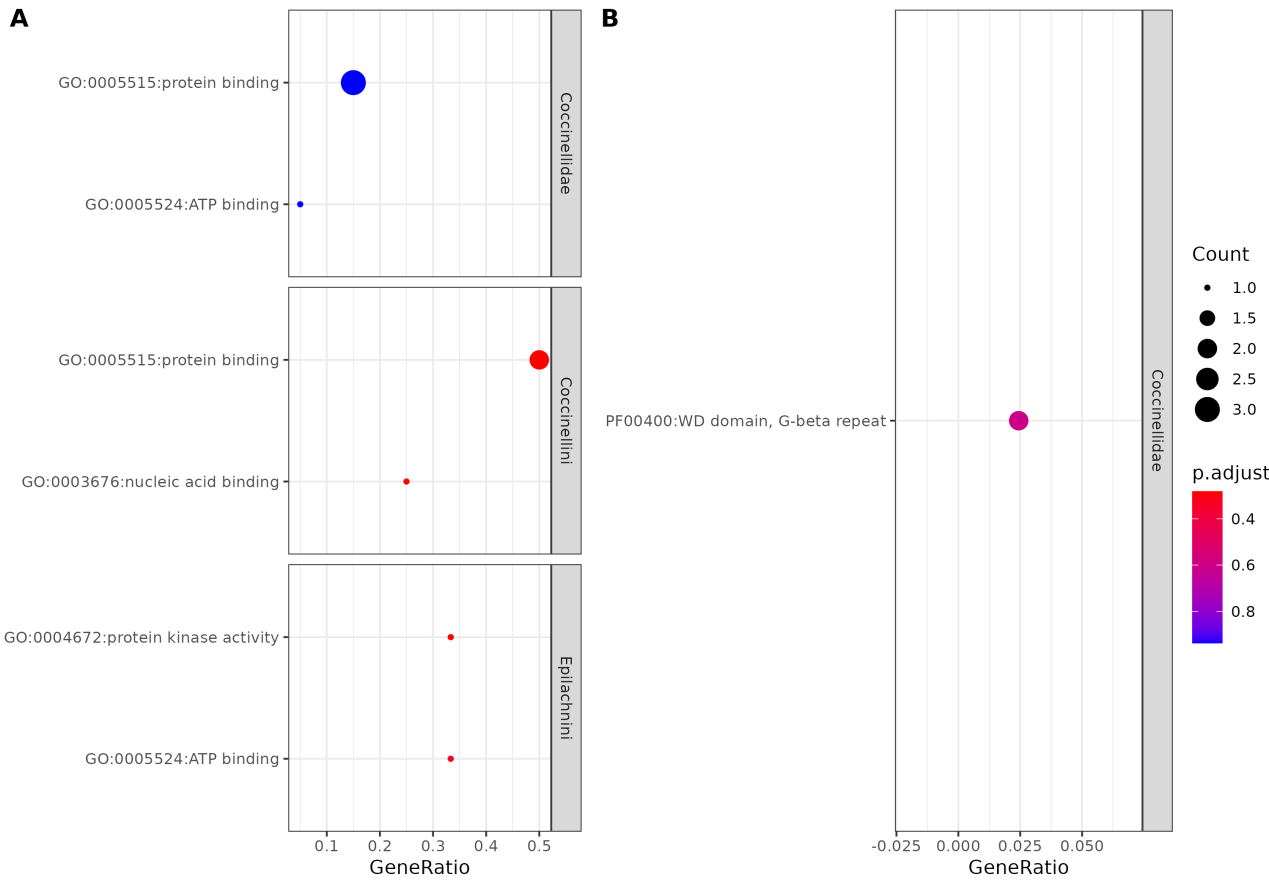


Figure S3.2 Enrichment of (A) gene ontology (GO) and (B) Pfam annotation of OGs with positively selected sites of ladybirds. A high-quality figure can be downloaded from https://github.com /huangyh45/ladybird-genomes-supplementary-figures.


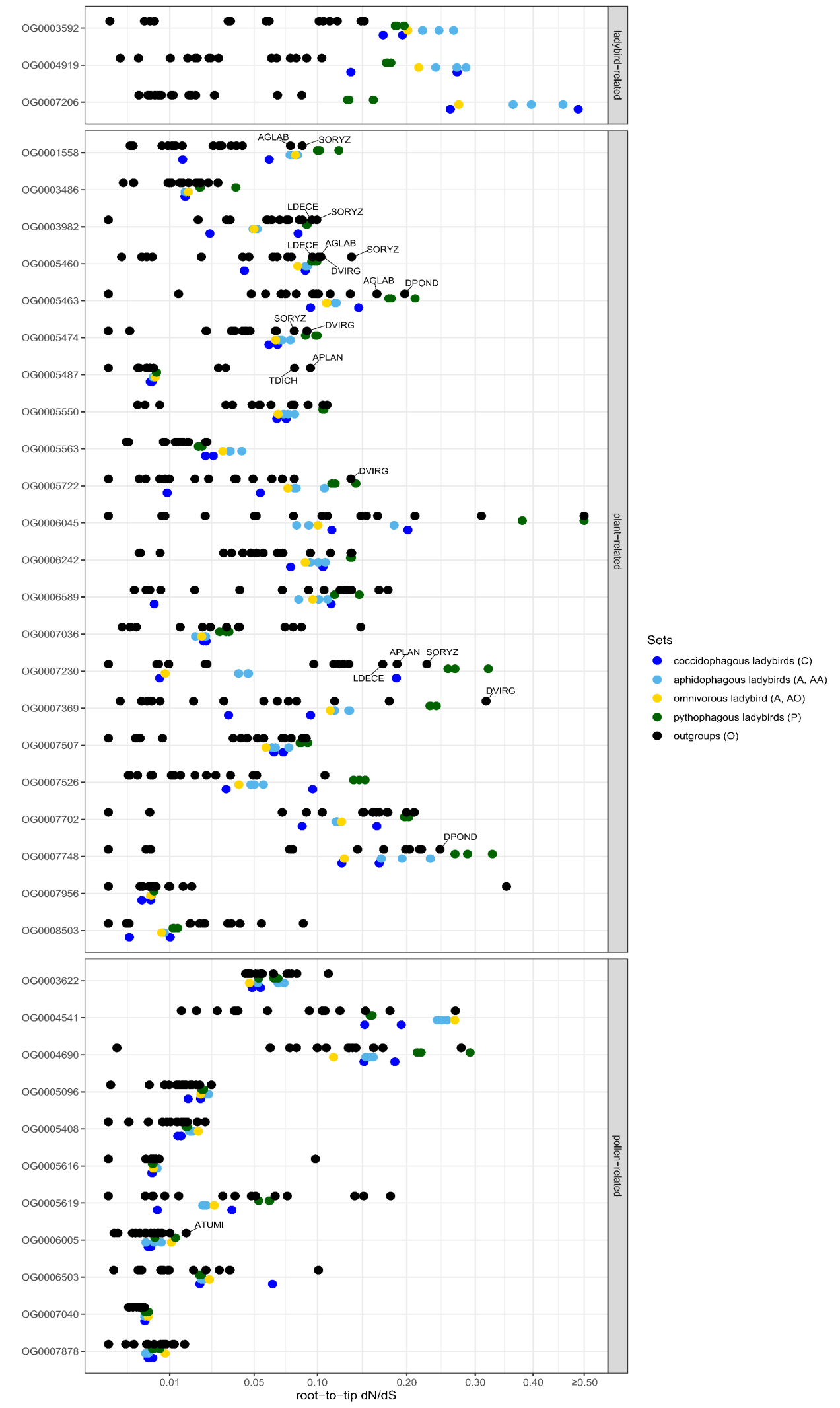


Figure S3.3 Root-to-tip dN/dS of each species of the candidate single-copy ortholog groups (OGs) under selection relaxation or intensification related to the whole ladybird clade, plant diet or pollen diet in the ladybirds. The x axis is uneven due to sqrt-transform. The information of these OGs is in Table S3.1. A high-quality figure can be downloaded from https://github.com/huangyh45/ladybird-genomes-supplementary-figures.


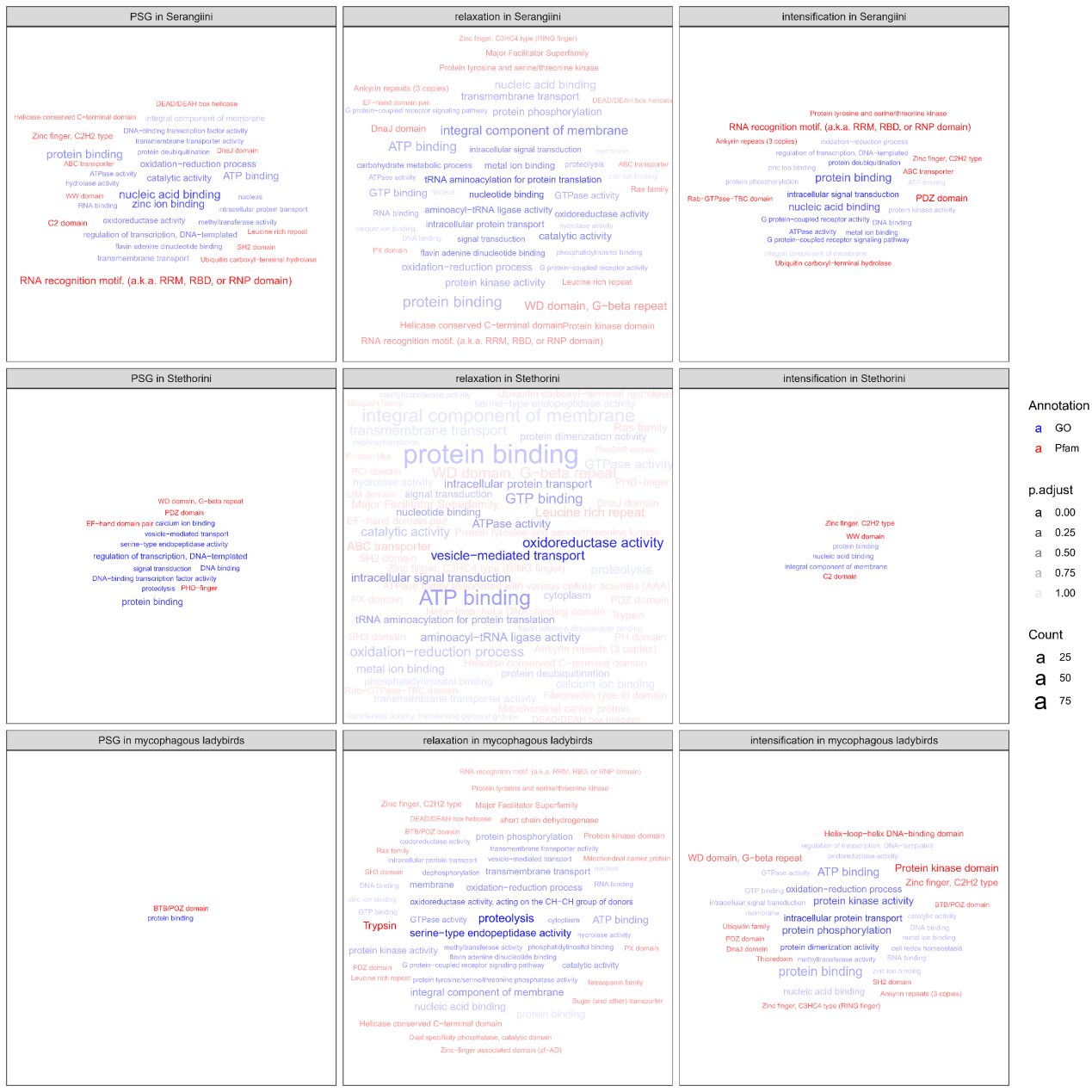


Figure S3.4 Enrichment of gene ontology (GO) or Pfam annotation of the ortholog groups (OGs) undergoing positive selection, selection relaxation or intensification at the nodes of Serangiini, Stethorini or fungivorous ladybirds using extended dataset. Only the top 30 GO or Pfam annotations in the enrichment results are shown. PSG: positively selected genes (genes with positively selected sites). A high-quality figure can be downloaded from https://github.com/huangyh45/ladybird-genomes-supplementary-figures.

# 4 Gene count evolution of ortholog groups

## 4.1 Materials and methods

Genome-wide scan of OG evolution was performed with the genome dataset. The gene counts of OGs of the genome dataset, from which PJAPO was excluded as mentioned in Section 1.3, were input to CAFE v5.0 (Mendes et al., 2020) to assess the contractions and expansions using the birth/death parameter (λ) without gamma modelling. To avoid the impact on incorrect gene family counts caused by assembly quality or assignment accuracy, error model was estimated and applied to the input data. The species tree used in CAFE was obtained through pruning the time tree estimated for the extended dataset in Section 2.2. OGs that did not exist at the root were also included in the analysis. In each branch, OGs with *P*-values < 0.05 were considered significant expansions or contractions.

Furthermore, based on the gene counts of the whole genome dataset (including PJAPO), those OGs with gene counts only in specific species or all species in specific clades were considered as emergence, while those OGs with genes only lost in specific species or all species in specific clades were considered as loss. The OGs with significant expansions, significant contractions, emergences and losses were identified as lineage-specific evolving gene families (LEGFs). Additionally, the OGs only present or absent in all carnivorous ladybirds (ladybirds except Epilachnini) or coccidophagous ladybirds (NPUMI and CMONT) in the genome dataset were also explored.

Functional enrichment of LEGFs was conducted by hypergeometric distribution testing using clusterProfiler package (Yu et al., 2012), with the Pfam and GO annotations respectively of OGs inferred by KinFin above in Section 1.1.7. *P*-values were further adjusted for multiple testing using Benjamini-Hochberg Procedure without the cutoffs in order to observe all the enriched terms.

In addition, to fully utilize our transcriptomic data, we also observed the group-specific emergences and losses of the OGs in the extended dataset. The OGs only present or only absent in all the ladybirds compared with the outgroups were explored, while the OGs only present or only absent in Coccinellinae (= reverse results of Microweiseinae), Serangiini, Stethorini, Epilachnini and Coccinellini respectively but not in other ladybirds were also found. In consideration of the feeding habits, we additionally observed the OGs only present or absent in fungivorous ladybirds (former Psylloborini) and omnivorous ladybirds (MDISC, Tran_MFREN, Tran_CQUAT and Tran_CMACU) compared with other ladybirds.

## 4.2 Results

Among 20,862 OGs from the genome dataset, CAFE (Mendes et al., 2020) analysis (without PJAPO) and basic statistics of gene counts found totally 13,343 (63.96%) LEGFs (significant expansions, significant contractions, emergences and losses) of all nodes, including 7,610 (36.48%) LEGFs identified in any nodes of ladybirds (Additional file 2: Table SE3).

Among the terminal nodes, the first ten with most LEGFs include seven nodes of ladybird species, along with three nodes of carnivorous outgroup species, MBIPU, DHELO and PPYRA (Figure S4.1). These large amounts of LEGFs are mostly contributed by species-specific emergences. Additionally, the nodes of ancestor of HVIGI and HVIMA and ancestor of Epilachnini have the largest number of LEGFs among the inner nodes. The LEGFs at the inner nodes within the ladybird clade are mainly caused by emergences and expansions.

Subsequently, we mainly focused on the LEGFs at the nodes with diet shifts or expansions (the ancestors of Coccinellidae, Epilachnini, Coccinellini and MDISC, respectively), and thus conducted the GO and Pfam enrichments of the OGs with emergences, losses, significant expansions and significant contractions at these four nodes, respectively. And we also performed the enrichment analyses of the OGs only present or absent in carnivorous ladybirds or coccidophagous ladybirds. The enrichments of GO and Pfam terms reveal that these LEGFs or present/absent OGs can be mainly enriched in the functions related to feeding habits, such as chemosensation (odorant-binding protein (OBP), odorant receptor (OR), 7tm chemosensory receptor, insect pheromone-binding family), digestion (several peptidases, transporters and symporters, cysteine proteases, trypsins, Glycosyl hydrolases family 18 (GH18, chitinase), major facilitator superfamily, chitin binding Peritrophin-A domain (CBPD)), detoxification (ATP-binding cassette transporter (ABC), cytochrome P450 monooxygenase (P450), glutathione S-transferase (GST), UDP-glucuronosyltransferase (UGT), carboxylesterases, aldo-keto reductase (AKR), GMC oxidoreductase) and immunity (C-type lectin (CTL), serpin) (Figure S4.2).

For the expanding OGs, 24 OGs expand both in Coccinellidae and Epilachnini, including serpin, OBP and sugar transport, while 28 OGs expand both in Epilachnini and the omnivorous MDISC, including UGT, indication important role of detoxifying enzymes in plant tissue feeding in the ladybirds (Figure S4.3). And 136 OGs expand only in Epilachnini, mainly related to large amounts of detoxifying genes such as P450, GMC oxidoreductase and AKR. Additionally, totally 7,049 OGs are present in all the ten ladybirds, mainly related to general cellular process, such as ATPase family associated with various cellular activities (AAA), Helix-loop-helix DNA-binding domain, WD domain, G-beta repeat, Helicase conserved C-terminal domain and FERM central domain (Figure S4.4). 221 OGs mainly related to serpin, Haemolymph juvenile hormone binding protein (JHBP), trypsin and insect pheromone-binding family are only present in three Epilachnini species compared with carnivorous ladybirds. 8 OGs are only present in Epilachnini and MDISC, without clear function. In five Coccinellini species (including MDISC), 50 OGs are absent in other ladybirds, including cathepsin propeptide inhibitor and OBPs. 25 OGs are specifical in two coccidophagous ladybirds, mainly related to ecdysteroid kinase and THAP domain. These expanding and specifical OGs in groups with different feeding habits include relative amounts of genes possibly related to chemosensation, digestion, detoxification and immunity.

In the extended dataset, we also found five OGs only lost in the ladybirds including genes encoding two transporters with putatively digestive function including major facilitator superfamily and Sodium:neurotransmitter symporter family (Additional file 2: Table SE3). Furthermore, compared with Microweiseinae, eleven emergent OGs are found in Coccinellinae, while 62 OGs are lost. In Serangiini which mainly feeds on whiteflies, 65 OGs are emergent and seven are lost compared with other ladybirds. Nineteen OGs are emergent in Stethorini, which feeds on mites, instead of other ladybirds. And eight OGs are emergent in the herbivorous ladybirds, Epilachnini, while two OGs are emergent in Coccinellini, without existence in other ladybirds. Additionally, 89 and two OGs are detected to be emergent in fungivorous ladybirds and omnivorous ladybirds respectively. These emergent or lost OGs include several genes related to chemosensation (OBPs, insect pheromone-binding family), digestion (transporters and peptidases, lipases, CBPDs), detoxification (UGTs, P450s and carboxylesterases) and immunity (gram-negative binding proteins (GNBPs), serpins and I-type lysozymes (ILYSs)).

## ­­­­4.3 Summary

Combining OG assignment by OrthoFinder with CAFE analysis, we identified the LEGFs on each node of the beetle genomes, including significant expansions, significant contractions, emergences and losses. These LEGFs were enriched in the functions like chemosensation, digestion, detoxification and immunity, which are possibly related to feeding habits of the ladybirds. Combining the transcriptomic data from different species, we also found OGs only emergent or lost in the specific ladybird groups with different feeding habits, which similarly include several OGs with putative function of chemosensation, digestion, detoxification and immunity. However, because unexpressed genes are absent in the transcriptomes, exact emergences and losses of these genes in the ladybird genomes needs further verification.


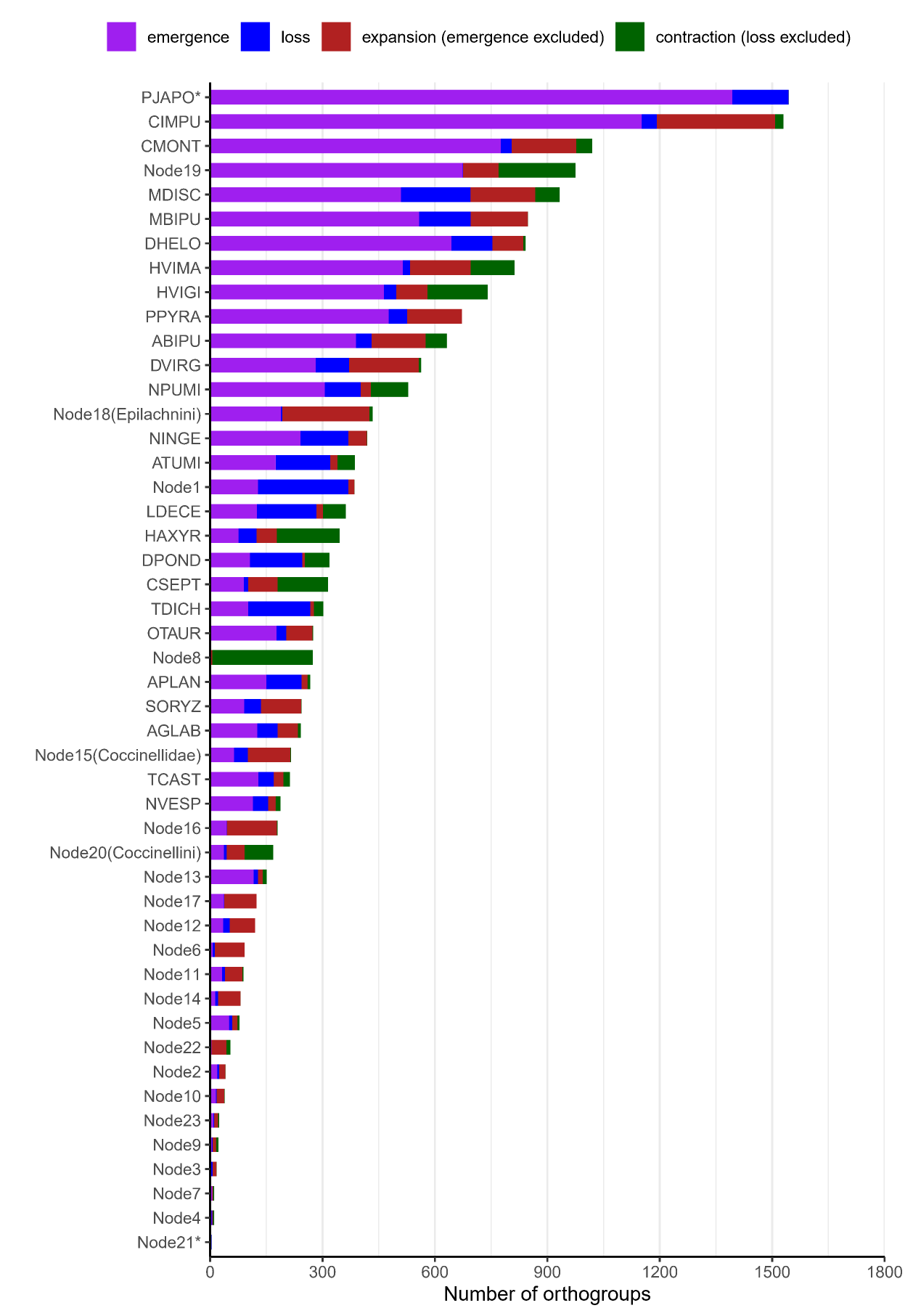


Figure S4.1 Number of Orthogroups (OGs) with significant expansions, significant contractions, emergences and losses in each node. The OGs with emergences are not included in the OGs with significant expansions, and the OGs with losses are not included in the OGs with significant contractions. The node IDs are shown in Figure S3.1. *: only emergences and losses were analyzed because PJAPO is excluded from the expansion and contraction analysis due to large amounts of gene duplications. A high-quality figure can be downloaded from https://github.com/huangyh45/ ladybird-genomes-supplementary-figures.


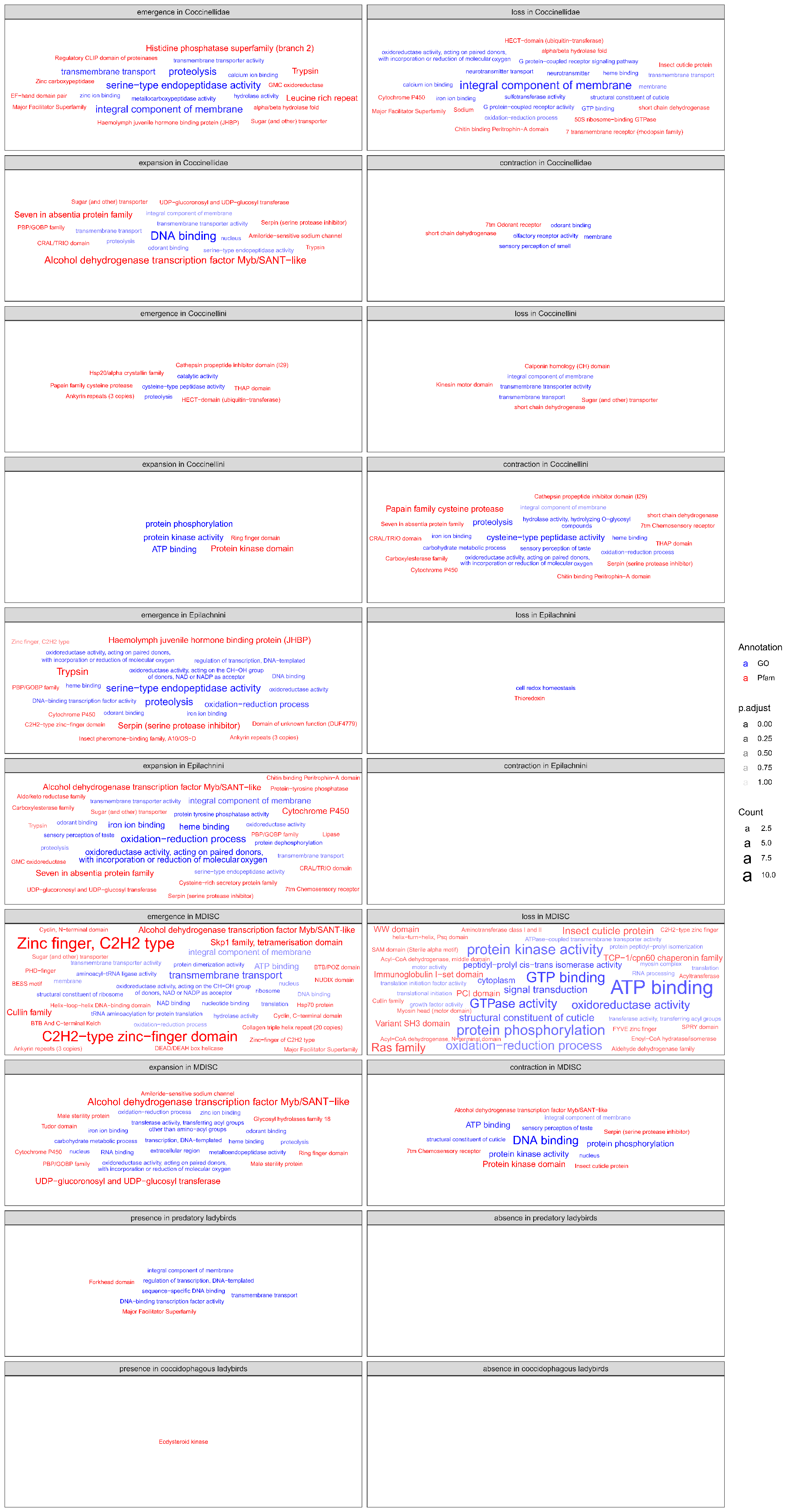


Figure S4.2 Enrichment of gene ontology (GO) or Pfam annotation of lineage-specific evolving gene families (LEGFs) and present/absent ortholog groups (OGs) at the nodes or groups within ladybird clade related to diet shift. Only the top 20 GO or Pfam annotations in the enrichment results are shown. A high-quality figure can be downloaded from https://github.com/huangyh45/ladybird-genomes-supplementary-figures.


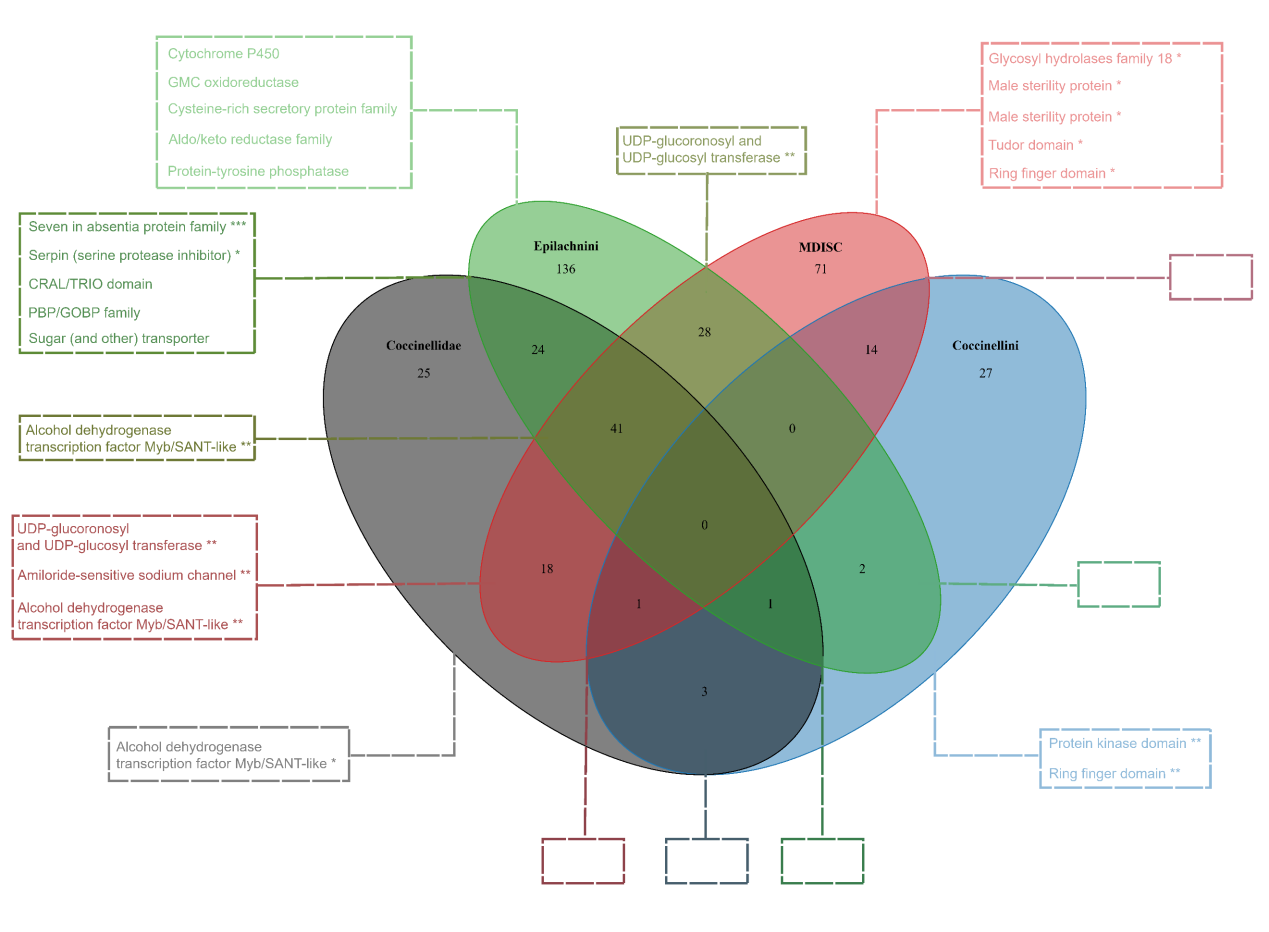


Figure S4.3 The expanding OGs and enrichment of Pfam annotation of them at the nodes or groups within ladybird clade related to diet shift. Only the top 5 Pfam annotations in the enrichment results are shown. *: adjusted P-value < 0.05, **: adjusted P-value < 0.01, ***: adjusted P-value < 0.001. A high-quality figure can be downloaded from https://github.com/huangyh45/ladybird-genomes-supplementary-figures.


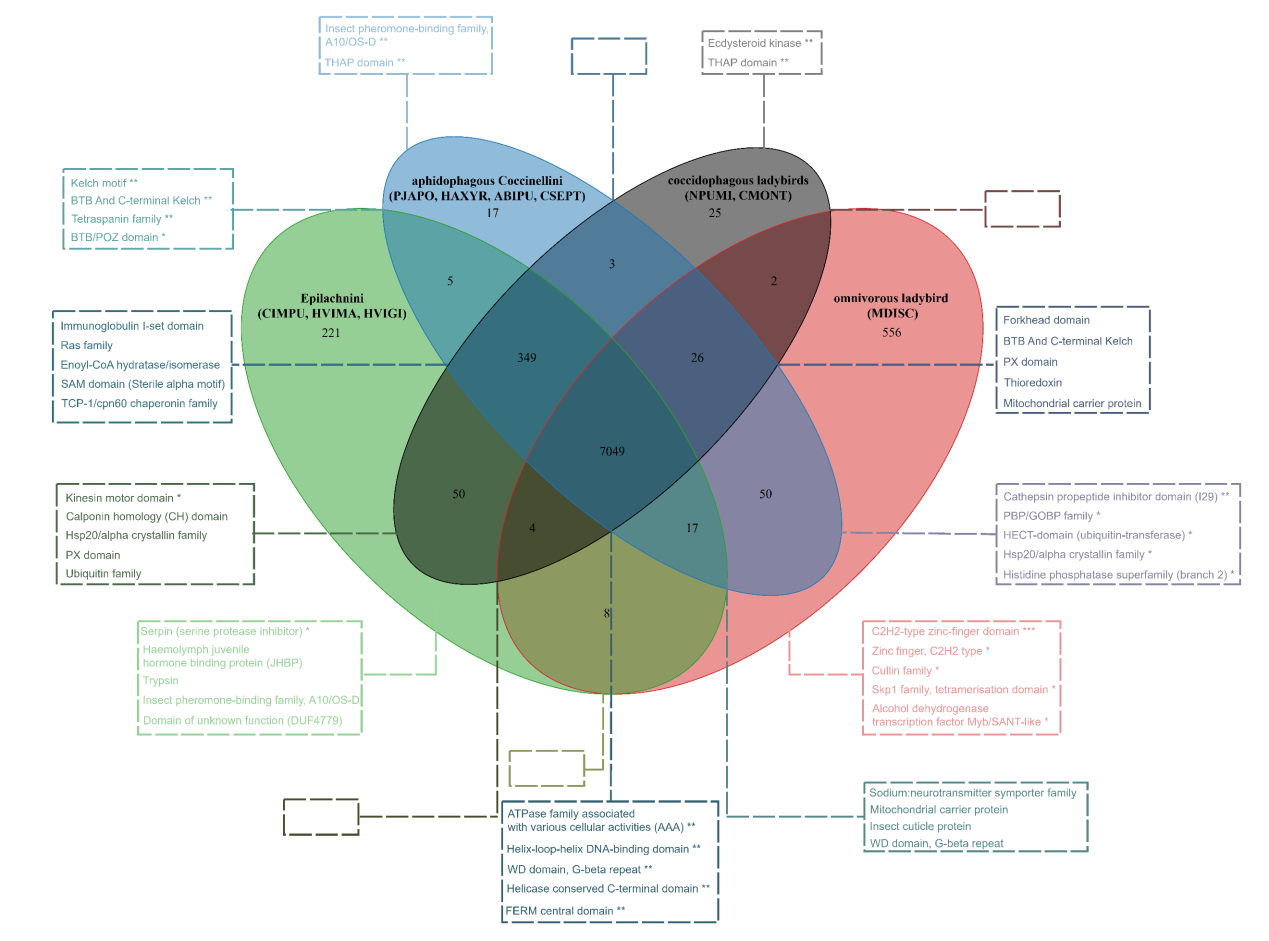


Figure S4.4 The OG existence and enrichment of Pfam annotation of them at the nodes or groups within ladybird clade related to diet shift. For Epilachnini, aphidophagous and coccidophagous ladybirds including several species, only the OGs present or absent in all species are considered in the picture. Only the top 5 Pfam annotations in the enrichment results are shown. *: adjusted P-value < 0.05, **: adjusted P-value < 0.01, ***: adjusted P-value < 0.001. A high-quality figure can be downloaded from https://github.com/huangyh45/ladybird-genomes-supplementary-figures.


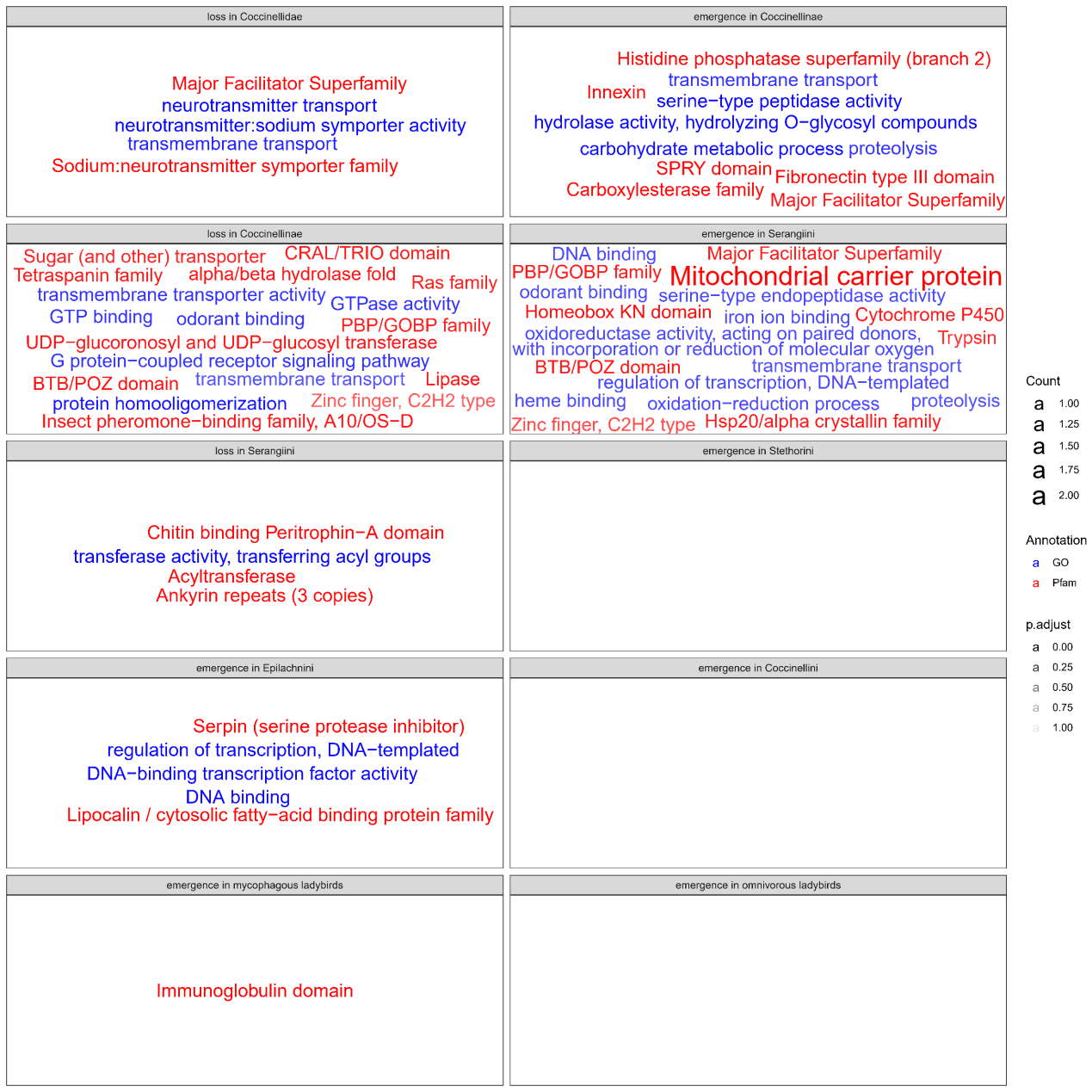


Figure S4.5 Enrichment of gene ontology (GO) or Pfam annotation of emergent or lost ortholog groups (OGs) at the nodes within ladybird clade related to diet shift using the extended data. The emergent and lost OGs of Coccinellidae are obtained by comparison with the outgroups, and the emergent and lost OGs of other nodes are obtained by comparison within the ladybirds. A high-quality figure can be downloaded from https://github.com/huangyh45/ladybird-genomes-supplementary-figures.

# 5 Diet-specific transcriptome comparison

## 5.1 Materials and methods

### 5.1.1 Insect rearing and sample collection

Five carnivorous ladybird species included in the genome dataset, CMONT, PJAPO, MDISC, CSEPT and HAXYR, and Tran_LSAUC in the extended dataset, were fed different diets respectively, including APHID (the aphid *Aphis craccivora* for MDISC and *Megoura japonica* for the rest), MEALYBUG (the mealybug *Planococcus citri*), MOTHEGG (eggs of the flour moth *Ephestia kuehniella*) and POLLEN (pollen of *Brassica campestris*) (Table S5.1, Additional file 2: Table SE2). These diets were the main or alternative diets from the nature, which could support the life cycle of the ladybirds. Some of these transcriptomes were published and we added some comparisons in this study. In the experiments, newly hatched ladybird larvae were fed the diets and each individual was kept in a separate plastic Petri dish (3.5 cm diameter and 1.2 cm height) in climatic chambers set at 25 ± 1 °C with a relative humidity (RH) of 75 ± 5% and a photoperiod of 14:10 (L:D) h. Two or three fourth instar larvae (~24 h after molting) of the ladybirds from each diet treatment were collected for transcriptome analysis and frozen in liquid nitrogen or at −80 °C before RNA extraction. Additionally, we also collected other diet-specific transcriptome comparisons of carnivorous ladybirds previously published, in order to extend our knowledge.

In addition, to explore the herbivory of HVIGI, we also designed diet-specific experiments. The male adults of the ladybirds which were reared using leaves of *Solanum nigrum* from larvae, were randomly divided into three groups and fed still leaves of *S. nigrum* (PLANT), sugar water for one day (SUGAR1d) and five days (SUGAR5d), respectively. Each individual was kept, collected and frozen in the same way as the carnivorous ladybirds.

### 5.1.2 Transcriptome sequencing and analysis

RNA was extracted from the whole body of each collected individual, and the libraries were constructed and sequenced on an Illumina HiSeq 2500 platform with a 6-Gb sequencing depth for each individual. Adaptors and low-quality sequences were removed using the default settings for Trimmomatic v0.36 (Bolger et al., 2014).

Subsequently, the clean reads of each sample were mapped to the genome of corresponding species using HISAT2 v2.2.0 (Kim et al., 2019). Gene-level abundance estimation was performed using StringTie v2.1.4 (Kovaka et al., 2019) based on genome predictions. For Tran_LSAUC without genomes, the coding sequences (CDSs) obtained by EvidentialGene in Section 1.1.6 was used as reference to map the reads by Bowtie2 v2.3.5 (Langmead and Salzberg, 2012) and then estimate abundance by RSEM v1.3.3 (Li and Dewey, 2011). The coefficient of determination (*r*^2^) from Pearson’s correlation analysis was used to analyze the relationship of each sample pair based on fragments per kilobase of transcript per million mapped reads (FPKM) values calculated by StringTie or RSEM, from which the values of genes with mean values < 1 were filtered out. Differential expression analysis between treatments was performed using DESeq2 (Love et al., 2014) according to the standard workflow, with a log2(fold change) (log2FC) value > 1 or < -1 and an adjusted *P*-value (*Q*-value) < 0.05 used as the criteria for defining differentially expressed genes (DEGs). The OG enrichment was then conducted by clusterProfiler package (Yu et al., 2012) with the adjusted *P*-value cutoff of 0.05 based on the OG assignment results (gene-to-OG relationships) obtained in Section 1.1.7. For our comparisons between APHID, MEALYBBUG, MOTHEGG and POLLEN treatments, we further connected the DEGs with the evolution events at the nodes of Coccinellidae, Coccinellini or MDISC detected in Section 3-4. The GO and Pfam enrichment in HVIGI were also performed based on the annotations of HVIGI. And those genes with both differential expression and evolution events were also used to perform GO and Pfam enrichments by clusterProfiler package in each species, respectively.

## 5.2 Results

### 5.2.1 Diet-specific differentially expressed genes in six carnivorous ladybirds

We selected totally fourteen diet-specific transcriptome comparisons of the larvae of six carnivorous ladybirds (Table S5.1). The *r*^2^ values for the relative gene expression among the replicates of each treatment are all larger than 0.7, mostly ranging from 0.86-0.99 (Figure S5.1), revealing relatively close expression patterns and high repeatability within treatments. We have enriched the GO or KEGG terms in most these comparisons in previous studies and always found that when ladybirds adapted to new diets the functions related to chemosensation, digestion, detoxification and immunity are significantly enriched (Chen et al., 2020; Huang et al., 2022; Li et al., 2024; Li et al., 2021a; Li et al., 2016a). To connect the evolution of feeding habits within the ladybirds with their expression regulation, in this study we performed an OG enrichment analysis to those DEGs found in the transcriptome comparisons. 52 OGs are significantly enriched (adjusted *p*<0.05) from the DEGs in at least one transcriptome comparison in the fourteen diet-specific comparisons we selected (Additional file 2: Table SE4, the corresponding node numbers are shown in Figure S3.1). Among the seventeen OGs significantly enriched (adjusted *p*<0.05) in at least two comparisons (Table S5.2), ten OGs are the members of LEGFs at the nodes where the ladybird ancestors change their diets (Coccinellidae, Coccinellini or MDISC). The seventeen OGs include two OGs potentially related to chemosensation (PBP/GOBP family, insect pheromone-binding family), five OGs related to protein, lipid or carbohydrate metabolism (facilitated trehalose transporter Tret1-like, fatty acid synthase-like, procathepsin L-like, fatty acyl-CoA reductase wat-like, glycosyl hydrolases family 18 (GH18, chitinase)), five OGs related to detoxification (cytochrome P450 (P450), UDP-glycosyltransferase (UGT)), one OG related to immunity (coleoptericin) and two OGs related to development (insect cuticle protein, ecdysteroid kinase).

Using all data of 143 diet-specific transcriptome comparisons of the carnivorous ladybirds, we also found sixteen OGs significantly (adjusted *p*<0.05) enriched from the DEGs in at least two species (Figure S5.3). Among the sixteen OGs, at least eight OGs undergo evolution events (emergence, expansion, loss and contraction) at the three nodes along with diet shift (Coccinellidae, Coccinellini or MDISC), and these sixteen OGs include two OGs related to chemosensation (PBP/GOBP family, insect pheromone-binding family), four OGs related to protein, lipid or carbohydrate digestion (facilitated trehalose transporter Tret1-like, procathepsin L-like, fatty acyl-CoA reductase wat-like, major facilitator superfamily), five OGs related to detoxification (P450, UGT), and two OGs related to development (insect cuticle protein, ecdysteroid kinase), which are mostly the same as the OGs significantly enriched in at least two comparisons among the fourteen selected comparisons.

Further, we explored the connection between the comparisons related to the specific diets and the evolution events occurring at the specific nodes with diet shift. The comparisons of APHID vs MEALYBUG in six carnivorous ladybirds reveal that, large amounts of DEGs belong to the OGs under evolution events at the ancestor of Coccinellidae or Coccinellini (Figure S5.2). These DEGs can be significantly enriched in GO or Pfam annotations including those related to chemosensation (odorant binding, PBP/GOBP family), digestion (peptidases, lipocalin, sugar transporter, papain family cysteine protease, trypsin), detoxification (UGT, glutathione S-transferase (GST), carboxylesterase). According to the counts of the DEGs, expansion in Coccinellidae and contraction in Coccinellini tend to contribute to upregulation to aphid diet, while emergence in Coccinellidae is likely to cause upregulation to the mealybug diet. Ratio of DEGs also shows that genes under evolution events at Coccinellidae tend to be upregulated in aphid diet or mealybug diet, and genes under evolution at Coccinellini also slightly tend to be upregulated in aphid diet. Additionally, the OGs with at least one upregulated DEGs in APHID vs MEALYBUG of four Coccinellini species with genomes (CSEPT, HAXYR, CSEPT and MDISC) include P450, major facilitator superfamily, juvenile hormone acid O-methyltransferase-like, glucose dehydrogenase [FAD, quinone]-like (GDH), multifunctional protein ADE2 and trifunctional purine biosynthetic protein adenosine-3 (Table S5.4), among which OG0000068 of major facilitator superfamily significantly expands in Coccinellidae and OG0003569 of trifunctional purine biosynthetic protein adenosine-3 (*GART*) is single-copy and positively selected at both Coccinellidae and Coccinellini ancestor. Interestingly, *GART* is also positively selected at the ancestor of whitefly feeding Serangiini and under selection relaxation at the ancestor of mite feeding Stethorini (Additional file 2: Table SE3), which indicates its potential role in carnivory to different Sternorrhyncha insects. It is coincidental that, *GART* is horizontally tranferred from bacteria to the ancestor of Sternorrhyncha (at least MRCA of aphids and coccids) (Tang et al., 2023b). Evolution of *GART* in both ladybirds and prey reveals similar demand of ability to synthesize purine, possibly due to management of some substances from plant to Sternorrhyncha insects and then the ladybirds.

In comparisons of Sternorrhyncha diet (APHID and MEALYBUG) versus other diet (MOTHEGG and POLLEN) in three carnivorous ladybirds, the DEGs also contain the genes belonging to the OGs under evolution at Coccinellidae ancestor (the node when the ladybirds acquire the ability to feed on Sternorrhyncha insects) (Figure S5.3). When the ladybirds feed on Sternorrhyncha diet, these genes from the evolving OGs are more likely to be upregulated based on the ratio, though this pattern does not show in PJAPO. Among the events, these upregulated genes tend to under emergence or expansion at Coccinellidae ancestor. In CMONT, major facilitator superfamily related to digestion is detected as both DEGs and the genes under evolution events, while PBP/GOBP family related to chemosensation is significantly enriched in the intersection of DEGs and evolving genes. Only OG0000406 (lytic polysaccharide mono-oxygenase (LMPO)) have at least one upregulated DEG in all the carnivorous ladybirds feeding on Sternorrhyncha diets (Table S5.4), which is lost in Epilachnini.

In the pollen-feeding ladybird MDISC, only few genes are upregulated in comparison of POLLEN diet versus other diets (APHID, MEALYBUG and MOTHEGG) (Figure S5.4). Only one gene (UGT-like) is in the expanding OGs at the node of MDISC and upregulated when eating POLLEN diet. Most genes with differential expression and evolution events are downregulated and mainly contributed by expansion. The ratio of downregulated DEGs under evolution events (~3.2%) is higher than the ratio in all the genes (~1%). These DEGs under evolution events in MDISC are mainly related to detoxification (including P450), carbohydrate metabolism and chitin-related process (including GH18).

### 5.2.2 Diet-specific differentially expressed genes in the herbivorous ladybird *Henosepilachna vigintioctopunctata*

The *r*^2^ values for the relative gene expression among the replicates of each treatment are all higher than 0.75 (Figure S5.5). The enrichments of GO and Pfam terms reveal that, the downregulated DEGs in HVIGI when the ladybirds feed on sugar water instead of plant leaves, are mainly enriched in the functions related to digestion and detoxification, such as carboxypeptidase, papain family cysteine protease, serpin, glucosidase (including glycosyl hydrolase family 1 (GH1)), lipase, lipocalin, sugar transporter, chitin binding Peritrophin-A domain containing protein (CBPD), ABC transporter (ABC), cytochrome P450 (P450), glutathione S-transferase (GST), UDP-glucoronosyl and UDP-glucosyl transferase (UGT) and carboxylesterase (Figure S5.6). On the contrary, the upregulated DEGs are mainly enriched in motion and development, such as muscle development, microtubule and cuticle structure. Similarly, previous research found that the detoxification-related genes (P450s, UGTs and carboxylesterase), sugar transporters, lipocalins, GH1s and CBPDs were differentially expressed when long-horned beetle *Dectes texanus* or *Anoplophora glabripennis* fed on different plants or artificial diets (Aguirre-Rojas et al., 2021; McKenna et al., 2016), which indicates the importance of these genes to herbivory of HVIGI. According to these enrichment results of comparison between the three groups each other, the glucosidases, serpin, lipocalin and carboxylesterases tend to be downregulated as long as the ladybirds feed on sugar water instead of plant leaves, and those genes are upregulated are always with similar functions. In contrast, the transporters and the genes encoding detoxifying enzymes including ABCs, P450s, GSTs, UGTs, and CBPDs, sugar transporters are always downregulated in the comparison of SUGAR5d versus PLANT and SUGAR5d versus SUGAR1d, which means that these genes are more likely to respond to relatively long-time (five days) lack of plant leaves.

Similar with the carnivorous ladybirds, the volcano plots show that the genes from emergent or expanding OGs in Epilachnini are more likely to be upregulated when the ladybirds are fed plant leaves (Figure S5.7). Among the evolution events, expansion and emergence in Epilachnini mainly contribute to the DEGs, especially those upregulated DEGs. Moreover, the ratio of upregulated DEGs among the genes from emergent or expanding OGs in HVIGI (~6% and ~13% in comparison of SUGAR1d or SUGAR5d versus PLANT, respectively) is higher than the ratio of upregulated DEGs in all genes (~2.5% and ~7%). The genes identified as both diet-specific DEGs and members of emergent or expanded OGs in Epilachnini, mainly include serpin, lipocalin, sugar transporter, CBPD, P450, UGT, GST and carboxylesterase. Among these genes, the large amounts of detoxification-related genes seem to be able to help the herbivorous ladybirds, Epilachnini, to manage the plant secondary compounds, as same as other herbivorous insects (Heidel-Fischer and Vogel, 2015; Li et al., 2007).

In addition, several OGs contain both upregulated DEGs in pollen-fed MDISC and leaf-fed HVIGI, including insulin-like, organic cation transporter protein, chitooligosaccharidolytic beta-N-acetylglucosaminidase, prosaposin, regucalcin-like. In pollen-fed MDISC, 8/17 upregulated DEGs are involved in these OGs, indicating high convergence when the ladybirds feeding on different plant tissues. However, the functions of these OGs are mainly related to nutrient metabolism of oligosaccharides, glycoconjugates, lipid or ascorbic acid (Aizawa et al., 2013; Aumiller et al., 2006; Azuma et al., 1998; Cattaneo et al., 2006; Nagamatsu et al., 1995), instead of degradation of plant cell wall and detoxification, as discussed in our previous research of MDISC (Huang et al., 2022). These OGs are also lack of evolution events at the node of Epilachnini and MDISC. It seems that the involved nutrient metabolism-related DEGs are associated with short-term response to the plant tissues but not key mechanism of plant adaptation.

## 5.3 Summary

We compared the expression patterns of six carnivorous ladybirds and the herbivorous ladybird HVIGI when they feed on different diets and found that the genes from LEGFs (expansion, contraction, emergence and loss) but not other selection pressure at the specific nodes with diet shift are more likely to be DEGs between the different diet treatments. The OGs enriched from the DEGs of changed diets also include several LEGFs at the specific nodes, which are mainly related to the functions connected with feeding habits, such as chemosensation, digestion, detoxification and immunity. These results indicate that the evolution of the OGs related to chemosensation, digestion, detoxification and immunity may be a result of the adaptation to the new diets in the ladybirds.

However, no obvious correlation between evolution events and pollen-specific DEGs in the omnivorous ladybird MDISC is found. It is possible that the ability of pollen feeding of the omnivorous ladybirds depends on their symbiotic bacteria, as proposed in our previous research (Huang et al., 2022). In consideration of only one species in the omnivorous ladybirds with the genome and no obvious finding in evolution events of MDISC, we then focus on the aphidophagy rather than the pollinivory in MDISC in the gene family analyses and leave the problem of pollinivory for further research with more genomes and experimental evidence of the omnivorous ladybirds.

Table S5.1 Transcriptome comparisons of different diet treatments in the ladybirds. The upper-case diet treatments are used for further exploration of connection to evolution events in Section 5-10

| Species | Optimal food | Stage | Diet treatments | Reference | Usage |
| --- | --- | --- | --- | --- | --- |
| CMONT | Mealybugs | fourth instar larva | APHID, MEALYBUG, MOTHEGG | Li et al. (2021a); Li et al. (2016a) | Section 5.2.1, Section 7-10, main text |
|  |  |  | pollen, brine shrimp egg, bee pupa, pork meat, pork liver, yellow meal worm, chicken egg, maggot, earthworm, rice moth egg, black soldier fly larva, silkworm pupa, superworm |  | Section 5.2.1 |
|  |  | female adult | APHID vs MEALYBUG | Li et al. (2016a) | Section 5.2.1, Section 6, Section 7-10, main text |
| PJAPO | Aphids | fourth instar larva | MEALYBUG vs APHID | Chen et al. (2020) | Section 5.2.1, Section 7-10, main text |
|  |  |  | MOTHEGG vs APHID | Li et al. (2024) | Section 5.2.1, Section 7-10, main text |
|  |  | female adult | MEALYBUG vs APHID | This study | Section 5.2.1, Section 7-10, main text |
| MDISC | Aphids | fourth instar larva | MEALYBUG, APHID, MOTHEGG, POLLEN | Huang et al. (2022) | Section 5.2.1, Section 7-10, main text |
| CSEPT | Aphids | fourth instar larva | MEALYBUG vs APHID | Chen et al. (2020) | Section 5.2.1, Section 7-10, main text |
|  |  | adult | ARTIFICIAL_DIET vs APHID *Aphis craccivora* | Cheng et al. (2020) | Section 5.2.1, Section 6 |
| HAXYR | Aphids | fourth instar larva | MEALYBUG vs APHID | Chen et al. (2020) | Section 5.2.1, Section 7-10, main text |
|  |  |  | high-protein food, high-carbohydrate food, equivalent food | Sun et al. (2022) | Section 5.2.1 |
|  |  | female adult | artificial diet vs *Acyrthosiphon pisum* in long-day photoperiod condition | Gao et al. (2022) | Section 5.2.1 |
|  |  |  | artificial diet vs *Acyrthosiphon pisum* in short-day photoperiod condition | Gao et al. (2022) | Section 5.2.1 |
|  |  |  | artificial diet vs aphid | Liang et al. (2019) | Section 5.2.1 |
| Tran_LSAUC | Aphids | fourth instar larva | MEALYBUG vs APHID | This study | Section 5.2.1 |
| NPUMI | Scale insects | fourth instar larva | *Icerya aegyptiaca* vs starvation | Tang et al. (2022b) | Section 5.2.1 |
|  |  | female adult | *Icerya aegyptiaca* vs starvation | Tang et al. (2022b) | Section 5.2.1 |
| HVIGI | Plant leaves | male adult | PLANT, SUGAR1d, SUGAR5d | This study | Section 5.2.2, Section 7-10 and main text (only SUGAR5d vs PLANT) |

Table S5.2 Orthogroup (OG) enrichment results of differentially expressed genes (DEGs) in six carnivorous ladybird species under different diet treatments. Only the OGs with significant enrichment (adjusted *p*<0.05) observed in at least two comparisons are shown. The results indicate the ratio of significantly enriched comparisons/enriched comparisions/all comparisons in specific OGs.

| Orthogroup | CMONT | PJAPO | HAXYR | CSEPT | MDISC | Tran_LSAUC | Events | Annotation |
| --- | --- | --- | --- | --- | --- | --- | --- | --- |
| OG0000025 | 2/3/3 | 1/2/2 | 0/1/1 | 1/1/1 | 2/4/6 | 0/1/1 | contraction in MDISC | insect cuticle protein |
| OG0000113 | 0/0/3 | 1/2/2 | 0/0/1 | 0/0/1 | 5/6/6 | 0/0/1 | expansion in MDISC, expansion in Epilachnini | UDP-glucosyltransferase |
| OG0000120 | 0/0/3 | 1/1/2 | 0/0/1 | 0/0/1 | 5/5/6 | 0/0/1 | expansion in Coccinellidae, expansion in Epilachnini | PBP/GOBP family |
| OG0000044 | 1/3/3 | 1/1/2 | 0/1/1 | 0/0/1 | 3/4/6 | 0/0/1 | expansion in MDISC | cytochrome P450 |
| OG0000111 | 0/0/3 | 0/0/2 | 0/0/1 | 0/0/1 | 4/5/6 | 0/0/1 | - | facilitated trehalose transporter Tret1-like |
| OG0000134 | 0/0/3 | 0/0/2 | 0/0/1 | 0/0/1 | 4/5/6 | 0/0/1 | expansion in MDISC, expansion in Epilachnini | fatty acid synthase-like |
| OG0000041 | 0/3/3 | 0/0/2 | 0/1/1 | 0/0/1 | 2/5/6 | 1/1/1 | - | ecdysteroid kinase |
| OG0000047 | 0/3/3 | 1/1/2 | 0/1/1 | 0/1/1 | 2/6/6 | 0/1/1 | - | UDP-glucosyltransferase |
| OG0000184 | 0/0/3 | 0/0/2 | 0/0/1 | 0/0/1 | 3/5/6 | 0/0/1 | expansion in MDISC | glycosyl hydrolases family 18 |
| OG0000439 | 0/0/3 | 0/0/2 | 0/0/1 | 0/0/1 | 3/3/6 | 0/0/1 | expansion in MDISC | UDP-glycosyltransferase |
| OG0000458 | 0/0/3 | 0/0/2 | 0/0/1 | 0/0/1 | 3/3/6 | 0/0/1 | expansion in MDISC | coleoptericin |
| OG0000042 | 0/1/3 | 0/1/2 | 1/1/1 | 1/1/1 | 0/3/6 | 0/1/1 | - | insect pheromone-binding family |
| OG0000065 | 1/3/3 | 0/2/2 | 1/1/1 | 0/1/1 | 0/0/6 | 0/1/1 | contraction in Coccinellini | procathepsin L-like |
| OG0000081 | 1/3/3 | 1/1/2 | 0/1/1 | 0/0/1 | 0/0/6 | 0/1/1 | - | cytochrome P450 |
| OG0000106 | 0/3/3 | 1/1/2 | 0/0/1 | 0/0/1 | 1/3/6 | 0/0/1 | expansion in MDISC | fatty acyl-CoA reductase wat-like |
| OG0000212 | 0/0/3 | 1/2/2 | 0/0/1 | 0/0/1 | 0/0/6 | 1/1/1 | - | P-granule-associated novel protein 1-like |
| OG0001915 | 2/2/3 | 0/0/2 | 0/0/1 | 0/0/1 | 0/0/6 | 0/0/1 | - | uncharacterized protein |

Table S5.3 Orthogroup (OG) enrichment results of differentially expressed genes (DEGs) in all the carnivorous ladybird species under different diet treatment comparisons we collected. Only the OGs with significant enrichment (adjusted *p*<0.05) observed in at least two species are shown. The results indicate the ratio of significantly enriched comparisons/all comparisons in specific OGs.

| Orthogroup | NPUMI | CMONT | PJAPO | HAXYR | CSEPT | MDISC | Tran_LSAUC | Events | Annotation |
| --- | --- | --- | --- | --- | --- | --- | --- | --- | --- |
| OG0000025 | 0/2 | 35/121 | 1/3 | 0/7 | 1/3 | 2/6 | 0/1 | contraction in MDISC | insect cuticle protein |
| OG0000065 | 1/2 | 32/121 | 1/3 | 3/7 | 0/3 | 0/6 | 0/1 | contraction in Coccinellini | procathepsin L-like |
| OG0000217 | 0/2 | 33/121 | 1/3 | 0/7 | 0/3 | 0/6 | 0/1 | - | uncharacterized protein |
| OG0000247 | 0/2 | 26/121 | 1/3 | 0/7 | 0/3 | 0/6 | 0/1 | expansion in Coccinellidae, contraction in Coccinellini | UDP-glycosyltransferase |
| OG0000041 | 0/2 | 18/121 | 1/3 | 0/7 | 0/3 | 2/6 | 1/1 | - | ecdysteroid kinase |
| OG0000047 | 0/2 | 17/121 | 2/3 | 0/7 | 0/3 | 2/6 | 0/1 | - | UDP-glucosyltransferase |
| OG0000068 | 0/2 | 17/121 | 0/3 | 0/7 | 0/3 | 1/6 | 0/1 | expansion in Coccinellidae, expansion in Epilachnini | major facilitator superfamily |
| OG0000044 | 0/2 | 11/121 | 1/3 | 1/7 | 0/3 | 3/6 | 0/1 | expansion in MDISC | cytochrome P450 |
| OG0000081 | 0/2 | 11/121 | 1/3 | 2/7 | 0/3 | 0/6 | 0/1 | - | cytochrome P450 |
| OG0000027 | 1/2 | 6/121 | 0/3 | 4/7 | 0/3 | 0/6 | 0/1 | - | dynein axonemal heavy chain 1-like |
| OG0000042 | 0/2 | 9/121 | 0/3 | 1/7 | 1/3 | 0/6 | 0/1 | - | insect pheromone-binding family |
| OG0000106 | 0/2 | 7/121 | 1/3 | 0/7 | 0/3 | 1/6 | 0/1 | expansion in MDISC | fatty acyl-CoA reductase wat-like |
| OG0000120 | 0/2 | 0/121 | 2/3 | 0/7 | 0/3 | 5/6 | 0/1 | expansion in Coccinellidae, expansion in Epilachnini | PBP/GOBP family |
| OG0000113 | 0/2 | 0/121 | 1/3 | 0/7 | 0/3 | 5/6 | 0/1 | expansion in MDISC, expansion in Epilachnini | UDP-glucosyltransferase |
| OG0000111 | 0/2 | 0/121 | 0/3 | 1/7 | 0/3 | 4/6 | 0/1 | - | facilitated trehalose transporter Tret1-like |
| OG0000212 | 0/2 | 0/121 | 2/3 | 0/7 | 0/3 | 0/6 | 1/1 | - | P-granule-associated novel protein 1-like |

Table S5.4 Ortholog groups (OGs) with at least one upregulated DEG in each species under specific diet treatments in the ladybirds. The numbers of each species represent the counts of upregulated DEGs. Comparisons of APHID and MEALYBUG include APHID vs MEALYBUG (or MEALYBUG vs APHID) in larvae of CMONT, PJAPO, HAXYR, CSEPT, MDISC, with APHID vs MEALYBUG in larvae of Tran_LSAUC and female adults of CMONT and PJAPO as supplements. Specifically, for APHID-related OGs, only the OGs containing at least one upregulated DEG in larvae of Coccinellini species (PJAPO, HAXYR, CSEPT and MDISC) are shown in the table. Comparisons of Sternorrhyncha include APHID+MEALYBUG vs MOTHEGG in larvae of CMONT, APHID vs MOTHEGG in larvae of PJAPO and APHID+MEALYBUG vs MOTHEGG+POLLEN in larvae of MDISC, with ARTIFICIAL_DIET vs APHID in the female and male adults of CSEPT as supplements. Comparisons of plant tissues include POLLEN vs APHID+MEALYBUG+MOTHEGG in MDISC and PLANT vs SUGAR5d in HVIGI.

| Orthogroup | Diet | CMONT | PJAPO | HAXYR | CSEPT | MDISC | HVIGI | Others | Annotation |
| --- | --- | --- | --- | --- | --- | --- | --- | --- | --- |
| OG0000044 | APHID | 5 | 2 | 1 | 2 | 1 | - | 7 in female CMONT; 2 in female PJAPO | cytochrome P450 |
| OG0000068 | APHID | 3 | 1 | 2 | 1 | 1 | - | 1 in Tran_LSAUC | major facilitator superfamily |
| OG0000112 | APHID | 0 | 1 | 2 | 3 | 1 | - | 1 in Tran_LSAUC; 2 in female CMONT | juvenile hormone acid O-methyltransferase-like |
| OG0000136 | APHID | 0 | 4 | 1 | 3 | 1 | - | 2 in Tran_LSAUC | glucose dehydrogenase [FAD, quinone]-like |
| OG0001844 | APHID | 1 | 5 | 1 | 1 | 3 | - | 1 in female CMONT | multifunctional protein ADE2 |
| OG0003569 | APHID | 1 | 2 | 1 | 1 | 1 | - | 1 in female CMONT | trifunctional purine biosynthetic protein adenosine-3 |
| OG0000406 | Sternor-rhyncha | 4 | 1 | - | - | 2 | - | 1 in ARTIFICIAL_DIET vs APHID of female CSEPT | lytic polysaccharide mono-oxygenase |
| OG0000005 | plant tissues | - | - | - | - | 1 | 2 | - | uncharacterized protein |
| OG0000521 | plant tissues | - | - | - | - | 1 | 1 | - | insulin-like |
| OG0001678 | plant tissues | - | - | - | - | 1 | 1 | - | unknown protein |
| OG0003731 | plant tissues | - | - | - | - | 1 | 1 | - | organic cation transporter protein |
| OG0004113 | plant tissues | - | - | - | - | 1 | 1 | - | uncharacterized protein |
| OG0004610 | plant tissues | - | - | - | - | 1 | 1 | - | chitooligosaccharidolytic beta-N-acetylglucosaminidase |
| OG0005318 | plant tissues | - | - | - | - | 1 | 1 | - | prosaposin |
| OG0006750 | plant tissues | - | - | - | - | 1 | 1 | - | regucalcin-like |


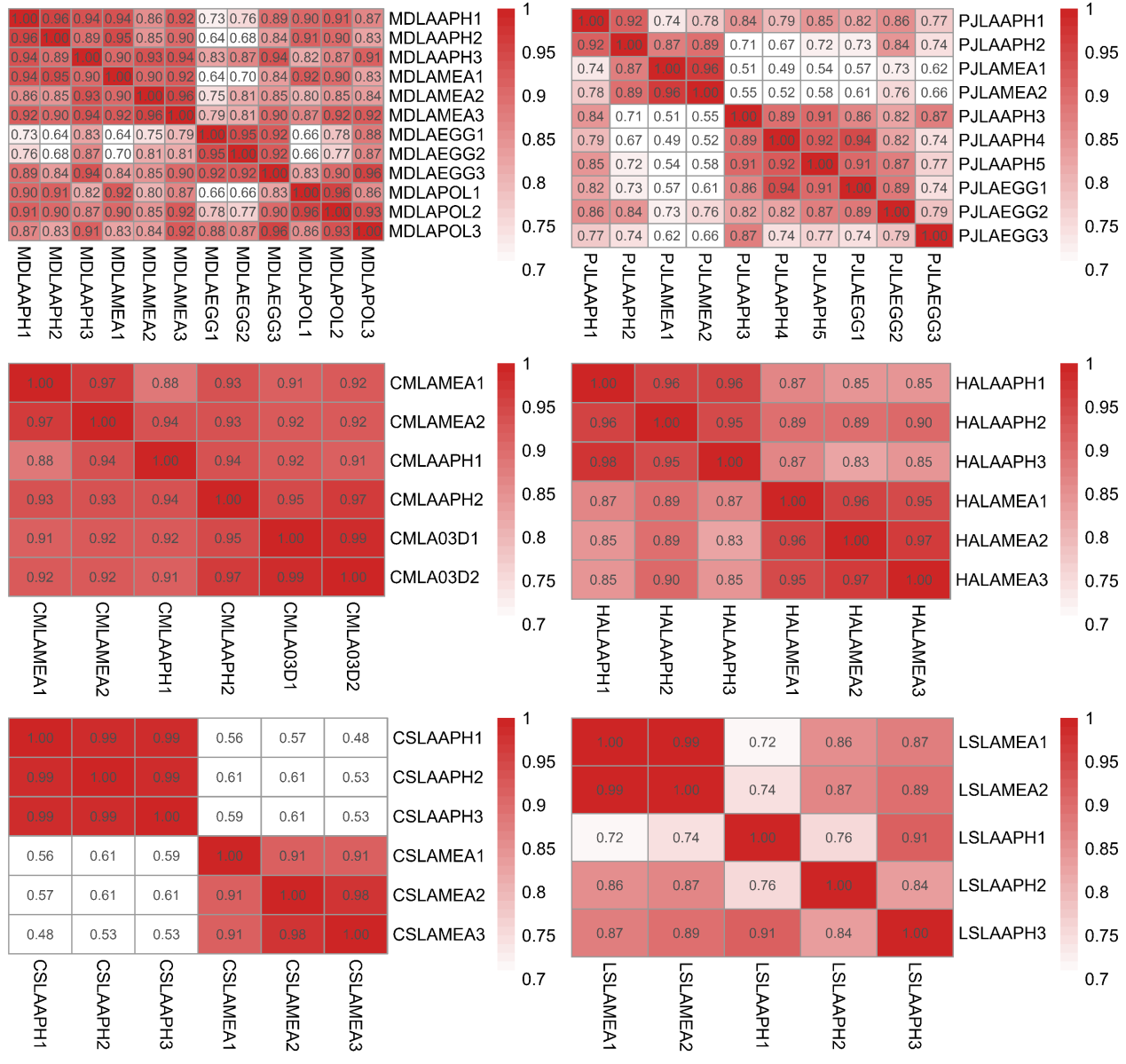


Figure S5.1 *r*^2^ of each diet treatment comparison in carnivorous ladybirds. MD: MDISC, PJ: PJAPO, CM: CMONT, HA: HAXYR, CS: CSEPT, LS: Tran_LSAUC, LA: fourth instar larvae, APH: APHID, MEA: MEALYBUG, EGG or 03D: MOTHEGG, POL: POLLEN. A high-quality figure can be downloaded from https://github.com/huangyh45/ladybird-genomes-supplementary-figures.


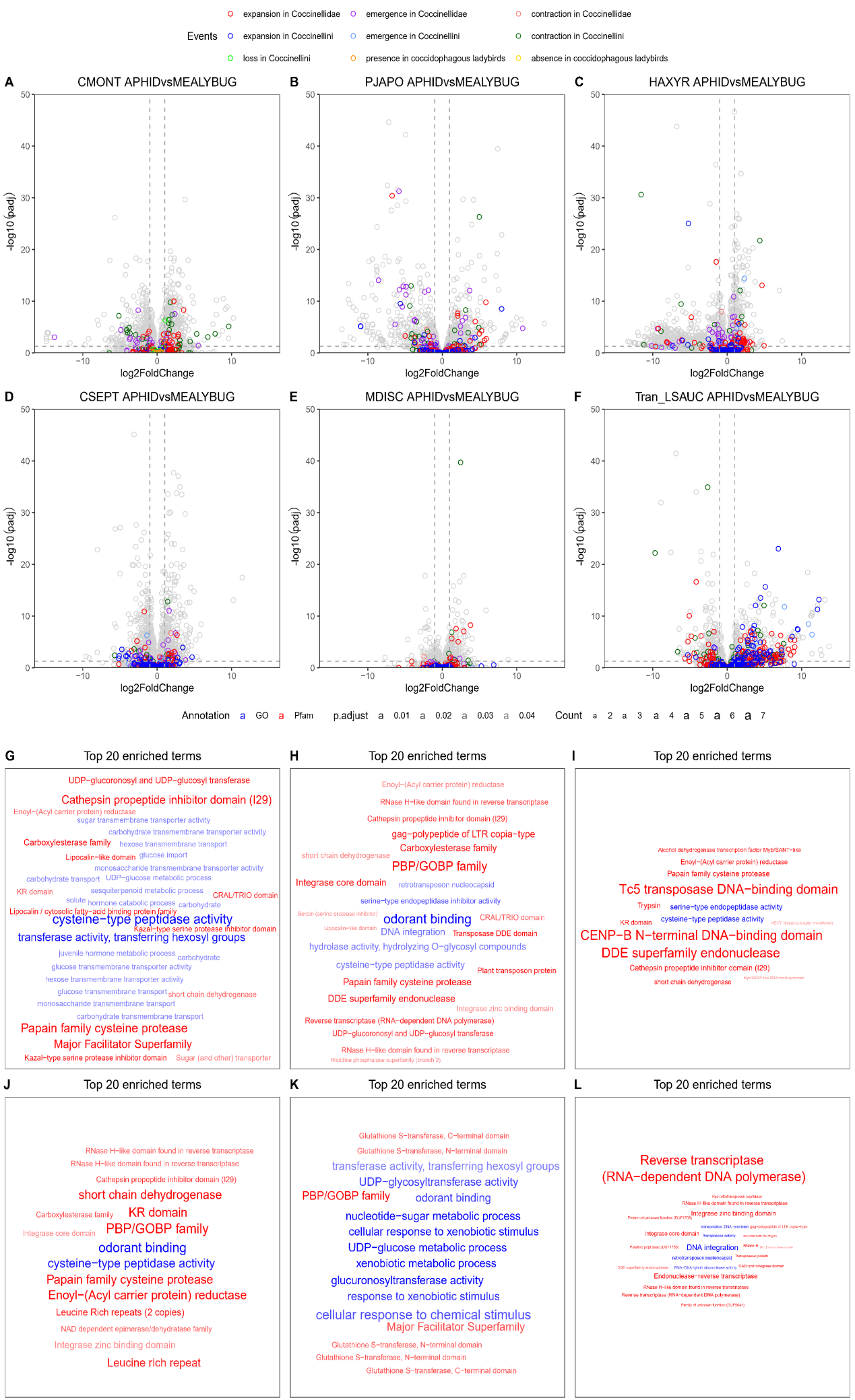


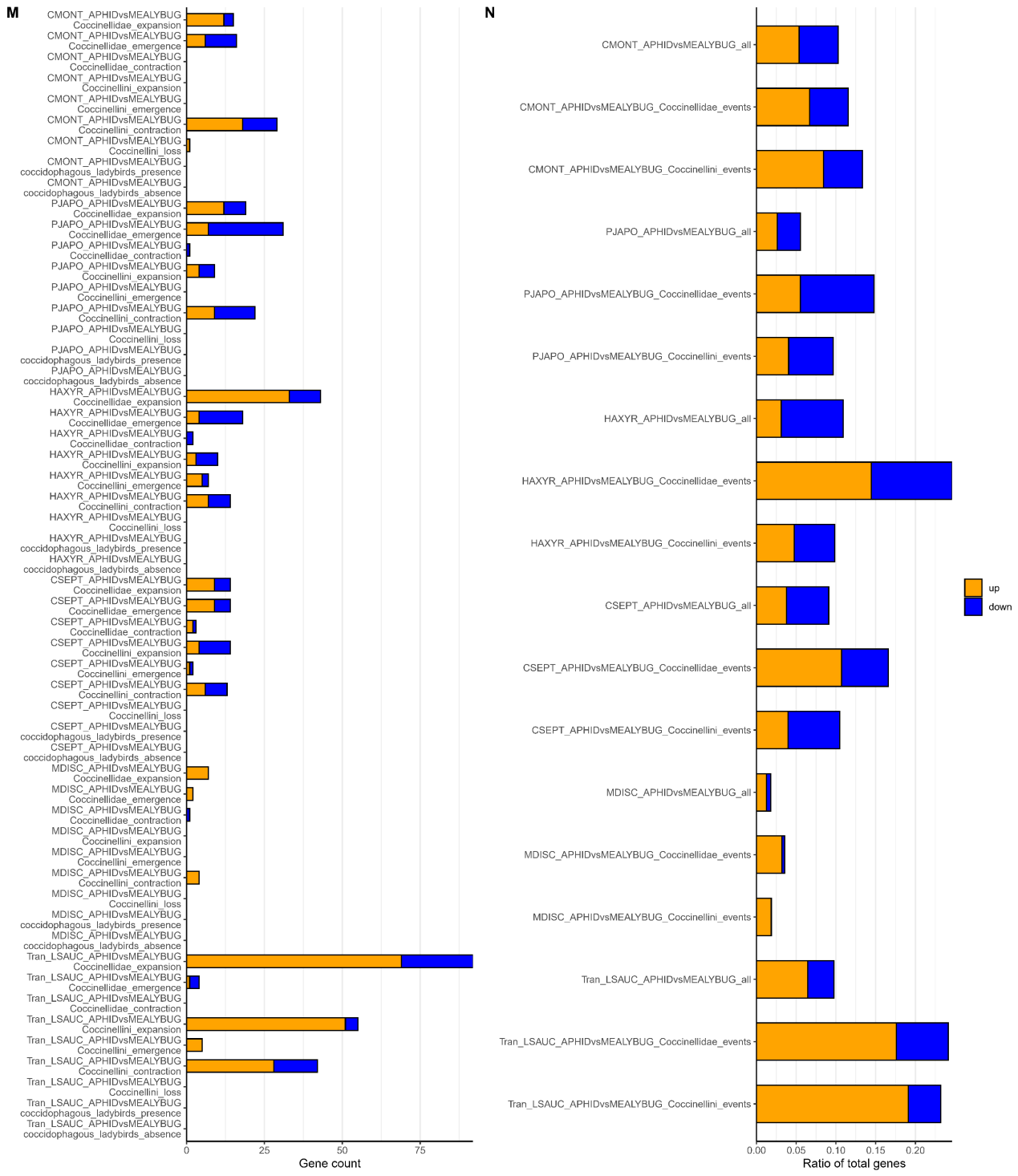


Figure S5.2 Relation between evolution events and differentially expressed genes (DEGs) in aphid and mealybug diet-specific transcriptome comparisons in the ladybirds. The enrichments are conducted using the DEGs under evolution events. A high-quality figure can be downloaded from https://github.com/huangyh45/ladybird-genomes-supplementary-figures.


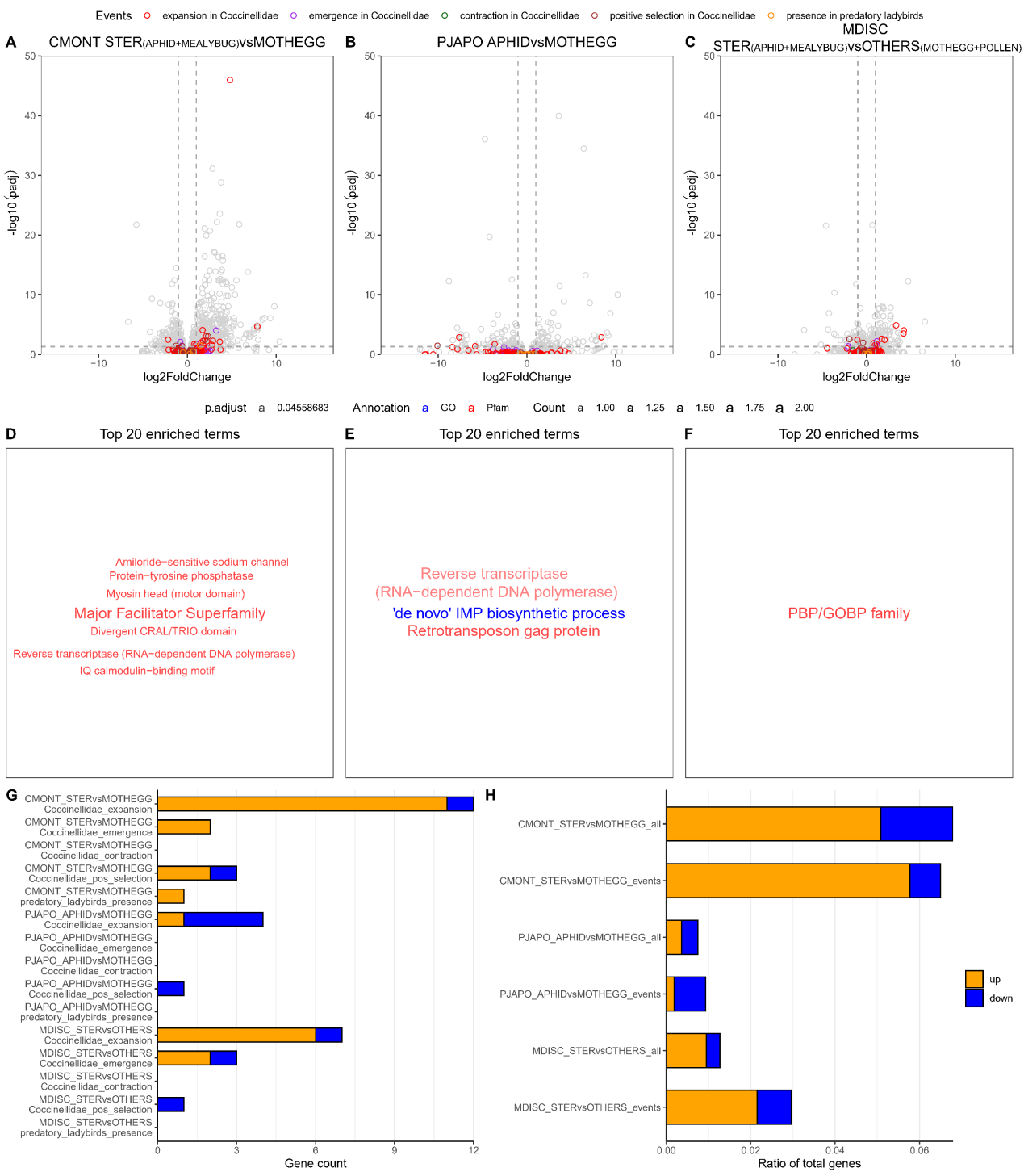


Figure S5.3 Relation between evolution events and differentially expressed genes (DEGs) in Sternorrhyncha diet-specific transcriptome comparisons in the ladybirds. The enrichments are conducted using the DEGs under evolution events. A high-quality figure can be downloaded from https://github.com/huangyh45/ladybird-genomes-supplementary-figures.


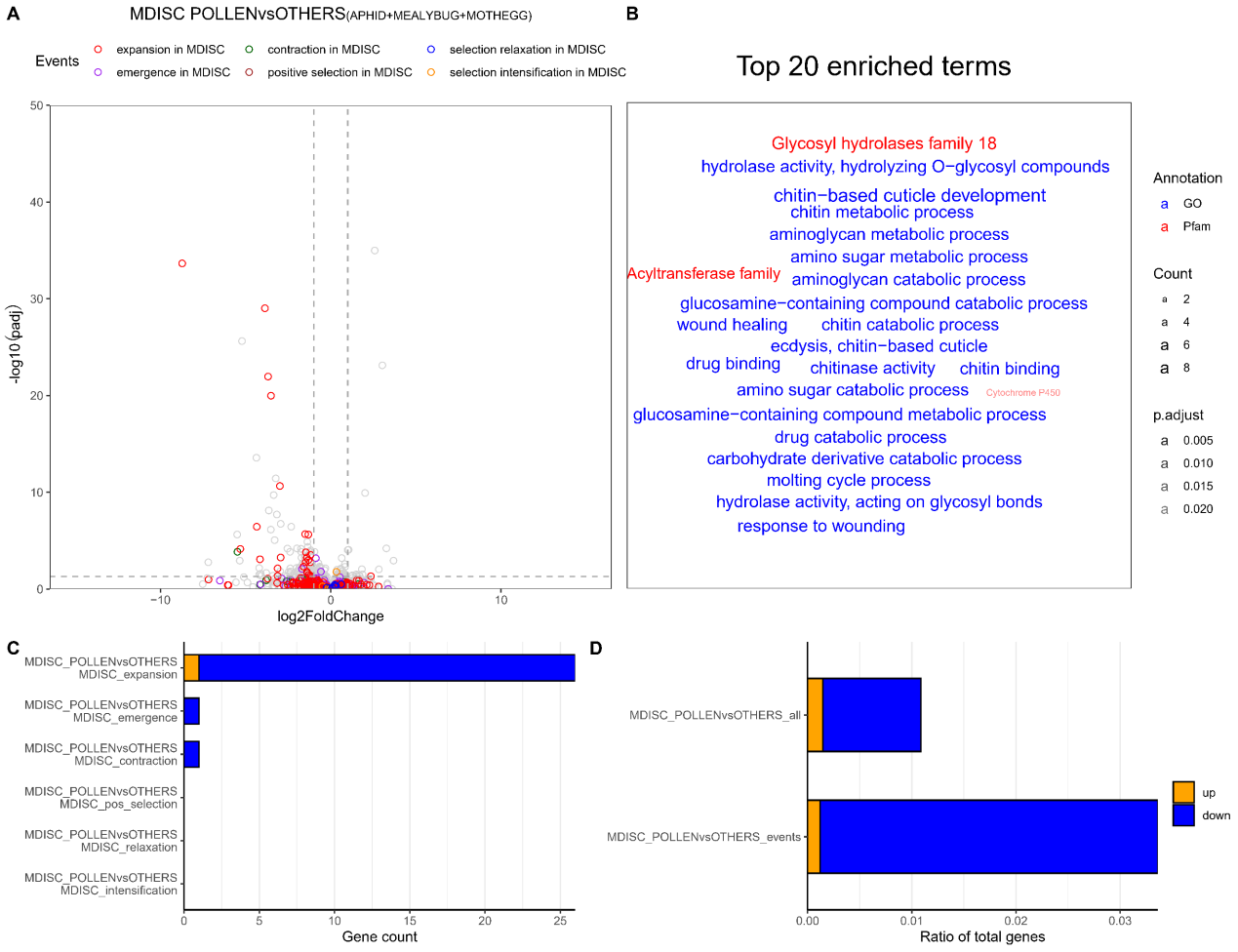


Figure S5.4 Relation between evolution events and differentially expressed genes (DEGs) in pollen diet-specific transcriptome comparisons in MDISC. The enrichments are conducted using the DEGs under evolution events. A high-quality figure can be downloaded from https://github.com/huangyh45/ladybird-genomes-supplementary-figures.


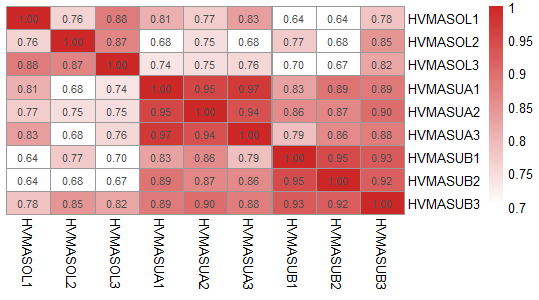


Figure S5.5 *r*^2^ of diet treatment comparison in HVIGI. HV: HVIGI, MA: male adult, SOL: feeding on leaves of *Solanum nigrum* (PLANT treatment), SUA: feeding on sugar water for one day (SUGAR1d treatment), SUB: feeding on sugar water for five days (SUGAR5d treatment).


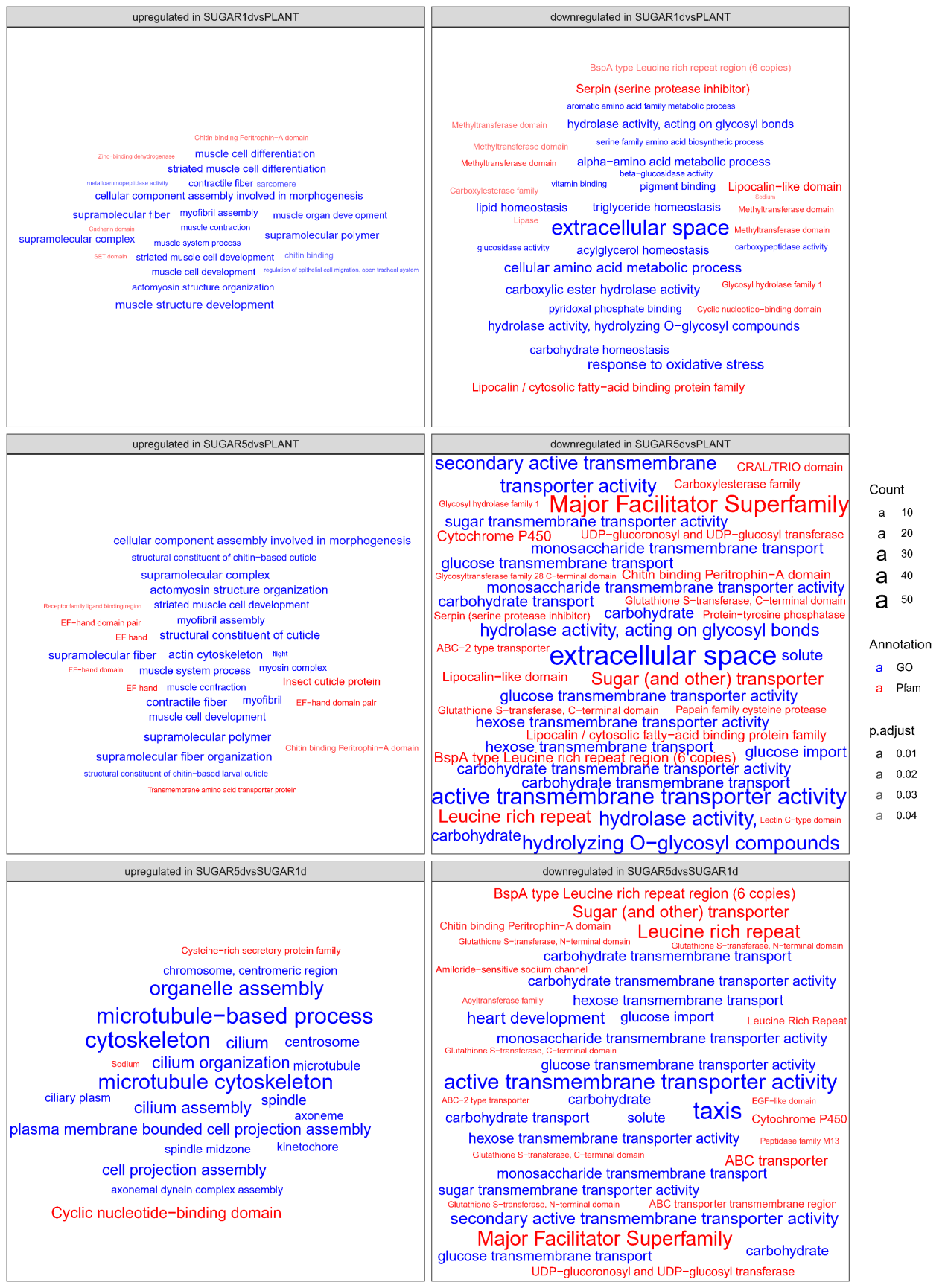


Figure S5.6 Enrichment of gene ontology (GO) or Pfam annotation of differentially expressed genes (DEGs) between diet treatments in HVIGI. Only the top 20 GO or Pfam annotations in the enrichment results are shown. A high-quality figure can be downloaded from https://github.com/huangyh45/ladybird-genomes-supplementary-figures.


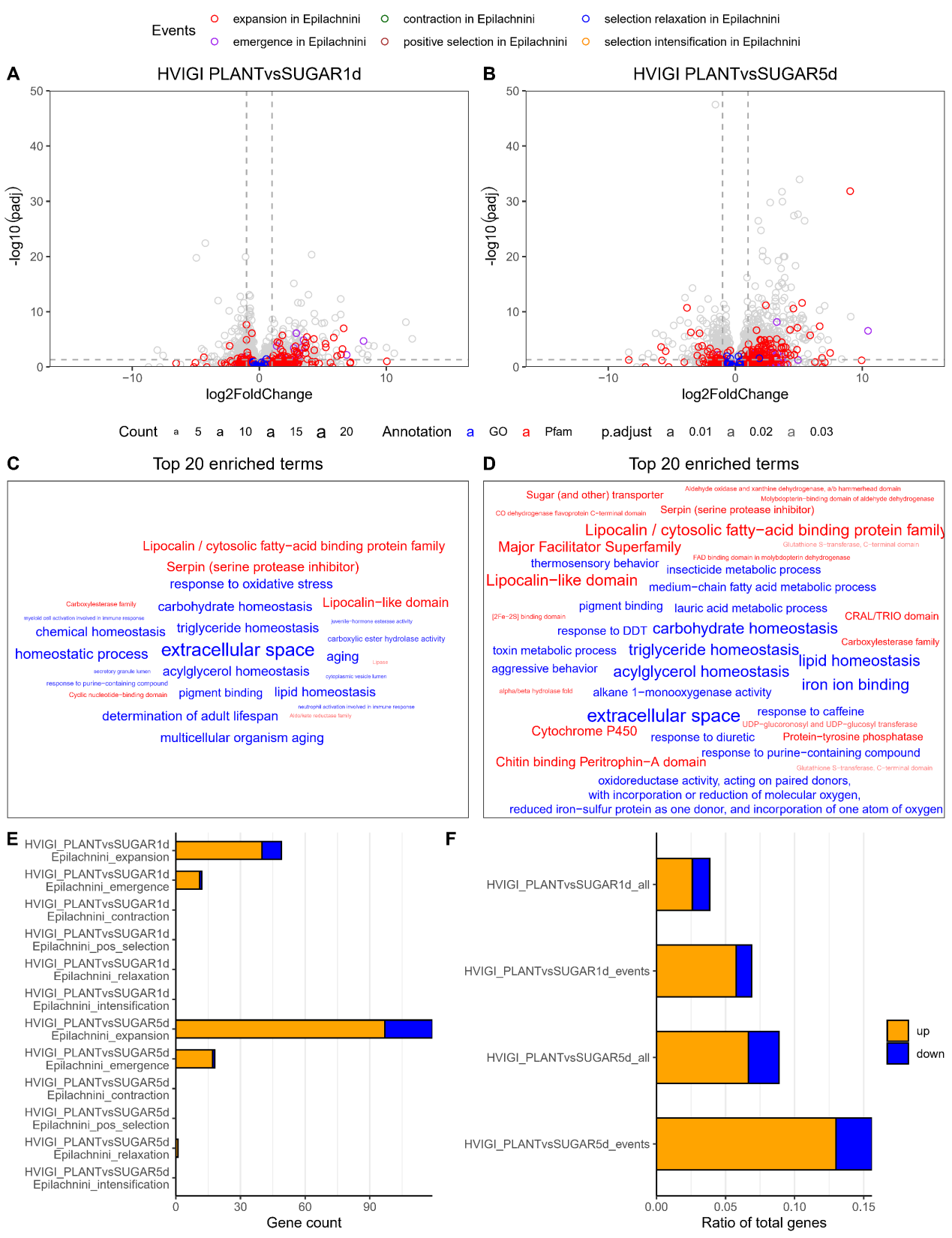


Figure S5.7 Relation between evolution events and differentially expressed genes (DEGs) in plant diet-specific transcriptome comparisons in Epilachnini. The enrichments are conducted using the DEGs under evolution events. A high-quality figure can be downloaded from https://github.com/huangyh45/ladybird-genomes-supplementary-figures.

# 6 Tissue-specific transcriptome comparison

## 6.1 Materials and methods

Different tissue samples were mainly obtained from two ladybird species, CMONT and CSEPT. The population of CMONT was reared on the mealybug *Planococcus citri*, and the population of CSEPT was reared on the aphid *Megoura japonica*, as described in our previous research (Chen et al., 2020; Li et al., 2021a). Antenna, gut, leg, head, thorax and abdomen were separated from the healthy female or male adults of the ladybirds after eclosion within a week. The adults were fed by enough prey before dissection. For CMONT, each antenna sample was acquired from 350 individuals, along with each leg or head sample from 100 individuals, each gut sample from 5 individuals, each thorax or abdomen sample from 2 individuals. For CSEPT with larger body size, 120 individuals generated one antenna sample, with 50 individuals for each leg or head sample, 3 individuals for each gut sample, 2 individuals for each thorax or abdomen sample. Female and male groups were set for each tissue, and biological replicates were set for each group.

Sample storage, RNA extraction, sequencing and transcriptome analyses were the same as diet-specific transcriptomes mentioned in Section 5.1. Specifically, to explore the highly expressed genes in each tissue, all samples of this tissue were compared with all other samples to detect DEGs, with the sex as covariate. Only the genes with highest mean FPKM values among all the six tissues, and identified as significantly upregulated DEGs in this tissue are considered as tissue-specific highly expressed genes. These tissue-specific highly expressed genes are enriched based on their GO or Pfam annotation in each species, respectively. Further, we connected the tissue-specific highly expressed genes with diet-specific transcriptome comparisons of the adult CMONT (APHID versus MEALYBUG) or CSEPT (APHID vs ARTIFICIAL_DIET) (Figure S5.1). To avoid missing the genes specially expressed in two or more tissues, we also performed cluster analysis using the FPKM matrix through ClusterGVis R package (https://github.com/junjunlab/ClusterGVis). Optimal number of clusters was chosen with help though visualization by getClusters function and then the gene expression was clustered by k-means method.

In addition, the tissue-specific transcriptomes of CIMPU were also sequences and analyzed. Only gut-specific transcriptome comparison was conducted, due to limited individuals. The gut and other body tissue were separated from the same wild caught individual. Each sample was generated from one individual and two biological replicates of gut or other body tissue were prepared. Sample storage, RNA extraction, sequencing and DEG detection were the same as diet-specific transcriptomes mentioned in Section 5.1.

## 6.2 Results

The *r*^2^ values for the relative gene expression among the replicates of each group are mostly larger than 0.8, except the samples of thorax in male adults of CSEPT (Figure S6.1), revealing relatively close expression patterns within the same groups. The expression in the same tissues, especially antenna, leg, gut and head (*r*^2^ > 0.8), is always similar between female and male adults, but those of thorax and abdomen are more different (*r*^2^ = 0.5-0.9). Between the tissues, the expression show relatively difference (mostly *r*^2^ < 0.5), but the expression in leg may be similar to antenna, head or thorax, and thorax may have similar patterns with abdomen. Gut in CIMPU also shows large difference with other body tissue (*r*^2^ = 0.11-0.34), indicating potential gut-specific expression patterns in herbivorous ladybirds.

The enrichments of tissue-specific highly expressed DEGs in CMONT, CSEPT and CIMPU (only gut-specific) reveal distribution of functional genes in different tissues (Figure S6.2). In the antenna, large amounts of chemosensory genes are enriched, including 7tm chemosensory receptor, 7tm odorant receptor, PBP/GOBP family, ion transport protein, ligand-gated ion channel. In the gut of the ladybirds, mainly genes related to digestion (sugar transporter, peptidase, papain family cysteine protease, major facilitator super family) and detoxification (ABC transporter (ABC), UDP-glycosyl and UDP-glucosyl transferase (UGT), Aldo/keto reductase family (AKR)) are highly expressed. Specifically, in the herbivorous ladybird CIMPU, glycosyl hydrolase family 1 (GH1), lipocalin, Peritrophin-A domain containing protein (CBPD), glutathione S-transferase (GST), carboxylesterase related to digestion or detoxification are gut-specifically expressed. These more digestion and detoxification-related genes in the gut of herbivorous ladybirds indicate potentially more need of complicated system of digestion and detoxification to manage the plant leaves. The longer gut length and more complicated gut structure of herbivorous ladybirds than carnivorous ladybirds also support this assumption (Hodek, 1973). But tissue data of other herbivorous ladybirds may be required to avoid species-specific expression patterns in CIMPU. In the leg, the main functions of the highly expressed genes are associated with motor ability and muscle development, along with some chemosensory genes, such as receptor family ligand binding region, ligand ion channel and solute-binding protein. Head-specifically expressed genes are mainly related to neuro (neurotransmitter-gated ion-channel and synapse) and chemosensation (PBP/GOBP family, receptor family ligand binding region, ion channel, 7 transmembrane sweet-taste receptor, solute-binding protein). In the thorax, the highly expressed genes are mainly energy-related, including mitochondrial process, ATP metabolism, glycolytic process, which seems to be related to energy demand of flight (Mitterboeck et al., 2017; Shen et al., 2010; Shen et al., 2009). The highly expressed genes in the abdomen of the ladybirds mainly include those related to cell division (meiosis, mitosis, chromosome segregation, spindle, microtubule, DNA replication, organelle fission) and reproduction (meiosis, sperm motility, male sterility protein). These different expression patterns in different tissues in the ladybirds may reveal their corresponding roles, such as chemosensation of antenna, food management of gut, motor ability and chemosensation of leg, neuro structure and chemosensation of head, flight ability of thorax, reproduction of abdomen.

When connecting with diet-specific transcriptome comparisons, we found that the diet-specific DEGs of APHID vs MEALYBUG treatment in CMONT include relative amounts of highly expressed genes in gut, antenna or abdomen and a few genes highly expressed in leg or thorax (Figure S6.3). Among these genes, mainly gut-specific highly expressed genes go through expansion in Coccinellidae or contraction in Coccinellini. Some aphid diet-specific upregulated DEGs are highly expressed in antenna and under expansion in Coccinellidae, while some mealybug diet-specific upregulated DEGs are highly expressed in abdomen and emergent in Coccinellidae. In APHID vs ARTIFICIAL_DIET comparison of CSEPT, DEGs also include highly-expressed DEGs in antenna, gut or abdomen, and some in head, leg or thorax (Figure S6.4). Among these genes, antenna-specific highly expressed genes are upregulated in APHID diet treatment in female adults and under expansion in Coccinellini, while antenna-specific and APHID diet-specific DEGs in male adults undergo emergence in Coccinellini, expansion or emergence in Coccinellidae. Furthermore, several gut-specific highly expressed genes are upregulated when both the female and male ladybirds not feed on aphid, which is under expansion in Coccinellidae.

Using the k-means cluster method by ClusterGVis, we also got similar expression patterns in different tissues. Elbow method show that 7 or 8 clusters are the best in CMONT or CSEPT (Figure S6.5). We set the number of clusters as 8 in both species and obtained relatively similar expression patterns in CMONT and CSEPT (Table S6.1, Figure S6.6, S6.7). The clusters include Cluster Antenna (highly expressed in antenna of both sexes, Cluster 6 in CMONT and Cluster 8 in CSEPT), Cluster Head (highly expressed in head of both sexes, Cluster 7 in CMONT and Cluster 7 in CSEPT), Cluster Leg (highly expressed in leg of both sexes, Cluster 8 in CMONT and Cluster 5 in CSEPT), Cluster Gut (highly expressed in gut of both sexes, Cluster 1 in CMONT and Cluster 2 in CSEPT), Cluster Male abdomen (highly expressed in abdomen of male adults, Cluster 3 in CMONT and Cluster 1 in CSEPT), Cluster Female abdomen1 (highly expressed in thorax or abdomen of female adults, Cluster 4 in CMONT and Cluster 3 in CSEPT), Cluster Female abdomen2 (highly expressed in thorax or abdomen of female adults as well as antenna and head of both sexes, Cluster 5 in CMONT and Cluster 4 in CSEPT) and Unclassified (unclear patterns, Cluster 2 in CMONT and Cluster 6 in CSEPT).

## 6.3 Summary

We explored the expression patterns of different tissues (antenna, gut, leg, head, thorax and abdomen) in two carnivorous ladybird CMONT and CSEPT along with gut-specific expression pattern in the herbivorous ladybird CIMPU. It found that different tissues have obviously different expression patterns and the patterns in specific tissue are related to functional role of the tissue. Chemosensory genes are highly expressed in antenna, head and leg, while genes-related to digestion and detoxification are highly expressed in the gut. The diet-specific DEGs also include relatively amounts of genes highly expressed in gut and antenna, some of which go through evolution events at the nodes with diet shift. We thus infer that these genes may be tightly associated with feeding habits in the ladybirds and deeply explore them in the downstream gene family analyses combined with the results of diet-specific and tissue-specific transcriptome comparisons.

Table S6.1 Cluster of expression patterns of genes in different tissues of CMONT and CSEPT.

|  | CMONT | CSEPT |
| --- | --- | --- |
| Cluster Antenna | Cluster 7 | Cluster 8 |
| Cluster Head | Cluster 6 | Cluster 7 |
| Cluster Leg | Cluster 8 | Cluster 5 |
| Cluster Gut | Cluster 1 | Cluster 2 |
| Cluster Female abdomen1 | Cluster 4 | Cluster 3 |
| Cluster Female abdomen2 | Cluster 5 | Cluster 4 |
| Cluster Male abdomen | Cluster 3 | Cluster 1 |
| Unclassified | Cluster 2 | Cluster 6 |


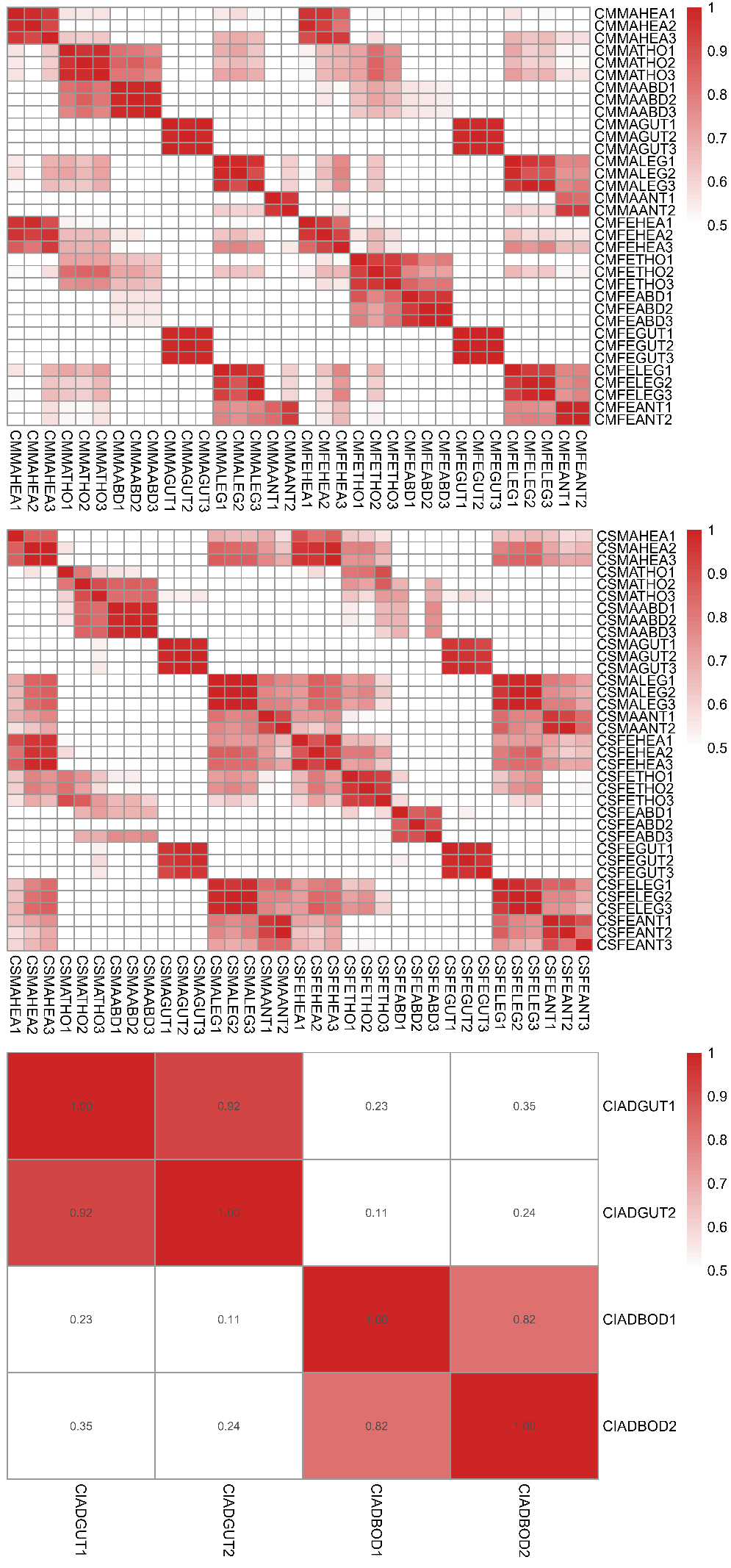


Figure S6.1 *r*^2^ of each tissue group in carnivorous ladybirds. CM: CMONT, CS: CSEPT, CI: CIMPU, FE: female adult, MA: male adult, AD: adult without sex information, ANT: antenna, GUT: gut, LEG: leg, HEA: head, THO: thorax, ABD: abdomen, BOD: body without gut. A high-quality figure can be downloaded from https://github.com/huangyh45/ladybird-genomes-supplementary-figures.


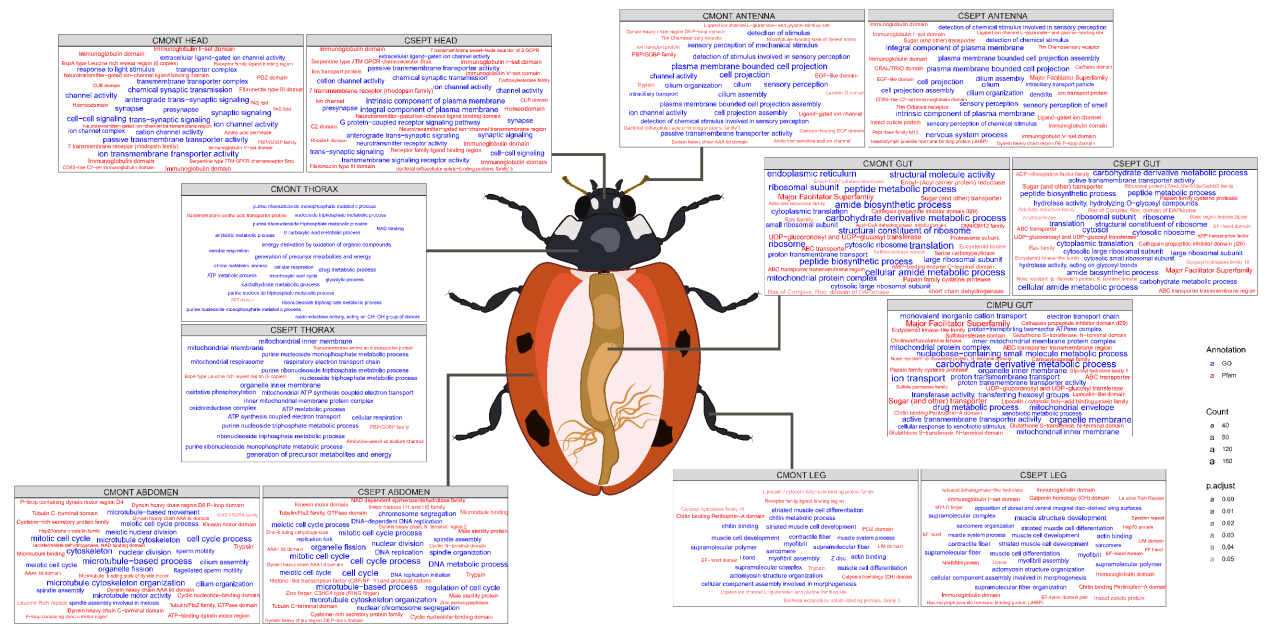


Figure S6.2 Enrichment of gene ontology (GO) or Pfam annotation of highly expressed genes in different tissues of the ladybirds. Only the top 20 GO or Pfam annotations in the enrichment results are shown. A high-quality figure can be downloaded from https://github.com/huangyh45/ladybird-genomes-supplementary-figures.


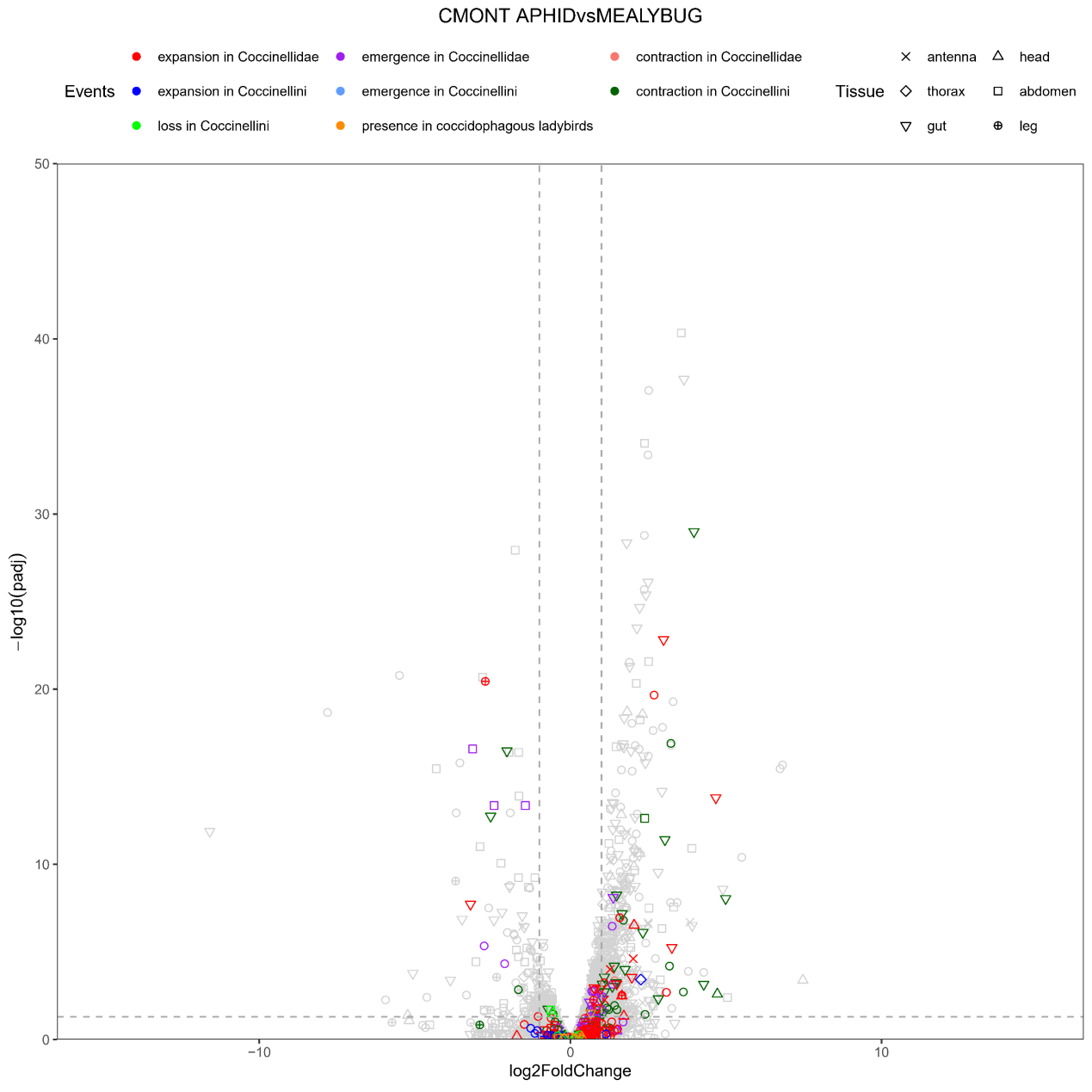


Figure S6.3 Volcano plot of APHID vs MEALYBUG diet transcriptome comparison in female adults of CMONT. The evolution events at the ancestor of Coccinellidae or Coccinellini are marked by color, and the tissue-specific highly expressed genes are marked by shape. The hollow circles represent the genes without tissue-specific expression patterns. A high-quality figure can be downloaded from https://github.com/huangyh45/ladybird-genomes-supplementary-figures.


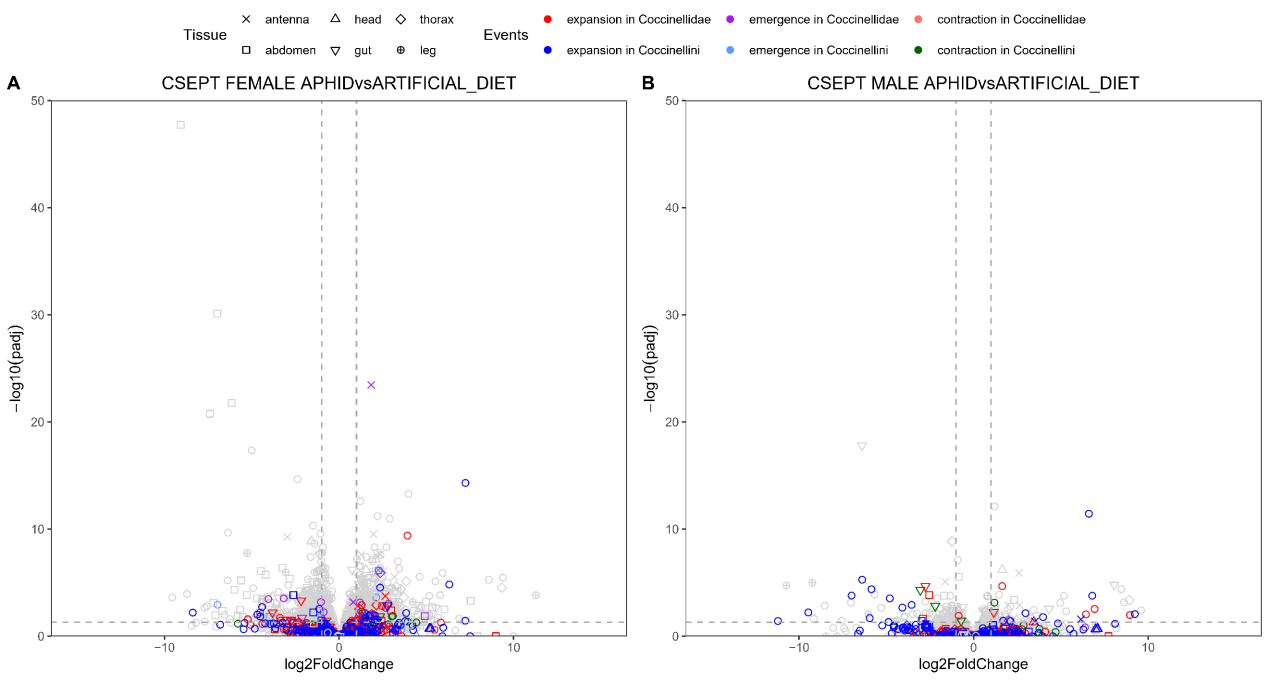


Figure S6.4 Volcano plot of APHID vs ARTIFICIAL_DIET transcriptome comparison in female adults of CSEPT. The evolution events at the ancestor of Coccinellidae or Coccinellini are marked by color, and the tissue-specific highly expressed genes are marked by shape. The hollow circles represent the genes without tissue-specific expression patterns. A high-quality figure can be downloaded from https://github.com/huangyh45/ladybird-genomes-supplementary-figures.


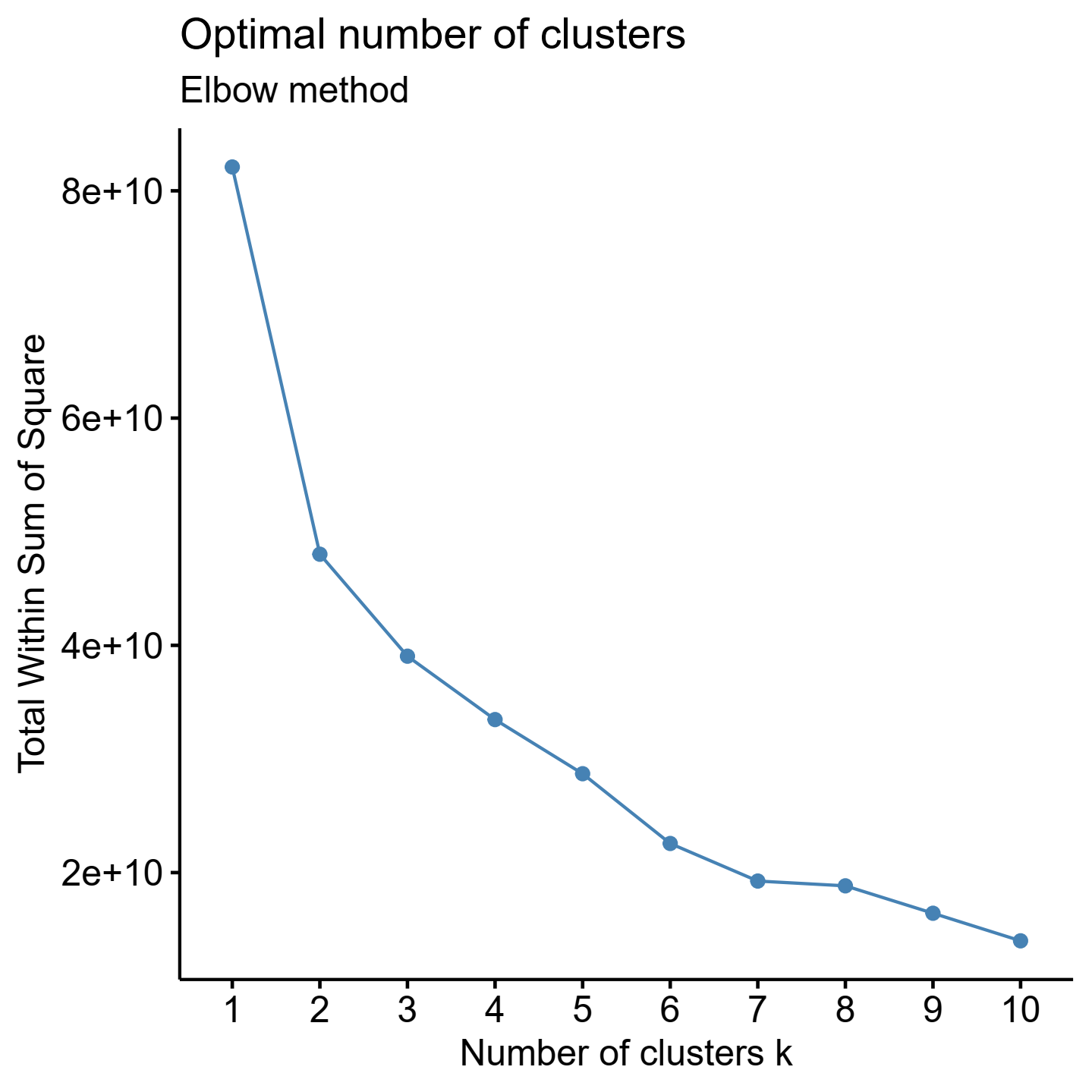

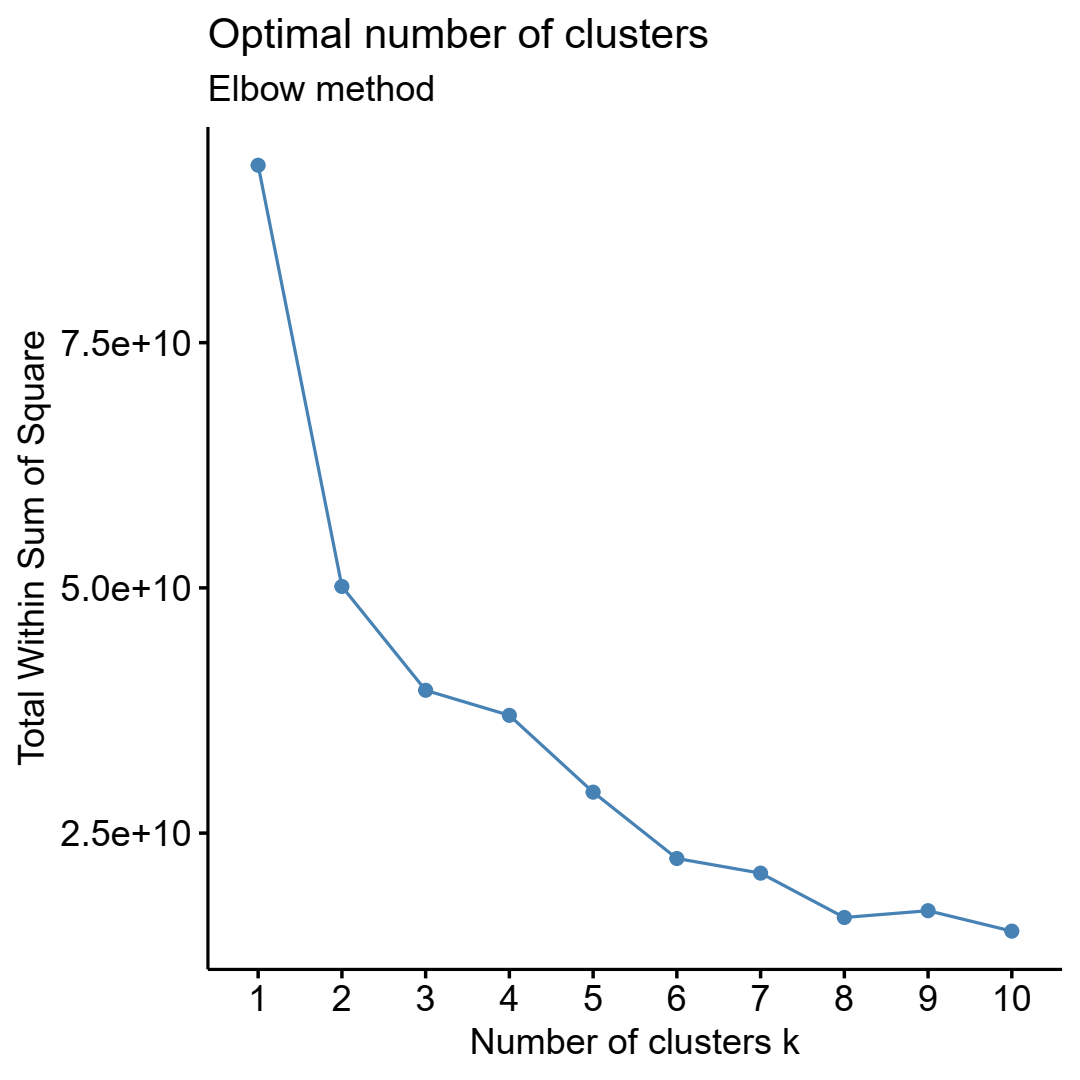


Figure S6.5 Selection of the best k value for CMONT (left) and CSEPT (right)


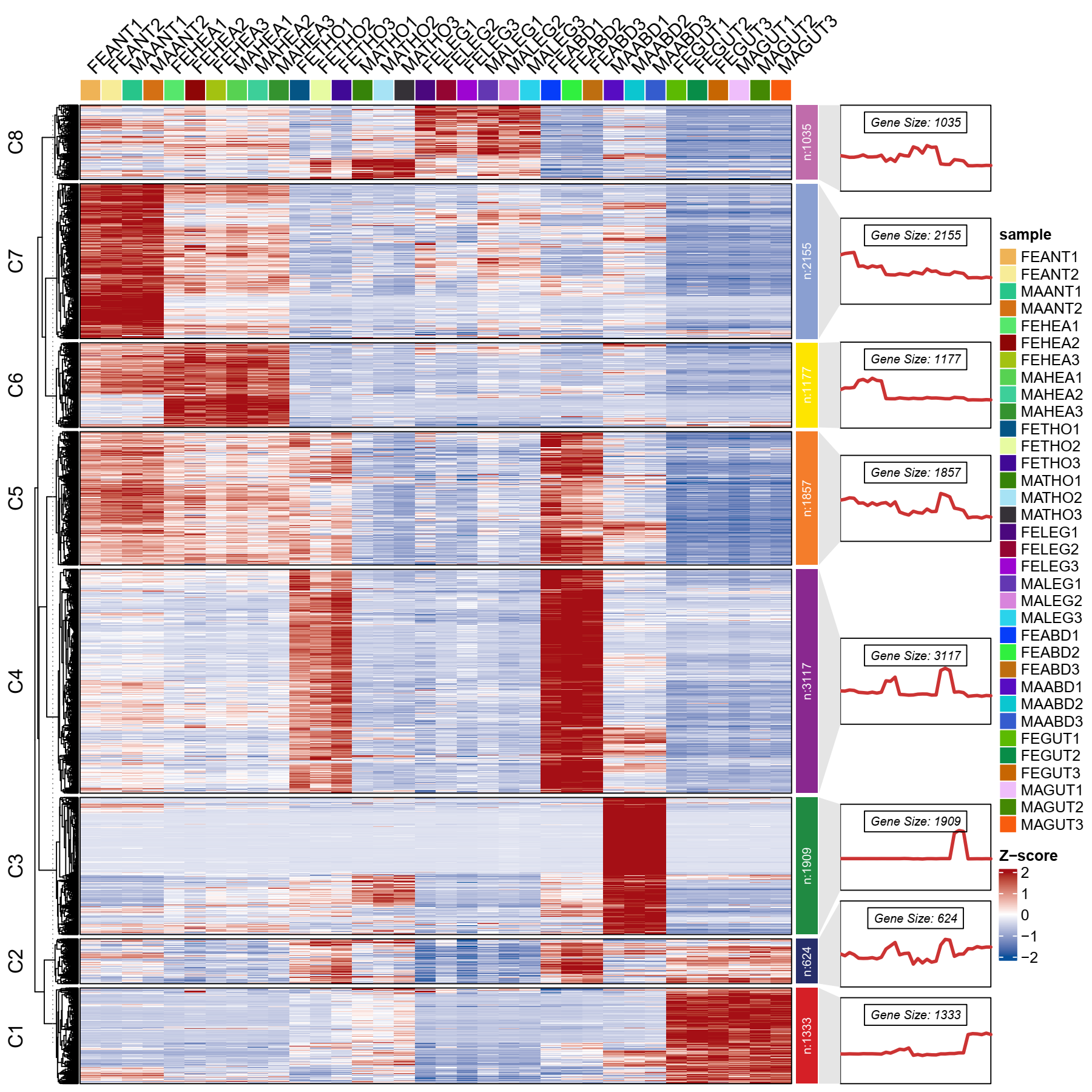


Figure S6.6 K-means clustering of CMONT. The genes with mean FPKM values < 1 are excluded.


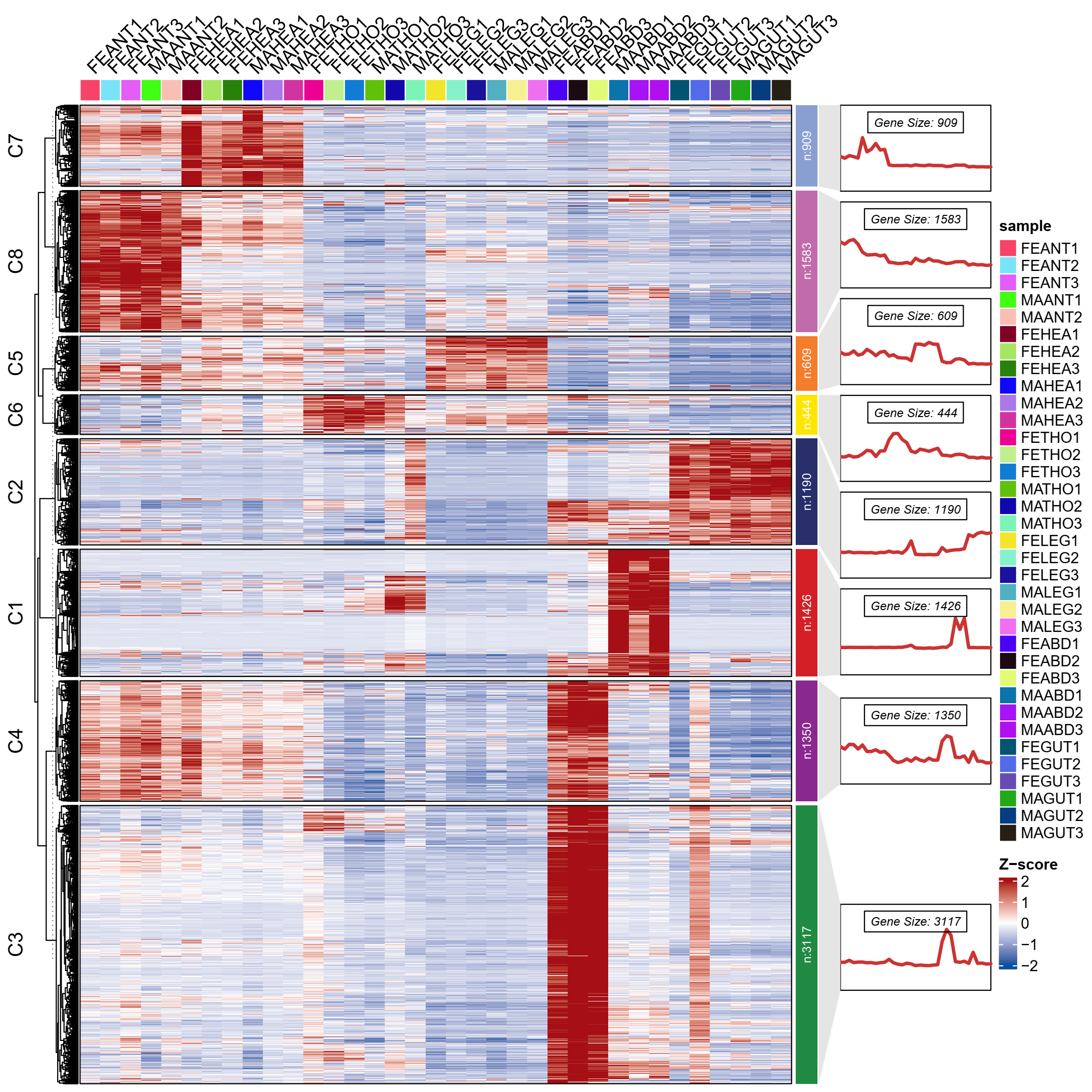


Figure S6.7 K-means clustering of CSEPT. The genes with mean FPKM values < 1 are excluded.

# 7 Evolution of candidate chemosensory genes

## 7.1 Materials and methods

The OGs of candidate gene families were identified using a three-step method to ensure that all the OGs were found. First, reference genes were used as query to search the protein sets of the extended dataset by BLASTP (Camacho et al., 2009), with the E-value cutoff of 1e-5. If an OG includes >= 75% genes as BLAST hits of the reference genes, it was considered as a candidate OG and the seed of the downstream steps. Second, the Pfam annotations of the candidate OGs inferred by KinFin above in Section 1.1.7 were manually checked. The Pfam accessions which were considered accord with the candidate gene families are set as the search criteria. The OGs with the target Pfam annotations are also added into the candidate OGs. Third, the sequences from all the candidate OGs of a specific gene family were collected and aligned by MAFFT v7.480 (Katoh and Standley, 2013). Then the alignment was used as a reference to search the protein sets by HMMER v3.3 (Mistry et al., 2013) with the E-value cutoff of 1e-5. If an OG includes >= 75% genes in the HMMER results and is not included in the candidate OGs, it was added into the candidate list.

In order to connected the report functions of the specific genes to our data, we collected the reported and available genes of the ladybirds and used them as query to search our protein sets of the extended dataset by BLASTP with the E-value cutoff of 1e-5. The protein with best hit to the query, percent identity >= 95, coverage >= 95 and the same species as the query were considered as the same protein as the query and the reported function were thus connected.

The genes in the candidate OGs were used to reconstruct the phylogeny by IQ-TREE (Minh et al., 2020) with 1000 UFBoot replicates after MAFFT alignments and trimAl v1.4 (Capella-Gutierrez et al., 2009) processing, respectively. The rooted species tree and the OG trees were submitted to OrthoFinder v2.5.4 (Emms and Kelly, 2019), in order to resolve the OG trees and identify the phylogenetic hierarchical orthogroups (HOGs) of the node of Coccinellidae in the species tree, which could help manually analyze the groups of the target OGs. The gene duplication events at the nodes of Coccinellidae, Coccinellini or Epilachnini within the OGs are also detected via gene tree reconciliation at the same time, with support values >= 0.8. The FPKM values and expression patterns of different diet treatments and different tissue of the genes included in the target OGs were obtained using the methods in Section 5.1.2 and Section 6.1. Only the diet-specific expression patterns in fourth instar larvae or female adults of the carnivorous ladybirds, CMONT, PJAPO, HAXYR, CSEPT and MDISC, and SUGAR5d versus PLANT comparison of the herbivorous ladybird HVIGI (Table S5.1) were considered in the gene family analyses, which had relatively high reliability with the reference genome. Motifs of the protein sequences of the target OGs were predicted by MEME v5.4.1 (Bailey and Elkan, 1994). Domains of the proteins in the target OGs were identified in Pfam v35.0 database (Mistry et al., 2021) using PfamScan script (http://ftp.ebi.ac.uk/pub/databases/Pfam/Tools/). Composition of sites in sequences was shown by the R package ggseqlogo (Wagih, 2017). The visualization of the OG trees was mainly performed by the R package ggtree (Yu et al., 2017).

For candidate chemosensory genes, the reference genes obtained from the antennal transcriptomes of *H. axyridis* (HAXYR) (Rondoni et al., 2021) (Table S7.1). And the identification and analyses of the chemosensory OGs were performed as described above. Specifically, we performed molecular docking analyses of the OBPs to explore the candidate odor molecule ligands. The 3D structure of the OBPs were constructed based on the protein sequences on trRosetta (https:// yanglab.nankai.edu.cn/trRosetta/) (Du et al., 2021). Only the modeled structures with TM-score >= 0.7 were considered available. Then the quality of the 3D structure models was checked by SAVES v6.0 server (https://saves.mbi.ucla.edu/). The ligand 3D structures of eight representative volatiles from aphids, plants, ladybirds and coccids (Table S7.3) were downloaded from PubChem (https://pubchem.ncbi.nlm.nih.gov/) (Kim et al., 2023). Molecular Operating Environment 2019.0102 (MOE) was used to perform docking analyses. The binding pockets were predicted, and the pocket with the highest score was selected by docking analysis against the ligands. The best binding poses of ligands were ranked according to the docking score and the lowest scores were recorded.

Table S7.1 Information of candidate chemosensory genes

|  | Gene name | Pfam accession | No. orthogroups with genes >= 30 / total orthogroups |
| --- | --- | --- | --- |
| OR | Odorant receptor | PF02949 | 3/47 |
| IR | Ionotropic receptor | PF00060, PF10613 | 7/17 |
| GR | Gustatory receptor | PF01148, PF08395 | 3/36 |
| OBP | Odorant-binding protein | PF01395 | 14/120 |
| CSP | Chemosensory protein | PF03392 | 3/29 |
| SNMP | Sensory neuron membrane protein | PF01130 | 12/19 |

## 7.2 Results

Combining homology search to the reference chemosensory genes in *H. axyridis* (HAXYR) (Rondoni et al., 2021) with the information of the OGs, we identified and analyzed the chemosensory OGs in both the genome dataset and the extended dataset. Details of identification of OG and the expression patterns of their genes can be found in Additional file 2: Table SE6 and SE5 and the corresponding node numbers are shown in Figure S3.1.

### 7.2.1 Chemosensory receptors: GR, IR, OR

Three OGs of OR, seven OGs of IR and three OGs of GR was identified with >= 30 gene members in all the species. Among all these OGs, only the largest OG of OR, OG0000083, is found to contract in Coccinellidae (Figure S7.1), with relatively low expression of most genes in the whole-body individuals in the diet treatments (Additional file 2: Table SE5). ORs detect volatile chemicals, including pheromones, and food-, plant- or microbe-derived compounds (Leal, 2013). Recently an OR of ladybird is reported to be related to perception of aphid-induced, aphid-derived, and plant-derived volatiles (Xie et al., 2022). Therefore, this evolution event is probably associated with the evolution of feeding habits of ladybirds, or the family-specific pheromone communication. For example, contraction of this OG may lead to lost ability to feel volatiles not related to prey and plant, as occurred in herbivorous insects compared with other insects (Obiero et al., 2021; Oeyen et al., 2020). Unfortunately, although GRs, IRs and ORs are expressed actively in the antenna or head of the ladybirds, as reported in Rondoni et al. (2021), only low expression of most GRs, IRs and ORs are found in our whole-body transcriptomic data of different diet treatments, which prevents us to analyze the expression regulations of most GRs, IRs and ORs responding to different diets and consider if the evolution events are related to evolution of carnivory in ladybird.

Only considering OGs with enough expression in the whole body, we found that in the largest IR OG, OG0000243, Clade C2 is Coccinellini-specific, while Clade C1 lost the genes of Coccinellini (Figure S7.2). Genes in this OGs are mainly expressed highly in the head, but no diet-specific DEGs in the ladybirds are detected. Therefore, the results cannot support the connection of OG0000243 to evolution of feeding habits in the ladybirds.

### 7.2.2 Soluble binding protein: OBP, CSP, and SNMP

We identified fourteen OGs as OBPs, three OGs as CSPs and twelve OGs as SNMPs with gene members >= 30.

-OBP

Among these OGs, the largest OG of OBP, OG0000120, significantly expands in Coccinellidae and Epilachnini, respectively (Figure S7.1). Five groups of the ladybirds are found in the gene tree of OG0000120, including Clade C1-C5 (Figure S7.2). Clade C5 mainly contributes to the expansion in Coccinellidae, with most genes highly expressed in the antenna, while most genes of Clade C2 and C3 are highly expressed in the head (Figure S7.3). Clade C5 also largely contribute to the expansion in Epilachnini, with a gene duplication event detected. Compared with other OGs of OBP, OG0000120 has higher ratio of diet-specific DEGs (32/61 genes, other OGs: 27/110 genes) (Figure 9.4). For example, the coccidophagous ladybird CMONT has 5/8 genes upregulated when the larvae feed on the optimal diet MEALYBUG compared with other diets, APHID or MOTHEGG respectively. And the aphidophagous ladybird MDISC has 4/11 genes downregulated when the larvae feed on the optimal diet APHID compared with other diets, MEALYBUG, MOTHEGG or POLLEN respectively, which all belong to Clade C1. No sex-biased gene is found in OG0000120. In addition, OG0000159 of OBP significant expands in MDISC and contract in CSEPT, with 1/3 upregulated DEGs in PJAPO, 2/2 upregulated DEGs in CSEPT, 1/2 upregulated DEGs in HAXYR when they feed on MEALYBUG instead of APHID, and 1/3 downregulated DEGs in HVIGI when they feed on sugar water instead of plant leaves (Additional file 2: Table SE5).

-CSP

The largest OG of CSP is OG0000042, which mainly contains genes annotated as ejaculatory bulb-specific protein 3-like. The gene tree reveals that 14 groups of ladybird genes exist, among which Clade C14 consists of several subgroups of Coccinellidae and Epilachnini (Figure S7.2). However, no significant expansion is detected at the node of Coccinellidae and Epilachnini. A gene duplication event in Epilachnini occurs in Clade C12, but without diet-specific DEGs in HVIGI (Figure S7.4). Most genes in Clade C14 are with higher expression in the antenna, head and/or leg than other tissues, with 9/20 genes significantly higher in the antenna or head (Figure S7.3). Similarly, the genes in Clade C1 are also expressed more highly in the antenna. When the ladybirds feed on non-optimal diets, large amounts of CSP genes are upregulated and most of the DEGs are members of OG0000042 (Figure S7.4). No sex-biased genes are found in OG0000042.

-SNMP

OG0000509 of SNMP has at least two duplication events in Coccinellidae, leading to Clade C2-C4, with 2/3 upregulated DEGs in MOTHEGG-fed CMONT and 1/4 downregulated DEGs in sugar water-fed HVIGI (Figure S7.2, S7.4). Genes in this OG are highly expressed in head or gut. In OG0001163 of SNMP with most genes annotated as protein croquemort-like, a gene duplication events occur in Epilachnini. The duplicated clades contain 1/2 downregulated DEGs when HVIGI feeds on sugar water. Genes in OG0001163 are mainly expressed in leg or gut.

In addition, several OGs of OBP and CSP are under emergence in Coccinellidae or Epilachnini, including OG0009444 of OBP emergent in Coccinellidae, OG0018304 and OG0023486 of OBP and OG0026388 of CSP emergent in Epilachnini. OG0001204 of OBP has a Coccinellini-specific clade. But few diet-specific DEGs (only 1/15 of PJAPO in OG0001204) are found in these clades.

CSPs and insect OBPs, are known to bind the semiochemicals through the sensillar lymph and activate or enhance the sensitivity of specific ORs, playing a key role in an insect’s successful perception of sex pheromones, alarm pheromones, and host plant volatiles (Pelosi et al., 2014; Pelosi et al., 2006). The carnivorous ladybirds can receive alarm pheromone of prey (e.g. (E)-β-farnesene from the aphids) and the damaged or undamaged plant volatiles through their OBPs (Pervez and Yadav, 2018; Qu et al., 2022a; Tang et al., 2023a; Yang et al., 2023), while the herbivorous ladybirds can also locate the host plant through feeling the plant volatiles (Piersanti et al., 2022). Our analysis found several expanding or emergent OBP and CSP OGs in Coccinellidae and/or Epilachnini, especially OG0000120, within which DEGs between different diets are detected. The genes in these OGs are mostly expressed higher in the antenna and/or head, which seem to be related to chemosensation. These results indicate that these expansions and emergences of OBPs and CSPs are probably related to food forage through prey or plant volatiles during diet adaptation at the ancestor of Coccinellidae or Epilachnini. Molecular docking analyses by MOE also reveal that OBPs of different ladybirds in OG0000120 have relatively low binding energy (docking score < -5) to aphid alarm pheromone, mealybug sex pheromone and plant volatiles, as similar to OBPs in OG0000490, which contain the reported OBP in HAXYR binding aphid alarm pheromone and several plant volatiles (Qu et al., 2022a) (Table S7.2, S7.3). This indicates the potential ability of the expanding OG0000120 to feel the volatiles from prey and plant, but binding assays, electroantennographic experiment and RNA inference are still needed to verify these functions.

A gene of HAXYR in OBP0000120 (HAXYR.125) have been reported to be expressed higher in the female adults (Qu et al., 2021) (Table S7.2), which indicate that this OG may also have the binding ability of sex pheromones. Actually, some sex pheromones of the ladybirds are the same as some herbivore-induced plant volatiles, such as (−)-β-caryophyllene and α-humulene in HAXYR (Tang et al., 2022a; Tang et al., 2023a; Verheggen et al., 2020), and this OG possibly has dual/multiple functions in recognition of sex pheromone components and others, e.g., plant volatiles, as similar to some OBPs in other insects (Brito et al., 2016).

When the ladybirds feed on non-optimal diets, the genes from OG0000120 are upregulated or downregulated, and the CSP genes are mainly upregulated, which is inconformity to the propose that chemosensory genes are used to search the volatiles related to the optimal diet (Seagraves, 2009). It is more likely that the ladybirds search their diet by responding to the positive and negative stimuli of volatiles from their diets (Seagraves, 2009).

## 7.3 Summary

We analyzed the OGs related to six types of chemosensory genes in this study. Gene counts in the OGs of IR and SNMP are relatively similar in different species, while most LEGFs are the OGs of GR, OR, OBP and CSP. Among these LEGFs, the largest OG of OBP, OG00000120 expands in Coccinellidae and Epilachnini, and contain high ratio of diet-specific DEGs in the ladybirds, which indicates its important role in evolution of feeding habit in the ladybirds. In addition, OG0000509 of SNMP undergoes two duplication events at clade-level in Coccinellidae and contains diet-specific DEGs in the ladybirds, which is possibly related to feeding habits.

Table S7.2 The reported function of the candidate chemosensory genes in the ladybirds.

| Reported gene name | Gene ID | Ortholog group | Gene family | High expression in qPCR | Functional verification | Reference |
| --- | --- | --- | --- | --- | --- | --- |
| HaxyOBP1 | HAXYR.9865 | OG0000609 | OBP | whole body except antenna and legs of the adults | - | Qu et al. (2021) |
| HaxyOBP2 | HAXYR.8828 | OG0001775 | OBP | adult antenna | - | Qu et al. (2021) |
| HaxyOBP3 | HAXYR.12474 | OG0000159 | OBP | adult antenna | bind the volatiles from the plants | Qu et al. (2021); Qu et al. (2022a) |
| HaxyOBP4 | HAXYR.13864 | OG0000308 | OBP | female adult heads | - | Qu et al. (2021) |
| HaxyOBP5 | HAXYR.7826 | OG0000490 | OBP | larvae, adult antenna | bind the volatiles from the plants | Qu et al. (2021); Qu et al. (2022a) |
| HaxyOBP6 | HAXYR.12620 | OG0008518 | OBP | whole body of the adults | - | Qu et al. (2021) |
| HaxyOBP7 | HAXYR.6425 | OG0001204 | OBP | whole body of the adults | - | Qu et al. (2021) |
| HaxyOBP8 | HAXYR.12474 | OG0000159 | OBP | adult antenna, heads, legs and wings | - | Qu et al. (2021) |
| HaxyOBP9 | HAXYR.10794 | OG0000609 | OBP | whole body of the adults | - | Qu et al. (2021) |
| HaxyOBP10 | HAXYR.125 | OG0000120 | OBP | female adult antenna | - | Qu et al. (2021) |
| HaxyOBP11 | HAXYR.3451 | OG0008762 | OBP | adult wings | - | Qu et al. (2021) |
| HaxyOBP12 | HAXYR.4847 | OG0001329 | OBP | adult antenna | bind the volatiles from the plants | Qu et al. (2021); Qu et al. (2022a) |
| HaxyOBP13 | HAXYR.10836 | OG0001329 | OBP | male adult heads | - | Qu et al. (2021) |
| HaxyOBP14 | HAXYR.1753 | OG0000490 | OBP | male adult heads | - | Qu et al. (2021) |
| HaxyOBP15 | HAXYR.10522 | OG0000490 | OBP | adult antenna | bind the volatiles from the aphids and the plants | Qu et al. (2021); Qu et al. (2022a) |
| HaxyOBP16 | HAXYR.8841 | OG0003462 | OBP | whole body of the adults | - | Qu et al. (2021) |
| HaxyOBP17 | HAXYR.168 | OG0012636 | OBP | adult abdomens | - | Qu et al. (2021) |
| HaxyOBP18 | HAXYR.3035 | OG0000120 | OBP | adult thoraxes | - | Qu et al. (2021) |
| HaxyOBP19 | HAXYR.8057 | OG0001204 | OBP | female adult antenna, male adult heads and adult legs | - | Qu et al. (2021) |
| HaxyOBP1_Han | HAXYR.12862 | OG0000490 | OBP | male adult heads or antenna | - | Han et al. (2019) |
| HaxyOBP6_Han | HAXYR.6209 | OG0000159 | OBP | male adult heads or antenna and female adult wings | - | Han et al. (2019) |
| HvarOBP1 | Tran_HVARI.35671 | OG0008973 | OBP | adult antenna | - | Tang et al. (2023a) |
| HvarOBP3 | Tran_HVARI.26222 | OG0000159 | OBP | larvae, adult legs | - | Tang et al. (2023a) |
| HvarOBP5 | Tran_HVARI.40074 | OG0001329 | OBP | adult antenna | feel the semiochemical cues from the aphids and habitat plants­ | Tang et al. (2023a) |
| HvarOBP8 | Tran_HVARI.32388 | OG0001775 | OBP | larvae, adult antenna | - | Tang et al. (2023a) |
| HvarOBP9 | Tran_HVARI.29152 | OG0008518 | OBP | larvae, adult legs and wings | - | Tang et al. (2023a) |
| HvarOBP10 | Tran_HVARI.6362 | OG0000490 | OBP | adult heads | - | Tang et al. (2023a) |

Table S7.3 Molecular docking analysis of ligands and its binding energy toward the ladybird OBPs in OG0000120 and OG0000490.

| Type | Ligand | PubChem CID | Reference | OG0000120 | | | OG0000490 | | |
| --- | --- | --- | --- | --- | --- | --- | --- | --- | --- |
|  |  |  |  | CMONT.3585 | CSEPT.9119 | HVIGI.13875 | CMONT.4963 | HAXYR.10522 | HVIGI.1836 |
| Aphid alarm pheromone | (E)-β-Farnesene | 5281517 | Qu et al. (2022a); Tang et al. (2023a) | -6.07 | -6.12 | -6.22 | -5.93 | -6.09* | -5.85 |
| Aphid sex pheromone | (4aS,7S,7aR)-Nepetalactone | 161367 | Birkett and Pickett (2003) | -5.18 | -4.80 | -4.55 | -5.13 | -5.15 | -5.09 |
| Plant volatiles | β-Ionone | 638014 | Qu et al. (2022a); (Tang et al., 2022a) | -5.74 | -5.68 | -5.45 | -5.77 | -5.35* | -5.52 |
| Herbivore-induced plant volatiles | Methyl Salicylate | 4133 | Yang et al. (2023) | -4.82 | -4.68 | -4.58 | -5.01 | -4.71 | -4.74 |
| Ladybird volatiles | 2-isopropyl-3-methoxypyrazine | 33166 | Al Abassi et al. (1998); Cai et al. (2007) | -5.22 | -4.75 | -4.86 | -5.11 | -4.89 | -5.18 |
| Ladybird sex pheromone | (-)-β-Caryophyllene | 5281515 | Verheggen et al. (2020) | -5.73 | -5.02 | -4.93 | -5.25 | -5.20 | -5.40 |
| Coccid and fungi volatiles | 1-Octen-3-Ol | 18827 | Li et al. (2016b); (Tabata et al., 2011) | -4.99 | -4.71 | -4.78 | -5.03 | -4.77 | -5.06 |
| Mealybug sex pheromone | Chrysanthemyl 2-Acetoxy-3-Methylbutanoate | 45112181 | Urbina et al. (2018) | -6.94 | -6.23 | -6.76 | -7.07 | -6.48 | -6.89 |

* The binding ability has been reported based on binding assay in Qu et al. (2022a).


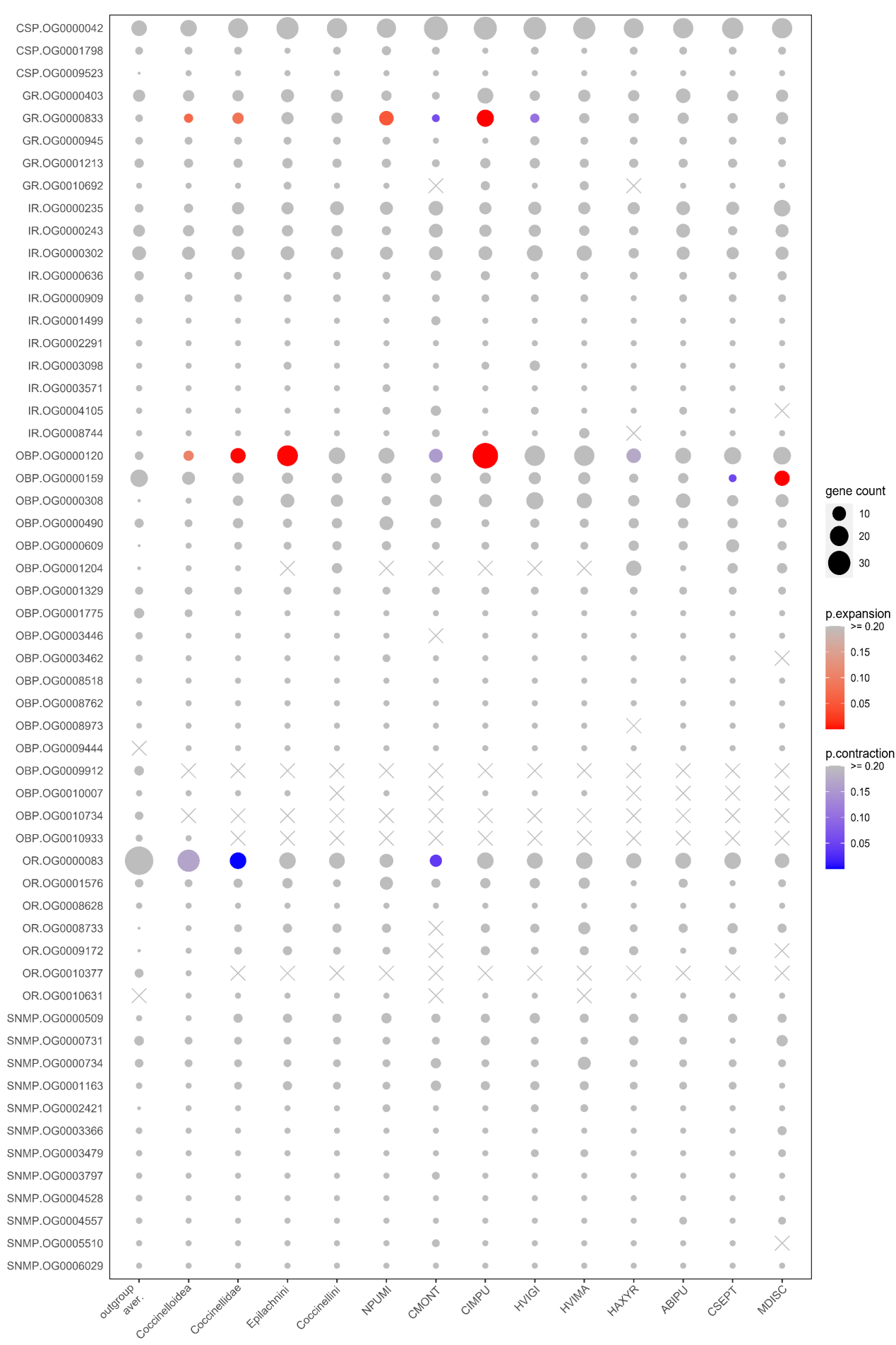


Figure S7.1 Comparison of gene counts in ortholog groups (OGs) related to chemosensation in the ten ladybird genomes. Only OGs with total genes >= 30 are shown. Gene counts at the nodes of Coccinelloidea, Coccinellidae, Epilachnini and Coccinellini are reconstructed by CAFE. A high-quality figure can be downloaded from https://github.com/huangyh45/ladybird-genomes-supplementary-figures.


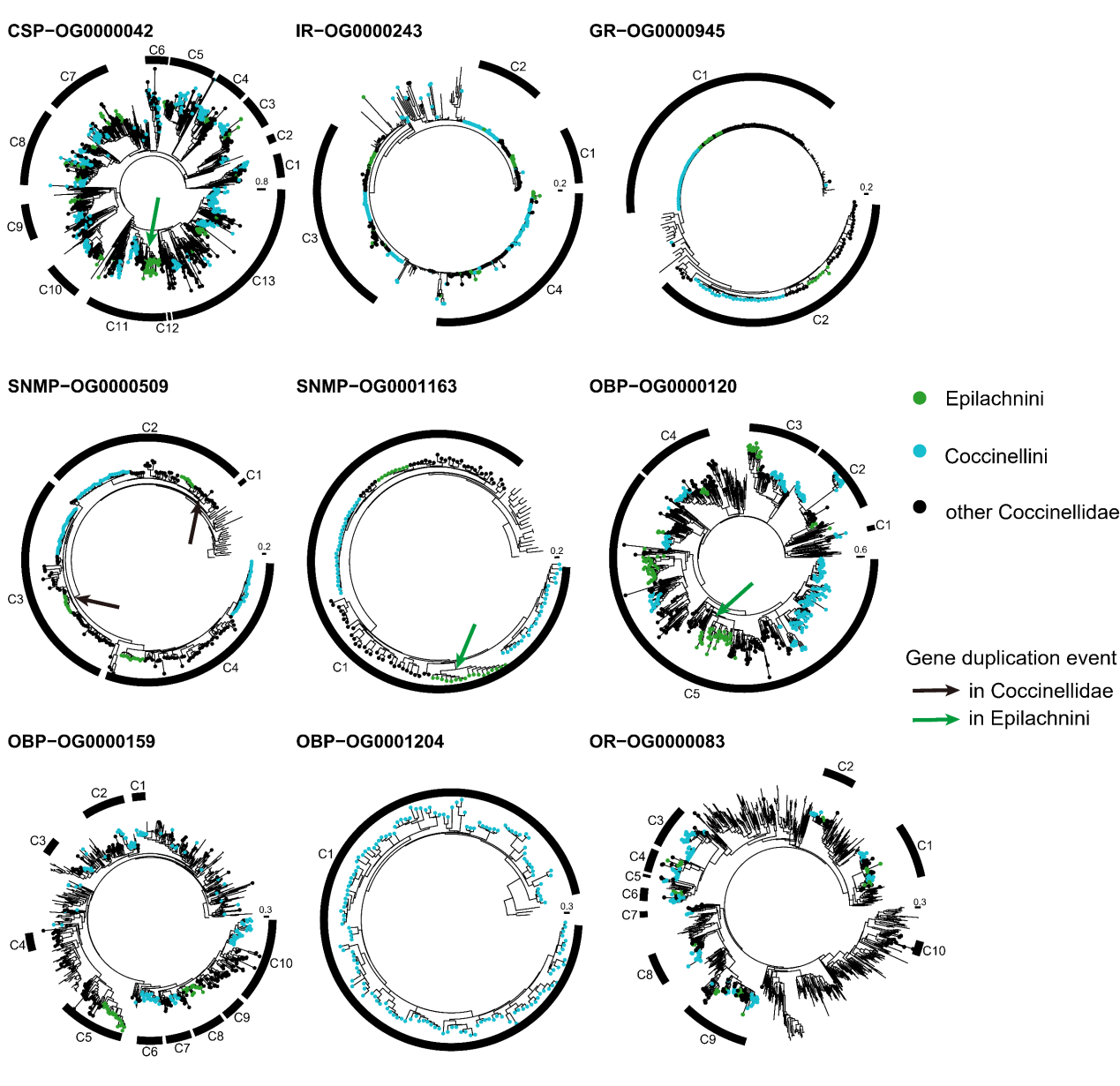


Figure S7.2 Gene trees of ortholog groups (OGs) of chemosensory genes. The genes in the ladybirds are marked in color and the others without circles are the genes in the outgroup beetles. A high-quality figure can be downloaded from https://github.com/huangyh45/ladybird-genomes-supplementary-figures.


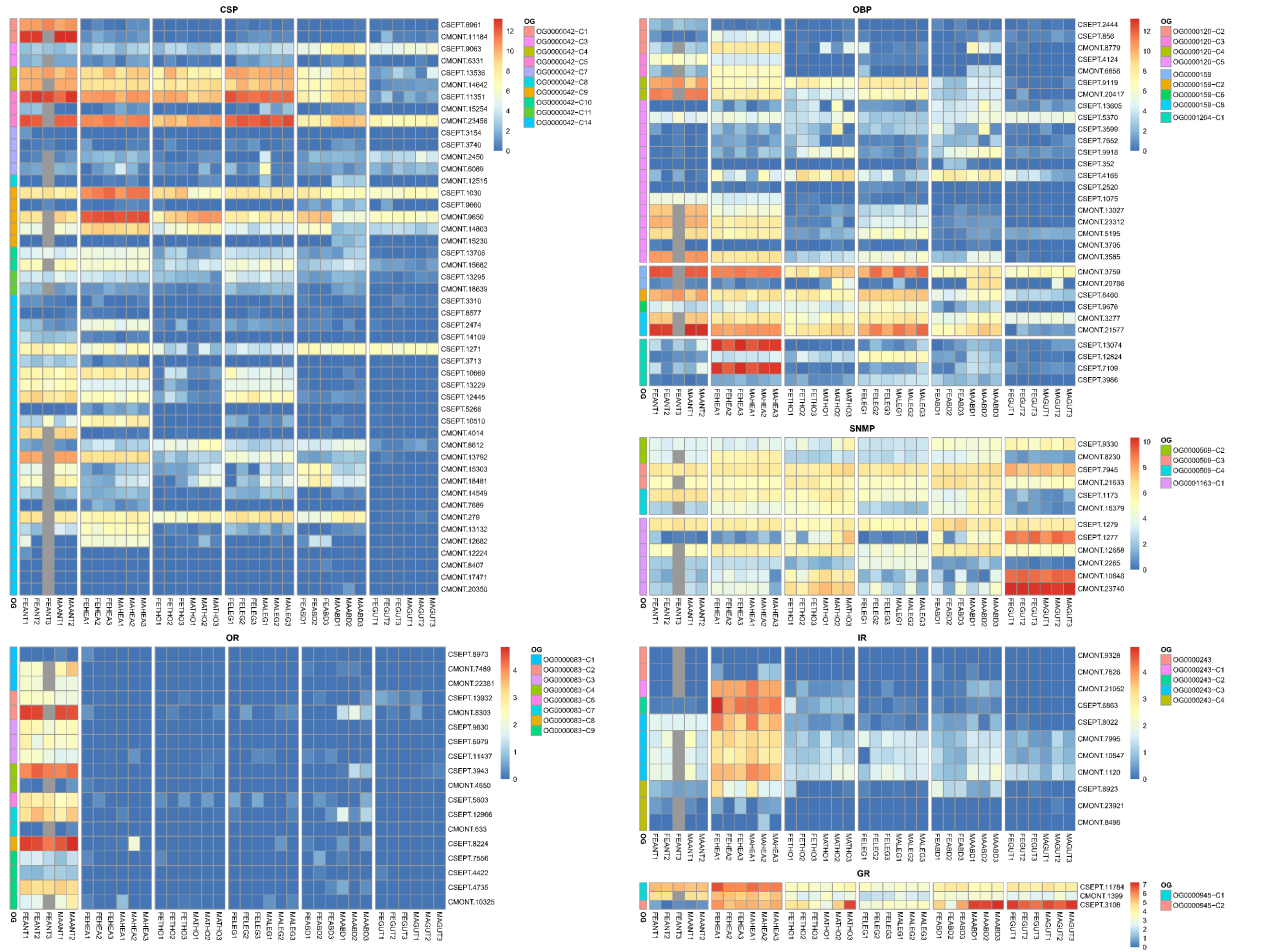


Figure S7.3 Expression patterns of different tissue of each group in ortholog groups (OGs) of chemosensory genes. Abbreviation in the sample names: FE: female adult, MA: male adult, ANT: antenna, HEA: head, THO: thorax, LEG: leg, ABD: abdomen, GUT: gut. A high-quality figure can be downloaded from https://github.com/huangyh45/ladybird-genomes-supplementary-figures.


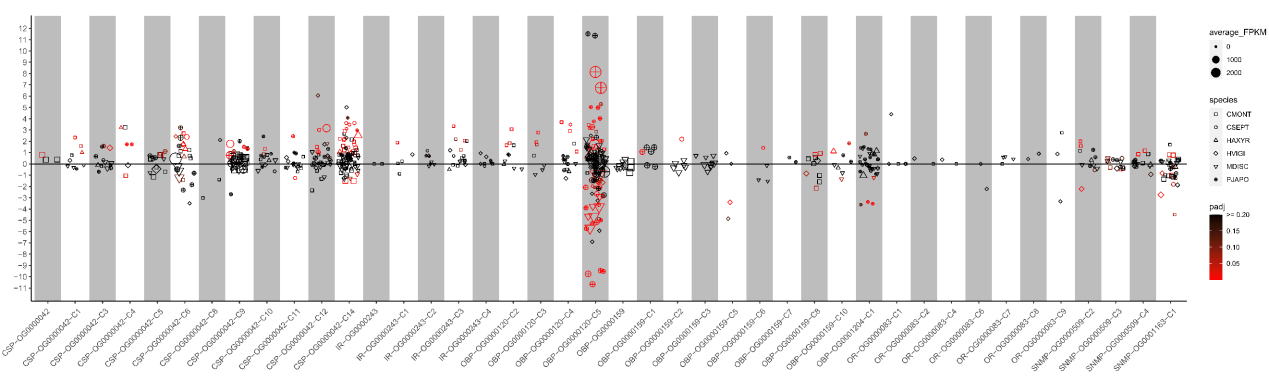


Figure S7.4 Expression patterns under different diet treatments of each group in ortholog groups (OGs) of chemosensory genes. The vertical axis represents the log2(fold change) values. The positive values on the vertical axis represent upregulation when feeding on the optimal diets, while the negative values represent upregulation when feeding on the non-optimal diets. A high-quality figure can be downloaded from https://github.com/huangyh45/ladybird-genomes-supplementary-figures.

# 8 Evolution of candidate genes related to nutrient digestion

## 8.1 Materials and methods

The methods to identify and analyze the OGs related to nutrient digestion were the same as the methods for chemosensory genes in Section 7.1. The reference genes as the query were the candidate digestion genes of *Tribolium castaneum* (TCAST) from the NCBI Protein database. These nutrient digestion genes were obtained through searching the keywords (Table S8.1) on NCBI. Specially, we also considered digestive chitinase (Arakane and Muthukrishnan, 2010; Woodring, 2020) since most ladybirds are carnivorous and need to digest the chitin of prey, and two genes related to peritrophic membranes or potential cellulase activity, CBPD and LMPO (Jasrapuria et al., 2010; Qu et al., 2022b; Qu et al., 2022c; Rodriguez-de la Noval et al., 2019; Sabbadin et al., 2018; Tetreau et al., 2015), were considered as well. Chitinase (PF00704), CBPD (PF01607) and LPMO (PF03067) were initially identified through Pfam annotations of OGs in the second step of identification and the first step was skipped. In order to further explore the glucosidases and chitinases, the protein sequences from the target OGs were combined with reported glucosidases (only GH1) (Beran et al., 2014; Byeon et al., 2005; Cairo et al., 2013; Ferreira et al., 2001; Jones et al., 2002; Pentzold et al., 2017; Weil et al., 2007) and chitinases (Genta et al., 2006; Zhu et al., 2008a; Zhu et al., 2008b) with clear function, and the phylogenetic analyses were conducted.

Table S8.1 Candidate genes associated with nutrient digestion

|  | Gene name | Notes | Pfam accession | No. orthogroups with genes >= 30 / total orthogroups |
| --- | --- | --- | --- | --- |
| CP | Carboxypeptidase | Exopeptidase | PF00246, PF02244 | 9/50 |
| AP | Aminopeptidase | Exopeptidase | PF01433, PF17900, PF11838 | 11/42 |
| SP | Serine proteinase | Endopeptidase, including trypsin, brachyurin | PF00089 | 52/346 |
| CAT | Cysteine proteinase | Endopeptidase, including cathepsin B and L | PF00112, PF08127, PF08246 | 10/74 |
| ASP | aspartic proteinases | Endopeptidase, including cathepsin D | PF14543, PF00026 | 2/29 |
| MMP | metalloproteinase | Endopeptidase | PF00413, PF00045 | 5/21 |
| AMY | Alpha-amylase | For the initial digestion of carbohydrates | PF00128 | 6/12 |
| GLC | Alpha-glucosidase  Beta-glucosidase  Myrosinase | For the subsequent degradation into monosaccharides | PF00232, PF01055 | 3/18 |
| LIP | Lipase | Glycerol ester hydrolase for lipid digestion, including phospholipase | PF04083 | 12/50 |
| CHT | Chitinase | For chitin degradation | PF00704 | 10/22 |
| NAT | nutrient amino acid transporter | amino acid transporter | PF00209 | 11/23 |
| GLUT | Glucose transporter protein | Transporter of monosaccharide | PF07690, PF00083 | 75/318 |
| SGLT | Sodium-driven glucose symporter | Transporter of monosaccharide, sodium-coupled monocarboxylate transporter | PF00474 | 6/17 |
| FABP | Fatty acid binding protein | Transporter of free fatty acids | PF00061 | 6/28 |
| FATP | Fatty acid transport protein | Transporter of free fatty acids | PF13193, PF00501 | 15/42 |
| NPC | Niemann-Pick C1 | Transporter of sterol | PF12349, PF02460 | 7/23 |
| SCP | Sterol carrier protein | Transporter of sterol | PF00108, PF02036, PF02803 | 8/29 |
| CBPD | chitin binding Peritrophin-A domain containing protein | Chitin binding protein, particularly peritrophic matrix proteins of insects and animal chitinases | PF01607 | 6/72 |
| LPMO | lytic polysaccharide mono-oxygenase | Redox enzyme of polysaccharide (mainly cellulose and chitin) that act in conjunction with carbohydrate-active enzymes | PF03067 | 3/3 |

## 8.2 Results

As the same method as the chemosensory genes with TCAST digestive reference genes, we used both the genome dataset and the extended dataset to identify and analyze the nutrient digestion-related OGs. Details of identification of OG and the expression patterns of their genes can be found in Additional file 2: Table SE6 and SE5 and the corresponding node numbers are shown in Figure S3.1.

### 8.2.1 Candidate genes encoding digestive enzymes

We identified nine OGs as CPs, eleven OGs as APs, 52 OGs as SPs, ten OGs as CATs, two OGs as ASPs, five OGs as MMPs, six OGs as AMYs, three OGs as GLCs, twelve OGs as LIPs and ten OGs as CHTs, with at least 30 gene members.

-MMP, SP, CP, ASP

In the peptidases and proteinases, one OG of MMP, OG0000287, which mainly contains matrix metallopeptidase 3, expand in Epilachnini and contract in Coccinellini, while one OG of SP, OG0000255, mainly including trypsin-like genes, expand in Coccinellidae and Epilachnini (Figure S8.1). The gene tree of OG0000287 reveals that the expansion in Epilachnini occur in Clade C2 and C7 (Figure S8.2). The genes in OG0000287 are with relatively low expression in gut, with only one gene expressed highly in female abdomen (Figure S8.3). When the ladybirds feed on non-optimal diets, no DEGs are detected (Figure S8.4). For OG0000255, Clade C7 is the largest group in the gene tree. Most genes in this OG are highly expressed in the male abdomen instead of other tissue, without diet-specific DEGs detected. Therefore, we cannot consider that these two OGs are related to feeding habits due to lack of support evidence of diet-specific and tissue-specific expression. Additionally, OG0000196 of CP (venom serine carboxypeptidase-like), OG0000380 (lysosomal aspartic protease) and OG0003251 (lysosomal aspartic protease-like) of ASP are detected to have a gene duplication event in Epilachnini. These OGs are mainly gut-specific and contain 2/2, 1/2 and 2/2 downregulated DEGs in sugar water-fed HVIGI. OG0000164 of SP mainly containing genes annotated as serine protease snake-like or venom protease, lost genes of Coccinellini in Clade C8 without any diet-specific DEGs in CMONT and HVIGI, while Clade C3 in OG0000440 of MMP consists of only Epilachnini species with 2/2 downregulated DEGs in sugar water-fed HVIGI, but gene expression in this OG is mainly antenna-specific.

-CAT

OG0000065 of CAT (procathepsin L-like) significantly contract in Coccinellini, and expand in Coccinellidae, though not significantly. However, a gene duplication event in Coccinellini is detected in Clade C11. It is also enriched from the diet-specific DEGs in several carnivorous ladybirds in Section 5.2.1, mainly including upregulated DEGs in CMONT and downregulated DEGs in Coccinellini when the ladybirds feed on non-optimal diets. Most genes in OG0000065 are highly expressed in the gut, which seem to be related to digest their prey. In the herbivorous ladybird HVIGI, 6/20 genes in OG0000065 are downregulated when they feed on sugar water. Similarly, OG0000293 of CAT (cathepsin B) also significantly contracts in Coccinellini, with most gut-specific genes and few diet-specific DEGs (1/6 downregulated in CMONT, 1/3 downregulated in MDISC and 2/3 upregulated in CSEPT). OG0000414 of CAT has an Epilachnini-specific clade C4, and genes are duplicated in this clade, but without diet-specific DEGs. CAT is previously reported as the main digestive enzyme in both the carnivorous ladybirds and herbivorous ladybirds (Álvarez-Alfageme et al., 2008; Gholamzadeh-Chitgar et al., 2017; Koo and Park, 2002; Walker et al., 1998; Zibaee, 2020), which support our discovery of diet-specific DEGs. In the adults of the coccidophagous ladybird CMONT, the activity will be reduced when they feed on aphids (Zibaee, 2020). Combined with expansion in Coccinellidae and contraction in Coccinellini, we considered that OG0000065 of CAT is likely to digest coccids or mealybugs rather than the aphids, probably due to different proteinaceous components or even glycoproteins in the aphids and coccids (Brown, 1975). The main upregulation in CMONT and downregulation in aphidophagous Coccinellini of CATs seem to be feedbacks to the amounts of some coccid-specific nutrition or components.

-Lipase

Additionally, OG0000139 of LIP is found to expand in Epilachnini, whose gene tree reveal that Clade C6 mainly contribute to the expansion (Figure S8.2). And genes of Coccinellini species are lost in Clade C4. However, the genes of OBP0000139 are not expressed obviously in specific tissues (Figure S8.3). Furthermore, few diet-specific DEGs are detected. In HVIGI, only 2/17 genes are downregulated DEGs under sugar water diet treatment (Figure S8.4). We do also not have enough evidence to support the relation between this OG and feeding habits.

-GLC

A GLC OG (OG0000110, GH1, mainly annotated as myrosinase 1-like) expanded at the node of Epilachnini without significance and significantly contract in Coccinellini. GH1 may be related to its herbivory through plant cell wall digestion or detoxification (Beran et al., 2014; McKenna et al., 2019). But no detected event can support that this OG is related to evolution of feeding habits of Epilachnini. In Clade C2, two Epilachnini-specific duplication events are found with only support of 0.67 or 0.78. It is also the only group with mainly gut-specific and plant leaf treatment-specific genes (Figure S8.5). Phylogenetic analysis finds that Clade C2 is close to digestive β-glycosidase to cellobiose in *Tenebrio molitor* (Ferreira et al., 2001), implying potential hemicellulase function of this group. In contrast, Clade C3 loses one of the activated sites in most sequences, mainly as proton donor (Jones et al., 2002). GH1 may hydrolyze glycosidic bonds to release non-reducing terminal glucosyl residues from glycosides and oligosaccharides in the downstream of cellulose degradation and thus help herbivorous beetle digest the plant cell wall, along with other upstream plant cell wall-degrading enzymes (PCWDEs), such as GH9, GH28, GH45 and so on (He et al., 2022; McKenna et al., 2019). However, no other PCWDEs are detected in HVIGI, which is different with other herbivorous beetles like weevils and leaf beetles, having a variety of plant cell wall-degrading enzymes (McKenna et al., 2019). Only GH1 exists in the whole ladybird lineage and is putative to be inherited vertically from ancient animals (Chang and Lai, 2018; McKenna et al., 2019). When the pollen-feeding ladybird MDISC eat pollen, GH1 has no respond to the pollen diet, and it is even downregulated in CMONT (Huang et al., 2022). It seems that GH1 in those carnivorous ladybirds have other function instead of plant cell wall digestion. It has been also reported that myrosinase of GH1 can contribute to detoxification of plant toxin (e.g. glucosinolates) in the insects (Beran et al., 2014; He et al., 2022). Although no ladybird clade is close to reported myrosinases, and compared with the myrosinase in *Phyllotreta striolata* (Beran et al., 2014) without motif 6, all ladybird groups have this motif (Figure S8.5), myrosinase can independently originate in different linages (Beran et al., 2014; Ferreira et al., 2001; He et al., 2022). The exact function of GH1 in Epilachnini is needed to be further explored in the future.

-Chitinase

For chitinase, OG0000184 (Group IV chitinases) has two clades (Clade C3 and C5) lost genes of Epilachnini and contains 2/2 and 1/1 downregulated DEGs when MDISC feeds on pollen. Most genes belonging to this OG are highly expressed in the gut. Phylogeny shows that Group IV chitinases in *Tribolium castaneum* and *Tenebrio molitor*, which are highly expressed in the gut and make no contribution to phenotypic development (Genta et al., 2006; Zhu et al., 2008a; Zhu et al., 2008b), are homologous to this OG (Figure S8.6). All of these results show that this OG is digestion-related, and likely to digest the chitin of the insect prey. However, Epilachnini genes are duplicated in Clade C6, though without diet-specific DEGs in HVIGI. Other clades in OG0000184 also contain diet-specific DEGs, including 3/6 upregulated genes in CMONT, 5/7 downregulated genes in MDISC and 1/5 downregulated genes in HVIGI. The group containing diet-specific DEG in HVIGI, Clade C2, contains the only two genes in without downregulation under pollen treatment compared with insect diet treatments in MDISC. This group has chitin binding Peritrophin-A domain in the C-terminal. These genes may be associated with chitin degradation in the gut structure of the ladybirds or in immunity against pathogens containing chitin, as reported in other insects (Arakane and Muthukrishnan, 2010). The absence of this domain in other groups may lead to easiness of inactivation under proteolytic attack and low activity against colloidal chitin, which possibly contributes to digestive activity without peritrophic peritrophic damage (Genta et al., 2006). In addition, Clade C5 of OG0000537 lost Coccinellini genes, but genes in this OG are highly expressed in head or leg, excluding its digestive role.

Furthermore, OG0008949 of CP (carboxypeptidase D-like), OG0009358 and OG0009447 are emergent in Coccinellidae, while OG0011191 of CAT (procathepsin L-like) is emergent in Coccinellini. OG0014174 (Kallikrein 1-peptidase b1), OG0015067, OG0030813 (Trypsin-2) of SP and OG0047347 of CAT are emergent in Epilachnini. OG0000774 (lipase-like) and OG00009300 ((Lyso)-N-acylphosphatidylethanolamine lipase-like) of LIP are emergent in Coccinellidae and OG0012264 of LIP (Abhydrolase domain-containing protein 2) is lost in Coccinellidae, indicating fast evolution of LIPs in the ladybirds. But these OGs are not gut-specific or diet-specific.

### 8.2.2 Candidate genes encoding nutrient transporters

Eleven, 75, six, six, fifteen, seven and eight OGs containing at least 30 gene members are identified as NATs, GLUTs, SGLTs, FABPs, FATPs, NPCs and SCPs, respectively.

-FABP

Among these OGs, OG0000283 of FABP, mainly including apolipoprotein D-like genes, expands in both Coccinellidae and Epilachnini (Figure S8.7). Clade C5 in the gene tree mainly contribute to the expansion in Coccinellidae and Epilachnini, with a gene duplication event in Epilachnini, while Clade C4 completely consists of genes in Epilachnini species (Figure S8.8). The heatmap of tissue expression patterns shows high expression of this OG in gut (Figure S8.9). There are large amounts of diet-specific DEGs in OG0000283, including 3/12 DEGs (mainly upregulated) in CMONT and 9/27 downregulated DEGs in HVIGI under non-optimal diet treatments (Figure S8.10).

-GLUT

Two OGs of GLUT, including OG0000068 and OG0000088, are detected as significantly expanding OGs in both Coccinellidae and Epilachnini. The expansion in OG0000068 mainly occur in Clade C5, while the expansion in OG0000088, which are mostly annotated as facilitated trehalose transporter Tret1-like genes, mainly occur in Clade C3 (Figure S8.8). A gene duplication event in Coccinellidae occur in OG0000068, leading to Clade C3 and C4, and Epilachnini genes are duplicated in Clade C5. Most genes of OG0000088 are specifically expressed in the gut, while most genes in Clade C5 of OG0000068 are highly expressed in the thorax or abdomen (Figure S8.9). OG0000068 contain relatively less diet-specific DEGs, including 5/29 genes in CMONT and 4/31 (3 downregulated and 1 upregulated) genes in HVIGI when feeding on non-optimal diets, while OG0000088 contain more diet-specific DEGs, including 8/12 genes (mainly upregulated) in CMONT and 8/21 downregulated DEGs (Figure S8.10). Besides, OG0000180 (facilitated trehalose transporter Tret1-like), OG0000204 (synaptic vesicle glycoprotein 2-like), OG0000260 (facilitated trehalose transporter Tret1-like), OG0000315 (facilitated trehalose transporter Tret1-like), OG0000375 (solute carrier family 22), OG0000398 (feline leukemia virus subgroup C receptor-related protein-like), OG0000574 (facilitated trehalose transporter Tret1-like), OG0000803 (facilitated trehalose transporter Tret1-like) of GLUT also have Epilachnini-duplicated clades, while OG0000180, OG0000315, OG0002318 (organic cation transporter protein-like) of GLUT have duplicated clades in Coccinellini. Most these OGs are mainly gut-specific and contain diet-specific DEGs.

-FATP

Two gene duplication events in Epilachnini take place in Clade C2 and C4 of OG0000118 of FATP, with 3/10 downregulated DEGs in HVIGI in the duplicated clades. Clade C1 of OG0000118 also has a clade containing only genes in carnivorous ladybird, with 1/1 diet-specific DEGs in HAXYR. Most genes in OG0000118 are highly expressed in gut or abdomen. Further, OG0000295 and OG0000391 (4-coumarate--CoA ligase 1-like) have an Epilachnini-specific gene duplication respectively, but few genes in them are gut-specifically expressed and diet specific DEGs in HVIGI.

Additionally, Clade C1 and C2 in OG0000989 of GLUT (facilitated trehalose transporter Tret1-like) lost all genes of Coccinellini, with high expression in antenna or head in CMONT. OG0008542 (facilitated trehalose transporter Tret1-like) and OG0008954 (sialin-like) of GLUT are emergent in Coccinellidae, and OG0008906 of GLUT only exists in carnivorous ladybirds, while OG0010502 of GLUT (monocarboxylate transporter) and OG0010643 of NAT (sodium-dependent nutrient amino acid transporter 1-like) are lost in Coccinellidae. OG0011948, OG0019650, OG0021245 and OG0026466 of FABP are emergent in Epilachnini. Among these OGs, only OG0008906 and OG0011948 mainly contain gut-specific genes and downregulated DEGs in non-optimal diet-fed ladybirds, which indicates important roles of GLUT and FABP in the evolution of ladybird feeding habits.

Combined with the expression patterns of different tissues and diets, OG0000118 (FATP), OG0000283 (FABP) and OG0000088 (GLUT) are most likely to be related to the evolution of feeding habits in the ladybirds. FATP, FABP and GLUT are the transporters of free fatty acids and monosaccharide respectively. FATP can also be related to fat storage in the ladybirds (Xiang et al., 2021), but the related OG does not experience evolution events (Table S8.2). The expansion or duplication of FATP, FABP and GLUT in Coccinellidae or Epilachnini is possibly associated with adaptation to new nutrition in their prey or plant diets, compared with their original diet fungi or insects, respectively, such as some glucans, melezitose, linoleic acid, palmitoleic, oleic and linolenic acids (Lundgren, 2009). In addition, fatty acid is also one of the main components in the wax of the scale insects (Tong et al., 2022). When the ladybird ancestor adapts to the scale insects, the wax shell of the prey may need to be managed and drive the evolution of FATP and FABP.

### 8.2.3 Other candidate genes related to digestion

-CBPD, LMPO

Six CBPD OGs with gene members >= 30 are identified, among which OG0000227 expands in Epilachnini and contract in Coccinellini (Figure S8.11). The expansion in Epilachnini is mainly in Clade C2 in the gene tree, with at least two gene duplication events in Epilachnini (Figure S8.12). The genes in this OG are mainly expressed in the gut, followed by thorax and abdomen (Figure S8.13). In HVIGI, 9/11 downregulated DEGs are found when they feed on sugar water compared with plant leaves (Figure S8.14). In addition, three LMPO OGs with total genes >= 30 are found, with OG0000406 lost in Epilachnini (Figure S8.11). Most genes in OG0000406 are highly expressed in the gut and some genes are highly expressed in the leg or antenna (Figure S8.13). Diet-specific DEGs of the carnivorous ladybirds are found in this OG, including 5/6 genes in CMONT and 2/4 genes in MDISC (Figure S8.14). OG0000406 also contains at least one respective upregulated DEG responding to Sternorrhyncha diets in CMONT (5/6), MDISC (2/4) and PJAPO (1/9), and 2/4 downregulated DEGs in pollen-fed MDISC compared with those fed by insect diets (Figure S8.15). Compared with other beetles, approximately half of ladybird genes in OG0000406 have a specific motif 9. Additionally, OG0014569 of CBPD is lost in Coccinellidae.

CBPD is likely to expressed in the insect midgut and associated with structural and functional integrity of peritrophic membranes, and thus can influence the digestion ability (Jasrapuria et al., 2010; Rodriguez-de la Noval et al., 2019; Tetreau et al., 2015), with one OG (OG0000227) in our study which has larger gene counts in not only in Epilachnini but also in other herbivorous beetles (e.g. leaf beetles, long-horned beetle and emerald ash borer) than carnivorous beetles (including other ladybirds) (Figure S8.16) and contains downregulated DEGs in diet treatment comparison in HVIGI, probably related to digestion of plant tissues or components. On the contrary, one LPMO OG (OG0000406) lost its gene members in Epilachnini. LPMO is mainly related to cellulose degradation, but it can also assist chitin degradation during development in insects, especially for development of peritrophic matrix (Qu et al., 2022b; Qu et al., 2022c). The loss of the LPMO genes seems to be related to chitin degradation and development instead of digestion of the plant diet, which is different with the role of cellulose digestion of some LPMO genes in *Thermobia domestica* (Sabbadin et al., 2018). The gene members downregulated under pollen diet treatment also indicate a potential function of direct digestion to chitin in the insect prey. But more experimental evidence may be needed.

## 8.3 Summary

The OGs of nine digestive enzymes, seven nutrient transporters and two other digestion-related genes were explored. Summarizing the information of evolution events and expression patterns, we found at least six OG related to evolution of feeding habits in the ladybirds, including OG0000065 of CAT, OG0000184 of chitinase, OG0000283 of FABP, OG0000088 of GLUT, OG0000227 of CBPD and OG0000406 of LMPO. Additionally, OG0000196 of CP, OG0000380 and OG0003251 of ASP, OG0000110 of GLC and OG0000118 of FATP are duplicated in clades of Epilachnini genes and have leaf diet-specific DEGs.

Table S8.2 The reported function of the candidate digestive genes in the ladybirds.

| Reported gene name | Gene ID | Ortholog group | Gene family | High expression in qPCR | Functional verification | Reference |
| --- | --- | --- | --- | --- | --- | --- |
| CsACSL | CSEPT.138 | OG0007680 | FATP | diapause preparation phase | fat storage during early diapause | Xiang et al. (2021) |


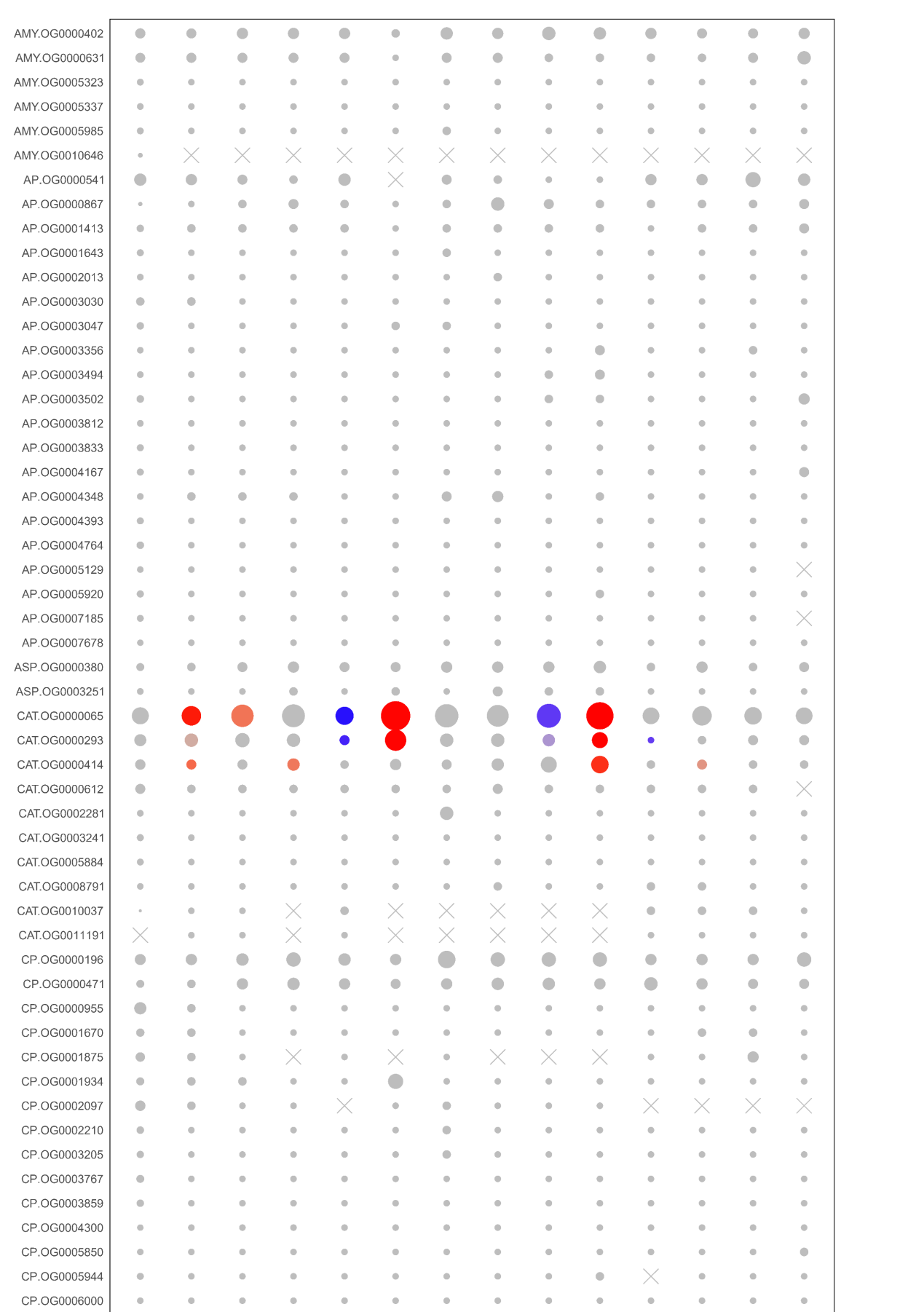


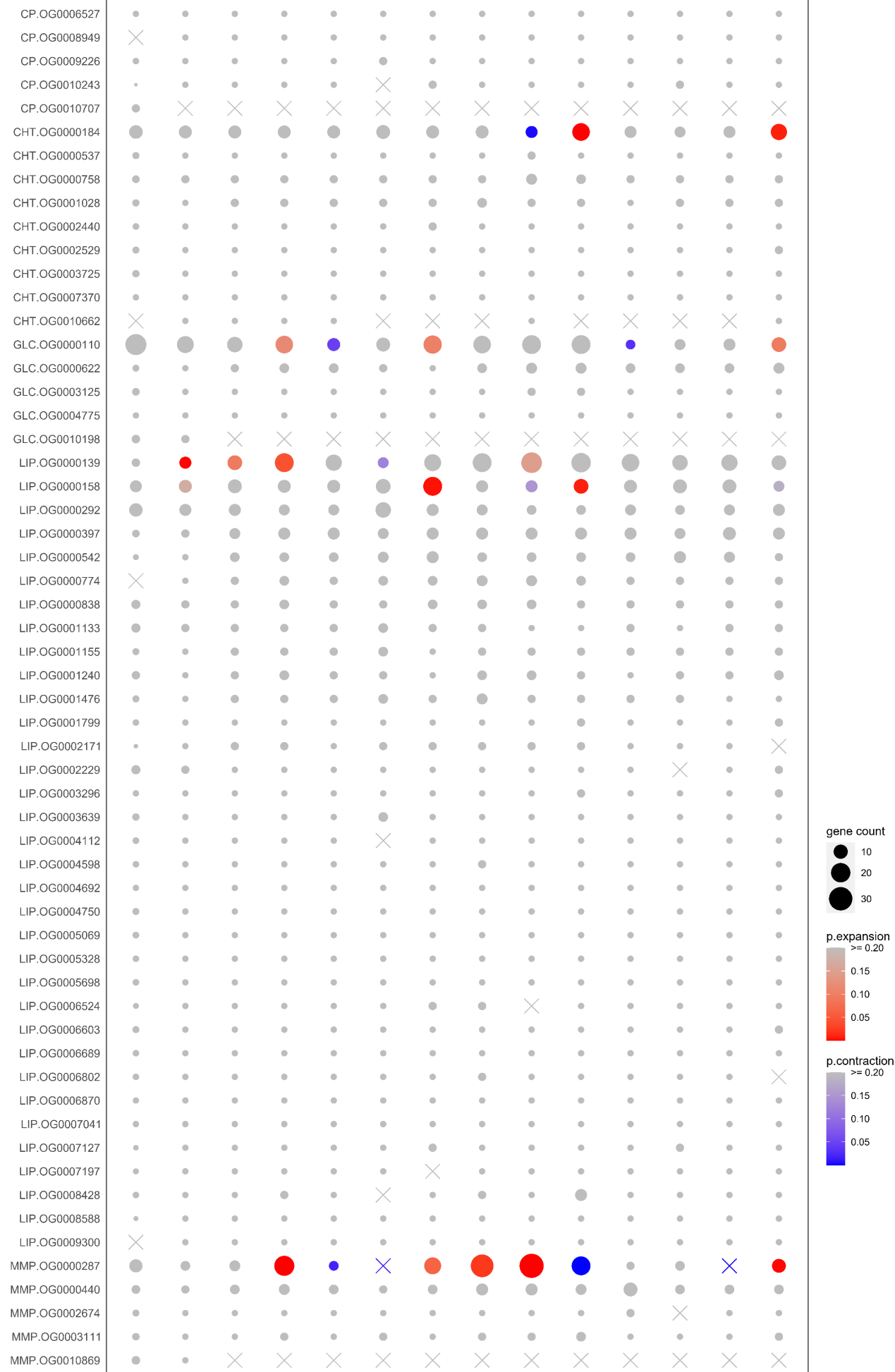


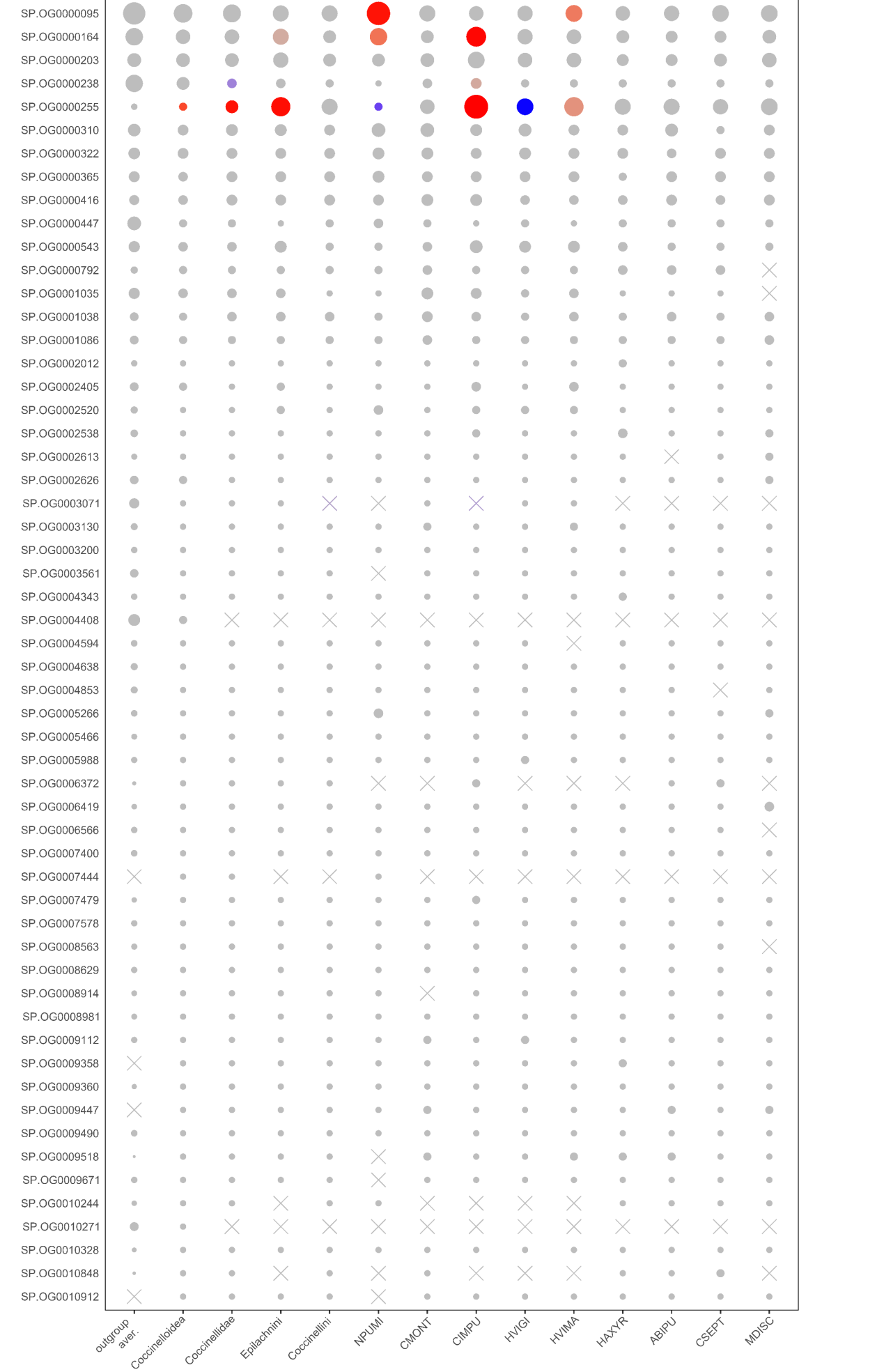


Figure S8.1 Comparison of gene counts in ortholog groups (OGs) of digestive enzymes in the ten ladybird genomes. Only OGs with total genes >= 30 are shown. Gene counts at the nodes of Coccinelloidea, Coccinellidae, Epilachnini and Coccinellini are reconstructed by CAFE. A high-quality figure can be downloaded from https://github.com/huangyh45/ladybird-genomes-supplementary-figures.


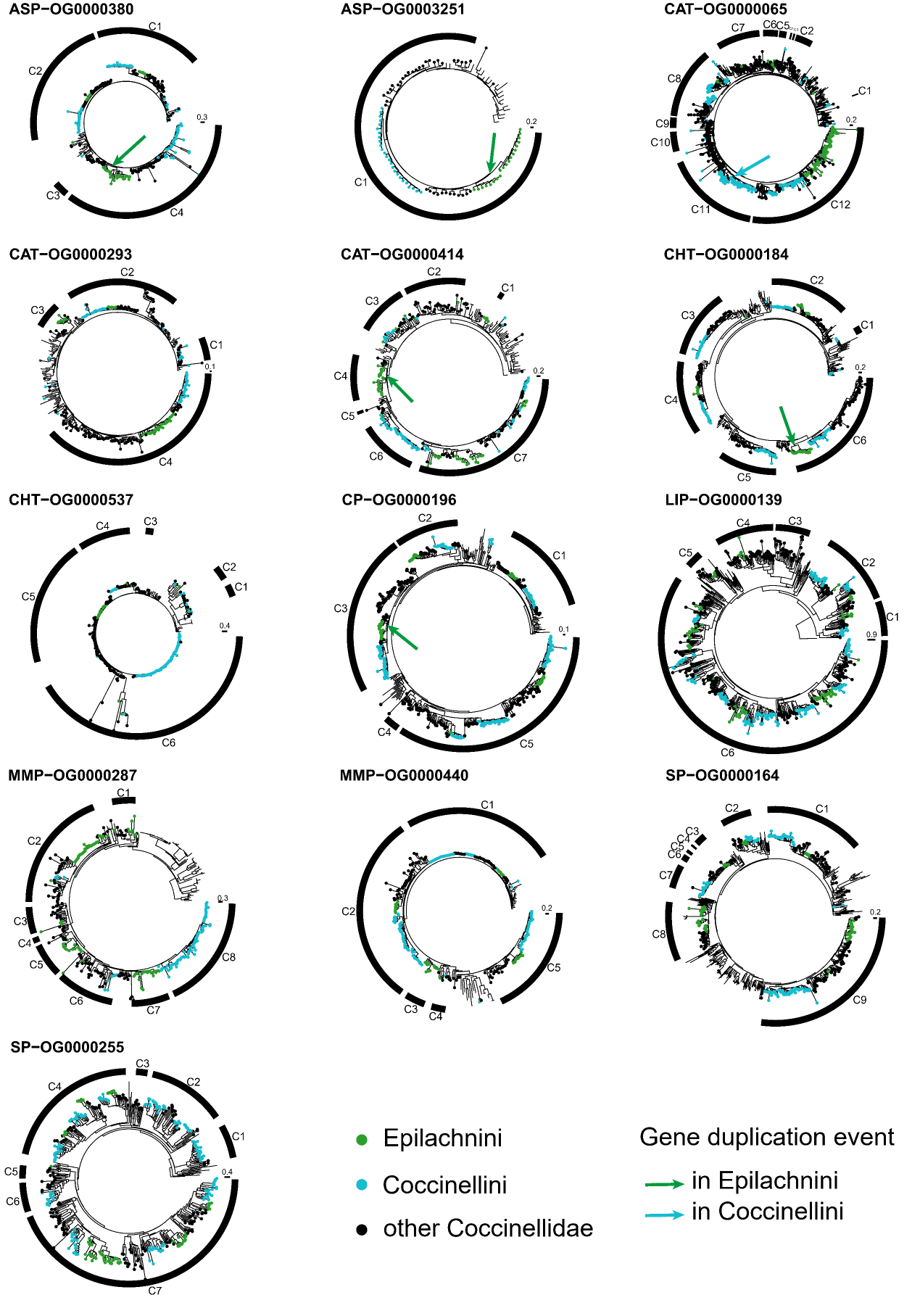


Figure S8.2 Gene trees of ortholog groups (OGs) of digestive enzymes. The genes in the ladybirds are marked in color and the others without circles are the genes in the outgroup beetles. A high-quality figure can be downloaded from https://github.com/huangyh45/ladybird-genomes-supplementary-figures.


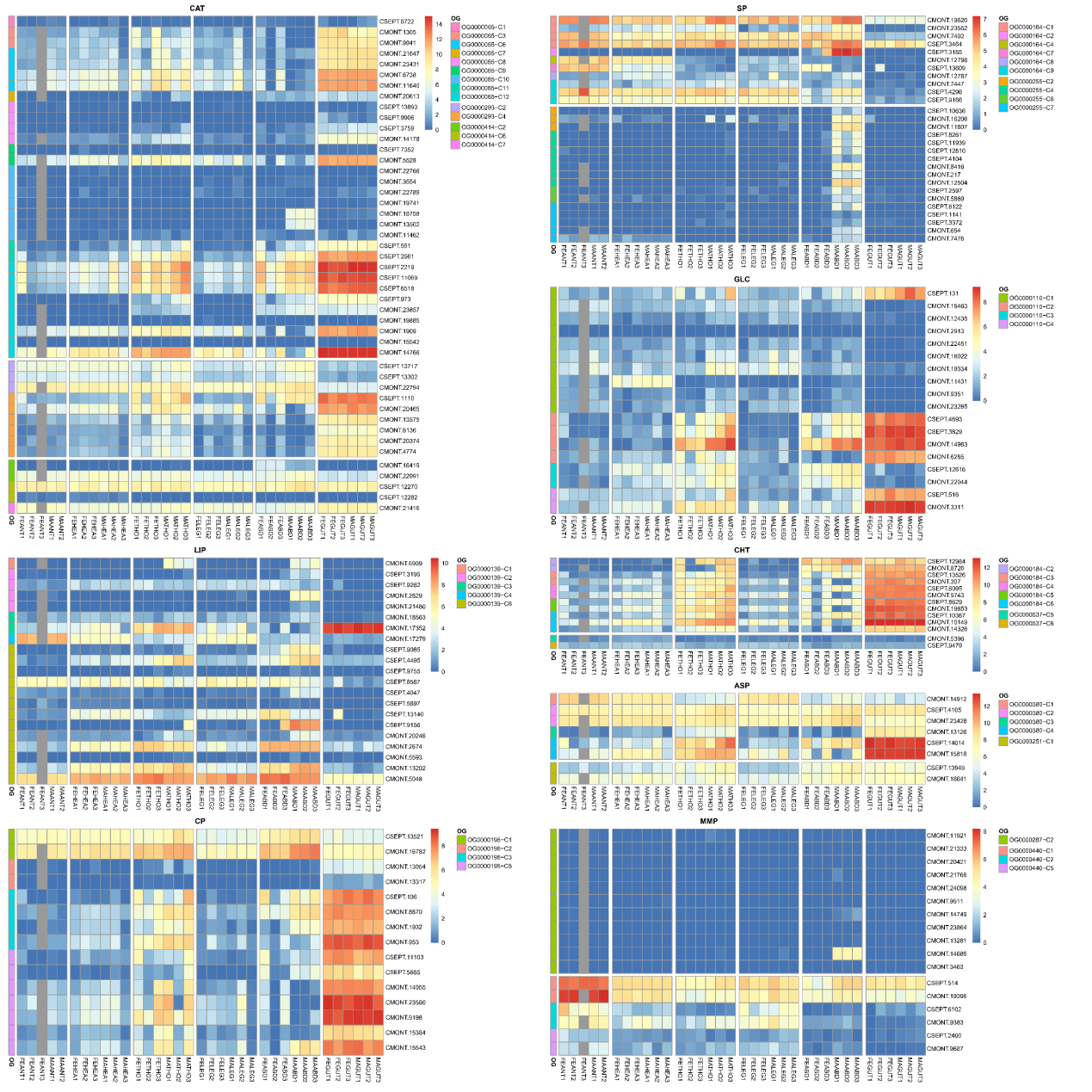


Figure S8.3 Expression patterns of different tissue of each group in ortholog groups (OGs) of digestive enzymes. Abbreviation in the sample names: FE: female adult, MA: male adult, ANT: antenna, HEA: head, THO: thorax, LEG: leg, ABD: abdomen, GUT: gut. A high-quality figure can be downloaded from https://github.com/huangyh45/ladybird-genomes-supplementary-figures.


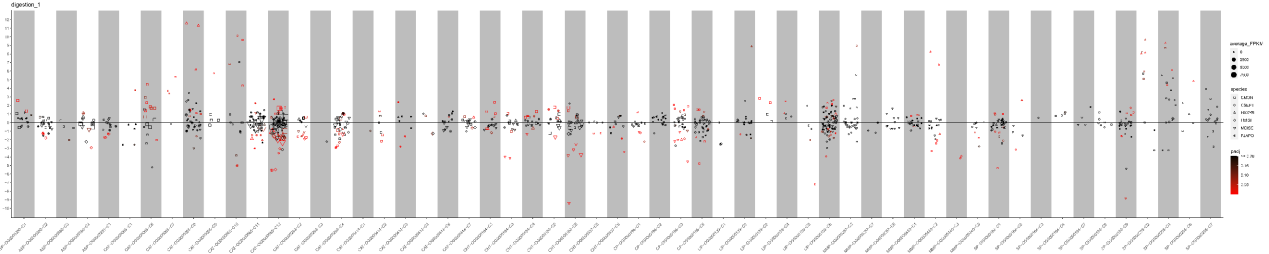


Figure S8.4 Expression patterns under different diet treatments of each group in ortholog groups (OGs) of digestive enzymes. The vertical axis represents the log2(fold change) values. The positive values on the vertical axis represent upregulation when feeding on the optimal diets, while the negative values represent upregulation when feeding on the non-optimal diets. A high-quality figure can be downloaded from https://github.com/huangyh45/ladybird-genomes-supplementary-figures.


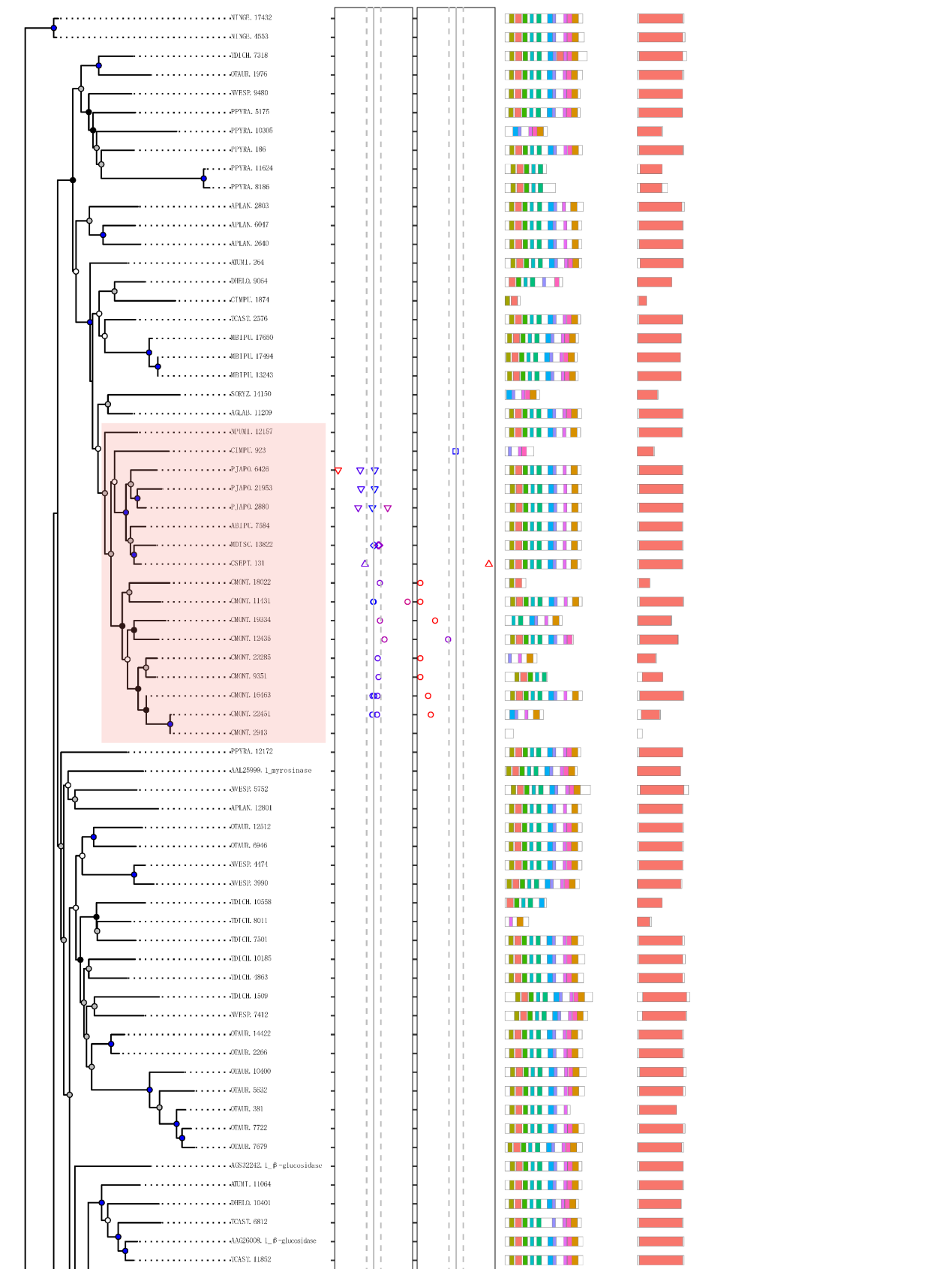


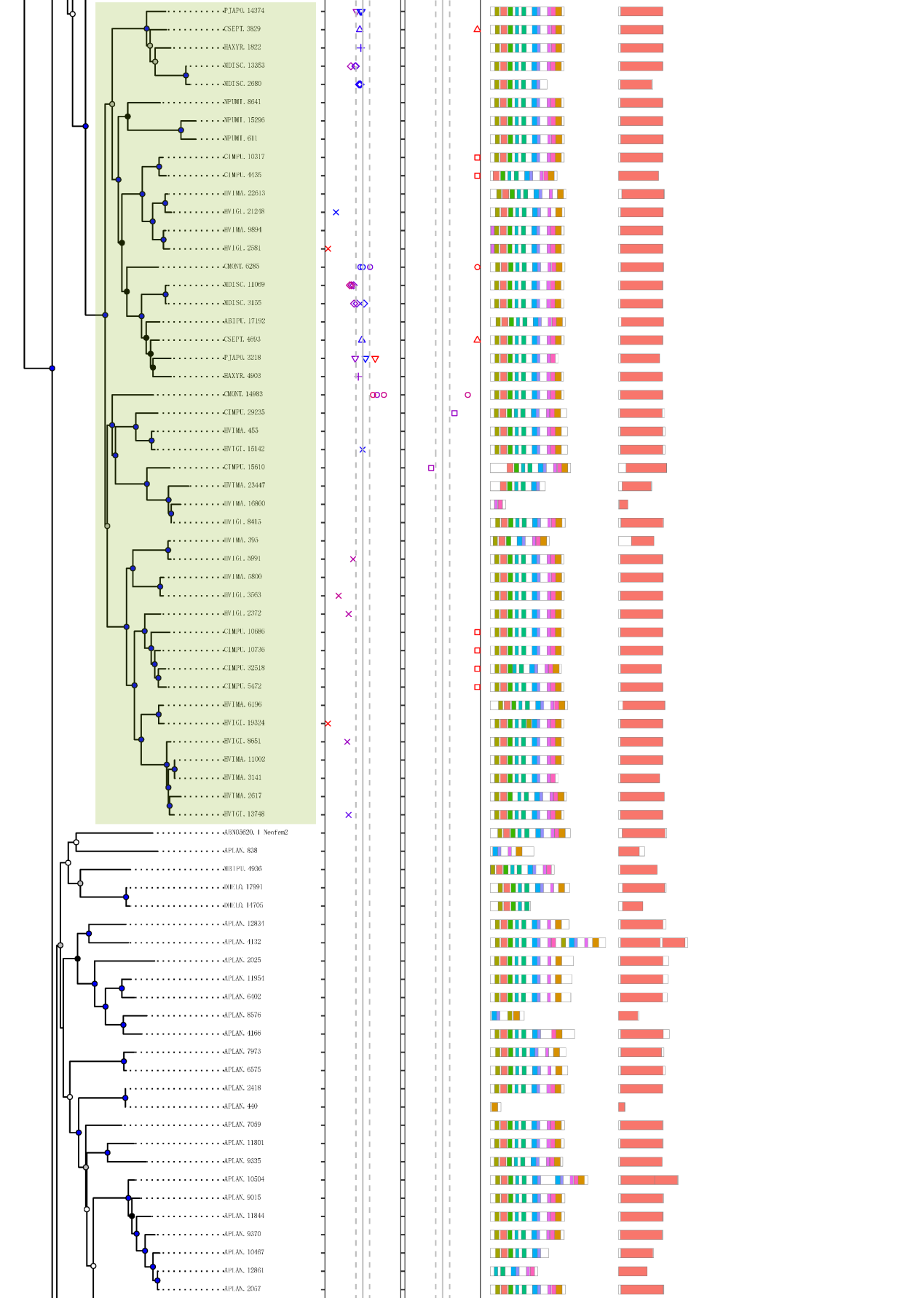


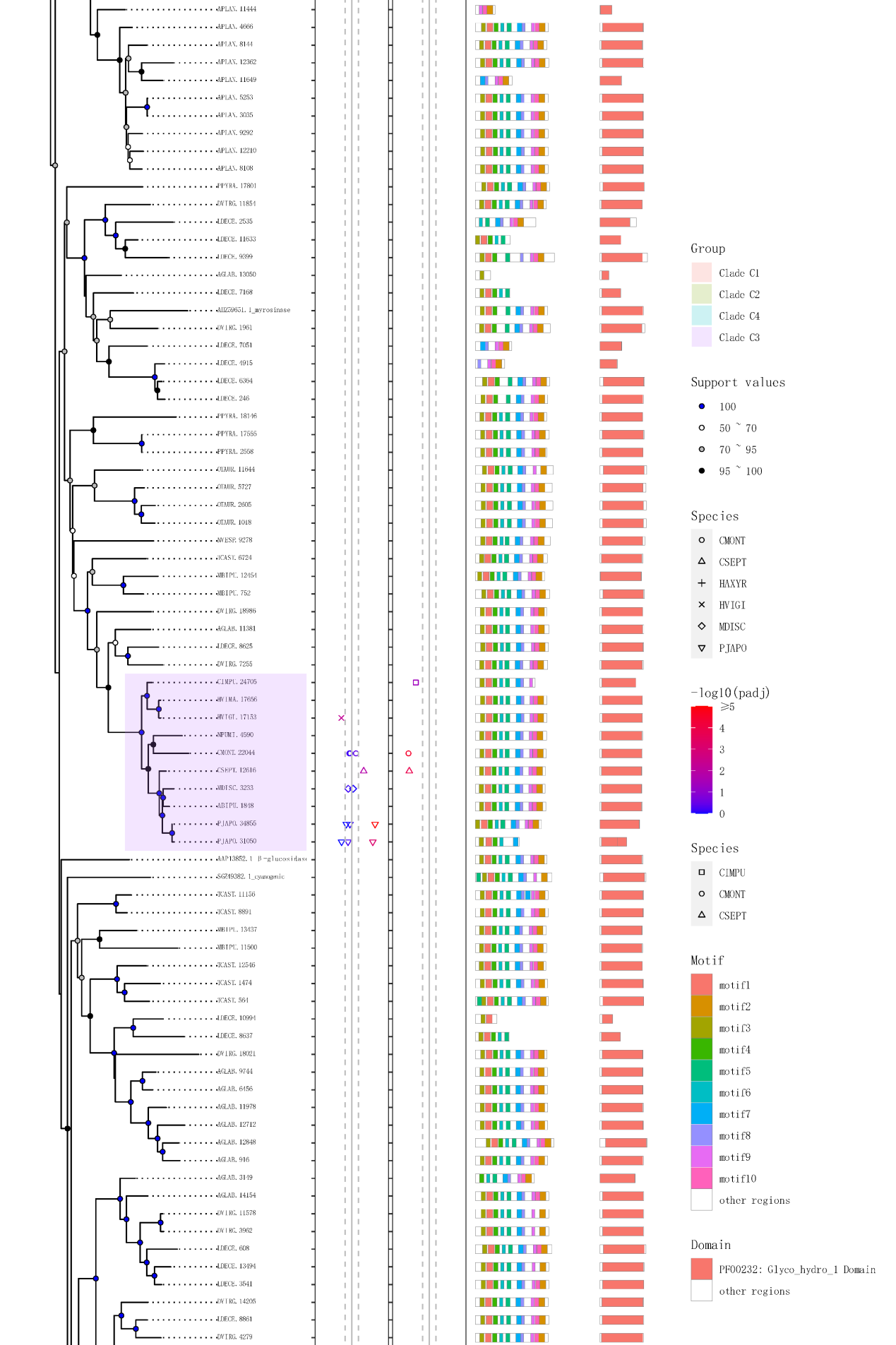


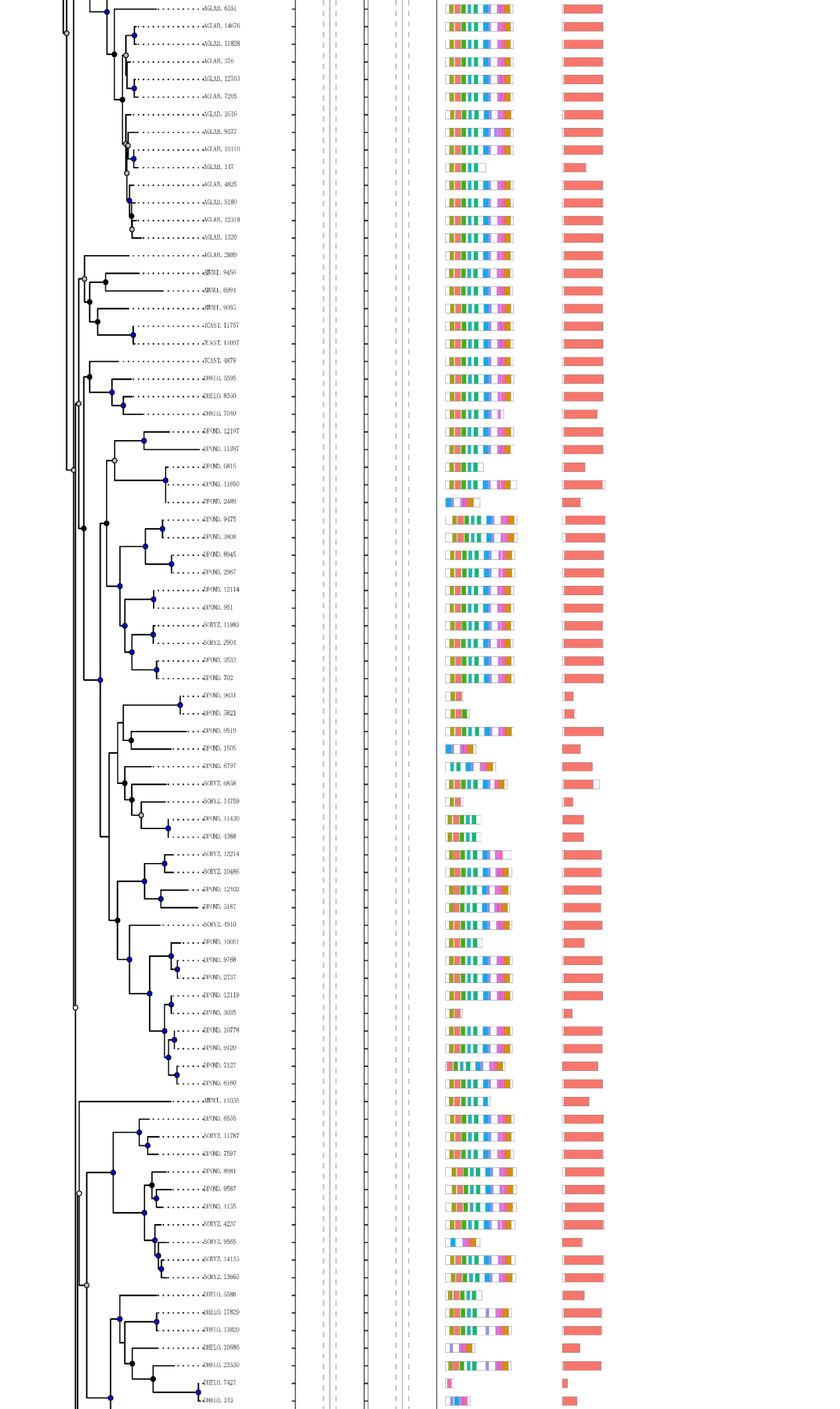


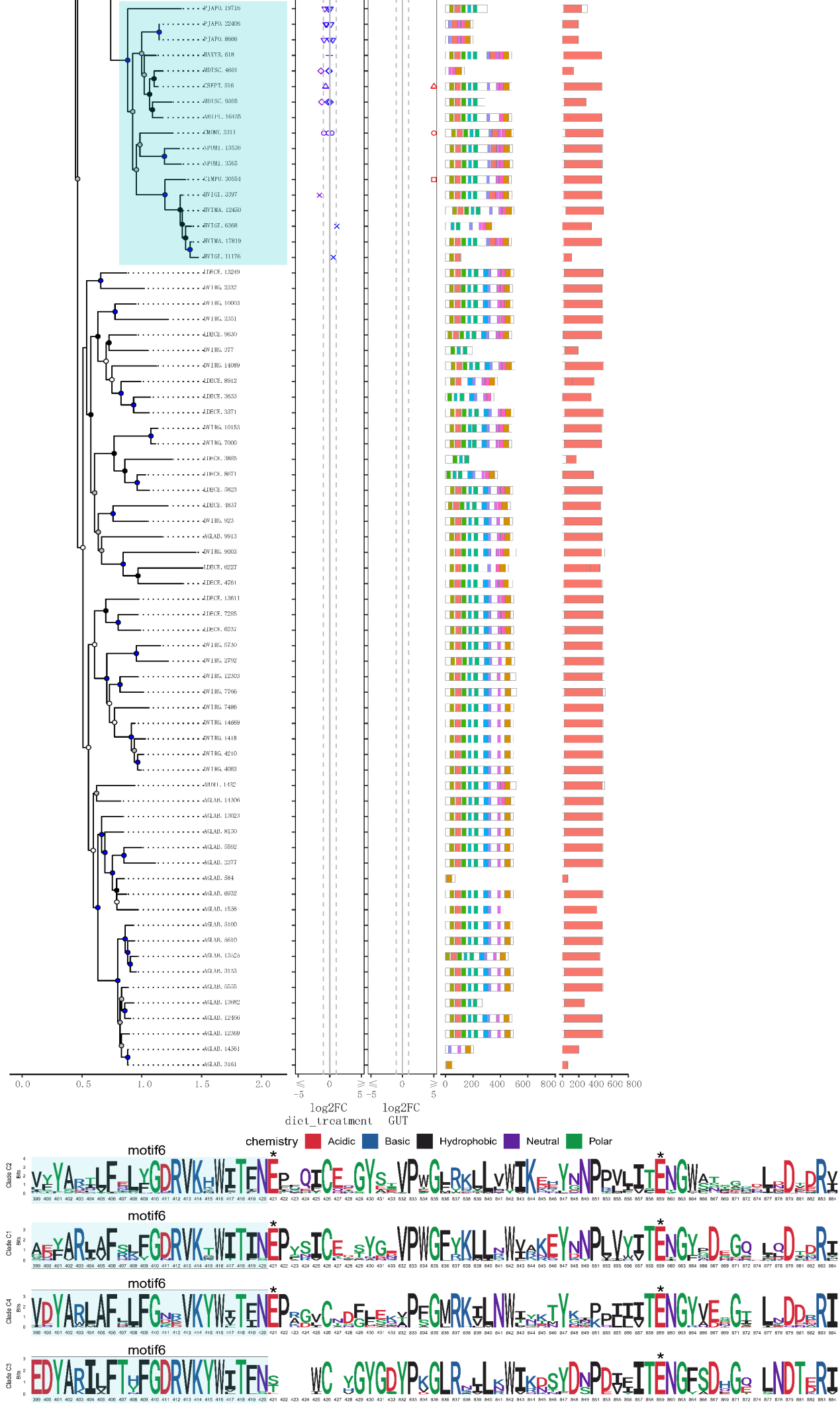


Figure S8.5 Phylogeny and sequence composition of OG0000110 of GLC. The asterisks represent active sites in the domains. A high-quality figure can be downloaded from https://github.com/huangyh45/ladybird-genomes-supplementary-figures.


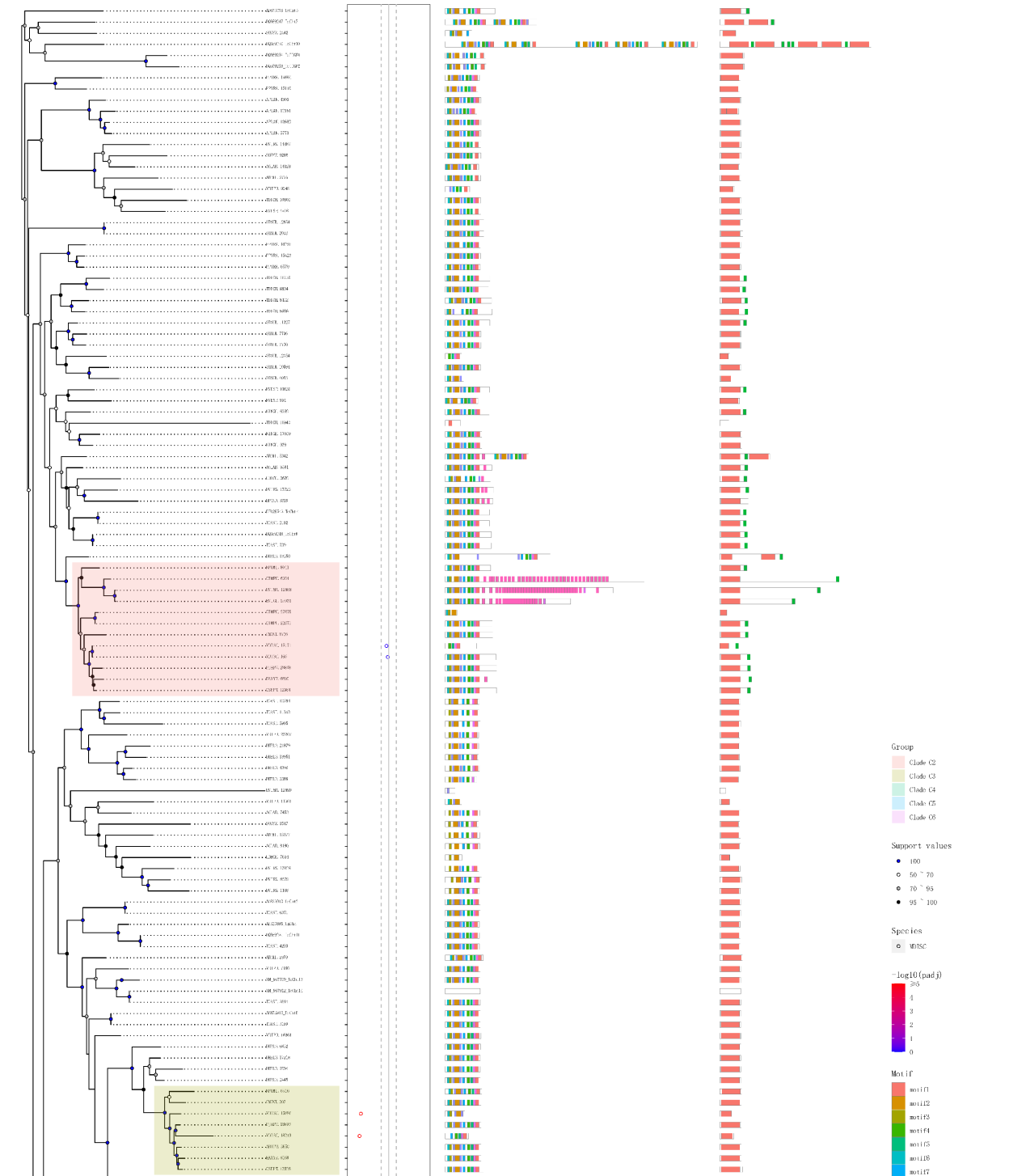


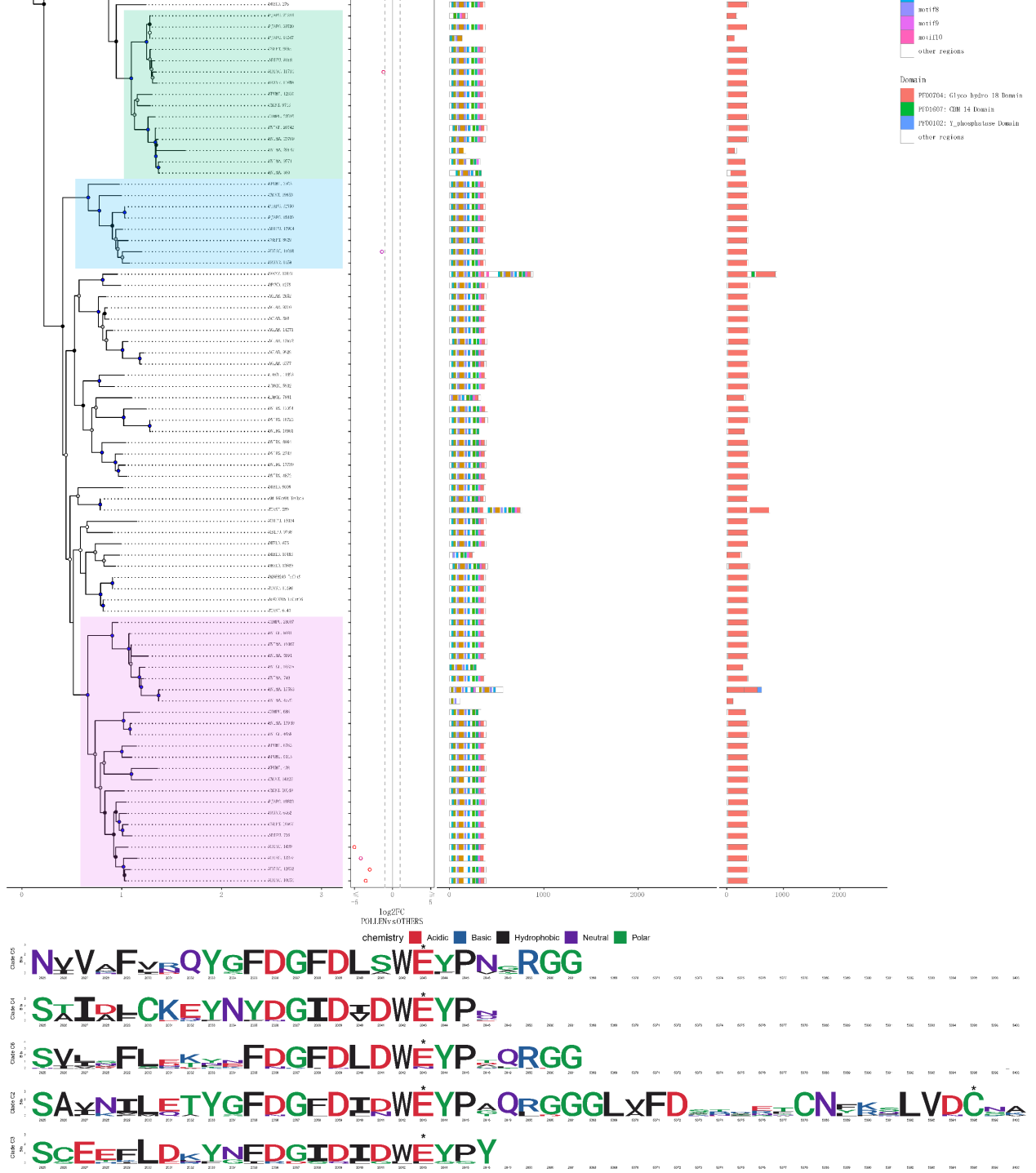


Figure S8.6 Phylogeny and sequence composition of OG0000184 of chitinase. The asterisks represent active sites in the domains. A high-quality figure can be downloaded from https://github.com/huangyh45/ladybird-genomes-supplementary-figures.


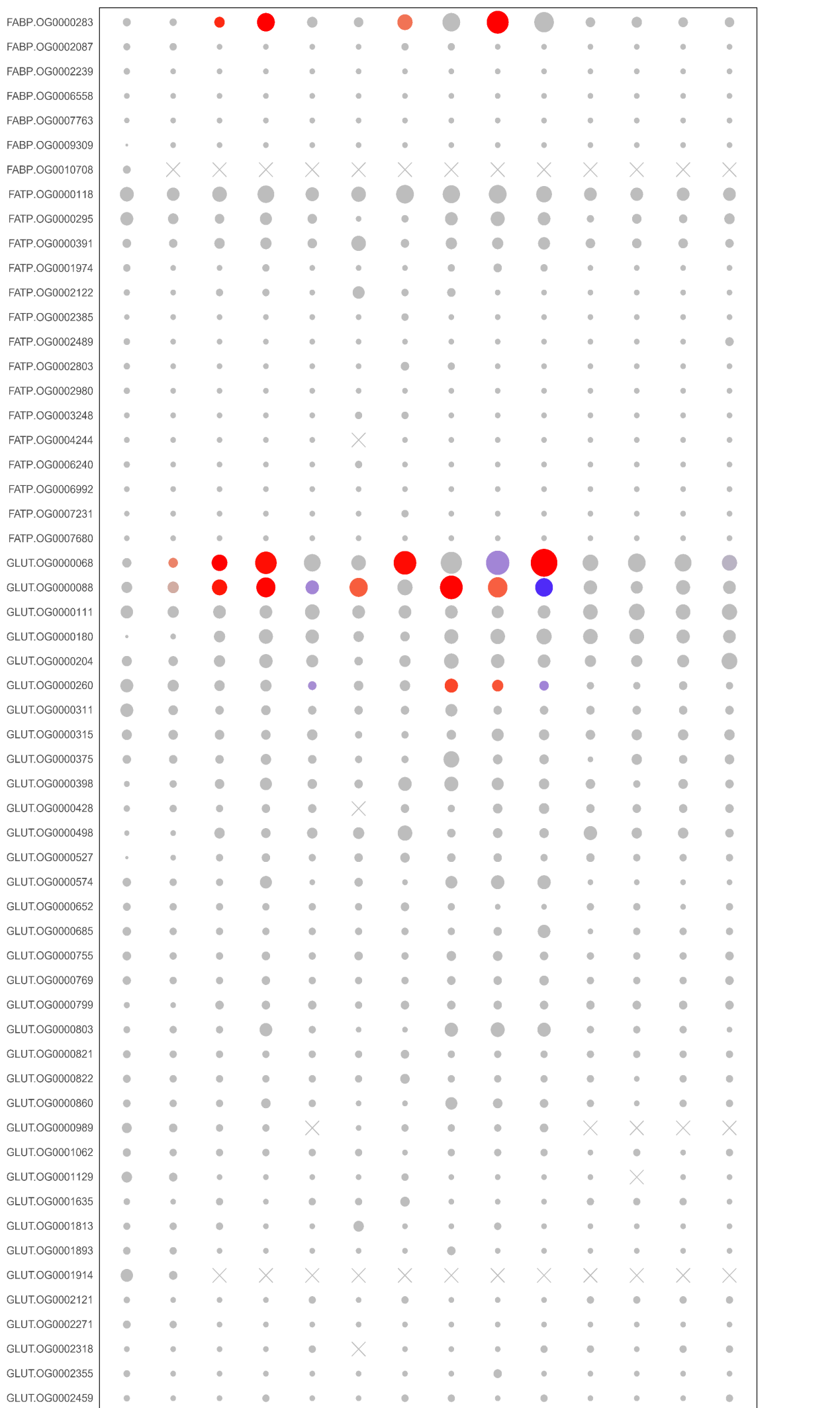


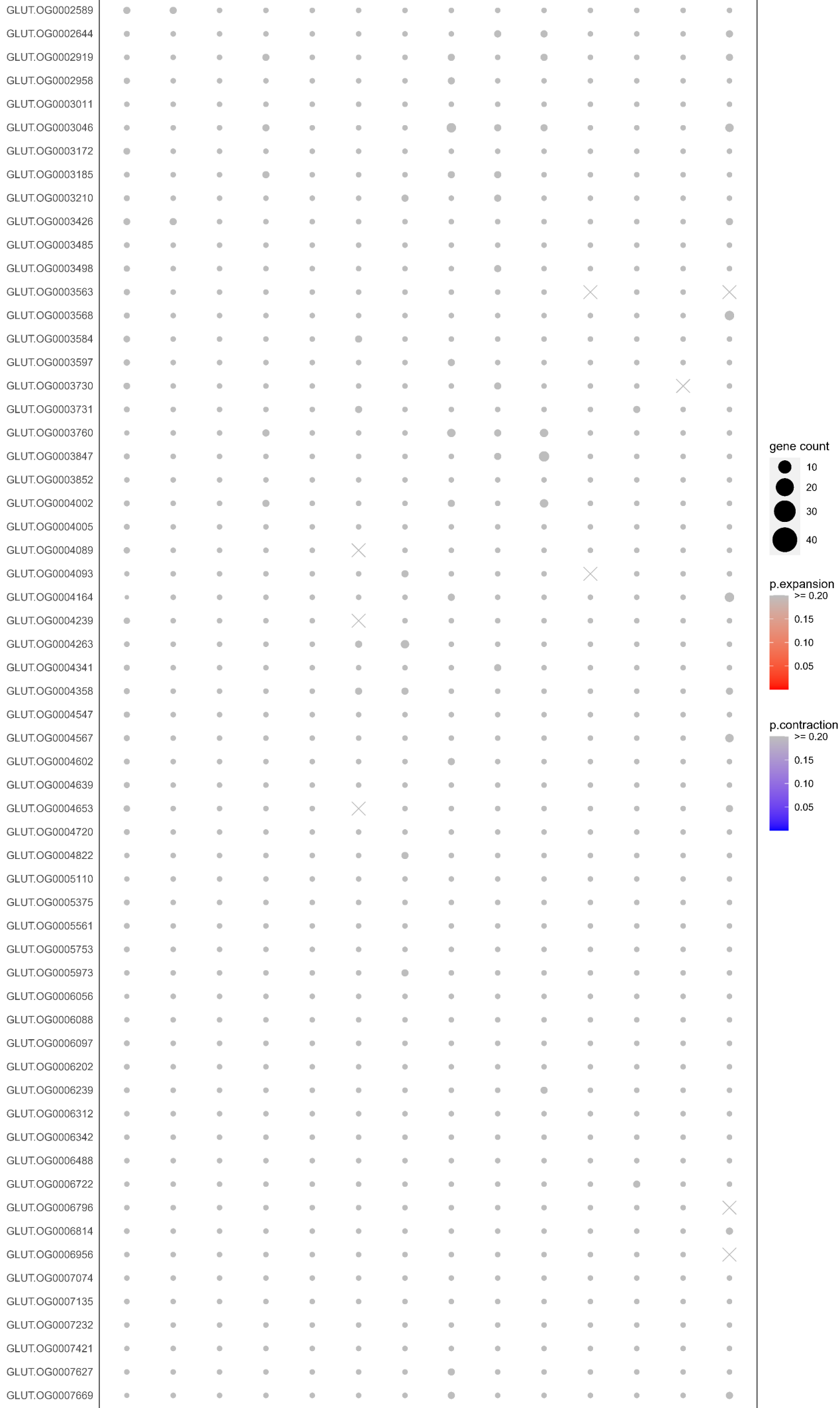


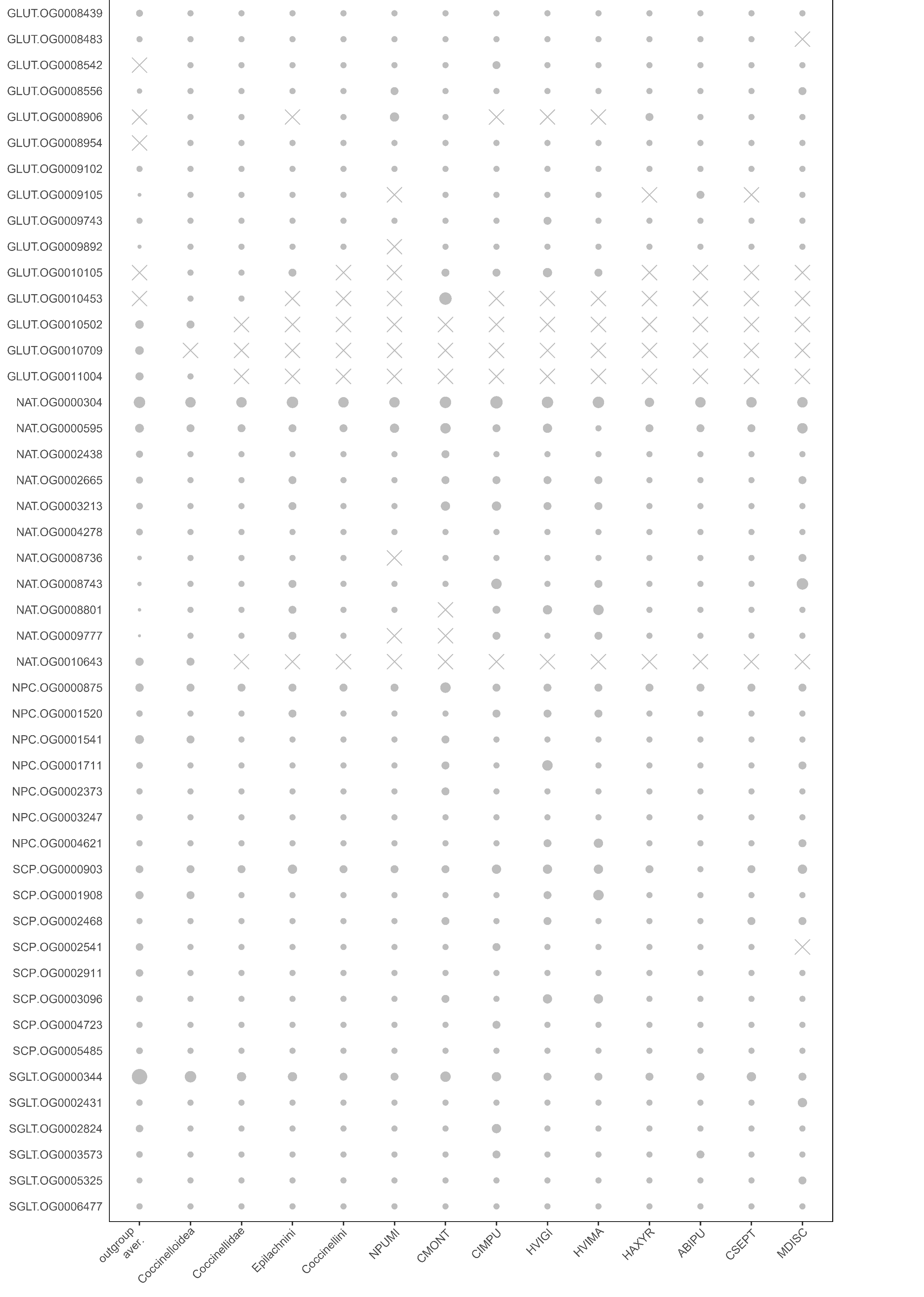


Figure S8.7 Comparison of gene counts in ortholog groups (OGs) of nutrient transporters in the ten ladybird genomes. Only OGs with total genes >= 30 are shown. Gene counts at the nodes of Coccinelloidea, Coccinellidae, Epilachnini and Coccinellini are reconstructed by CAFE. A high-quality figure can be downloaded from https://github.com/huangyh45/ladybird-genomes-supplementary-figures.


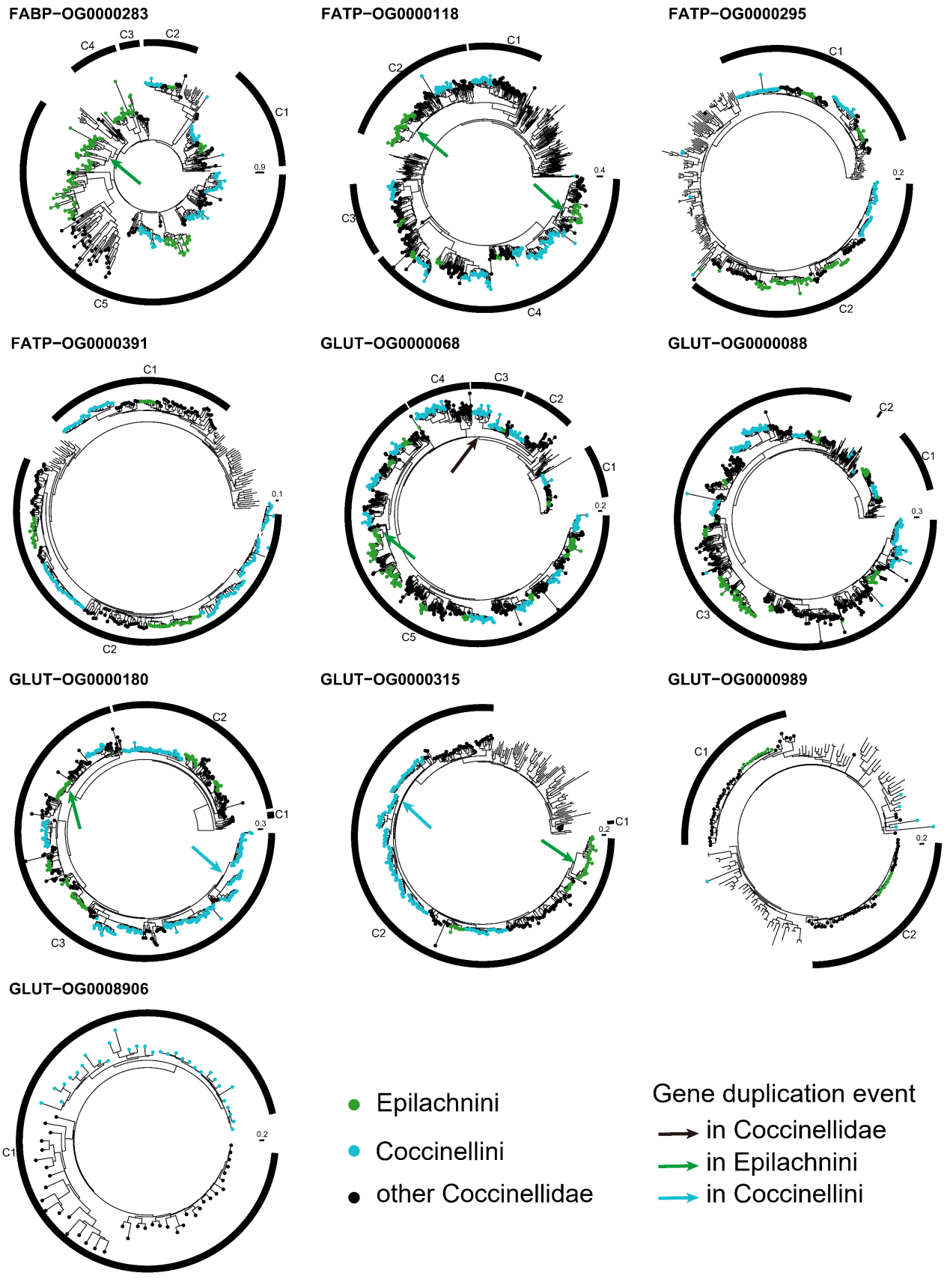


Figure S8.8 Gene trees of ortholog groups (OGs) of nutrient transporters. The genes in the ladybirds are marked in color and the others without circles are the genes in the outgroup beetles. A high-quality figure can be downloaded from https://github.com/huangyh45/ladybird-genomes-supplementary-figures.


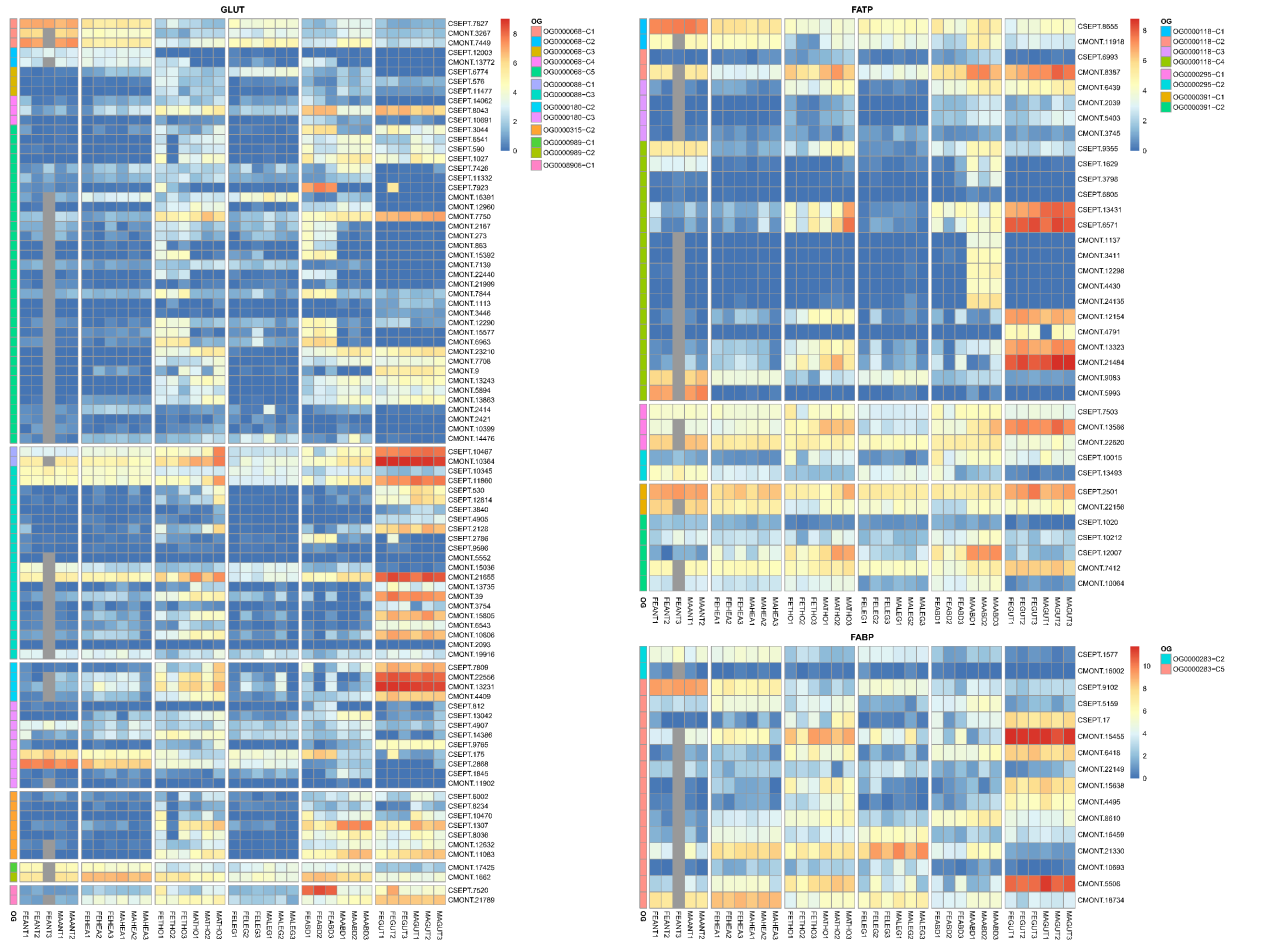


Figure S8.9 Expression patterns of different tissue of each group in ortholog groups (OGs) of nutrient transporters. Abbreviation in the sample names: FE: female adult, MA: male adult, ANT: antenna, HEA: head, THO: thorax, LEG: leg, ABD: abdomen, GUT: gut. A high-quality figure can be downloaded from https://github.com/huangyh45/ladybird-genomes-supplementary-figures.


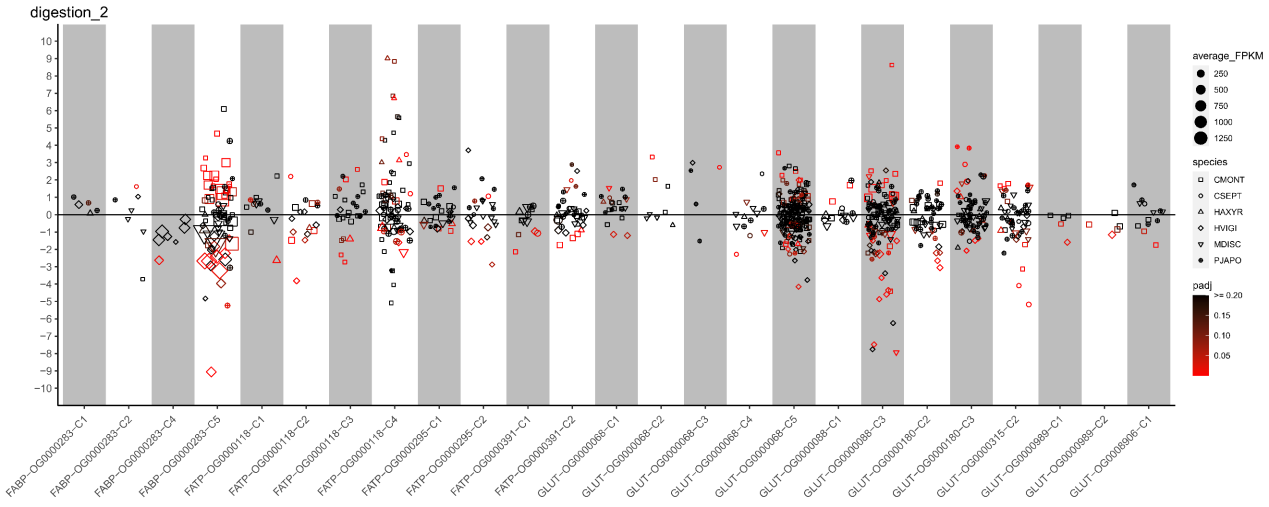


Figure S8.10 Expression patterns under different diet treatments of each group in ortholog groups (OGs) of nutrient transporters. The vertical axis represents the log2(fold change) values. The positive values on the vertical axis represent upregulation when feeding on the optimal diets, while the negative values represent upregulation when feeding on the non-optimal diets. A high-quality figure can be downloaded from https://github.com/huangyh45/ladybird-genomes-supplementary-figures.


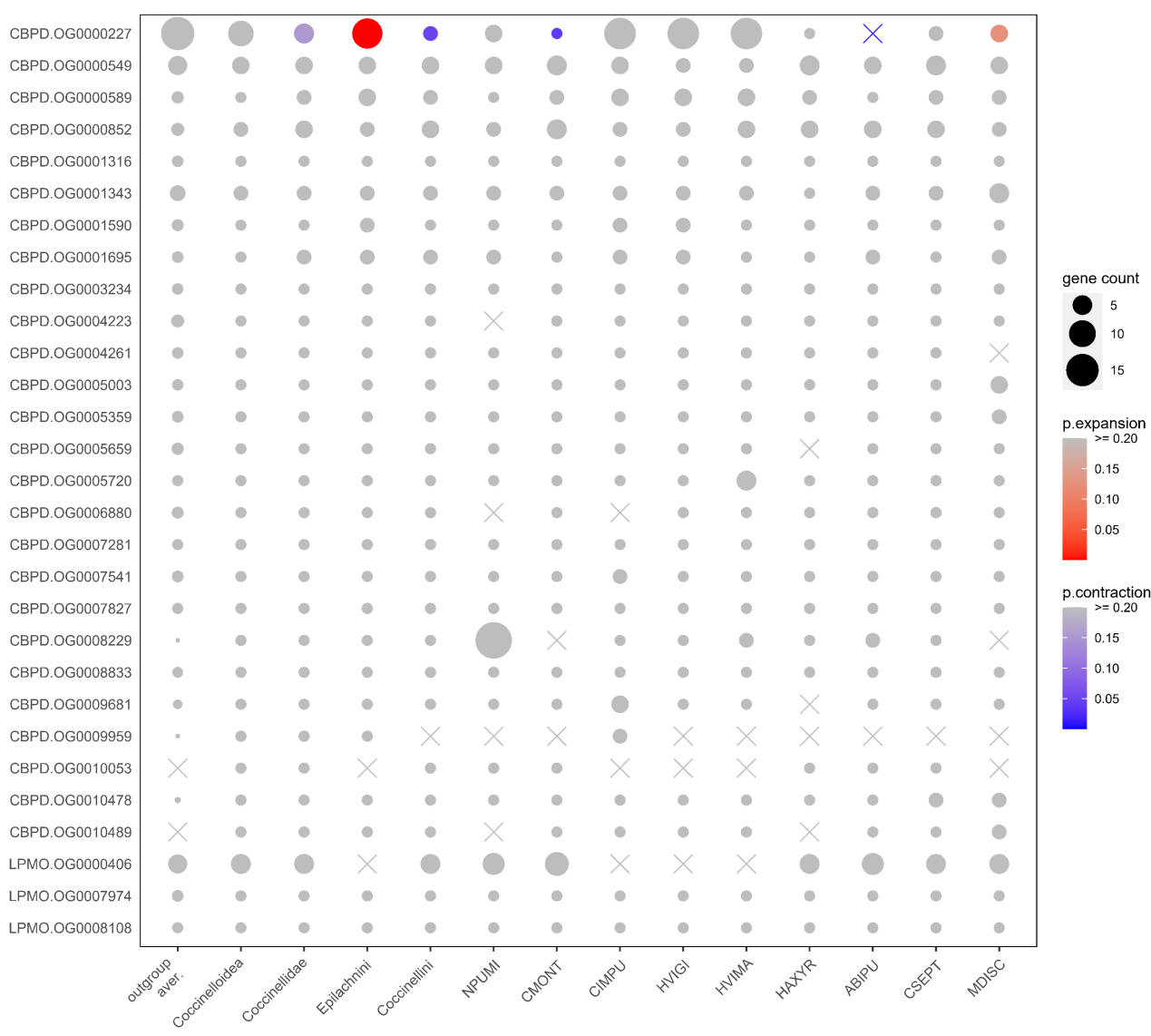


Figure S8.11 Comparison of gene counts in ortholog groups (OGs) of CBPDs and LMPOs in the ten ladybird genomes. Only OGs with total genes >= 30 are shown. Gene counts at the nodes of Coccinelloidea, Coccinellidae, Epilachnini and Coccinellini are reconstructed by CAFE. A high-quality figure can be downloaded from https://github.com/huangyh45/ladybird-genomes-supplementary-figures.


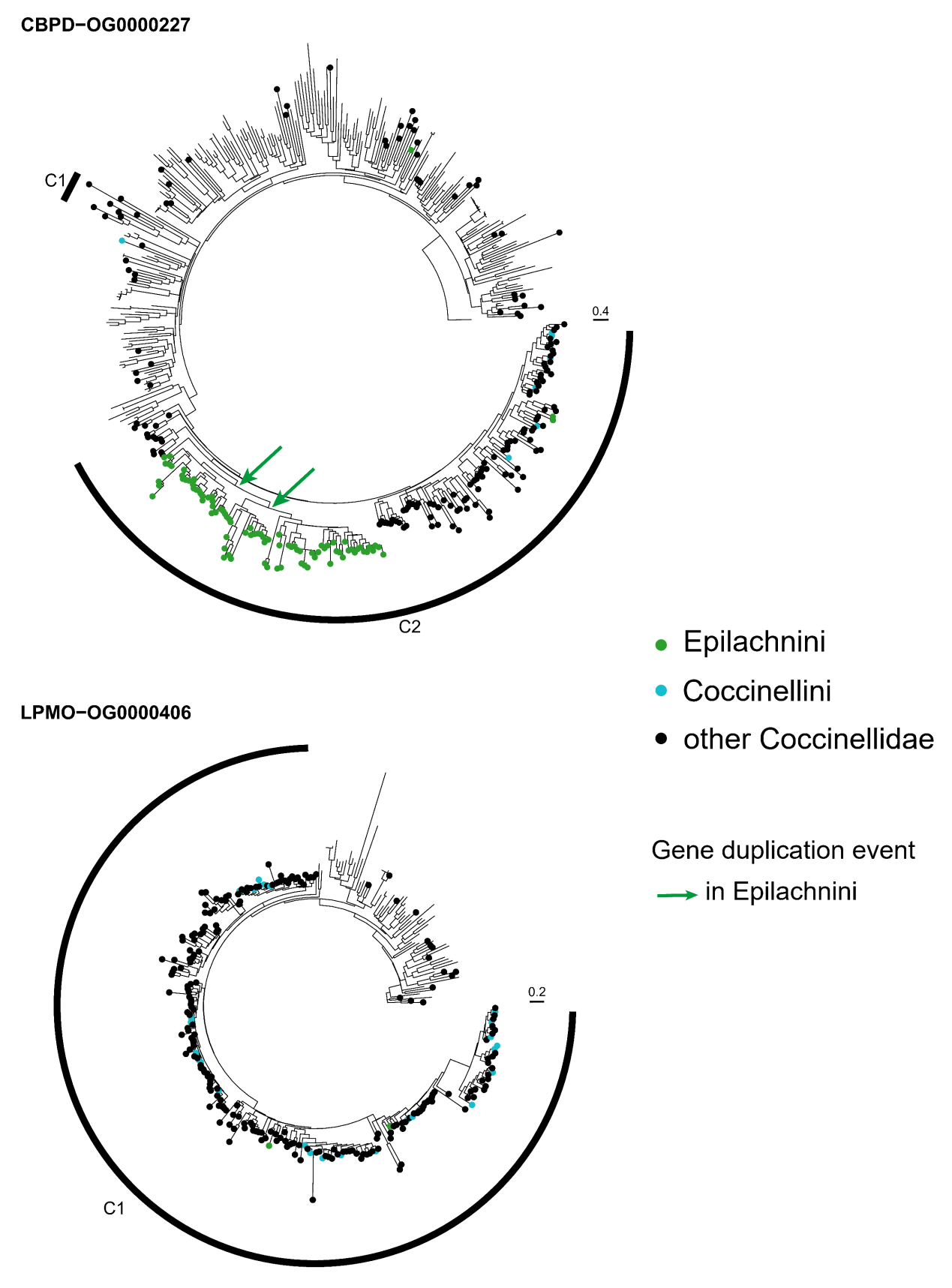


Figure S8.12 Gene trees of ortholog groups (OGs) of CBPDs and LMPOs. The genes in the ladybirds are marked in color and the others without circles are the genes in the outgroup beetles. A high-quality figure can be downloaded from https://github.com/huangyh45/ladybird-genomes-supplementary-figures.


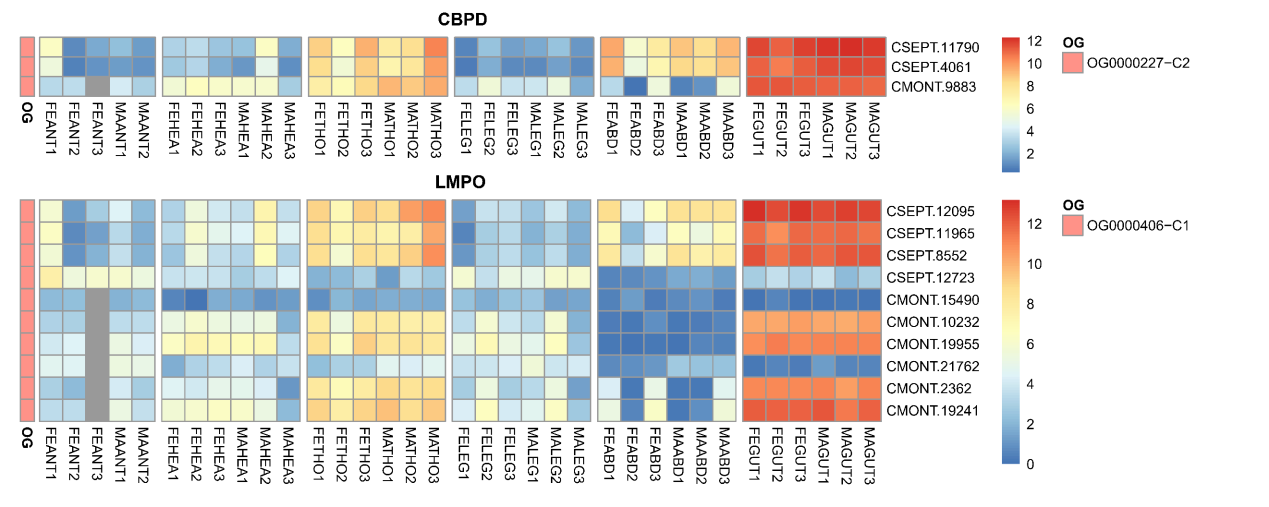


Figure S8.13 Expression patterns of different tissue of each group in ortholog groups (OGs) of CBPDs and LMPOs. Abbreviation in the sample names: FE: female adult, MA: male adult, ANT: antenna, HEA: head, THO: thorax, LEG: leg, ABD: abdomen, GUT: gut. A high-quality figure can be downloaded from https://github.com/huangyh45/ladybird-genomes-supplementary-figures.


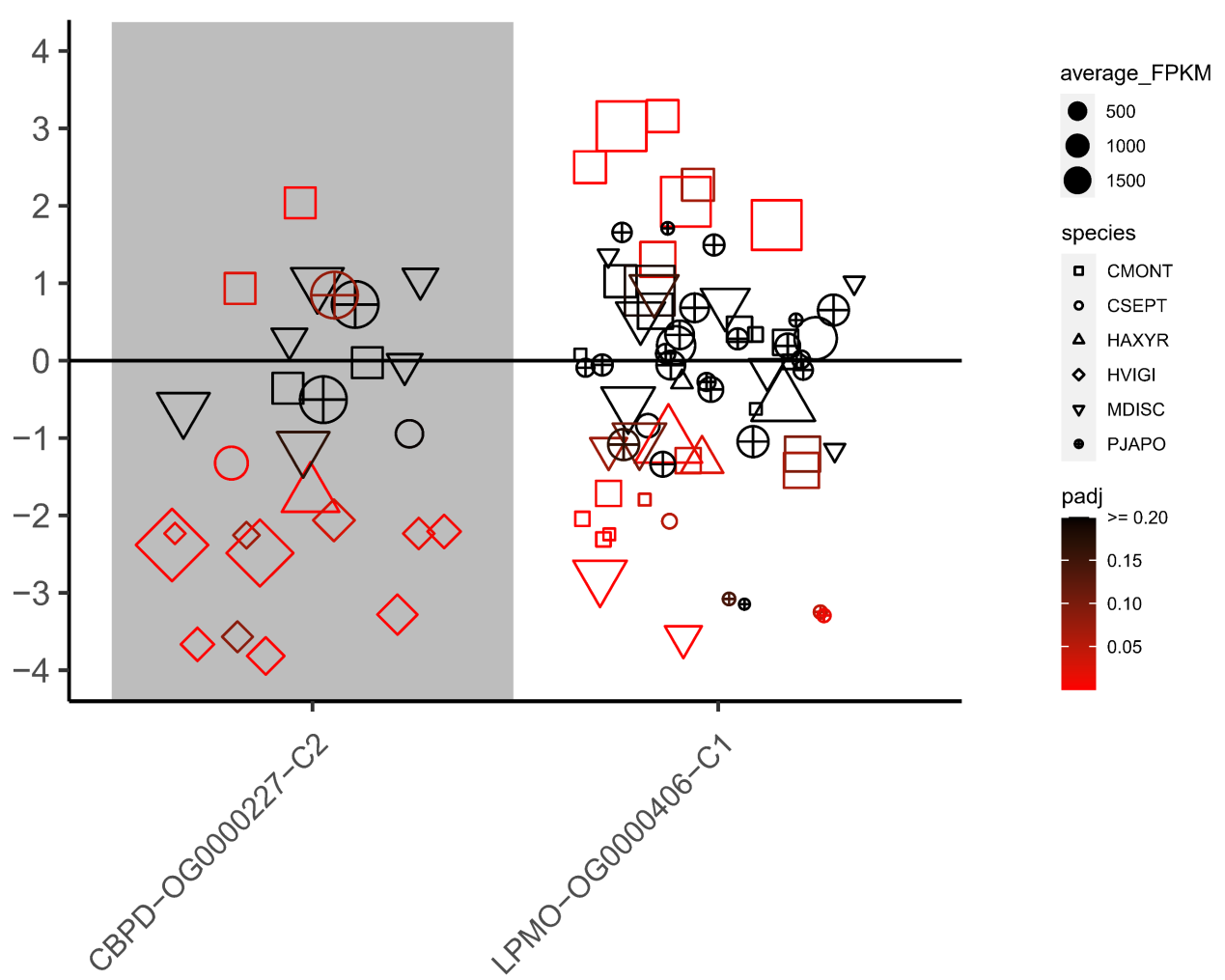


Figure S8.14 Expression patterns under different diet treatments of each group in ortholog groups (OGs) of CBPDs and LMPOs. The vertical axis represents the log2(fold change) values. The positive values on the vertical axis represent upregulation when feeding on the optimal diets, while the negative values represent upregulation when feeding on the non-optimal diets. A high-quality figure can be downloaded from https://github.com/huangyh45/ladybird-genomes-supplementary-figures.


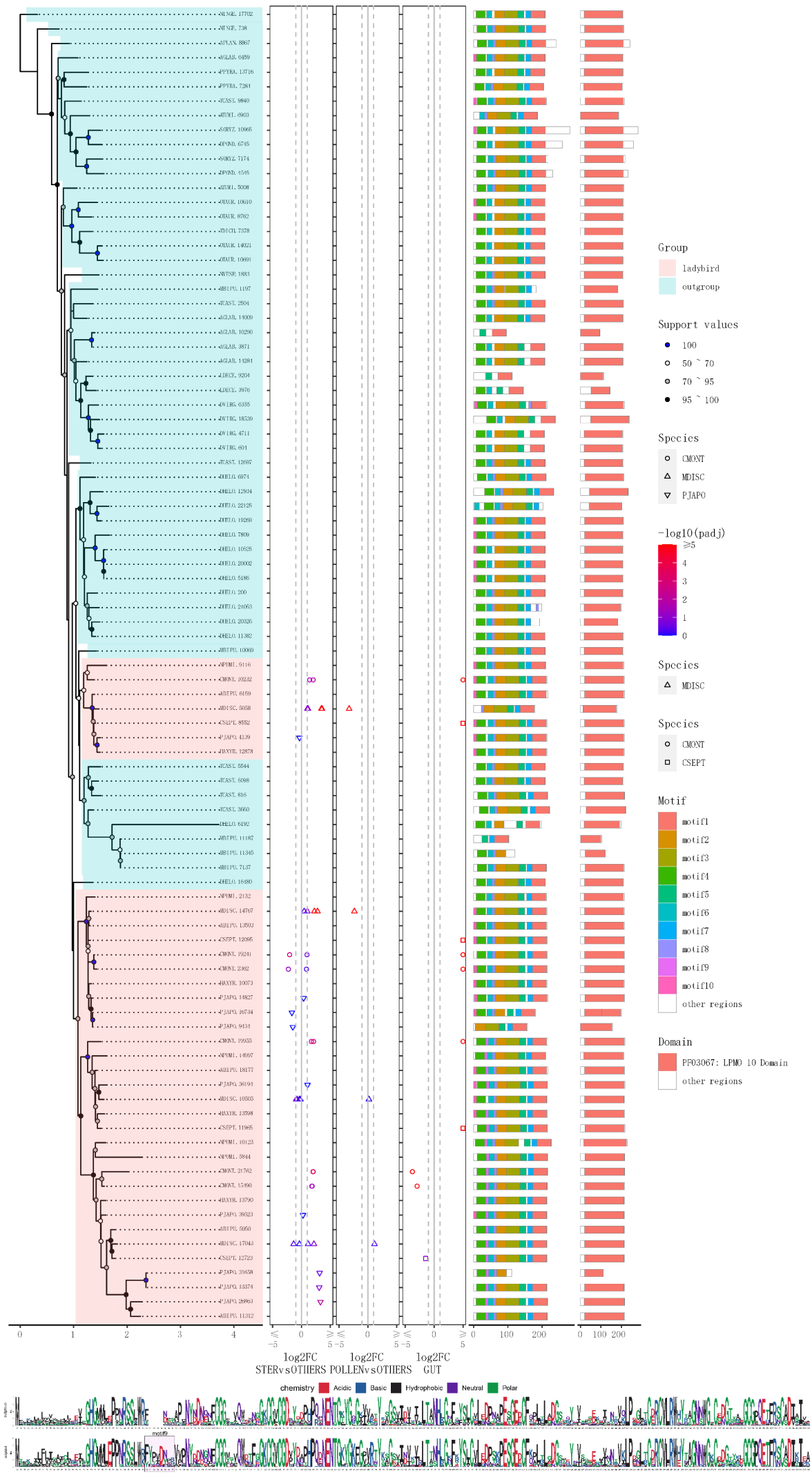


Figure S8.15 Phylogeny and sequence composition of OG0000406 of LMPO. A high-quality figure can be downloaded from https://github.com/huangyh45/ladybird-genomes-supplementary-figures.


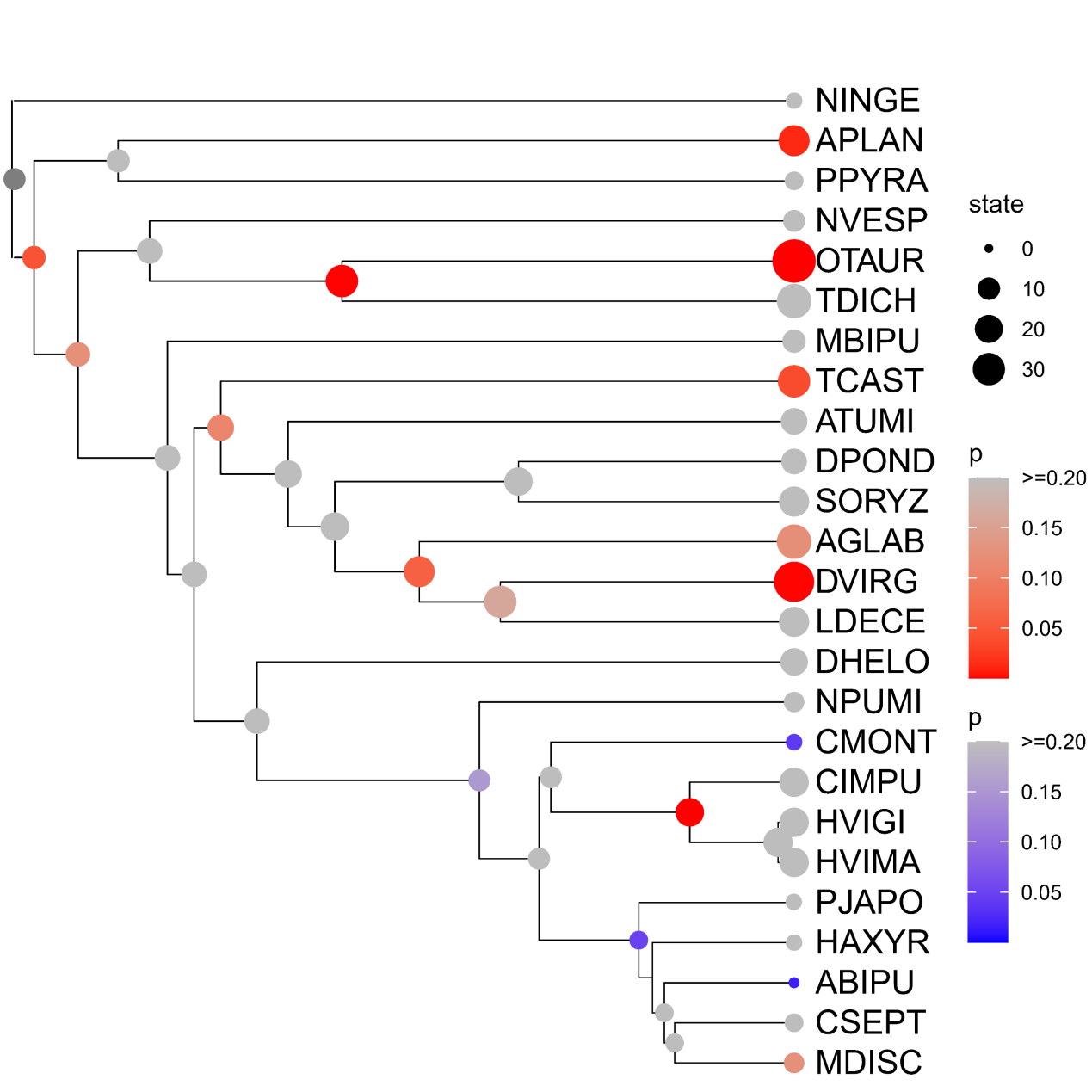


Figure S8.16 Expansion and contraction of OG0000227 (CBPD). The expansions are marked in red, while the contractions OGs are marked in blue. A high-quality figure can be downloaded from https://github.com/huangyh45/ladybird-genomes-supplementary-figures.

# 9 Evolution of candidate genes related to detoxification

## 9.1 Materials and methods

The methods for genes related to detoxification were the same as the methods for chemosensory genes in Section 7.1. The reference genes as the query were the candidate detoxification-related genes of *T. castaneum* (TCAST) from the NCBI Protein database. These detoxification-related genes were obtained through searching the keywords (Table S9.1) on NCBI.

Table S9.1 Information of candidate detoxification genes

|  | Gene name | Pfam accession | No. orthogroups with genes >= 30 / total orthogroups |
| --- | --- | --- | --- |
| ABC | ATP-binding cassette transporter | PF00664, PF01061, PF00005, PF12698, PF12679, PF12848 | 30/100 |
| P450 | Cytochrome P450 | PF00067 | 21/92 |
| GST | Glutathione S-transferase | PF13409, PF00043, PF17172, PF13417, PF02798, PF14497, PF17171, PF13410 | 13/37 |
| UGT | UDP-glucuronosyltransferase | PF00201 | 8/29 |
| COE | Carboxylesterase | PF00135 | 13/45 |
| AKR | Aldo-keto reductase | PF00248 | 5/26 |
| GDH | Glucose dehydrogenase | PF00732, PF05199 | 10/20 |

## 9.2 Results

As the same method as the chemosensory genes with TCAST genes related to detoxification, the genome dataset and the extended dataset are used to identify and analyze the OGs related to detoxification. Details of identification of OG and the expression patterns of their genes can be found in Additional file 2: Table SE6 and SE5 and the corresponding node numbers are shown in Figure S3.1.

### 9.2.1 Phase I: P450 and COE

We identified 21 OGs (>= 30 genes) of P450 and thirteen OGs (>= 30 genes) of COE, including two significantly expanding OGs of P450 (OG0000050 and OG0000153) and one expanding OG of COE (OG0000084) in Epilachnini (Figure S9.1).

-P450

Gene phylogenetic analysis of OG0000050 belonging to CYP6 reveals main expansion of Epilachnini in Clade C6 (Figure S9.2). In this OG, 5/17 genes are gut-specific highly expressed genes, which also mainly exist in Clade C6 experiencing at least two gene duplication events in Epilachnini (Figure S9.3). Among 24 genes of OG0000050 in HVIGI, six are downregulated and one is upregulated when the herbivorous ladybird feeds on sugar water (Figure S9.4). This OG also contain large amounts of diet-specific DEGs in the carnivorous ladybirds, including 6/7 genes in CMONT and 6/24 genes in PJAPO.

Expansion of OG0000153, whose gene member mainly belong to CYP4 or CYP345, mainly occurs in Clade C2 and C3 in the gene tree (Figure S9.2). Based on the heatmap of expression in different tissues, the gene expression in the gut is not higher than expression in other tissues (Figure S9.3). In HVIGI, 3/11 downregulated DEGs are detected under sugar water diet treatment (Figure S9.4).

-COE

Clade C6 of OG0000084 is the group where genes of Epilachnini mainly expand. Furthermore, two gene duplication events of Epilachnini happen in Clade C4 and C5 respectively, and Clade C5 lost the genes in Coccinellini species (Figure S9.2). Most genes of this OG have higher expression in the gut, thorax and abdomen, except Clade C4 (Figure S9.3). many diet-specific DEGs are found in OG0000084, including 4/17 downregulated DEGs in HVIGI and 6/7 DEGs in CMONT when the ladybirds eat non-optimal food (Figure S9.4).

In addition, OG0000044 of P450 (CYP9) significantly expands in MDISC, along with a gene duplication event in Epilachnini. Although relative amounts of genes in this OG are highly expressed in gut, only 1/10 sugar water-specific DEG in HVIGI is found in the duplicated clade. OG0000081 of P450 (CYP4) also has duplicated clades of Epilachnini genes in Clade C3, with no diet-specific DEGs detected in HVIGI. OG0000173 of COE also has a gene duplication event in Epilachnini. However, no diet-specific DEGs in HVIGI are detected when they feed on sugar water. OG0013146 of P450 (CYP12/301/334) are lost in Coccinellidae, and OG0030674 are emergent in Epilachnini, but without diet-specific DEGs in HVIGI. OG0010337 of P450 (CYP3A4) contain a clade losing Coccinellini species with 2/2 diet-specific DEGs in CMONT and 1/2 diet-specific DEGs in HVIGI, but genes in this OG are not highly expressed in gut.

The phase I enzymes (P450s and COEs) are able to add functional groups to a variety of endogenous toxic compounds and exogenous substances to reduce their biological activity (Chen, 2012). They are highly expressed in the midgut of insects, which are likely to manage the toxic components in the diets (Li et al., 2007). Based on our results, mainly CYP4, CYP6 and CYP9 of P450 expand or are dupliacted in Epilachnini. Among the four major clades of insect P450s, CYP6 and CYP9 are within the CYP3 clade, while CYP4 is in the CYP4 clade (Feyereisen, 2006). Multiple insect-specific blooms of gene duplications occurred in the CYP3 clade and the CYP4 clade shows a great diversity of gene sequences and functions (Feyereisen, 2006). The CYP4, CYP6 and CYP9 genes are reported involved in allelochemical tolerance, and the expansions of the members in CYP6 in insect generalists are one of the differences with specialists (Li et al., 2007). On these bases, the expansions of CYP4, CYP6 and CYP9 in Epilachnini can actually be connected with their diet shift from carnivory to photophagy. Highly expression in the gut and large amounts of the genes downregulated in sugar water-feeding HVIGI of at least OG0000050 (CYP6 of P450) and OG0000084 (COE) also indicate that their expansions in Epilachnini should be related to plant toxin management and eliminates the possibility of other function of P450s and COEs in the ladybirds (e.g. cuticular hydrocarbon biosynthesis of *CYP4G* in HAXYR) (Zhang et al., 2021) (Table S9.2). Additionally, P450s in HVIMA have been previously reported much more than Coccinellini speicies except PJAPO (Zhu et al., 2023), and we extend this discovery to other Epilachnini species and exclude the condition of species-specific patterns.

### 9.2.2 Phase II: GST and UGT

Thirteen OGs are identified as GSTs in our data, with gene members >= 30.

-GST

Among the GST OGs, OG0000289 significantly expand in Epilachnini, mainly in Clade C2 and C7, and contract in Coccinellini (Figure S9.5, S9.6). When fed by sugar water, 2/7 genes in Clade C7 are detected as downregulated DEGs in HVIGI (Figure S9.8). Furthermore, the genes in Clade C7 of this OG are highly expressed in the gut (Figure S9.7, Additional file 2: Table SE5). In OG0000125, Clade C7 lost Coccinellini genes and contains 1/5 upregulated DEG in CMONT that is highly expressed in antenna.

We also found eight OGs of UGT with gene members >= 30. OG0000113 significantly expand in Epilachnini, with main gene increase and a Epilachnini-specific gene duplication event in Clade C3 (Figure S9.6). Almost all the genes of OG0000113 are gut-specific (Figure S9.7) and the OG includes 4/22 downregulated DEGs in HVIGI and 10/15 DEGs in MDISC (Figure S9.8).

-UGT

In addition, OG0000247 of UGT significantly expands in Coccinellidae and contracts in Coccinellini (Figure S9.5), with 15/25 gut-specific highly expressed genes, ten of which belong to Cluster Gut in Section 6.2 (Figure S9.7). When eating non-optimal diets, CMONT have 9/21 DEGs, CSEPT have 1/4 upregulated DEGs and PJAPO have 4/14 upregulated DEGs (Figure S9.8).

OG0000439 of UGT also significantly expand in Coccinellidae (Figure S9.5). In the gut, 7/12 genes of this OG are highly expressed, including 5 genes in Cluster Gut in Section 6.2 (Figure S9.7). In CMONT, 4/10 genes are downregulated DEGs under non-optimal diet treatments, while 5/10 genes are downregulated DEGs in MDISC (Figure S9.8).

In OG0000047 of UGT, genes of Coccinellini are duplicated at least once and genes of Epilachnini are duplicated at least twice in Clade C8. This OG is mostly gut-specific and Clade C8 contains 2/9 DEGs in CMONT, 4/19 upregulated DEGs in PJAPO, 1/10 downregulated DEG in HAXYR, 2/13 downregulated DEGs in MDISC and 3/13 downregulated DEGs in HVIGI when the ladybirds eat non-optimal diets. OG0000229 of UGT also contains a Epilachnini-duplicated clade in Clade C3, with high expression in gut and 1/3 downregulated DEG in sugar water-fed HVIGI.

The phase II enzymes (GSTs and UGTs) couple endogenous small cofactor molecules to toxic byproducts of phase I metabolism and generate hydrophilic metabolites and they are also highly expressed in the insect midgut to manage the toxin in the diets (Chen, 2012; Li et al., 2007). At least OG0000113 of UGT and Clade C7 in OG0000289 of GST are highly expressed in the gut and tend to downregulated when HVIGI feeds on sugar water. Its expansion in Epilachnini is likely to be involved in detoxification during adaptation to plant. Interestingly, several UGT OGs also expand in Coccinellidae and differentially expressed in the carnivorous ladybirds, which seem to be associated with adaptation to their prey. In previous research, the activity of GSTs is connected to different diets in CSEPT (Nielsen et al., 2000), but we do not find evolution events of GST in Coccinellidae or Coccinellini.

### 9.2.3 Phase III: ABC

Our methods found 30 OGs of ABC, which contain at least 30 gene members (Table S9.1). However, no genes under significant expansion or contraction are detected (Figure S9.9). For the clade level, in OG0000169 of ABC, mainly containing genes of ABC-C subfamily, genes of Coccinellidae are duplicated between Clade C3 and C4, and genes of Epilachnini are duplicated in Clade C3 (Figure S9.10). This OG is mainly expressed highly in gut and contain 3/8 upregulated DEGs in CMONT, 3/8 downregulated DEGs in PJAPO, 2/5 DEGs in MDISC and 2/5 DEGs in CSEPT when feeding on non-optimal diets (Figure S9.11, S9.12).

In OG0000205, with genes mainly annotated as ABC-B subfamily, Epilachnini genes are duplicated at least twice in Clade C1 and at least once in Clade C4, while the gene duplication in Coccinellidae causes Clade C3 and C4. Highly expressed genes in gut are mostly in Clade C1, which contain 1/5 sugar water-derived downregulated DEG in HVIGI.

OG0000385 (multidrug resistance-associated protein 1-like) and OG0000921 (ABC-G subfamily) has a Coccinellidae-specific gene duplication event respectively. But few genes in the duplicated clades are gut-specific and diet-specific DEGs. Clade C3 in OG0001461 (cationic amino acid transporter) lost all Coccinellini genes, but gut-specific expression pattern is not found obviously. Therefore, we consider that not enough evidence can support their connection to feeding habits in the ladybirds.

The phase III (or 0) transporters (ABCs) are related to excretion of soluble toxic compounds (Chen, 2012). In the leaf beetles, ABCs are also reported to transport phytotoxin or metabolites from the hemolymph into defensive secretions (Strauss et al., 2013). Our results indicate a relatively stable gene counts of ABC OGs between the herbivorous ladybirds, the carnivorous ladybirds and the outgroup beetles. However, at least OG0000169 of ABC-C and OG0000205 of ABC-B undergo duplication events in Coccinellidae or Epilachnini and contain highly expressed genes in gut and diet-specific DEGs. Actually, xenobiotic transporting roles have been suggested for the ABC-A, ABC-B, ABC-C, and ABC-G subfamilies, in which ABC-B and ABC-G may be involved in transport of plant secondary compounds (Dermauw and Van Leeuwen, 2014). The different ABC subfamilies are abundant in different insect groups, respectively, for example, the ABC-C family is much more in the Cucujiformia species than other insects, which indicates different patterns of detoxification in different insect linages (Denecke et al., 2021). The duplications of ABC-B and ABC-C in Epilachnini species also implies the improvement of their xenobiotic and plant toxin transporting ability with a specific evolutionary pattern. All these support that ABC may contribute to adaptation to new prey or plant diets at the ancestor of Coccinellidae and Epilachnini, possibly by managing the toxin in the diets.

### 9.2.4 Other candidate detoxification enzymes: AKR and GDH

-AKR

Five OGs of AKR are identified with at least 30 gene members, including OG0000276 under significant expansion in Epilachnini (Figure S9.13). Its gene tree shows that main expansion occurs in Clade C1 (Figure S9.14). At least four gene duplication events in Epilachnini occur in Clade C1. Most genes of OG0000276 are highly expressed in the gut (Figure S9.15). Feeding on sugar water instead of plant leaves, 2/14 genes are significantly downregulated in HVIGI (Figure S9.16). AKRs are both phase I enzymes catalyzing the reduced nicotinamide adenine dinucleotide (phosphate) (NAD(P)H)-dependent oxido-reduction of carbonyl groups on a wide range of phytotoxins and phase II enzymes functionalizing the carbonyl group for conjugation at the same time (Mindnich and Penning, 2009). The expansion in Epilachnini, gut-specific and diet-specific expression of OG0000276 reveal its potential contribution to plant detoxification.

-GDH

Additionally, we found ten OGs of GDH (>= 30 gene members) and OG0000136 (glucose dehydrogenase [FAD, quinone]-like) significantly expands in Epilachnini, mainly in Clade C8 (Figure S9.14). Gene duplication events of Coccinellini and Epilachnini genes take place in Clade C7 and C8 respectively. Only genes in Clade C2 are gut-specific according to the expression heatmap (Figure S9.15). But diet-specific DEGs are few in OG0000136, including 1/20 downregulated genes in HVIGI, 2/10 genes in CMONT, 1/7 genes in HAXYR and 1/9 genes in MDISC (Figure S9.16). GDHs are enzymes potentially related to plant defense suppression in Hemiptera (Carolan et al., 2011; Musser et al., 2002). The OG’s expansion in Epilachnini and gut-specific expression support its important role in plant adaptation but few diet-specific DEGs remain the role unclear. OG0009119 of GDH (glucose dehydrogenase [FAD, quinone]-like) is also found emergent in Coccinellidae, with high expression in head and no diet-specific DEGs, which is hardly connected to detoxification of the diets.

OG0000423 of GDH (glucose dehydrogenase [FAD, quinone]-like) experience at least two Epilachnini-specific gene duplication events in Clade C2. The clade includes highly expressed genes in gut of CIMPU and 2/9 downregulated DEGs in sugar water-fed HVIGI, which seems to be related to detoxification in the gut of herbivorous ladybirds.

## 9.3 Summary

In this study, the OGs related to detoxification were identified and analyzed, including P450s, COEs, GSTs, UGTs, ABCs, AKRs and GDHs. Notably, at least several OGs of P450, COE, GST, UGT, ABC, AKR and GDH are considered related to plant adaptation in Epilachnini, which cover all the three phases of the insect detoxification enzyme system, while few OGs significantly expand or contain duplicated clades in Coccinellidae with prey shift. These results show the connection between herbivory in this group of ladybirds and the wide expansions of detoxification-related OGs, which is similar with the previous conclusion in other beetles (McKenna et al., 2016; Seppey et al., 2019). These genes seem to be able to help the herbivorous ladybirds, Epilachnini, to manage the plant secondary compounds, as same as other herbivorous insects (Heidel-Fischer and Vogel, 2015; Li et al., 2007).

In addition, the detoxification-related genes seem to be also involved in diet adaptation of carnivorous ladybirds. Relatively large amounts of DEGs related to detoxification are detected in the transcriptomic comparison of APHID vs MEALYBUG in CMONT. The comparison of same treatments in CMONT adults present similar results (Li et al., 2016a). However, those aphidophagous species in Coccinellini has fewer detoxification-related DEGs in the comparison of MEALYBUG vs APHID. It seems that the coccidophagous CMONT needs to manage more toxins in the aphids. Combined with the evolutionary events, at least two UGT OG is likely to respond to the prey shift. It has been reported that the plant toxin or components (e.g. glucosinolates and phytoene) can be transmitted or processed into other toxins (e.g. isothiocyanates and carotenoids) through aphids and have an impact on the ladybirds (Francis et al., 2001; Takemura et al., 2021), which indicates a potential demand to manage toxins in prey adaptation of the carnivorous ladybirds.

Table S9.2 The reported function of the candidate detoxification-related genes in the ladybirds.

| Reported gene name | Gene ID | Ortholog group | Gene family | High expression in qPCR | Functional verification | Reference |
| --- | --- | --- | --- | --- | --- | --- |
| ComCYP9Z401 | CMONT.9527 | OG0000044 | P450 | - | - | Zhang et al. (2015) |
| HaxyCYP4G79 | HAXYR.6020 | OG0000517 | P450 | - | an oxidative decarbonylase in hydrocarbon biosynthesis and regulate cuticular melanization | Zhang et al. (2021) |
| Ha-ABCG-4C | HAXYR.7456 | OG0005520 | ABC | - | RNAi leads to lethality during the pupal period | Tsuji et al. (2018) |
| Ha-w-5 | HAXYR.8353 | OG0000175 | ABC | - | RNAi leads to no difference | Tsuji et al. (2018) |
| Ha-w-4 | HAXYR.12013 | OG0000175 | ABC | - | RNAi leads to no difference | Tsuji et al. (2018) |
| Ha-w-6 | HAXYR.2778 | OG0000175 | ABC | - | RNAi leads to no difference | Tsuji et al. (2018) |
| Ha-w-3 | HAXYR.4219 | OG0000175 | ABC | - | RNAi leads to no difference | Tsuji et al. (2018) |
| Ha-w-2 | HAXYR.10971 | OG0000175 | ABC | - | eye pigmentation | Tsuji et al. (2018) |
| Ha-St | HAXYR.11720 | OG0000175 | ABC | - | eye pigmentation | Tsuji et al. (2018) |
| HvWhite1 | HVIGI.17400 | OG0000175 | ABC | eggs and adults | RNAi leads to no difference | Xu et al. (2020) |
| HvWhite2 | HVIGI.16730 | OG0000175 | ABC | larvae | RNAi impair the climbing ability of the adults | Xu et al. (2020) |
| HvWhite3 | HVIGI.8540 | OG0000175 | ABC | eggs, first instar larvae and adults | eye pigmentation | Xu et al. (2020) |
| HvWhite4 | HVIGI.17828 | OG0000175 | ABC | eggs and adults | RNAi leads to no difference | Xu et al. (2020) |
| HvWhite5 | HVIGI.9651 | OG0000175 | ABC | eggs | RNAi leads to no difference | Xu et al. (2020) |


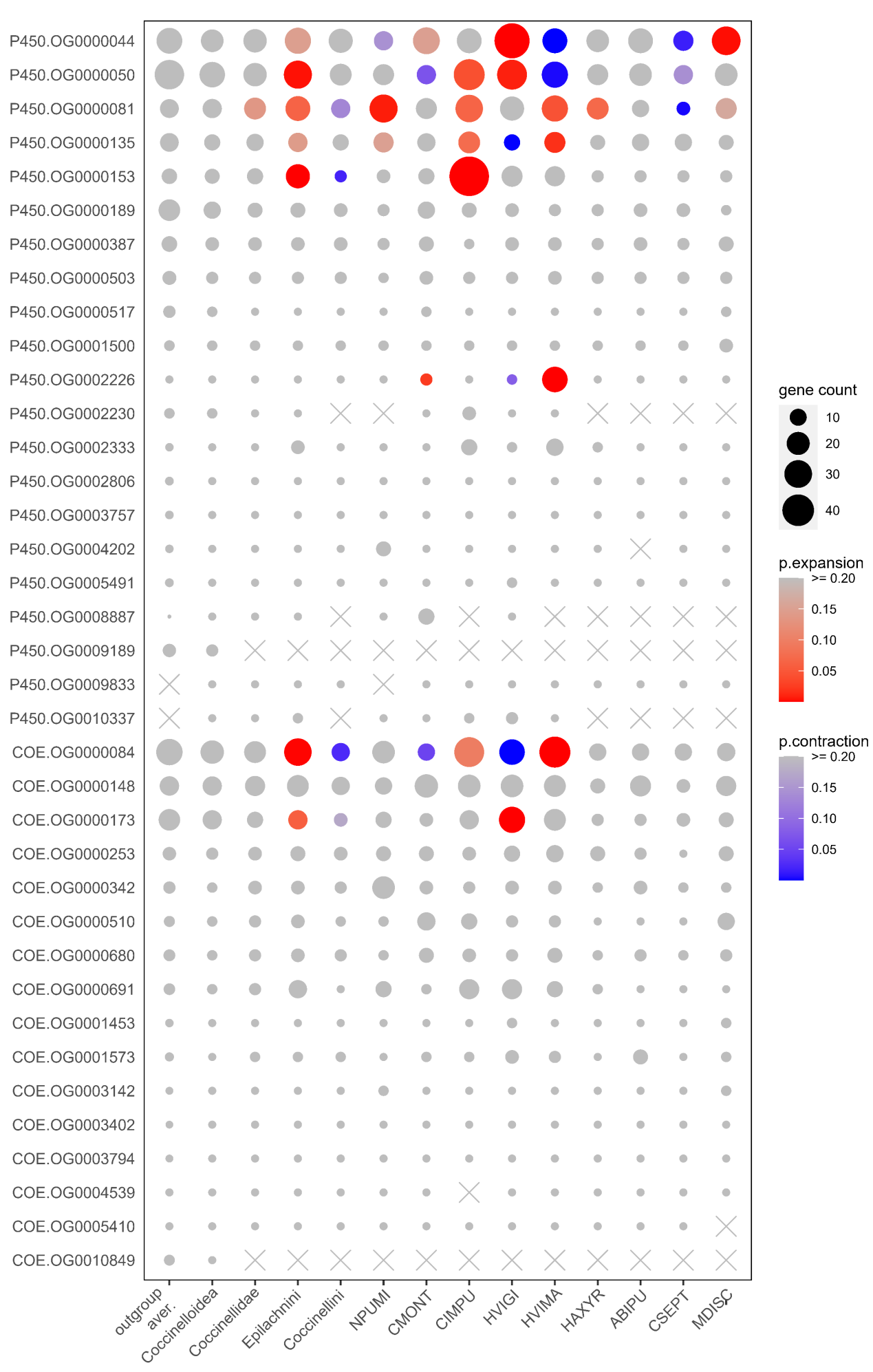


Figure S9.1 Comparison of gene counts in ortholog groups (OGs) of P450s and COEs in the ten ladybird genomes. Only OGs with total genes >= 30 are shown. Gene counts at the nodes of Coccinelloidea, Coccinellidae, Epilachnini and Coccinellini are reconstructed by CAFE. A high-quality figure can be downloaded from https://github.com/huangyh45/ladybird-genomes-supplementary-figures.


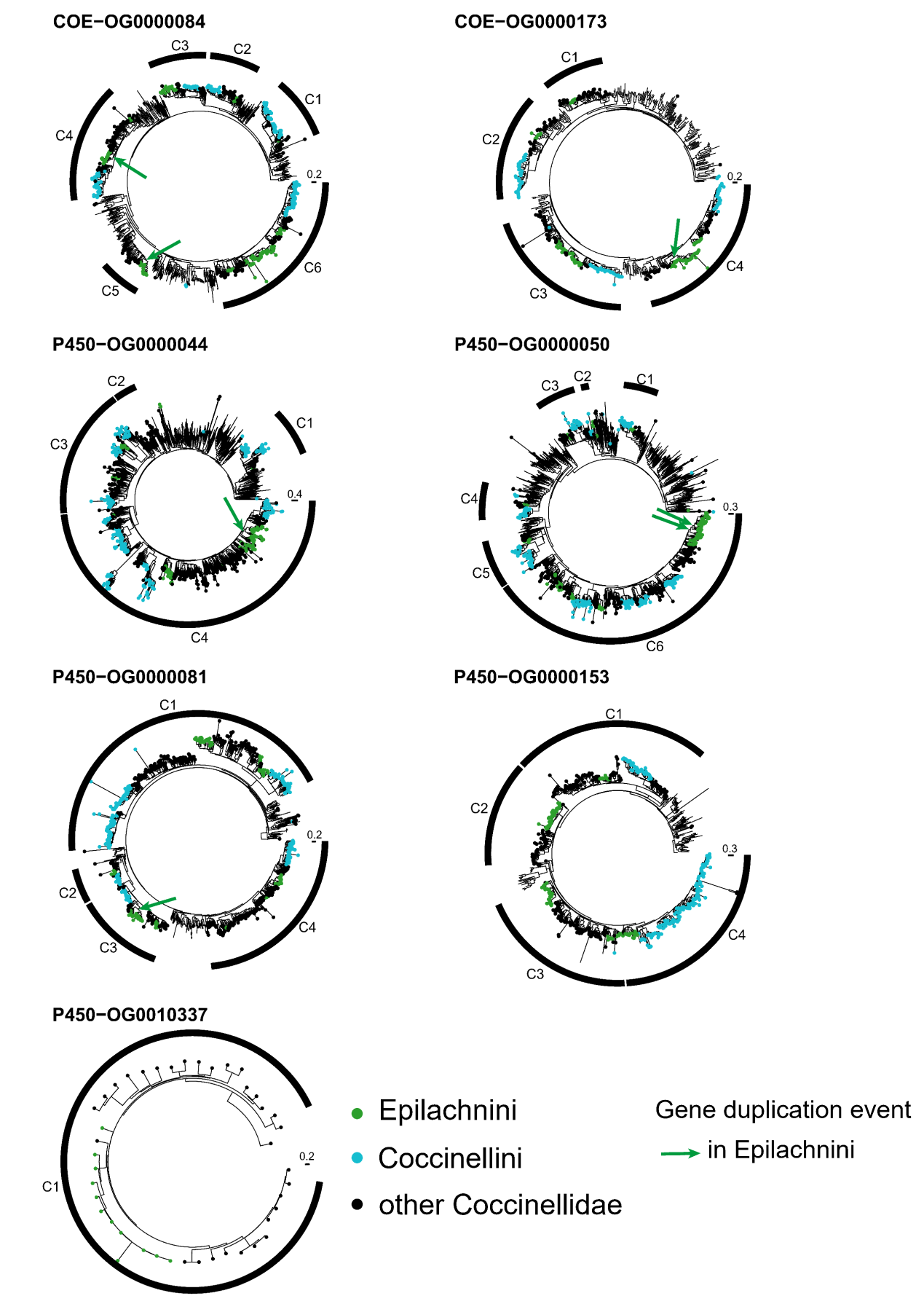


Figure S9.2 Gene trees of ortholog groups (OGs) of P450s and COEs. The genes in the ladybirds are marked in color and the others without circles are the genes in the outgroup beetles. A high-quality figure can be downloaded from https://github.com/huangyh45/ladybird-genomes-supplementary-figures.


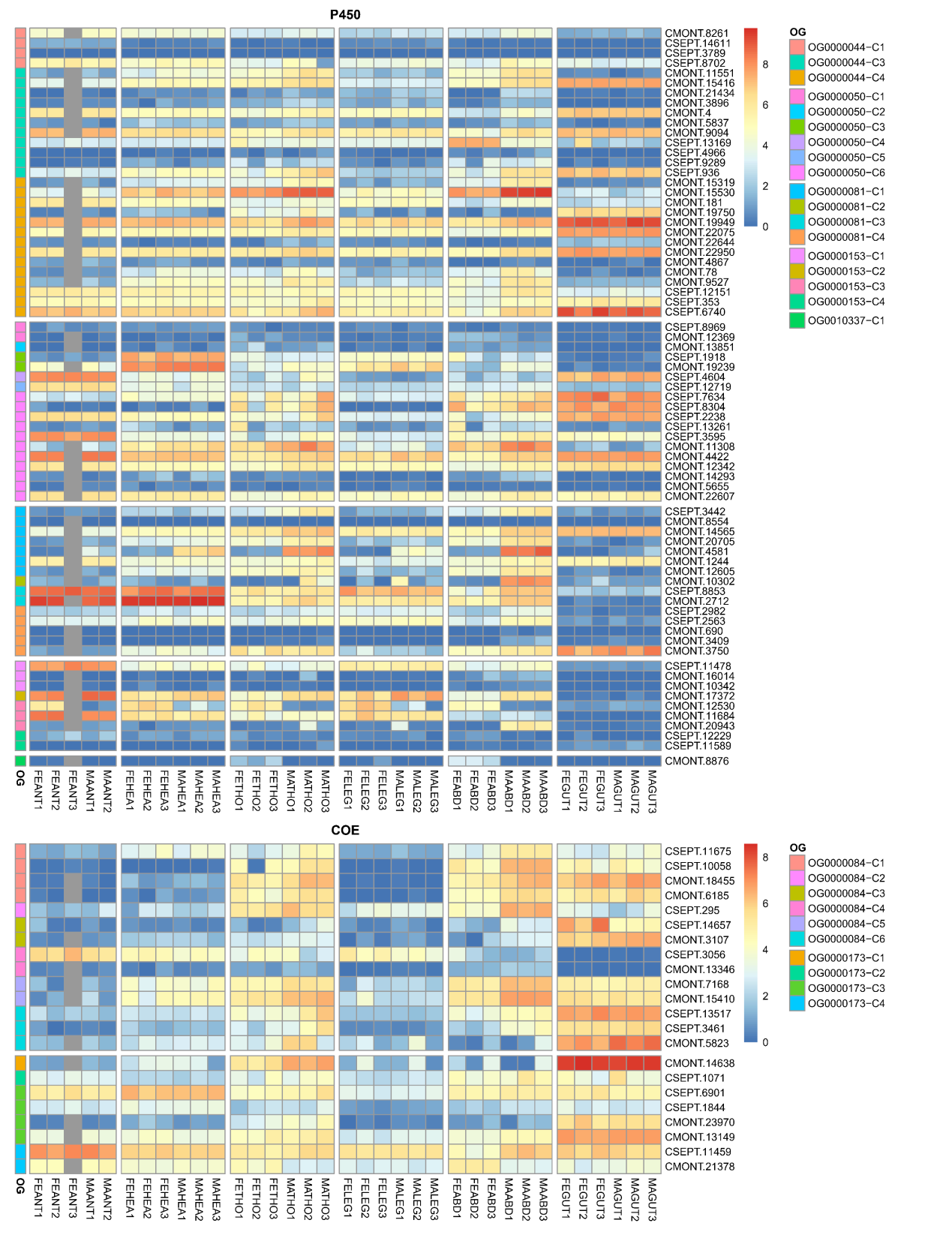


Figure S9.3 Expression patterns of different tissue of each group in ortholog groups (OGs) of P450s and COEs. Abbreviation in the sample names: FE: female adult, MA: male adult, ANT: antenna, HEA: head, THO: thorax, LEG: leg, ABD: abdomen, GUT: gut. A high-quality figure can be downloaded from https://github.com/huangyh45/ladybird-genomes-supplementary-figures.


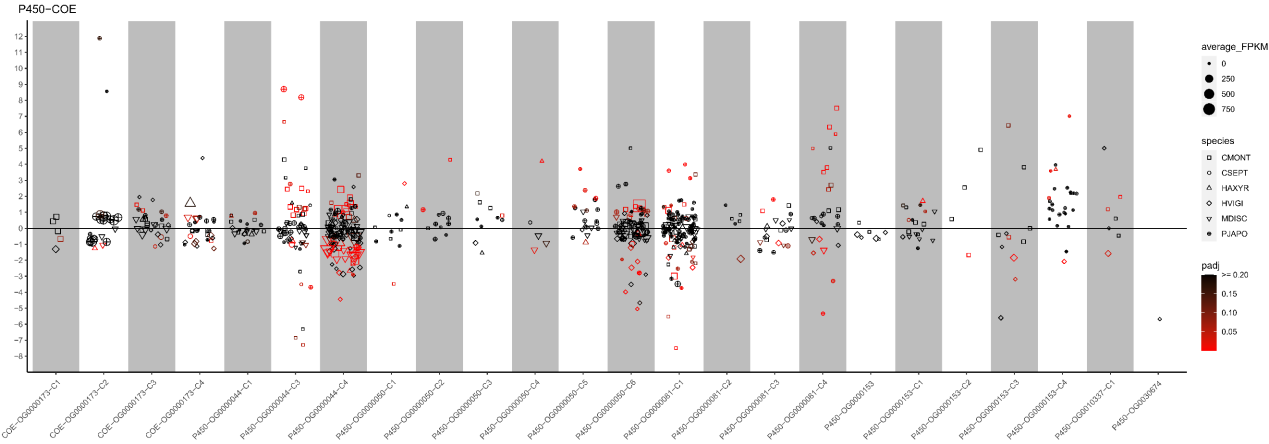


Figure S9.4 Expression patterns under different diet treatments of each group in ortholog groups (OGs) of P450s and COEs. The vertical axis represents the log2(fold change) values. The positive values on the vertical axis represent upregulation when feeding on the optimal diets, while the negative values represent upregulation when feeding on the non-optimal diets. A high-quality figure can be downloaded from https://github.com/huangyh45/ladybird-genomes-supplementary-figures.


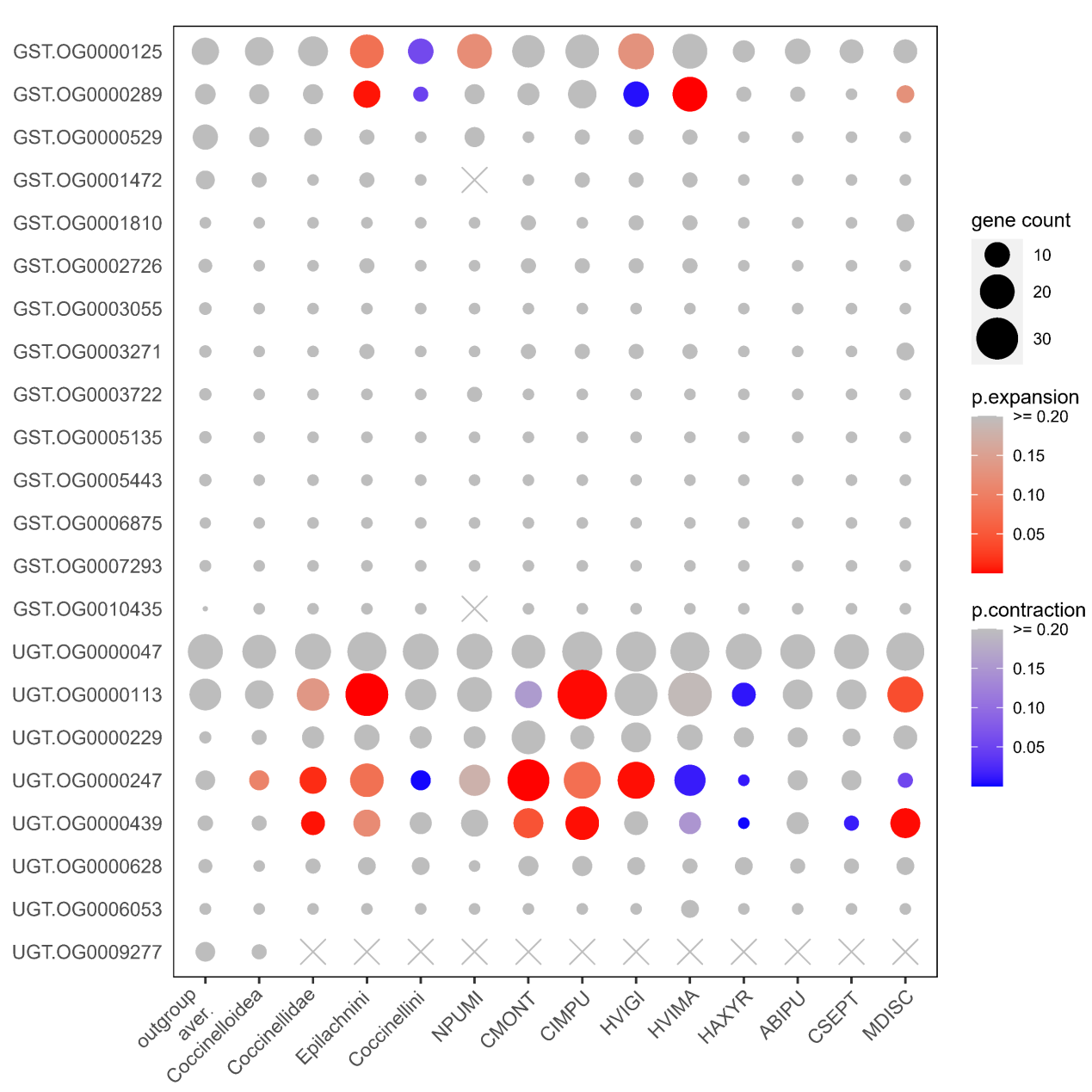


Figure S9.5 Comparison of gene counts in ortholog groups (OGs) of GSTs and UGTs in the ten ladybird genomes. Only OGs with total genes >= 30 are shown. Gene counts at the nodes of Coccinelloidea, Coccinellidae, Epilachnini and Coccinellini are reconstructed by CAFE. A high-quality figure can be downloaded from https://github.com/huangyh45/ladybird-genomes-supplementary-figures.


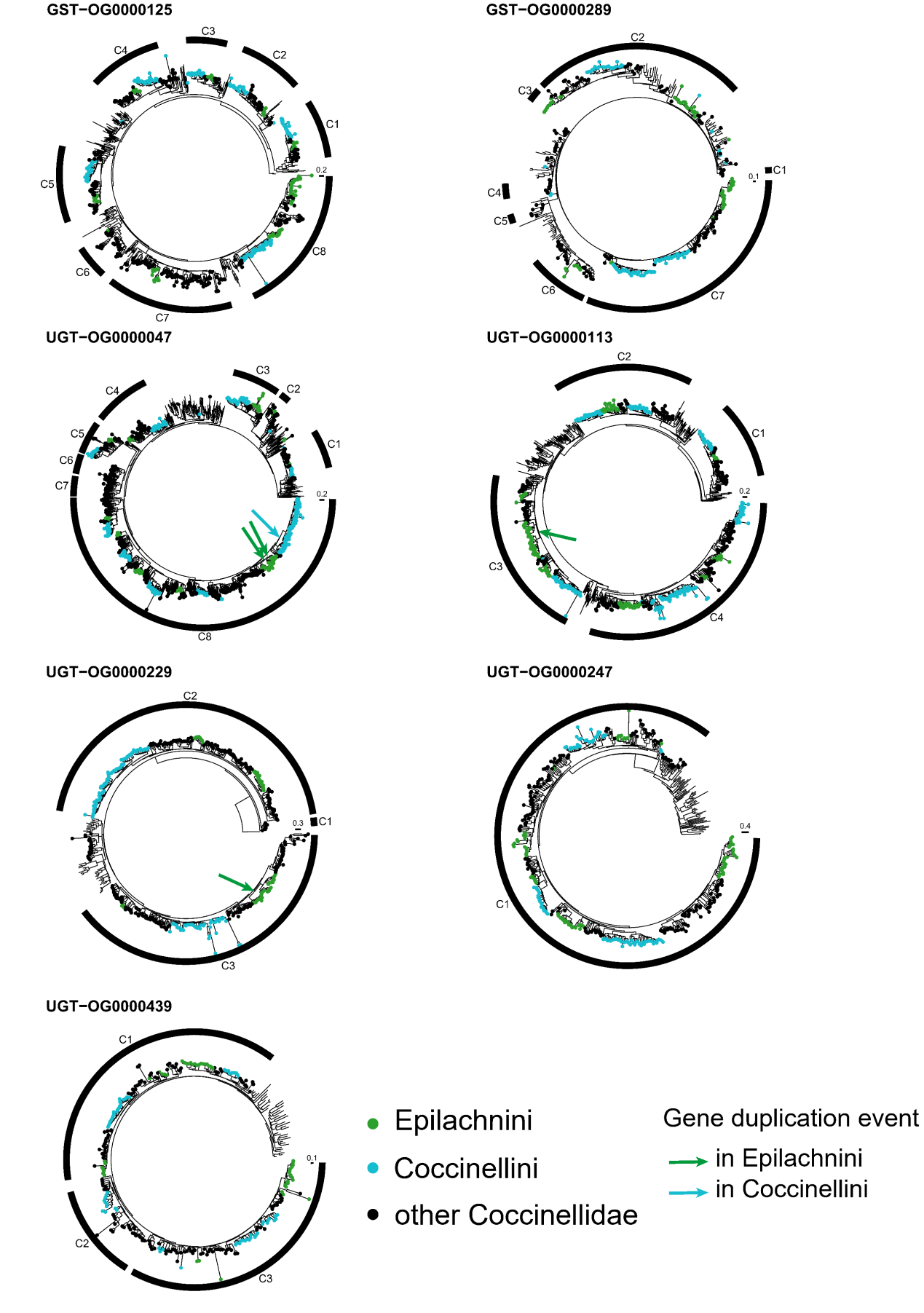


Figure S9.6 Gene trees of ortholog groups (OGs) of GSTs and UGTs. The genes in the ladybirds are marked in color and the others without circles are the genes in the outgroup beetles. A high-quality figure can be downloaded from https://github.com/huangyh45/ladybird-genomes-supplementary-figures.


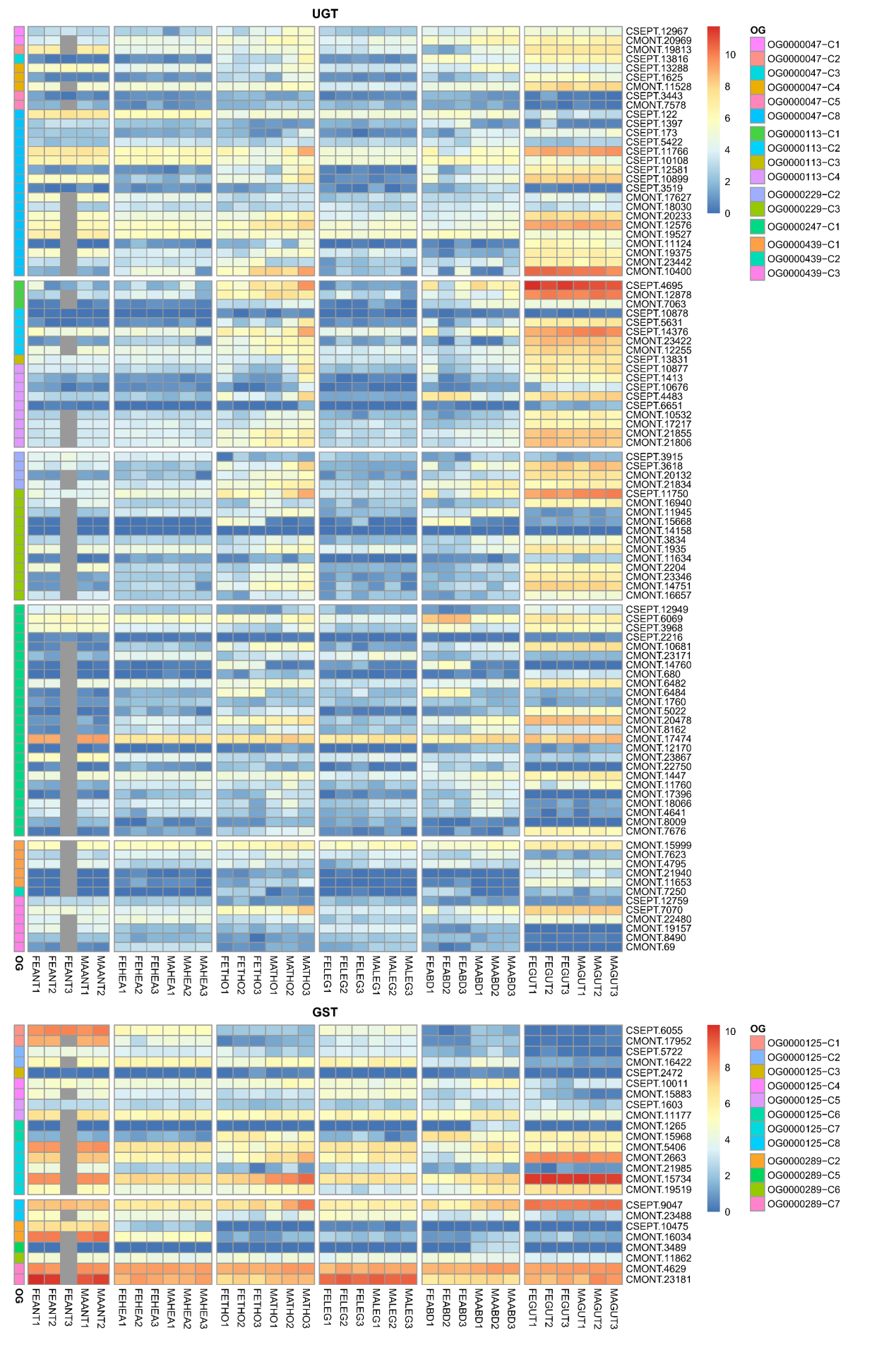


Figure S9.7 Expression patterns of different tissue of each group in ortholog groups (OGs) of GSTs and UGTs. Abbreviation in the sample names: FE: female adult, MA: male adult, ANT: antenna, HEA: head, THO: thorax, LEG: leg, ABD: abdomen, GUT: gut. A high-quality figure can be downloaded from https://github.com/huangyh45/ladybird-genomes-supplementary-figures.


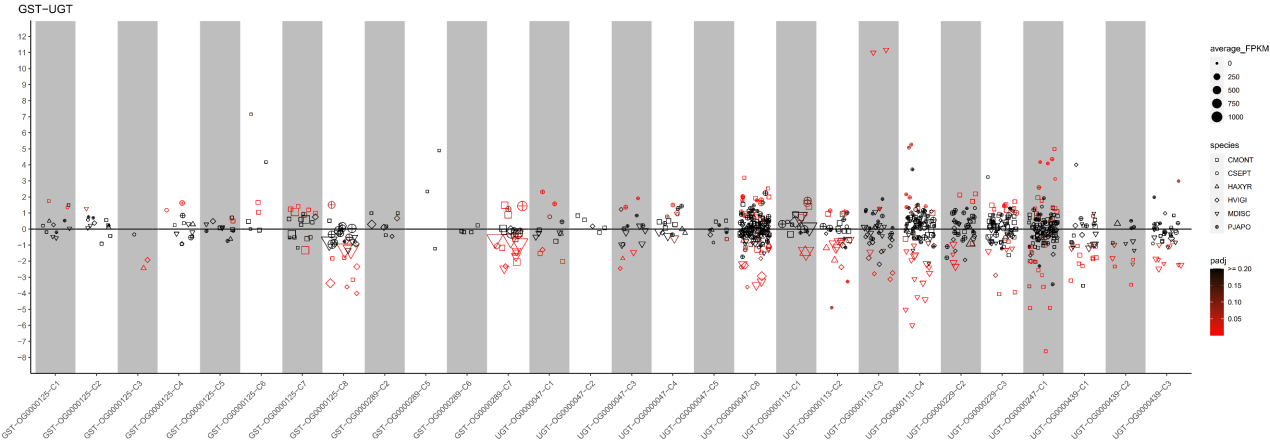


Figure S9.8 Expression patterns under different diet treatments of each group in ortholog groups (OGs) of GSTs and UGTs. The vertical axis represents the log2(fold change) values. The positive values on the vertical axis represent upregulation when feeding on the optimal diets, while the negative values represent upregulation when feeding on the non-optimal diets. A high-quality figure can be downloaded from https://github.com/huangyh45/ladybird-genomes-supplementary-figures.


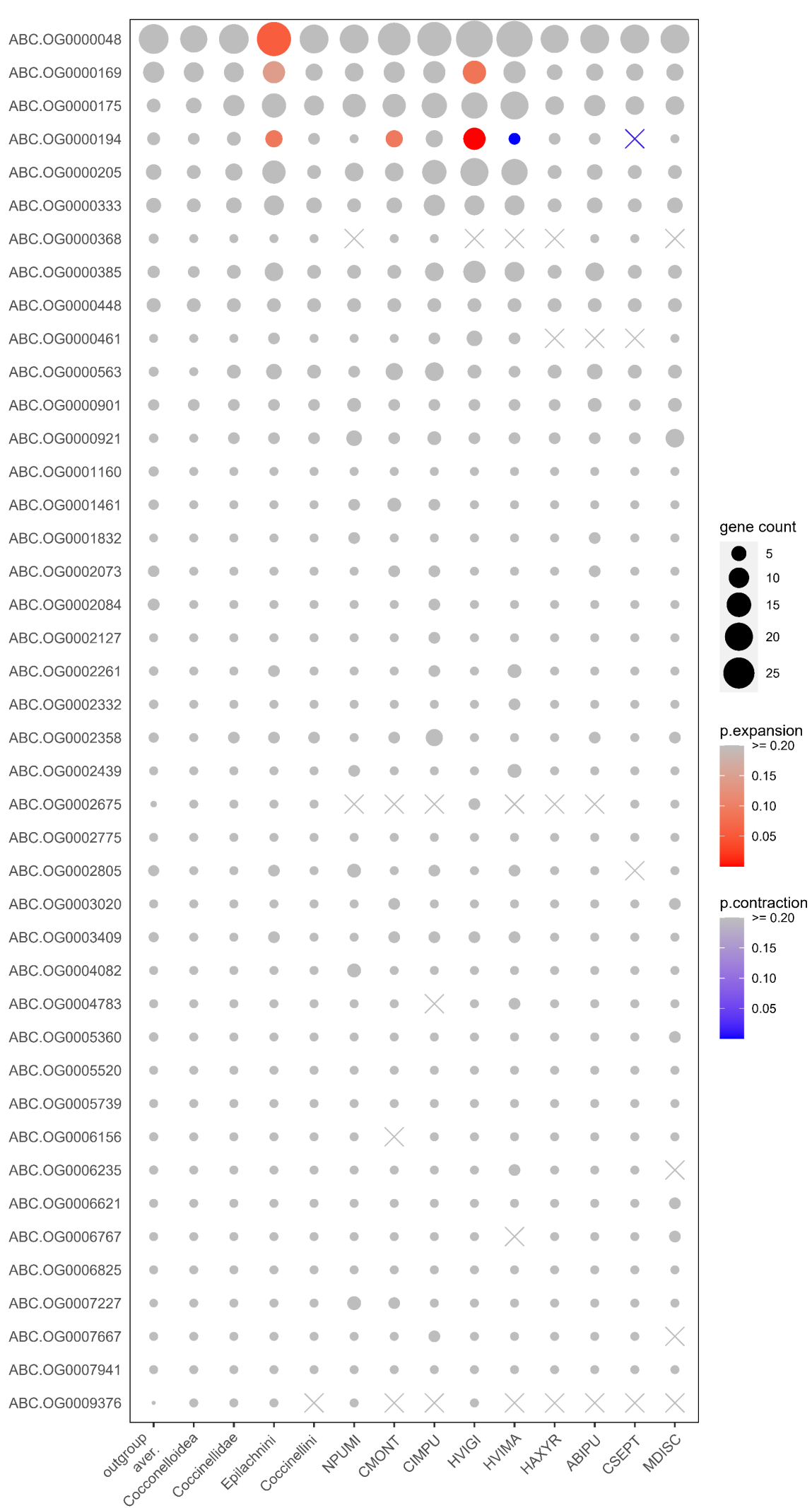


Figure S9.9 Comparison of gene counts in ortholog groups (OGs) of ABCs in the ten ladybird genomes. Only OGs with total genes >= 30 are shown. Gene counts at the nodes of Coccinelloidea, Coccinellidae, Epilachnini and Coccinellini are reconstructed by CAFE. A high-quality figure can be downloaded from https://github.com/huangyh45/ladybird-genomes-supplementary-figures.


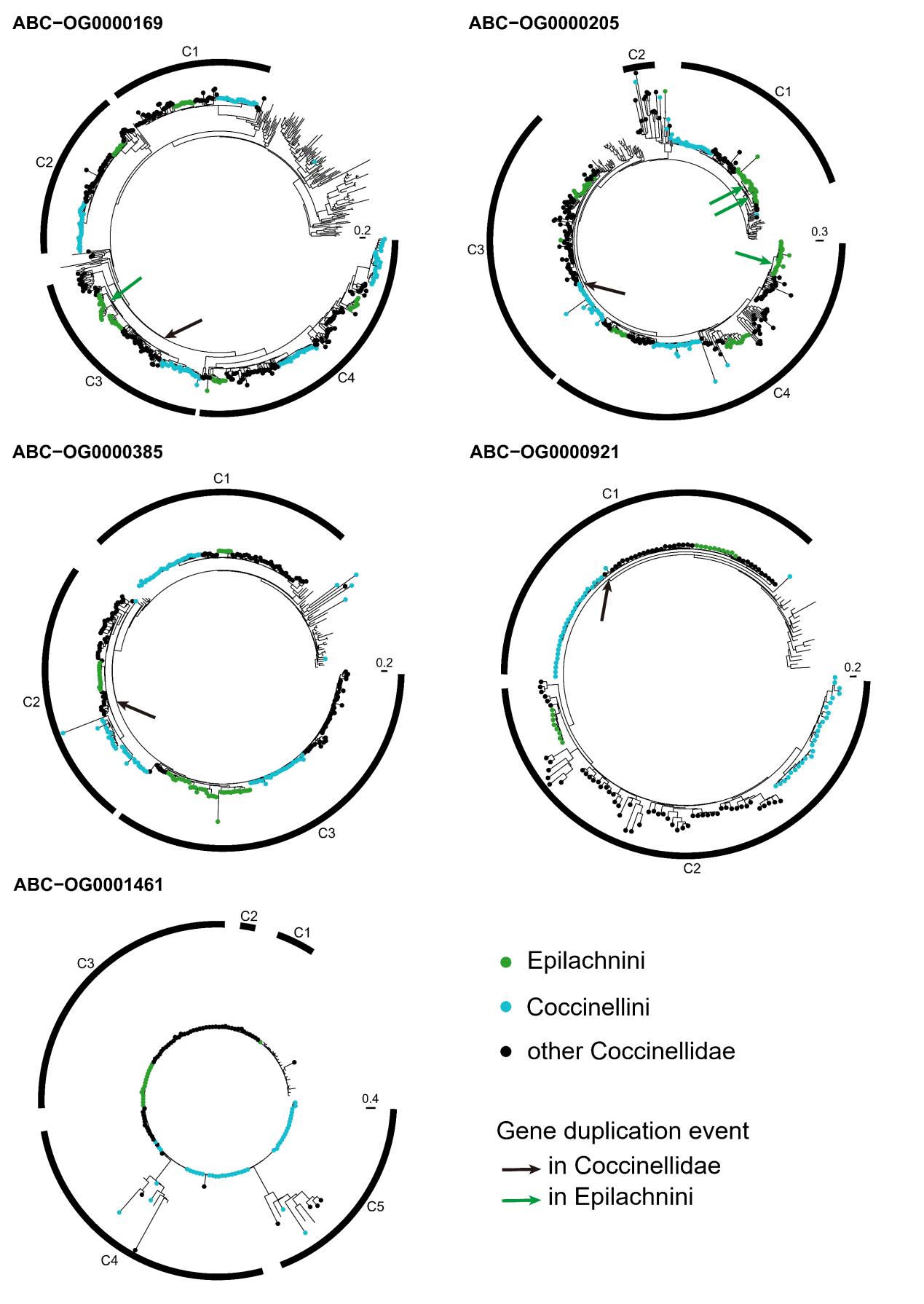


Figure S9.10 Gene trees of ortholog groups (OGs) of ABCs. The genes in the ladybirds are marked in color and the others without circles are the genes in the outgroup beetles. A high-quality figure can be downloaded from https://github.com/huangyh45/ladybird-genomes-supplementary-figures.


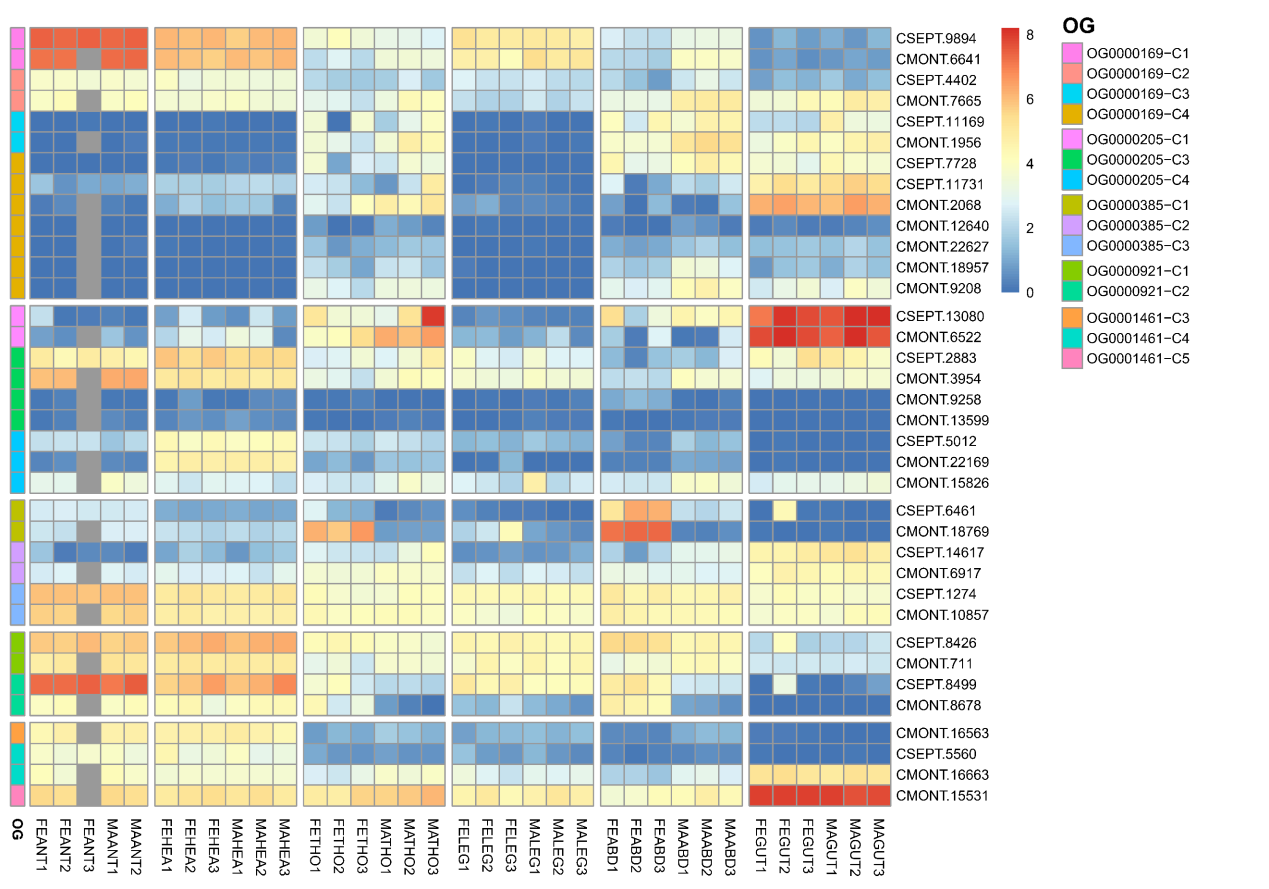


Figure S9.11 Expression patterns of different tissue of each group in ortholog groups (OGs) of ABCs. Abbreviation in the sample names: FE: female adult, MA: male adult, ANT: antenna, HEA: head, THO: thorax, LEG: leg, ABD: abdomen, GUT: gut. A high-quality figure can be downloaded from https://github.com/huangyh45/ladybird-genomes-supplementary-figures.


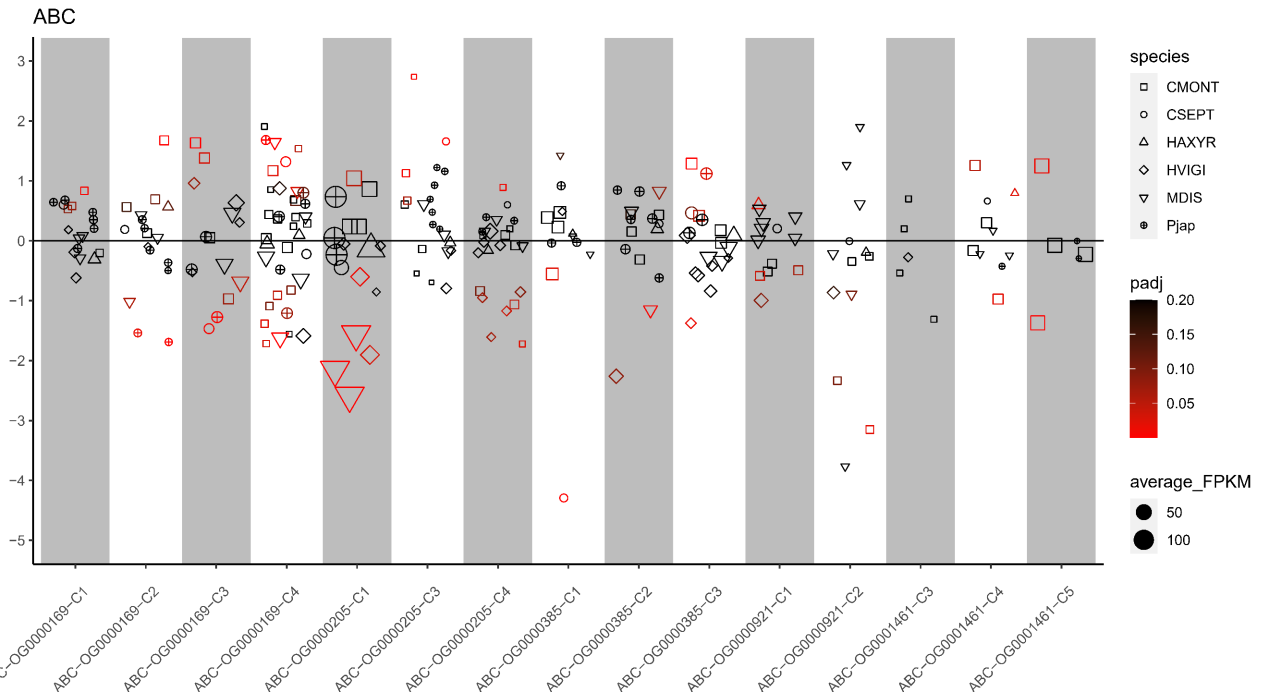


Figure S9.12 Expression patterns under different diet treatments of each group in ortholog groups (OGs) of ABCs. The vertical axis represents the log2(fold change) values. The positive values on the vertical axis represent upregulation when feeding on the optimal diets, while the negative values represent upregulation when feeding on the non-optimal diets. A high-quality figure can be downloaded from https://github.com/huangyh45/ladybird-genomes-supplementary-figures.


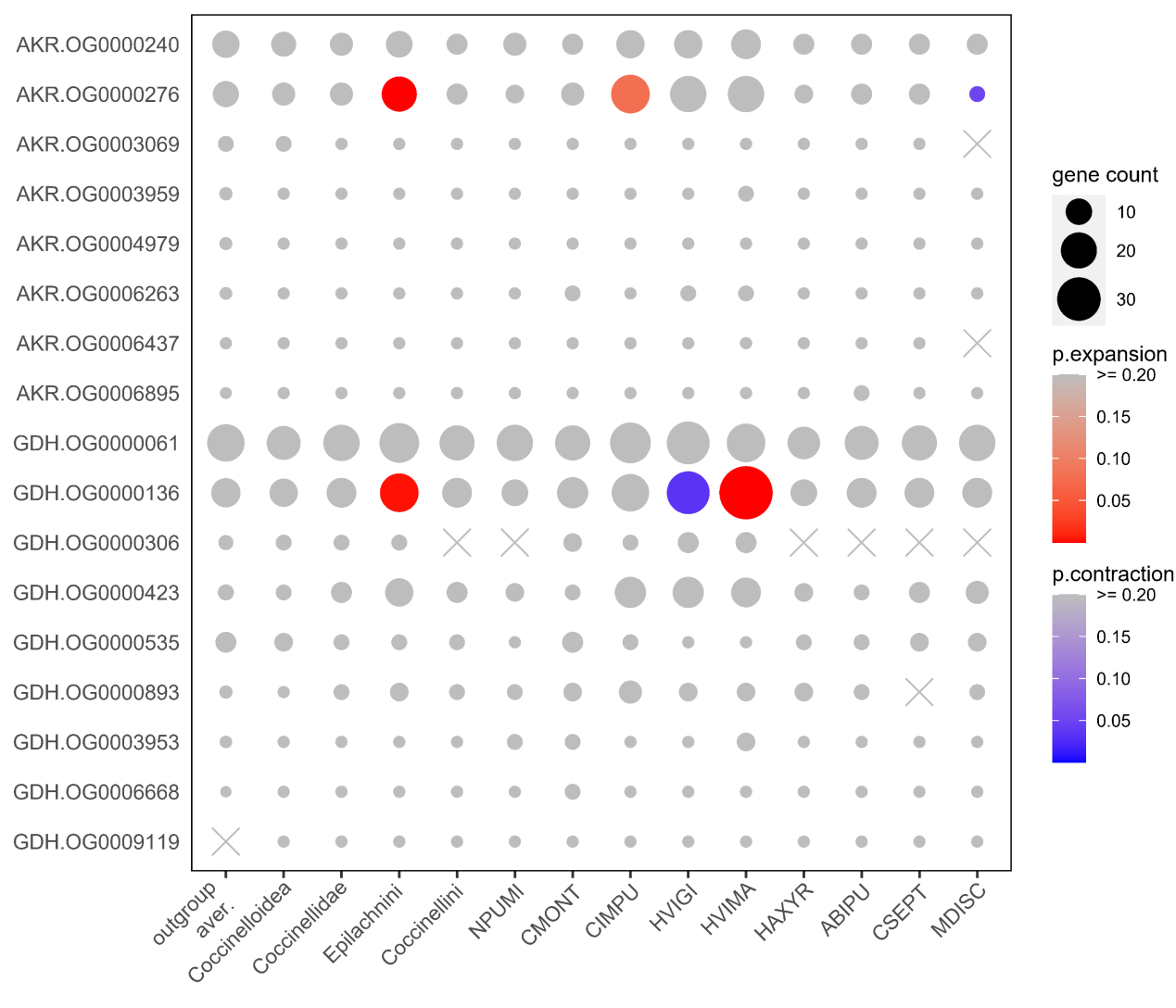


Figure S9.13 Comparison of gene counts in ortholog groups (OGs) of AKRs and GDHs in the ten ladybird genomes. Only OGs with total genes >= 30 are shown. Gene counts at the nodes of Coccinelloidea, Coccinellidae, Epilachnini and Coccinellini are reconstructed by CAFE. A high-quality figure can be downloaded from https://github.com/huangyh45/ladybird-genomes-supplementary-figures.


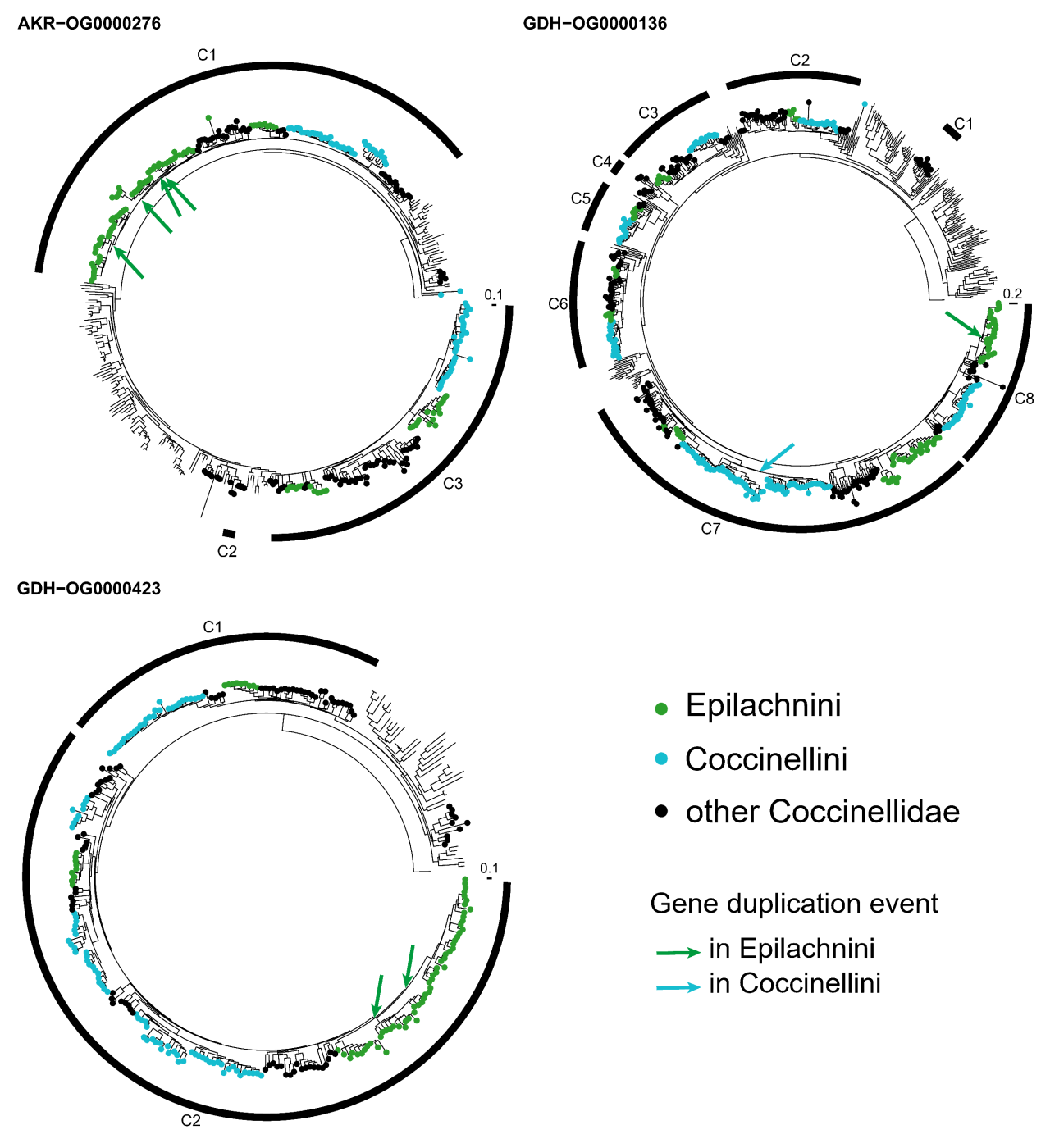


Figure S9.14 Gene trees of ortholog groups (OGs) of AKRs and GDHs. The genes in the ladybirds are marked in color and the others without circles are the genes in the outgroup beetles. A high-quality figure can be downloaded from https://github.com/huangyh45/ladybird-genomes-supplementary-figures.


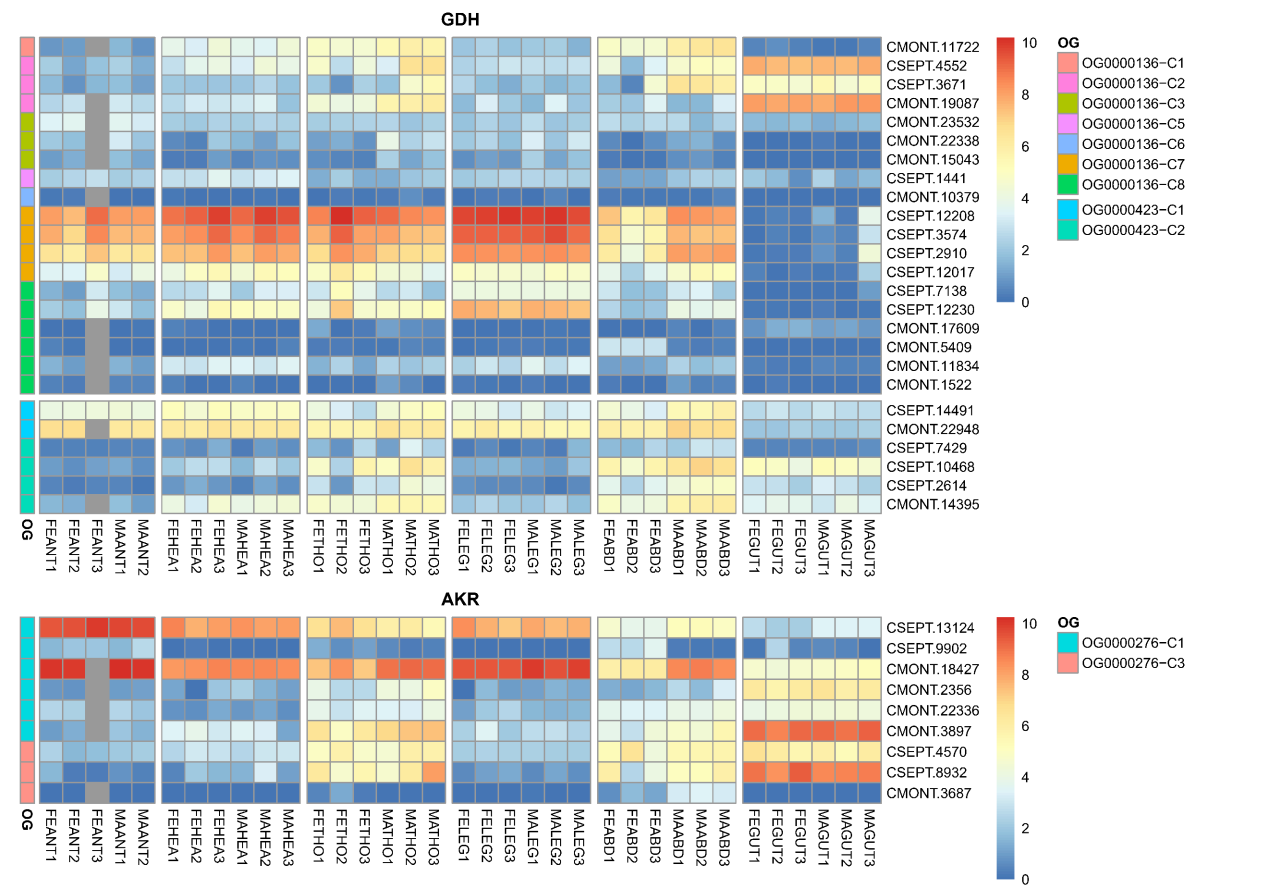


Figure S9.15 Expression patterns of different tissue of each group in ortholog groups (OGs) of AKRs and GDHs. Abbreviation in the sample names: FE: female adult, MA: male adult, ANT: antenna, HEA: head, THO: thorax, LEG: leg, ABD: abdomen, GUT: gut. A high-quality figure can be downloaded from https://github.com/huangyh45/ladybird-genomes-supplementary-figures.


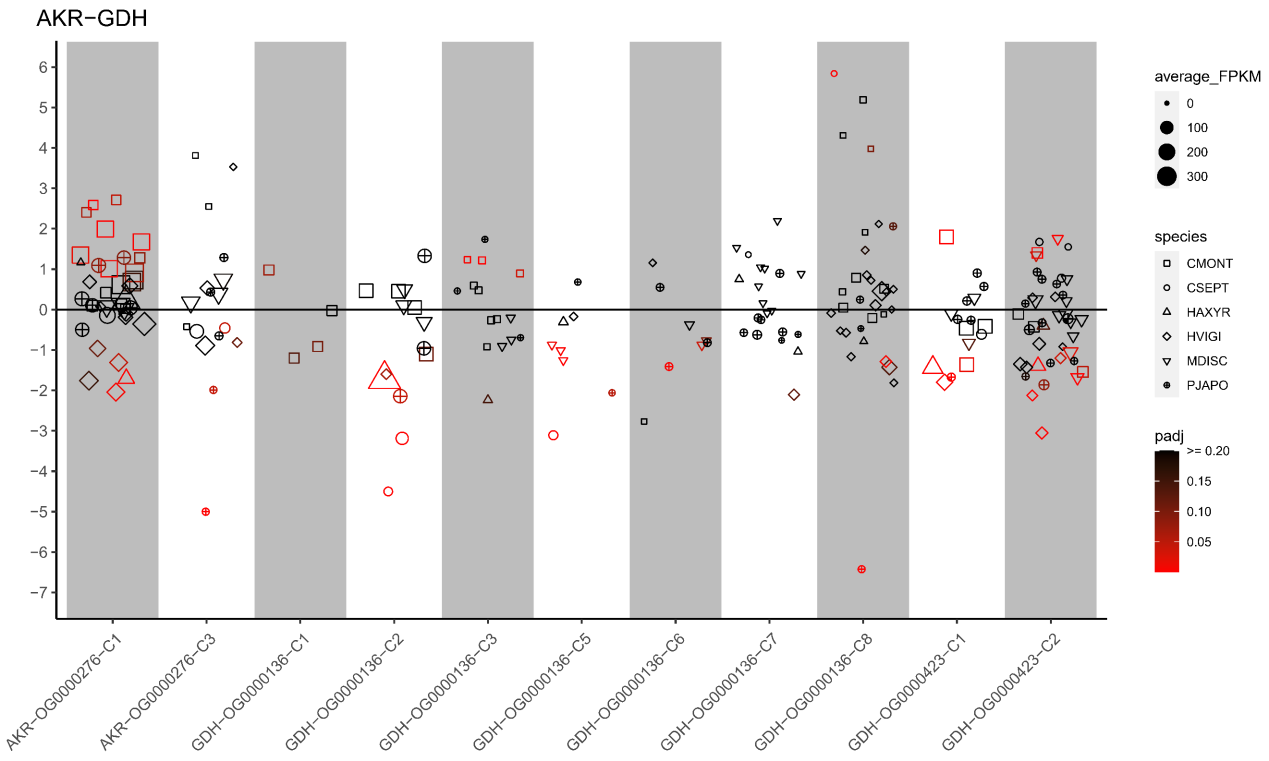


Figure S9.16 Expression patterns under different diet treatments of each group in ortholog groups (OGs) of AKRs and GDHs. The vertical axis represents the log2(fold change) values. The positive values on the vertical axis represent upregulation when feeding on the optimal diets, while the negative values represent upregulation when feeding on the non-optimal diets. A high-quality figure can be downloaded from https://github.com/huangyh45/ladybird-genomes-supplementary-figures.

# 10 Evolution of candidate genes related to immunity

## 10.1 Materials and methods

The methods for genes related to immunity were the same as the methods for chemosensory genes in Section 7.1. The reference genes as the query were the combination of genes of *Drosophila* in immunoDB (Waterhouse et al., 2007) and the immunity-related genes identified from the transcriptome of *H. axyridis* (HAXYR) (Vilcinskas et al., 2013). Additionally, a putative antimicrobial gene, cell wall hydrolase (CWH) found in the Coccinellinae (Li et al., 2021b), was also included in the reference genes. These candidate immunity genes were listed in Table S10.1, from which those antimicrobial peptides (AMPs) reported in other insects but not found in the ladybirds, such as cecropin and knottin, were excluded.

Figure S10.1 Candidate genes associated with immunity

|  | Gene name | Pfam accession | No. orthogroups with genes >= 30 / total orthogroups |
| --- | --- | --- | --- |
| PGRP | Peptidoglycan recognition protein | PF01510 | 5/8 |
| GNBP | Gram-negative binding protein | PF00722, PF15886 | 4/10 |
| FREP | Fibrinogen-related protein | PF00147 | 3/5 |
| CTL | C-type lectin | PF00084, PF00059 | 6/11 |
| ATT | Attacin | PF03768, PF03769 | 1/1 |
| DEFE | Defensin | PF01097 | 2/4 |
| COL | Coleoptericin | PF06286 | 1/1 |
| ILYS | I-type lysozyme | PF05497 | 3/7 |
| CLYS | C-type lysozyme | PF00062 | 2/2 |
| CWH | Cell wall hydrolase | PF07486 | 1/2 |
| SPI | Serine protease inhibitor (serpin) | PF00079 | 9/28 |

## 10.2 Results

With the previously reported immunity-related genes from *Drosophila* and HAXYR (Vilcinskas et al., 2013; Waterhouse et al., 2007), we identified and analyzed the OGs related to immunity using the genome dataset and the extended dataset. Details of identification of OG and the expression patterns of their genes can be found in Additional file 2: Table SE6 and SE5 and the corresponding node numbers are shown in Figure S3.1.

### 10.2.1 Recognition protein: PGRP, GNBP, FREP, CTL

Our immunity-related gene identification found five PGRP OGs, four GNBP OGs, three FREP OGs and six CTL OGs with genes >= 30. But no OGs are under emergence, loss, significant expansion or contraction in Coccinellidae, Coccinellini or Epilachnini (Figure S10.1). In PGRPs and GNBPs, the genes tend to be downregulated when the carnivorous ladybirds feed on non-optimal diets, while large amounts of the genes of CTLs are detected as diet-specific DEGs, including both upregulated and downregulated genes (Figure S10.5).

In OG0001879 of GNBP, genes of Epilachnini are duplicated, with 2/3 downregulated DEGs in sugar water-fed HVIGI (Figure S10.2, S10.4). Additionally, OG0001967 of FREP and OG0002820 of CTL (locomotion-related protein Hikaru genki-like) has a clade losing Coccinellini respectively. In contrast, OG0002820 also has a Coccinellini-specific clade. And OG0009397 of PGRP contains a clade without Epilachnini species. However, no diet-specific DEGs are detected in these four clades.

### 10.2.2 Antimicrobial peptide: Attacin, Defensin, Coleoptericin

For three types of AMPs, the genes are clustered into few OGs (gene members >= 30), including one attacin OG (OG0000273), two defensin OGs (OG0001269 and OG0009673) and one coleoptericin OG (OG0000458). Large counts of the genes of the three AMPs are extremely downregulated under non-optimal diet treatments (Figure S10.5). However, no significant expansion or contraction in Coccinellidae, Coccinellini or Epilachnini is detected (Figure S10.1).

-Defensin

Only OG0009673 is emergent in Coccinellidae, containing one gene in each species except NPUMI (2 genes). The genes in MDISC and HAXYR in OG0009673 are differentially expressed under different diet treatments (Figure S10.4). In the genes of Epilachnini species, however, the domains of defensin are lost, mainly led by replacement from motif 2 to motif 6 (Figure S10.6).

-Attacin

In OG0000273, Clade C5 lost genes of Coccinellini species, with 3/4 downregulated genes in CMONT as diet-specific DEGs when the ladybirds eat APHID and MOTHEGG instead of MEALYBUG (Figure S10.4). Clade C5 has a specific motif 9, which is replaced by motif 10 in Epilachnini genes in Clade C1 and C8, and lost in most genes of Clade C10 (Figure S10.7).

-Coleoptericin

And OG0000458 of coleoptericin significantly expands in MDISC and contracts in CSEPT. In OG0000458, the largest group, Clade C4 has arrangement of motif 5, motif 7/8 and motif 3, which is not found in other ladybird genes or genes from outgroup species (figure S10.8). Most diet-specific DEGs of coleoptericin belong to this group.

### 10.2.3 Lysozyme and Cell Wall Hydrolase

-ILYS, CLYS

The two types of lysozymes, I-type lysozyme (ILYS) and C-type lysozyme (CLYS), have three OGs and two OGs (gene members >= 30) identified, respectively. OG0009100 and OG0009356 (neurogenic locus Notch protein-like) of ILYS are emergent in Coccinellidae, including 2/2 downregulated DEGs in CMONT, 1/1 downregulated DEG in CSEPT and 2/2 downregulated DEGs in MDISC when the ladybirds feed on non-optimal diets in only OG0009100. In OG0002153 of CLYS, an Epilachnini-specific clade is detected, but no diet-specific DEG is found in the clade. A gene in this OG (HAXYR.13647) has been reported without muramidase activity (Beckert et al., 2015) (Table S10.2), which also support that OG0002153 contributes little to immunity and diet adaptation.

-CWH

The CWH OG with 144 gene members, OG0001441, is a ladybird LEGF emergent at the ancestor of Coccinellinae, which conforms to the previous result that this gene was horizontally transferred from bacteria to the ladybird ancestor (Li et al., 2021b). Genes of OG0001441 are mainly gut-specific and include 1/2 downregulated DEGs in CMONT, 2/5 downregulated DEGs in PJAPO, 1/4 upregulated DEGs in HAXYR, 1/2 downregulated DEGs in CSEPT, 2/5 upregulated DEGs in MDISC and 1/3 downregulated DEGs in HVIGI under non-optimal diet treatments. However, the diet-specific DEGs in OG0001441 are not placed in the same clade in the gut-specific clade.

Generally, similar with AMPs, several lysozyme encoding genes and CWH genes in CMONT, HAXYR, PJAPO and MDISC are detected as downregulated DEGs of different diets (Figure S10.5).

### 10.2.4 Serpin

We identified nine OGs of serpin with at least 30 gene members, including OG0000225 under significant expansion in Coccinellidae and Epilachnini and contraction in Coccinellini and MDISC (Figure S10.1). A gene duplication event in Epilachnini occurs in Clade C1 of OG0000225 (Figure S10.2). The OG includes 5/19 downregulated DEGs in HVIGI feeding on sugar water, which are all in Clade C1 (Figure S10.4). 2/16 genes in CIMPU are highly expressed in gut, and some genes in CMONT and CSEPT are highly expressed in abdomen or head (Figure S10.3). In addition, OG0011107 and OG0014613 are emergent in Epilachnini, with 1/7 downregulated DEG in HVIGI under sugar water treatment in OG0011107.

Serpins negatively regulate insect innate immunity via inhibition of serine proteinase cascades that initiate immune responses such as melanization and antimicrobial peptide production, and respond to pathogen in the insects (Meekins et al., 2017; Shakeel et al., 2019). Expansion of serpins in Coccinellidae indicates potential demand to regulate immunity when adapting to new prey. The drastic downregulations of AMPs towards the non-optimal diets above also support the connection between expansion of serpins and the immunity demand during the prey adaptation. However, most obviously, serpins expand in Epilachnini, and part of them are highly expressed in the gut of the herbivorous ladybirds and detected as diet-specific DEGs in HVIGI. It has been reported that serpins can also be expressed in the midgut and act on furin pathway to regulate secretion (Meekins et al., 2017). Therefore, it seems that serpins possibly play an important role in plant diet adaptation in the ladybirds, probably through immunity regulation to manage complicated pathogen from the plants, or secretion regulation to control the relatively various digestive proteins for plant diet. Large amounts of serpins expressed in the midgut of other herbivorous insects (e.g. the tobacco hornworm (Pauchet et al., 2010)) also support their importance in herbivory. In consideration of the target genes of serpins, mainly SPs mentioned in Section 8.2.1, OG0000255 of SP also expands in Coccinellidae and Epilachnini and genes in OG0000255 are mainly expressed in abdomen with high expression, which indicates most likely potential connection to OG0000225. But OG0000255 is not considered as digestive enzymes. Both function of these two OGs and their potential connection need to be verified and explored in the experiments.

## 10.3 Summary

We conducted the analysis on the immunity-related OGs, including the recognition proteins (PGRP, GNBP, FREP and CTL), the immune effectors (attacin, defensin, coleoptericin, lysozymes and CWH) and serpins. Notably, we found large amounts of immunity-related genes differentially expressed when the ladybirds feed on non-optimal diets, especially MOTHEGG, which leads to most downregulations of the immune effector genes. Additionally, several immune effector OGs emerged at the ancestor of Coccinellidae or Coccinellinae, such as defensin, ILYS and CWH. Evolution of the AMPs, including attacins, defensins and coleoptericins, mainly occurs with motif-level replacements or loss. And OG0000225 of serpins are detected with expansion in Coccinellidae or Epilachnini.

Most ladybirds are carnivorous and mainly prey on the insects in the suborder Sternorrhyncha (Hemiptera), such as aphids, coccids (e.g. mealybugs and scale insects), psyllids and whiteflies. These sap-feeding insects are known to have a specific bacteriome that harbors diverse symbiont bacteria, for example, *Tremblaya* and *Moranella* in the mealybugs, and *Buchnera* in the aphids (Baumann, 2005). Other symbiont bacteria also widely exist in Sternorrhyncha and may have an impact on the carnivorous ladybirds, such as *Serratia symbiotica* in the aphids (Du et al., 2022). The symbiont bacteria of these insects may protect their hosts from carnivorous, as reported in several insects (Brownlie and Johnson, 2009; McLean, 2019), and the ladybirds thus probably require the ability to manage these symbiont bacteria or control them in a specific region. The expression response of the immunity-related genes, mainly the immune effector genes, which can enhance immunity of the ladybirds, seem reasonable under this drive.

Table S10.2 The reported function of the candidate immunity-related genes in the ladybirds.

| Reported gene name | Gene ID | Ortholog group | Gene family | High expression in qPCR | Functional verification | Reference |
| --- | --- | --- | --- | --- | --- | --- |
| Haxy_C-Lys1 | HAXYR.9764 | OG0002153 | CLYS | - | - | Vilcinskas et al. (2013) |
| Haxy_C-Lys2 | HAXYR.13647 | OG0002153 | CLYS | - | no muramidase activity | Beckert et al. (2015); Vilcinskas et al. (2013) |
| Haxy_C-Lys3 | HAXYR.12434 | OG0000465 | CLYS | slightly inducible in the guts by immune challenge | muramidase activity | Beckert et al. (2015); Vilcinskas et al. (2013) |
| Haxy_C-Lys4 | HAXYR.11629 | OG0000465 | CLYS | highly inducible in the guts by immune challenge | muramidase activity | Beckert et al. (2015); Vilcinskas et al. (2013) |
| Haxy_i-Lys1 | HAXYR.9014 | OG0000441 | ILYS | - | - | Vilcinskas et al. (2013) |
| Haxy_i-Lys2 | HAXYR.13741 | OG0000441 | ILYS | fat body, but not inducible by immune challenge | no muramidase, isopeptidase and serine hydrolase activity | Beckert et al. (2016); Vilcinskas et al. (2013) |
| Haxy_i-Lys3 | HAXYR.1451 | OG0001109 | ILYS | - | - | Vilcinskas et al. (2013) |
| Haxy_i-Lys4 | HAXYR.4224 | OG0001109 | ILYS | - | - | Vilcinskas et al. (2013) |
| Haxy_i-Lys5 | HAXYR.11408 | OG0009100 | ILYS | - | - | Vilcinskas et al. (2013) |
| Haxy_i-Lys6 | HAXYR.10485 | OG0009356 | ILYS | - | - | Vilcinskas et al. (2013) |
| Haxy_Col9 | HAXYR.5271 | OG0000458 | COL | - | - | Vilcinskas et al. (2013) |
| Haxy_Col8 | HAXYR.5271 | OG0000458 | COL | highly immune‐induced | - | Vilcinskas et al. (2013) |
| Haxy_ColLB | HAXYR.4935 | OG0000458 | COL | immune‐induced | - | Vilcinskas et al. (2013) |
| Haxy_ColLA | HAXYR.4935 | OG0000458 | COL | - | - | Vilcinskas et al. (2013) |


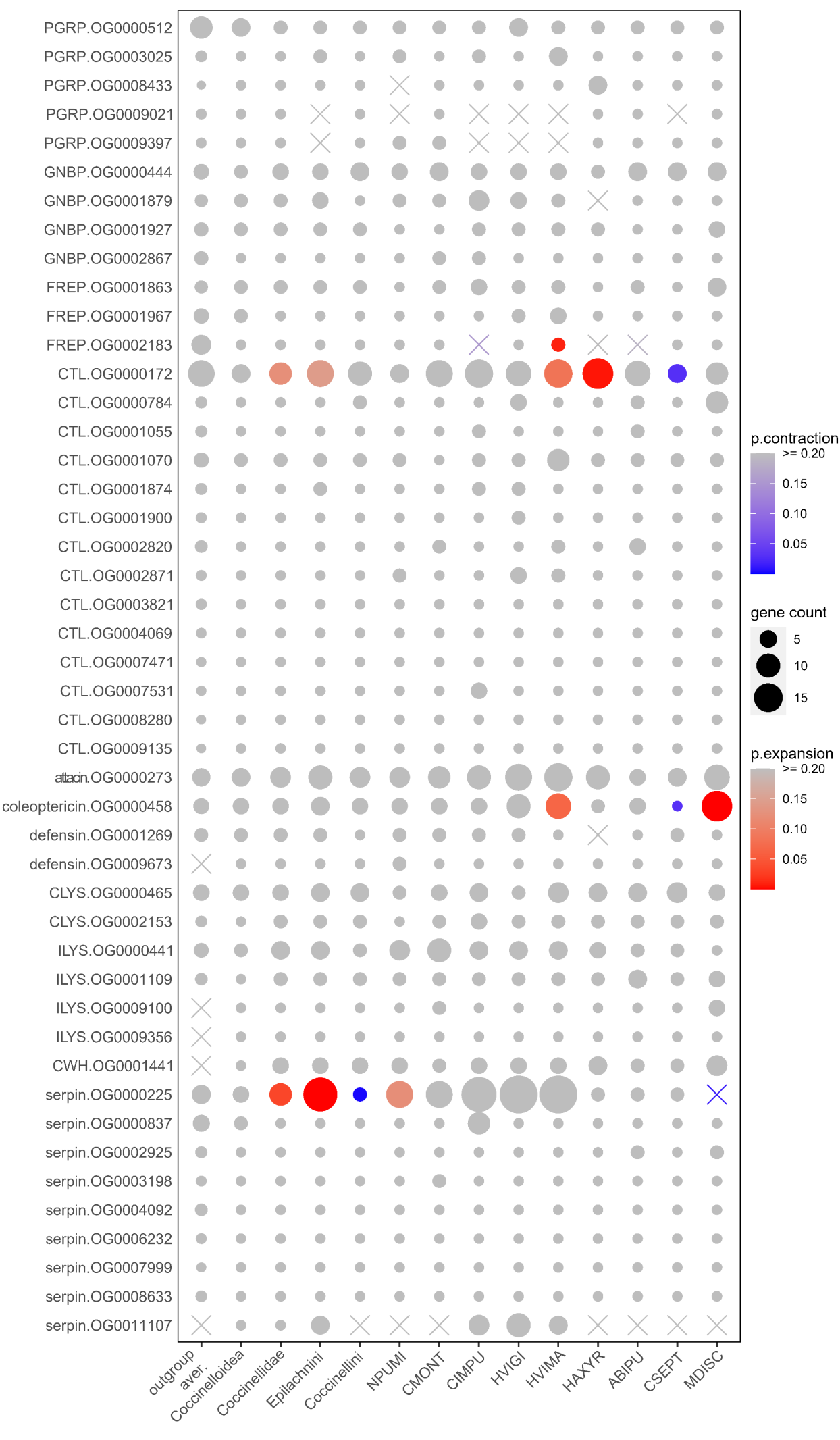


Figure S10.1 Comparison of gene counts in ortholog groups (OGs) related to immunity in the ten ladybird genomes. Only OGs with total genes >= 30 are shown. Gene counts at the nodes of Coccinelloidea, Coccinellidae, Epilachnini and Coccinellini are reconstructed by CAFE. A high-quality figure can be downloaded from https://github.com/huangyh45/ladybird-genomes-supplementary-figures.


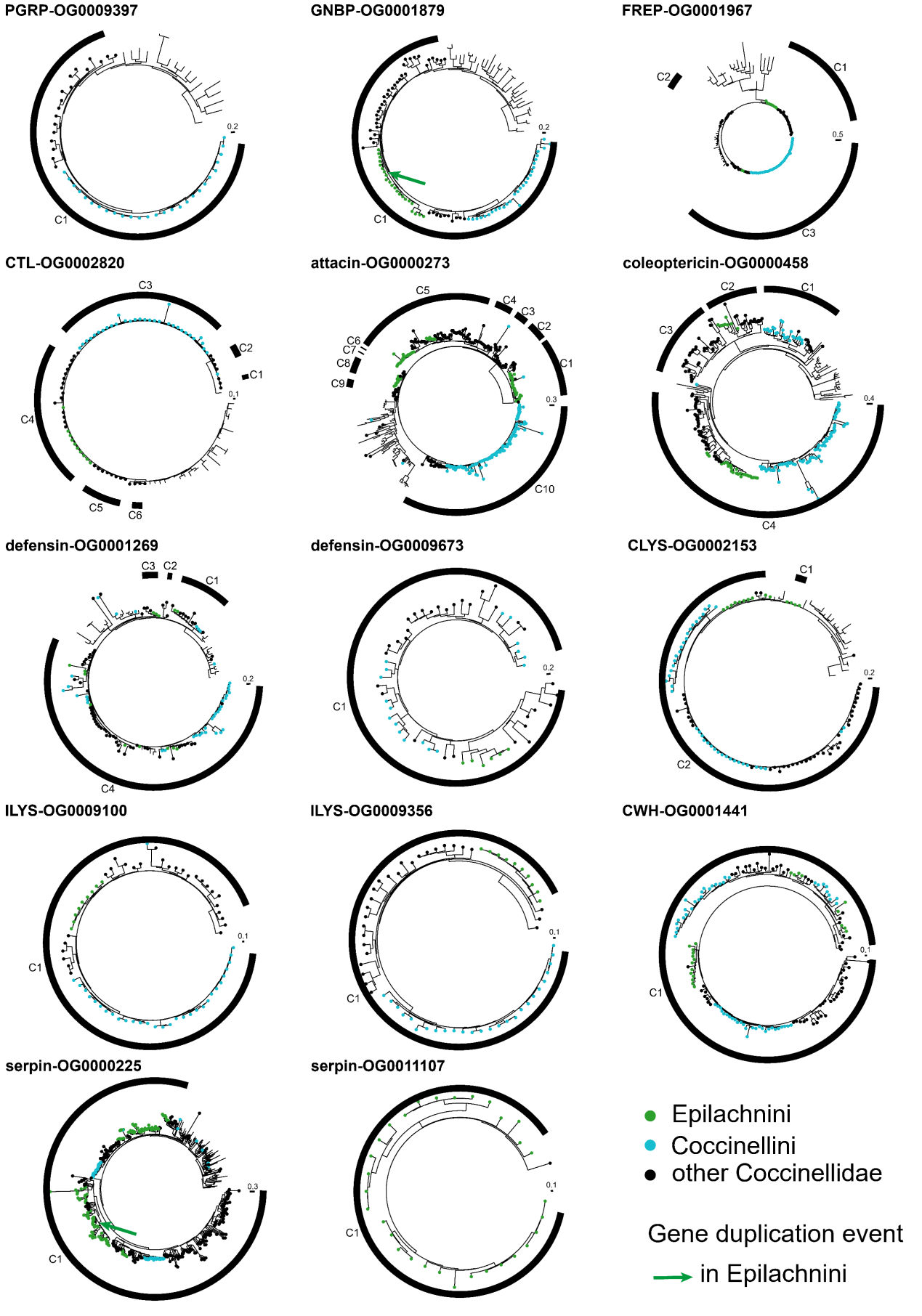


Figure S10.2 Gene trees of ortholog groups (OGs) related to immunity. The genes in the ladybirds are marked in color and the others without circles are the genes in the outgroup beetles. A high-quality figure can be downloaded from https://github.com/huangyh45/ladybird-genomes-supplementary-figures.


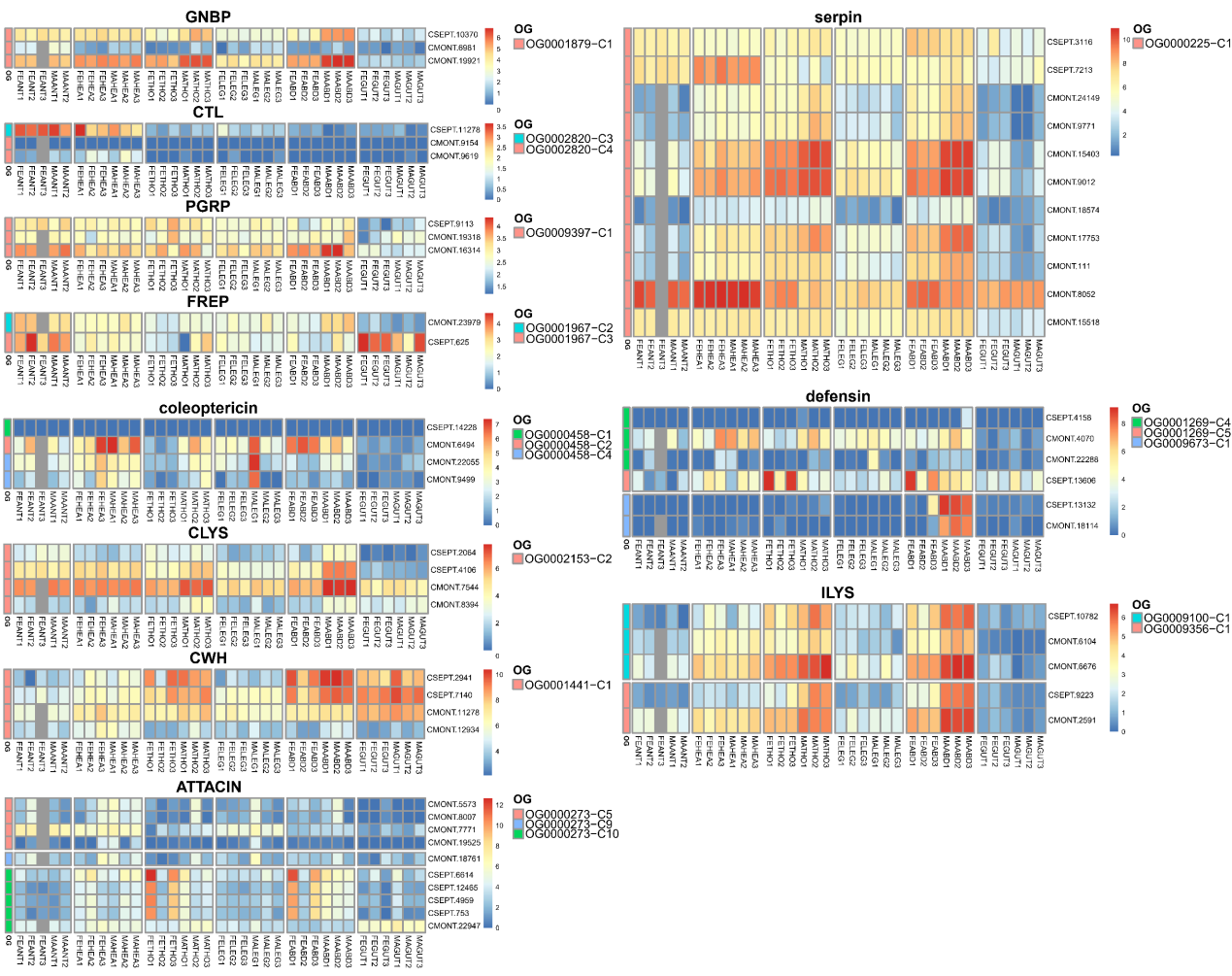


Figure S10.3 Expression patterns of different tissue of each group in ortholog groups (OGs) related to immunity. Abbreviation in the sample names: FE: female adult, MA: male adult, ANT: antenna, HEA: head, THO: thorax, LEG: leg, ABD: abdomen, GUT: gut. A high-quality figure can be downloaded from https://github.com/huangyh45/ladybird-genomes-supplementary-figures.


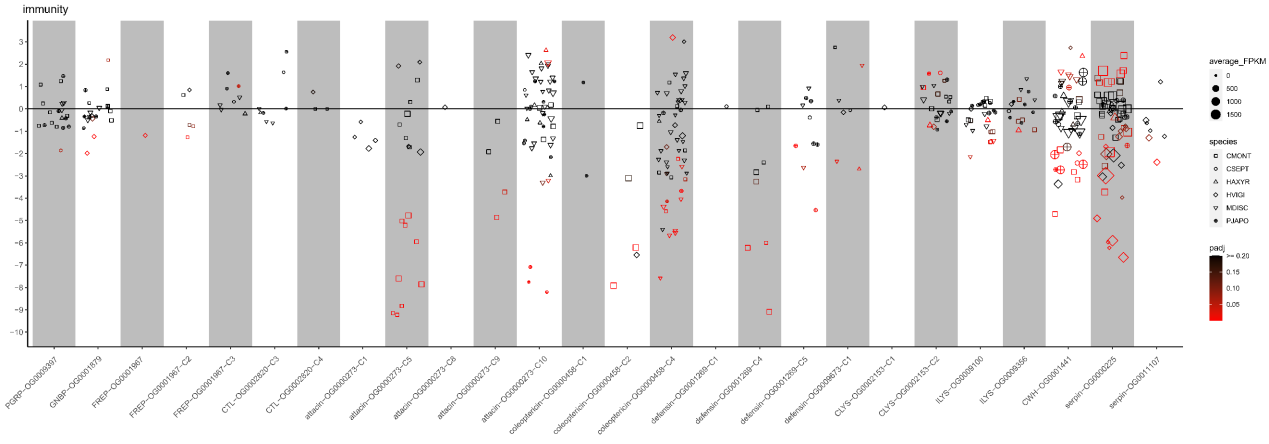


Figure S10.4 Expression patterns under different diet treatments of each group in ortholog groups (OGs) related to immunity. The vertical axis represents the log2(fold change) values. The positive values on the vertical axis represent upregulation when feeding on the optimal diets, while the negative values represent upregulation when feeding on the non-optimal diets. A high-quality figure can be downloaded from https://github.com/huangyh45/ladybird-genomes-supplementary-figures.


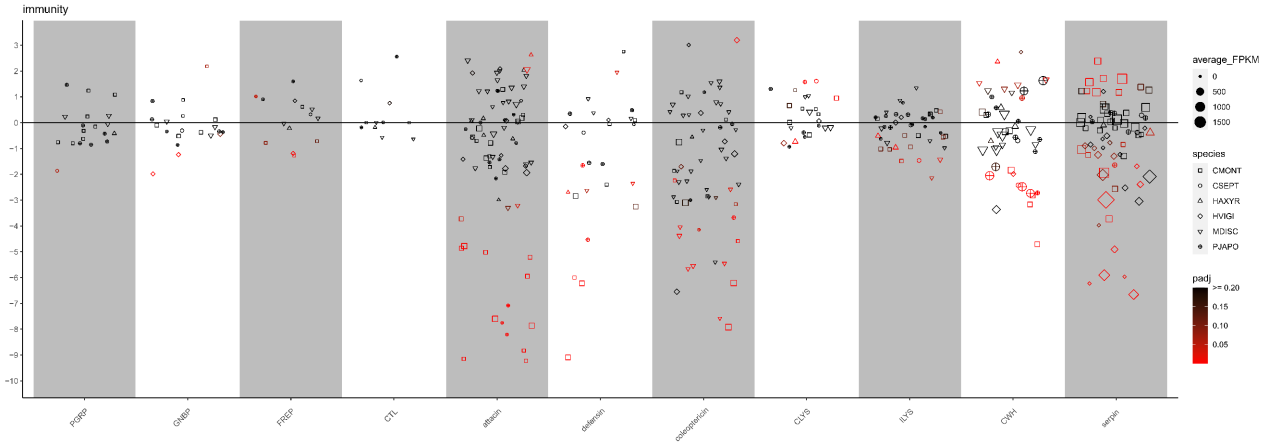


Figure S10.5 Expression patterns under different diet treatments of each immunity-related genes. The vertical axis represents the log2(fold change) values. The positive values on the vertical axis represent upregulation when feeding on the optimal diets, while the negative values represent upregulation when feeding on the non-optimal diets. A high-quality figure can be downloaded from https://github.com/huangyh45/ladybird-genomes-supplementary-figures.


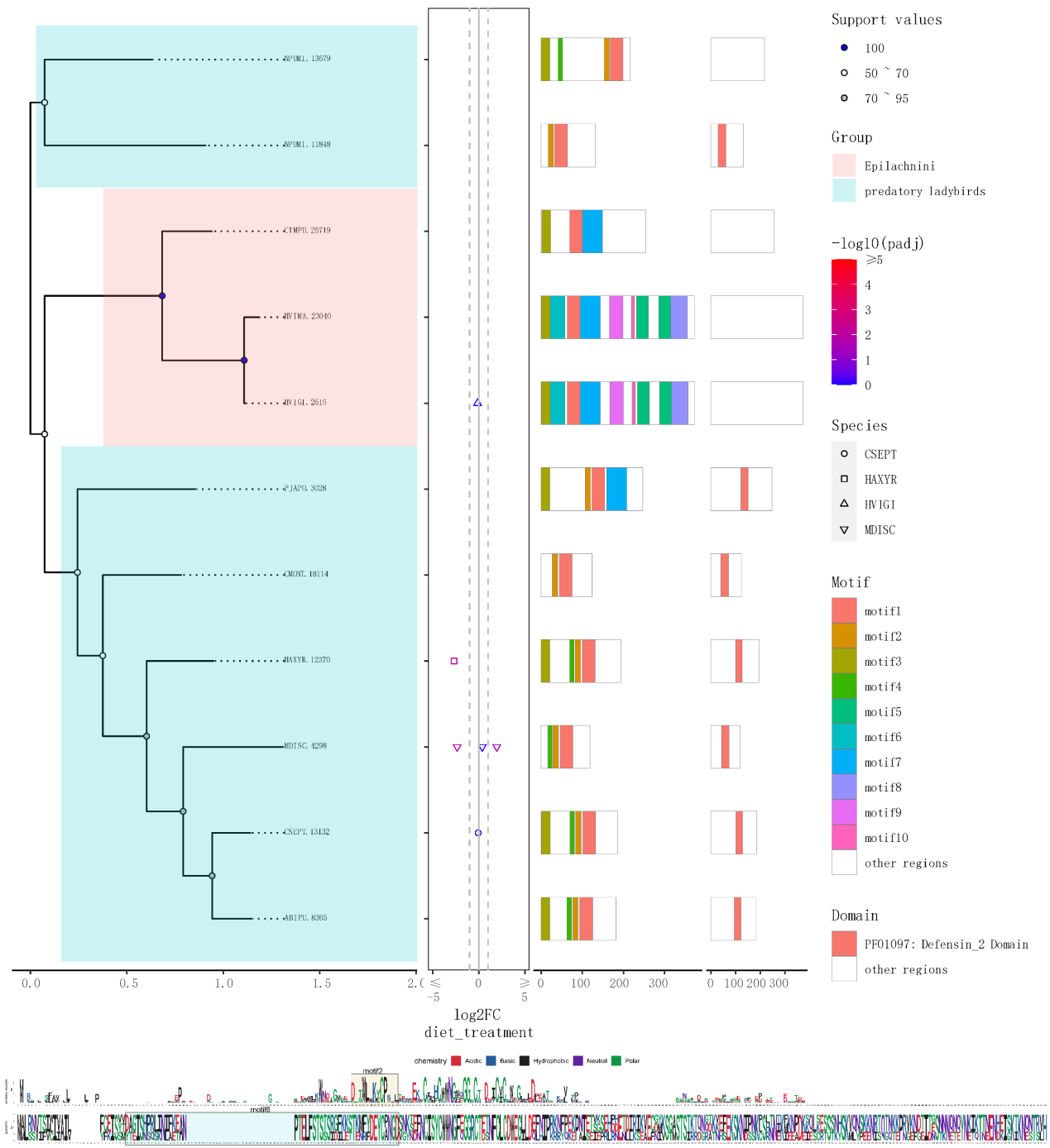


Figure S10.6 Phylogeny and sequence composition of OG0009673 of defensin. A high-quality figure can be downloaded from https://github.com/huangyh45/ladybird-genomes-supplementary-figures.


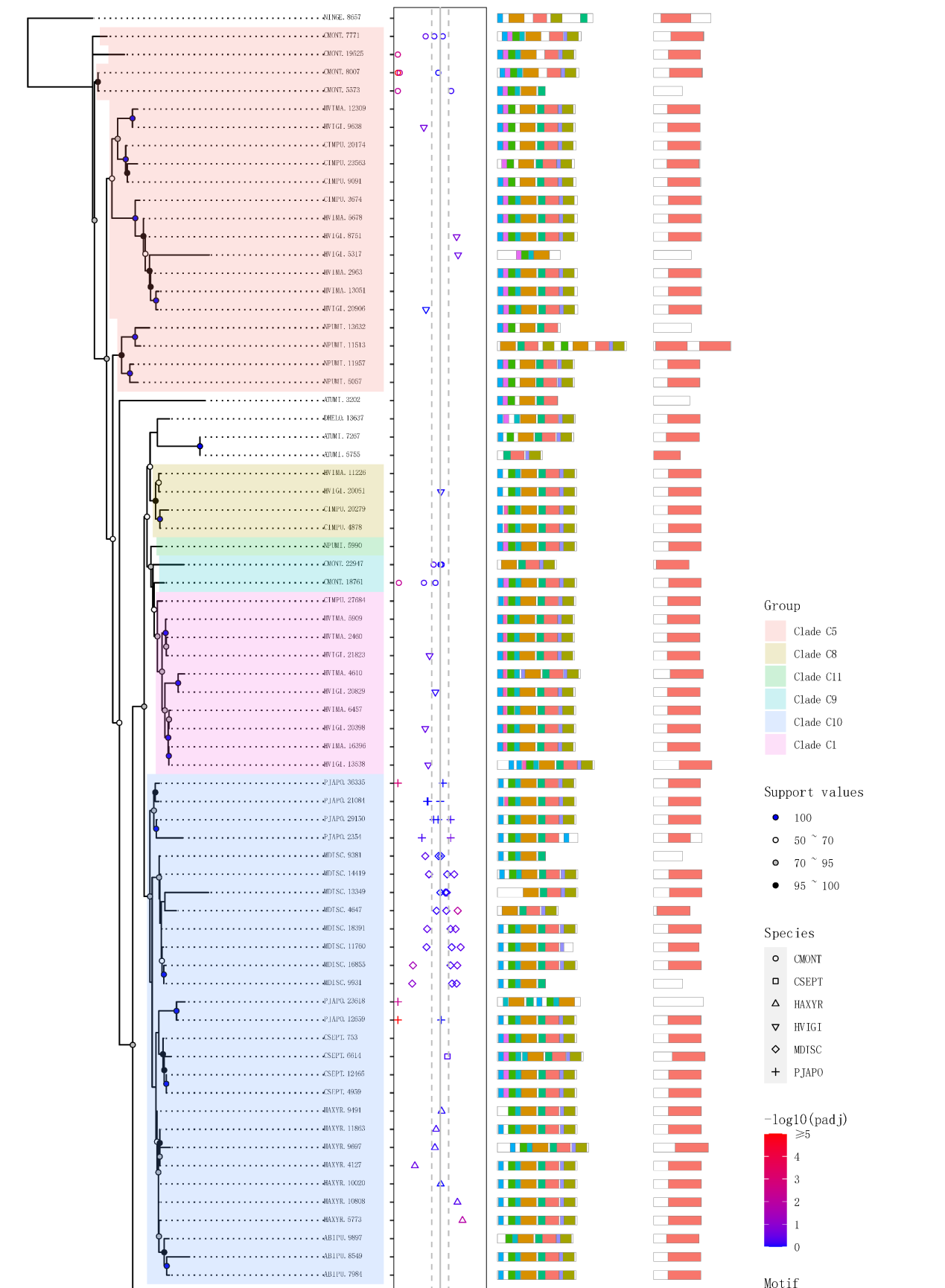


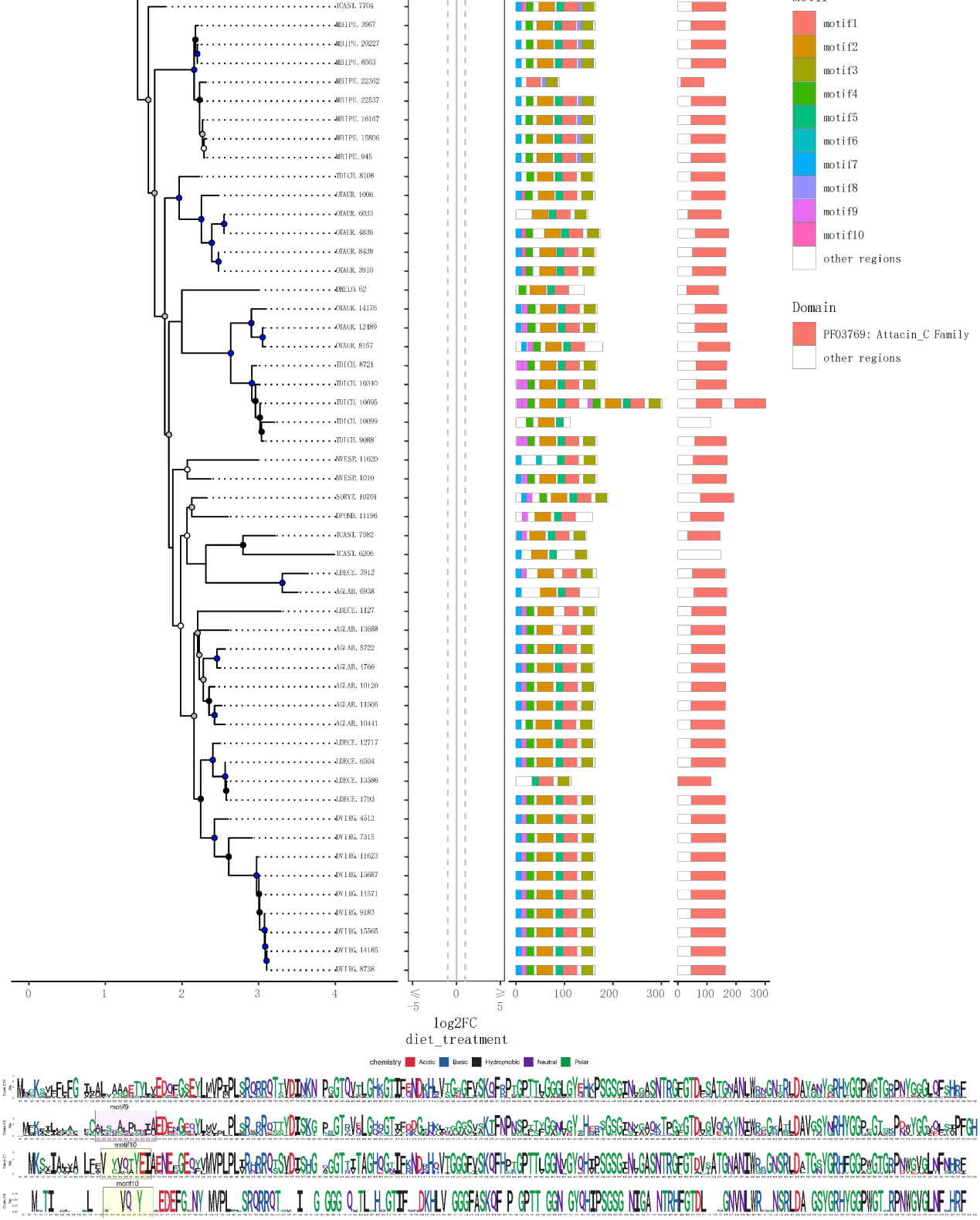


Figure S10.7 Phylogeny and sequence composition of OG0000273 of attacin. A high-quality figure can be downloaded from https://github.com/huangyh45/ladybird-genomes-supplementary-figures.


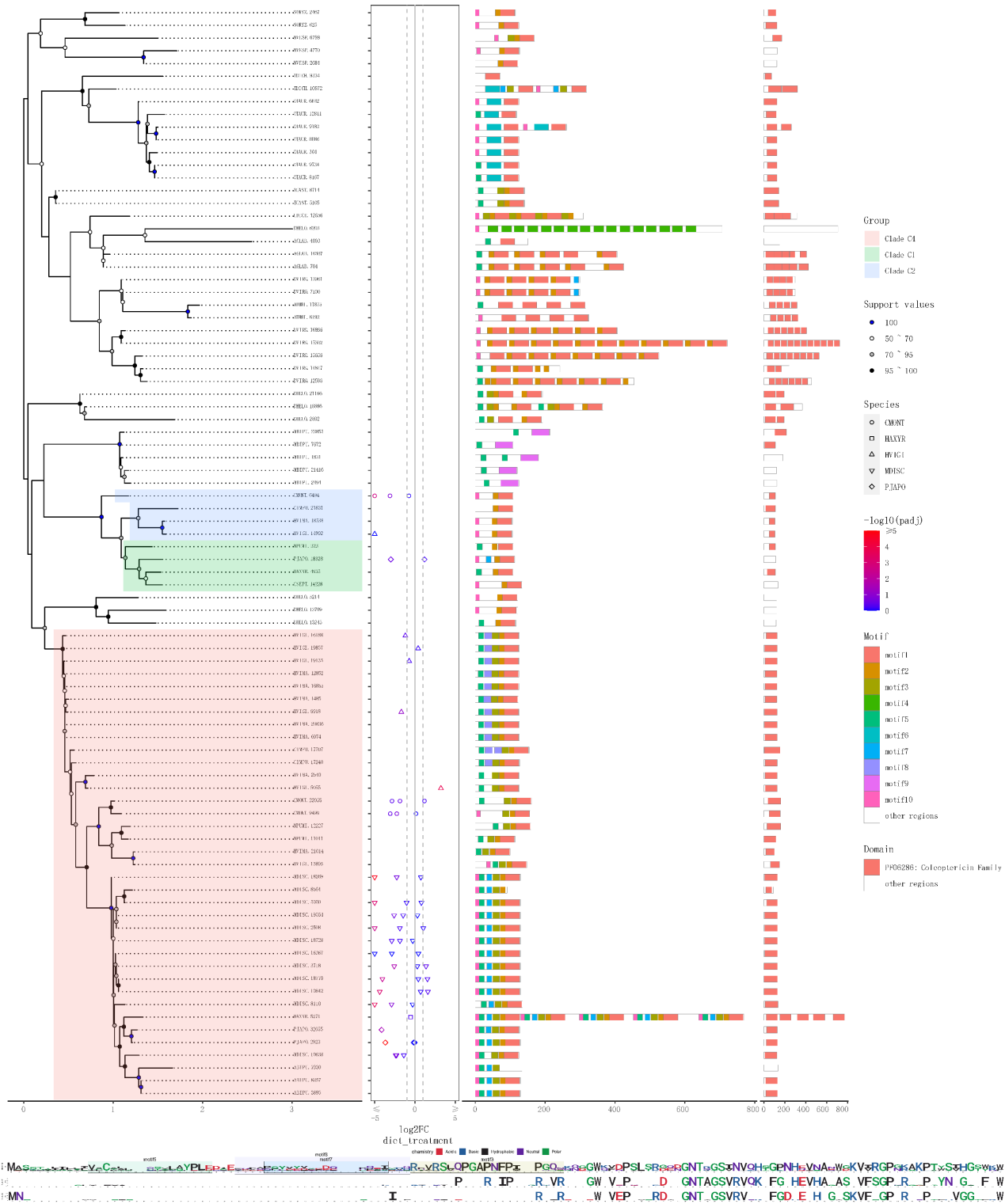


Figure S10.8 Phylogeny and sequence composition of OG0000458 of coleoptericin. A high-quality figure can be downloaded from https://github.com/huangyh45/ladybird-genomes-supplementary-figures.

# Excel table information

Tables in Excel Additional file:

Additional file 2: Table SE1 Information of Coccinellidae and other Coleoptera taxa used in this study

Additional file 2: Table SE2 Information of transcriptome used in this study

Additional file 2: Table SE3 Ortholog groups (OGs) under evolution events based on our genomic and transcriptomic data

Additional file 2: Table SE4 Ortholog group (OG) enrichment results of differentially expressed genes (DEGs) in six carnivorous ladybird species under different diet treatments

Additional file 2: Table SE5 Expression patterns of genes in the diet-specific and tissue specific transcriptome comparisons

Additional file 2: Table SE6 Candidate ortholog group (OG) related to chemosensation, digestion, detoxification and immunity

# References

Aguirre-Rojas, L.M., Scully, E.D., Trick, H.N., Zhu, K.Y., Smith, C.M., 2021. Comparative analyses of transcriptional responses of *Dectes texanus* LeConte (Coleoptera: Cerambycidae) larvae fed on three different host plants and artificial diet. Scientific Reports 11**,** 11448.

Aizawa, S., Senda, M., Harada, A., Maruyama, N., Ishida, T., Aigaki, T., Ishigami, A., Senda, T., 2013. Structural Basis of the γ-Lactone-Ring Formation in Ascorbic Acid Biosynthesis by the Senescence Marker Protein-30/Gluconolactonase. Plos One 8**,** e53706.

Al Abassi, S., Birkett, M.A., Pettersson, J., Pickett, J.A., Woodcock, C.M., 1998. Ladybird beetle odour identified and found to be responsible for attraction between adults. Cellular and Molecular Life Sciences CMLS 54**,** 876-879.

Alekseev, A.V., 1993. Jurassic and Lower Cretaceous Buprestidae (Coleoptera) from Eurasia. Paleontological Journal 27**,** 9-34.

Allen, M.L., 2015. Characterization of Adult Transcriptomes from the Omnivorous Lady Beetle *Coleomegilla maculata* Fed Pollen or Insect Egg Diet. J Genomics 3**,** 20-28.

Álvarez-Alfageme, F., Ferry, N., Castañera, P., Ortego, F., Gatehouse, A.M.R., 2008. Prey mediated effects of Bt maize on fitness and digestive physiology of the red spider mite predator *Stethorus punctillum* Weise (Coleoptera : Coccinellidae). Transgenic Research 17**,** 943-954.

Ando, T., Matsuda, T., Goto, K., Hara, K., Ito, A., Hirata, J., Yatomi, J., Kajitani, R., Okuno, M., Yamaguchi, K., Kobayashi, M., Takano, T., Minakuchi, Y., Seki, M., Suzuki, Y., Yano, K., Itoh, T., Shigenobu, S., Toyoda, A., Niimi, T., 2018. Repeated inversions within a *pannier* intron drive diversification of intraspecific colour patterns of ladybird beetles. Nature Communications 9**,** 3843.

Arakane, Y., Muthukrishnan, S., 2010. Insect chitinase and chitinase-like proteins. Cellular and Molecular Life Sciences 67**,** 201-216.

Armenteros, J.J.A., Salvatore, M., Emanuelsson, O., Winther, O., von Heijne, G., Elofsson, A., Nielsen, H., 2019a. Detecting sequence signals in targeting peptides using deep learning. Life Science Alliance 2**,** e201900429.

Armenteros, J.J.A., Tsirigos, K.D., Sonderby, C.K., Petersen, T.N., Winther, O., Brunak, S., von Heijne, G., Nielsen, H., 2019b. SignalP 5.0 improves signal peptide predictions using deep neural networks. Nature Biotechnology 37**,** 420-423.

Arnoldi, L.V., 1977. Rhynchopora, in Mezozoiskie zhestkokryiye [Mesozoic Coleoptera]. Trudy Paleontologicheskogo instituta 161**,** 142-176.

Aumiller, J.J., Hollister, J.R., Jarvis, D.L., 2006. Molecular cloning and functional characterization of β-*N*-acetylglucosaminidase genes from Sf9 cells. Protein Expression and Purification 47**,** 571-590.

Azuma, N., Seo, H.-C., Lie, Ø., Fu, Q., Gould, R.M., Hiraiwa, M., Burt, D.W., Paton, I.R., Morrice, D.R., O'Brien, J.S., Kishimoto, Y., 1998. Cloning, expression and map assignment of chicken prosaposin. Biochemical Journal 330**,** 321-327.

Bailey, T.L., Elkan, C., 1994. Fitting a mixture model by expectation maximization to discover motifs in biopolymers. Proceedings of the Second International Conference on Intelligent Systems for Molecular Biology**,** 28-36.

Baumann, P., 2005. Biology of bacteriocyte-associated endosymbionts of plant sap-sucking insects. Annual Review of Microbiology 59**,** 155-189.

Beckert, A., Wiesner, J., Baumann, A., Pöppel, A.-K., Vogel, H., Vilcinskas, A., 2015. Two c-type lysozymes boost the innate immune system of the invasive ladybird *Harmonia axyridis*. Developmental and Comparative Immunology 49**,** 303-312.

Beckert, A., Wiesner, J., Schmidtberg, H., Lehmann, R., Baumann, A., Vogel, H., Vilcinskas, A., 2016. Expression and characterization of a recombinant i-type lysozyme from the harlequin ladybird beetle *Harmonia axyridis*. Insect Molecular Biology 25**,** 202-215.

Bell, C.D., Soltis, D.E., Soltis, P.S., 2010. The Age and Diversification of the Angiosperms Re-Revisited. American Journal of Botany 97**,** 1296-1303.

Beran, F., Pauchet, Y., Kunert, G., Reichelt, M., Wielsch, N., Vogel, H., Reinecke, A., Svatoš, A., Mewis, I., Schmid, D., Ramasamy, S., Ulrichs, C., Hansson, B.S., Gershenzon, J., Heckel, D.G., 2014. *Phyllotreta striolata* flea beetles use host plant defense compounds to create their own glucosinolate-myrosinase system. Proceedings of the National Academy of Sciences of the United States of America 111**,** 7349-7354.

Biddinger, D.J., Weber, D.C., Hull, L.A., 2009. Coccinellidae as predators of mites: Stethorini in biological control. Biological Control 51**,** 268-283.

Birkett, M.A., Pickett, J.A., 2003. Aphid sex pheromones: from discovery to commercial production. Phytochemistry 62**,** 651-656.

Blackmon, H., Demuth, J.P., 2015. Coleoptera Karyotype Database. The Coleopterists Bulletin 69**,** 174-175.

Bolger, A.M., Lohse, M., Usadel, B., 2014. Trimmomatic: a flexible trimmer for Illumina sequence data. Bioinformatics 30**,** 2114-2120.

Boutet, E., Lieberherr, D., Tognolli, M., Schneider, M., Bansal, P., Bridge, A.J., Poux, S., Bougueleret, L., Xenarios, I., 2016. UniProtKB/Swiss-Prot, the Manually Annotated Section of the UniProt KnowledgeBase: How to Use the Entry View. Methods in Molecular Biology 1374**,** 23-54.

Boyes, D., Crowley, L.M., University of Oxford and Wytham Woods Genome Acquisition Lab, Darwin Tree of Life Barcoding collective, Wellcome Sanger Institute Tree of Life programme, Wellcome Sanger Institute Scientific Operations: DNA Pipelines collective, Tree of Life Core Informatics collective, Darwin Tree of Life Consortium, 2021. The genome sequence of the harlequin ladybird, *Harmonia axyridis* (Pallas, 1773) [version 1; peer review: 1 approved with reservations]. Wellcome Open Research 6**,** 300.

Branstetter, M.G., Danforth, B.N., Pitts, J.P., Faircloth, B.C., Ward, P.S., Buffington, M.L., Gates, M.W., Kula, R.R., Brady, S.G., 2017. Phylogenomic Insights into the Evolution of Stinging Wasps and the Origins of Ants and Bees. Current Biology 27**,** 1019-1025.

Bray, N.L., Pimentel, H., Melsted, P., Pachter, L., 2016. Near-optimal probabilistic RNA-seq quantification. Nature Biotechnology 34**,** 525-527.

Brito, N.F., Moreira, M.F., Melo, A.C.A., 2016. A look inside odorant-binding proteins in insect chemoreception. Journal of Insect Physiology 95**,** 51-65.

Brown, K.S., 1975. The chemistry of aphids and scale insects. Chemical Society Reviews 4**,** 263-288.

Brownlie, J.C., Johnson, K.N., 2009. Symbiont-mediated protection in insect hosts. Trends in Microbiology 17**,** 348-354.

Bruna, T., Hoff, K.J., Lomsadze, A., Stanke, M., Borodovsky, M., 2021. BRAKER2: automatic eukaryotic genome annotation with GeneMark-EP+ and AUGUSTUS supported by a protein database. Nar Genomics and Bioinformatics 3**,** lqaa108.

Buchfink, B., Xie, C., Huson, D.H., 2015. Fast and sensitive protein alignment using DIAMOND. Nature Methods 12**,** 59-60.

Burton, J.N., Adey, A., Patwardhan, R.P., Qiu, R.L., Kitzman, J.O., Shendure, J., 2013. Chromosome-scale scaffolding of *de novo* genome assemblies based on chromatin interactions. Nature Biotechnology 31**,** 1119-1125.

Byeon, G.M., Lee, K.S., Gui, Z.Z., Kim, I., Kang, P.D., Lee, S.M., Sohn, H.D., Jin, B.R., 2005. A digestive β-glucosidase from the silkworm, *Bombyx mori*: cDNA cloning, expression and enzymatic characterization. Comparative Biochemistry and Physiology B: Biochemistry and Molecular Biology 141**,** 418-427.

Cai, L.S., Koziel, J.A., O'Neal, M.E., 2007. Determination of characteristic odorants from *Harmonia axyridis* beetles using *in vivo* solid-phase microextraction and multidimensional gas chromatography–mass spectrometry–olfactometry. Journal of Chromatography A 1147**,** 66-78.

Cairo, J.P.L.F., Oliveira, L.C., Uchima, C.A., Alvarez, T.M., Citadini, A.P.D., Cota, J., Leonardo, F.C., Costa-Leonardo, A.M., Carazzolle, M.F., Costa, F.F., Pereira, G.A.G., Squina, F.M., 2013. Deciphering the synergism of endogenous glycoside hydrolase families 1 and 9 from *Coptotermes gestroi*. Insect Biochemistry and Molecular Biology 43**,** 970-981.

Camacho, C., Coulouris, G., Avagyan, V., Ma, N., Papadopoulos, J., Bealer, K., Madden, T.L., 2009. BLAST+: architecture and applications. BMC Bioinformatics 10**,** 421.

Cantalapiedra, C.P., Hernandez-Plaza, A., Letunic, I., Bork, P., Huerta-Cepas, J., 2021. eggNOG-mapper v2: Functional Annotation, Orthology Assignments, and Domain Prediction at the Metagenomic Scale. Molecular Biology and Evolution 38**,** 5825-5829.

Capella-Gutierrez, S., Silla-Martinez, J.M., Gabaldon, T., 2009. trimAl: a tool for automated alignment trimming in large-scale phylogenetic analyses. Bioinformatics 25**,** 1972-1973.

Carbon, S., Douglass, E., Good, B.M., Unni, D.R., Harris, N.L., Mungall, C.J., Basu, S., Chisholm, R.L., Dodson, R.J., Hartline, E., Fey, P., Thomas, P.D., Albou, L.P., Ebert, D., Kesling, M.J., Mi, H.Y., Muruganujan, A., Huang, X.S., Mushayahama, T., LaBonte, S.A., Siegele, D.A., Antonazzo, G., Attrill, H., Brown, N.H., Garapati, P., Marygold, S.J., Trovisco, V., Dos Santos, G., Falls, K., Tabone, C., Zhou, P.L., Goodman, J.L., Strelets, V.B., Thurmond, J., Garmiri, P., Ishtiaq, R., Rodriguez-Lopez, M., Acencio, M.L., Kuiper, M., Laegreid, A., Logie, C., Lovering, R.C., Kramarz, B., Saverimuttu, S.C.C., Pinheiro, S.M., Gunn, H., Su, R.Z., Thurlow, K.E., Chibucos, M., Giglio, M., Nadendla, S., Munro, J., Jackson, R., Duesbury, M.J., Del-Toro, N., Meldal, B.H.M., Paneerselvam, K., Perfetto, L., Porras, P., Orchard, S., Shrivastava, A., Chang, H.Y., Finn, R.D., Mitchell, A.L., Rawlings, N.D., Richardson, L., Sangrador-Vegas, A., Blake, J.A., Christie, K.R., Dolan, M.E., Drabkin, H.J., Hill, D.P., Ni, L., Sitnikov, D.M., Harris, M.A., Oliver, S.G., Rutherford, K., Wood, V., Hayles, J., Bahler, J., Bolton, E.R., De Pons, J.L., Dwinell, M.R., Hayman, G.T., Kaldunski, M.L., Kwitek, A.E., Laulederkind, S.J.F., Plasterer, C., Tutaj, M.A., Vedi, M., Wang, S.J., D'Eustachio, P., Matthews, L., Balhoff, J.P., Aleksander, S.A., Alexander, M.J., Cherry, J.M., Engel, S.R., Gondwe, F., Karra, K., Miyasato, S.R., Nash, R.S., Simison, M., Skrzypek, M.S., Weng, S., Wong, E.D., Feuermann, M., Gaudet, P., Morgat, A., Bakker, E., Berardini, T.Z., Reiser, L., Subramaniam, S., Huala, E., Arighi, C.N., Auchincloss, A., Axelsen, K., Argoud-Puy, G., Bateman, A., Blatter, M.C., Boutet, E., Bowler, E., Breuza, L., Bridge, A., Britto, R., Bye-A-Jee, H., Casas, C.C., Coudert, E., Denny, P., Estreicher, A., Famiglietti, M.L., Georghiou, G., Gos, A., Gruaz-Gumowski, N., Hatton-Ellis, E., Hulo, C., Ignatchenko, A., Jungo, F., Laiho, K., Le Mercier, P., Lieberherr, D., Lock, A., Lussi, Y., MacDougall, A., Magrane, M., Martin, M.J., Masson, P., Natale, D.A., Hyka-Nouspikel, N., Orchard, S., Pedruzzi, I., Pourcel, L., Poux, S., Pundir, S., Rivoire, C., Speretta, E., Sundaram, S., Tyagi, N., Warner, K., Zaru, R., Wu, C.H., Diehl, A.D., Chan, J.N., Grove, C., Lee, R.Y.N., Muller, H.M., Raciti, D., Van Auken, K., Sternberg, P.W., Berriman, M., Paulini, M., Howe, K., Gao, S., Wright, A., Stein, L., Howe, D.G., Toro, S., Westerfield, M., Jaiswal, P., Cooper, L., Elser, J., Gene Ontology Consortium, 2021. The Gene Ontology resource: enriching a GOld mine. Nucleic Acids Research 49**,** D325-D334.

Carolan, J.C., Caragea, D., Reardon, K.T., Mutti, N.S., Dittmer, N., Pappan, K., Cui, F., Castaneto, M., Poulain, J., Dossat, C., Tagu, D., Reese, J.C., Reeck, G.R., Wilkinson, T.L., Edwards, O.R., 2011. Predicted Effector Molecules in the Salivary Secretome of the Pea Aphid (*Acyrthosiphon pisum*): A Dual Transcriptomic/Proteomic Approach. Journal of Proteome Research 10**,** 1505-1518.

Cattaneo, F., Pasini, M.E., Intra, J., Matsumoto, M., Briani, F., Hoshi, M., Perotti, M.E., 2006. Identification and expression analysis of *Drosophila melanogaster* genes encoding β-hexosaminidases of the sperm plasma membrane. Glycobiology 16**,** 786-800.

Chang, W.H., Lai, A.G., 2018. Mixed evolutionary origins of endogenous biomass-depolymerizing enzymes in animals. Bmc Genomics 19**,** 483.

Che, L.H., Zhang, P., Deng, S.H., Escalona, H.E., Wang, X.M., Li, Y., Pang, H., Vandenberg, N., Ślipiński, A., Tomaszewska, W., Liang, D., 2021. New insights into the phylogeny and evolution of lady beetles (Coleoptera: Coccinellidae) by extensive sampling of genes and species. Molecular Phylogenetics and Evolution 156**,** 107045.

Chen, C.-H., 2012. Activation and Detoxification Enzymes. Springer Science+Business Media, LLC, New York.

Chen, M.-L., Huang, Y.-H., Qiu, B.-Y., Chen, P.-T., Du, X.-Y., Li, H.-S., Pang, H., 2020. Changes in life history traits and transcriptional regulation of Coccinellini ladybirds in using alternative prey. BMC Genomics 21**,** 44.

Chen, M.Y., Mei, Y., Chen, X., Chen, X., Xiao, D., He, K., Li, Q., Wu, M.M., Wang, S., Zhang, F., Li, F., 2021. A chromosome-level assembly of the harlequin ladybird *Harmonia axyridis* as a genomic resource to study beetle and invasion biology. Molecular Ecology Resources 21**,** 1318-1332.

Cheng, Y., Zhi, J.R., Li, F.L., Wang, H., Zhou, Y.H., Jin, J.X., 2020. Transcriptome sequencing of *Coccinella septempunctata* adults (Coleoptera: Coccinellidae) feeding on artificial diet and *Aphis craccivora*. Plos One 15**,** e0236249.

Crowley, L.M., University of Oxford and Wytham Woods Genome Acquisition Lab, Darwin Tree of Life Barcoding collective, Wellcome Sanger Institute Tree of Life programme, Wellcome Sanger Institute Scientific Operations: DNA Pipelines collective, Tree of Life Core Informatics collective, Darwin Tree of Life Consortium, 2021a. The genome sequence of the common malachite beetle, *Malachius bipustulatus* (Linnaeus, 1758) [version 1; peer review: 2 approved, 1 approved with reservations]. Wellcome Open Research 6**,** 322.

Crowley, L.M., University of Oxford and Wytham Woods Genome Acquisition Lab, Darwin Tree of Life Barcoding collective, Wellcome Sanger Institute Tree of Life programme, Wellcome Sanger Institute Scientific Operations: DNA Pipelines collective, Tree of Life Core Informatics collective, Darwin Tree of Life Consortium, 2021b. The genome sequence of the seven-spotted ladybird, *Coccinella septempunctata* Linnaeus, 1758 [version 1; peer review: 2 approved]. Wellcome Open Research 6**,** 319.

Cunningham, C.B., Ji, L.X., Wiberg, R.A.W., Shelton, J., McKinney, E.C., Parker, D.J., Meagher, R.B., Benowitz, K.M., Roy-Zokan, E.M., Ritchie, M.G., Brown, S.J., Schmitz, R.J., Moore, A.J., 2015. The Genome and Methylome of a Beetle with Complex Social Behavior, *Nicrophorus vespilloides* (Coleoptera: Silphidae). Genome Biology and Evolution 7**,** 3383-3396.

De Coster, W., D'Hert, S., Schultz, D.T., Cruts, M., Van Broeckhoven, C., 2018. NanoPack: visualizing and processing long-read sequencing data. Bioinformatics 34**,** 2666-2669.

Denecke, S., Rankic, I., Driva, O., Kalsi, M., Luong, N.B.H., Buer, B., Nauen, R., Geibel, S., Vontas, J., 2021. Comparative and functional genomics of the ABC transporter superfamily across arthropods. Bmc Genomics 22**,** 553.

Dermauw, W., Van Leeuwen, T., 2014. The ABC gene family in arthropods: Comparative genomics and role in insecticide transport and resistance. Insect Biochemistry and Molecular Biology 45**,** 89-110.

Drula, E., Garron, M.-L., Dogan, S., Lombard, V., Henrissat, B., Terrapon, N., 2022. The carbohydrate-active enzyme database: functions and literature. Nucleic Acids Research 50**,** D571-D577.

Du, X.-Y., Yang, H.-Y., Gong, S.-R., Zhang, P.-F., Chen, P.-T., Liang, Y.-S., Huang, Y.-H., Tang, X.-F., Chen, Q.-K., De Clercq, P., Li, H.-S., Pang, H., 2022. Aphidophagous ladybird beetles adapt to an aphid symbiont. Functional Ecology 36**,** 2593-2604.

Du, Z.Y., Su, H., Wang, W.K., Ye, L.S., Wei, H., Peng, Z.L., Anishchenko, I., Baker, D., Yang, J.Y., 2021. The trRosetta server for fast and accurate protein structure prediction. Nature Protocols 16**,** 5634-5651.

El-Gebali, S., Mistry, J., Bateman, A., Eddy, S.R., Luciani, A., Potter, S.C., Qureshi, M., Richardson, L.J., Salazar, G.A., Smart, A., Sonnhammer, E.L.L., Hirsh, L., Paladin, L., Piovesan, D., Tosatto, S.C.E., Finn, R.D., 2019. The Pfam protein families database in 2019. Nucleic Acids Research 47**,** D427-D432.

Emms, D.M., Kelly, S., 2019. OrthoFinder: phylogenetic orthology inference for comparative genomics. Genome Biology 20**,** 238.

Escalona, H.E., Ślipiński, A., 2012. Generic revision and phylogeny of Microweiseinae (Coleoptera: Coccinellidae). Systematic Entomology 37**,** 125-171.

Escalona, H.E., Zwick, A., Li, H.-S., Li, J.H., Wang, X.M., Pang, H., Hartley, D., Jermiin, L.S., Nedvěd, O., Misof, B., Niehuis, O., Ślipiński, A., Tomaszewska, W., 2017. Molecular phylogeny reveals food plasticity in the evolution of true ladybird beetles (Coleoptera: Coccinellidae: Coccinellini). Bmc Evolutionary Biology 17**,** 151.

Evans, J.D., McKenna, D., Scully, E., Cook, S.C., Dainat, B., Egekwu, N., Grubbs, N., Lopez, D., Lorenzen, M.D., Reyna, S.M., Rinkevich, F.D., Neumann, P., Huang, Q., 2018. Genome of the small hive beetle (*Aethina tumida*, Coleoptera: Nitidulidae), a worldwide parasite of social bee colonies, provides insights into detoxification and herbivory. Gigascience 7**,** giy138.

Fallon, T.R., Lower, S.E., Chang, C.-H., Bessho-Uehara, M., Martin, G.J., Bewick, A.J., Behringer, M., Debat, H.J., Wong, I., Day, J.C., Suvorov, A., Silva, C.J., Stanger-Hall, K.F., Hall, D.W., Schmitz, R.J., Nelson, D.R., Lewis, S.M., Shigenobu, S., Bybee, S.M., Larracuente, A.M., Oba, Y., Weng, J.-K., 2018. Firefly genomes illuminate parallel origins of bioluminescence in beetles. Elife 7**,** e36495.

Ferreira, A.H.P., Marana, S.R., Terra, W.R., Ferreira, C., 2001. Purification, molecular cloning, and properties of a β-glycosidase isolated from midgut lumen of *Tenebrio molitor* (Coleoptera) larvae. Insect Biochemistry and Molecular Biology 31**,** 1065-1076.

Feyereisen, R., 2006. Evolution of insect P450. Biochemical Society Transactions 34**,** 1252-1255.

Foster, C.S.P., Sauquet, H., Van der Merwe, M., McPherson, H., Rossetto, M., Ho, S.Y.W., 2017. Evaluating the Impact of Genomic Data and Priors on Bayesian Estimates of the Angiosperm Evolutionary Timescale. Systematic Biology 66**,** 338-351.

Francis, F., Lognay, G., Wathelet, J.-P., Haubruge, E., 2001. Effects of allelochemicals from first (Brassicaceae) and second (*Myzus persicae* and *Brevicoryne brassicae*) trophic levels on *Adalia bipunctata*. Journal of Chemical Ecology 27**,** 243-256.

Gao, Q., Li, B., Tian, Z., De Loof, A., Wang, J.-L., Wang, X.-P., Liu, W., 2022. Key role of juvenile hormone in controlling reproductive diapause in females of the Asian lady beetle *Harmonia axyridis*. Pest Management Science 78**,** 193-204.

Gautier, M., Yamaguchi, J., Foucaud, J., Loiseau, A., Ausset, A., Facon, B., Gschloessl, B., Lagnel, J., Loire, E., Parrinello, H., Severac, D., Lopez-Roques, C., Donnadieu, C., Manno, M., Berges, H., Gharbi, K., Lawson-Handley, L., Zang, L.-S., Vogel, H., Estoup, A., Prud'homme, B., 2018. The Genomic Basis of Color Pattern Polymorphism in the Harlequin Ladybird. Current Biology 28**,** 3296-3302.

Genta, F.A., Blanes, L., Cristofoletti, P.T., do Lago, C.L., Terra, W.R., Ferreira, C., 2006. Purification, characterization and molecular cloning of the major chitinase from *Tenebrio molitor* larval midgut. Insect Biochemistry and Molecular Biology 36**,** 789-800.

Gholamzadeh-Chitgar, M., Ghadamyari, M., Ghanbarinezhad, R., 2017. Biochemical properties of digestive proteases of melon ladybird, *Epilachna chrysomelina* (Fabricius) (Col.: Coccinellidae). Journal of Entomological Society of Iran 37**,** 293-304.

Gilbert, D., 2013. Gene-omes built from mRNA seq not genome DNA. 7th annual arthropod genomics symposium, Notre Dame.

Giorgi, J.A., Vandenberg, N.J., McHugh, J.V., Forrester, J.A., Ślipiński, S.A., Miller, K.B., Shapiro, L.R., Whiting, M.F., 2009. The evolution of food preferences in Coccinellidae. Biological Control 51**,** 215-231.

Grabherr, M.G., Haas, B.J., Yassour, M., Levin, J.Z., Thompson, D.A., Amit, I., Adiconis, X., Fan, L., Raychowdhury, R., Zeng, Q.D., Chen, Z.H., Mauceli, E., Hacohen, N., Gnirke, A., Rhind, N., di Palma, F., Birren, B.W., Nusbaum, C., Lindblad-Toh, K., Friedman, N., Regev, A., 2011. Full-length transcriptome assembly from RNA-Seq data without a reference genome. Nature Biotechnology 29**,** 644-U130.

Haas, B.J., Delcher, A.L., Mount, S.M., Wortman, J.R., Smith, R.K., Hannick, L.I., Maiti, R., Ronning, C.M., Rusch, D.B., Town, C.D., Salzberg, S.L., White, O., 2003. Improving the *Arabidopsis* genome annotation using maximal transcript alignment assemblies. Nucleic Acids Research 31**,** 5654-5666.

Haas, B.J., Salzberg, S.L., Zhu, W., Pertea, M., Allen, J.E., Orvis, J., White, O., Buell, C.R., Wortman, J.R., 2008. Automated eukaryotic gene structure annotation using EVidenceModeler and the program to assemble spliced alignments. Genome Biology 9**,** R7.

Han, S.-P., Liang, C., Li, X.-B., Han, H., Zhao, F., He, Y.-Z., 2019. Cloning and expression profiling of the odorant binding protein genes *HaxyOBP*1 and *HaxyOBP*6 in *Harmonia axyridis* (Coleoptera: Coccinellidae). Acta Entomologica Sinica 62**,** 284-293.

He, S.L., Jiang, B., Chakraborty, A., Yu, G.Z., 2022. The Evolution of Glycoside Hydrolase Family 1 in Insects Related to Their Adaptation to Plant Utilization. Insects 13**,** 786.

Heidel-Fischer, H.M., Vogel, H., 2015. Molecular mechanisms of insect adaptation to plant secondary compounds. Current Opinion in Insect Science 8**,** 8-14.

Herndon, N., Shelton, J.M., Gerischer, L., Ioannidis, P., Ninova, M., Dönitz, J., Waterhouse, R.M., Liang, C.Y., Damm, C., Siemanowski, J., Kitzmann, P., Ulrich, J., Dippel, S., Oberhofer, G., Hu, Y.G., Schwirz, J., Schacht, M.I., Lehmann, S., Montino, A., Posnien, N., Gurska, D., Horn, T., Seibert, J., Jentzsch, I.M.V., Panfilio, K.A., Li, J., Wimmer, E.A., Stappert, D., Roth, S., Schröder, R., Park, Y., Schoppmeier, M., Chung, H.-R., Klingler, M., Kittelmann, S., Friedrich, M., Chen, R., Altincicek, B., Vilcinskas, A., Zdobnov, E.M., Griffiths-Jones, S., Ronshaugen, M., Stanke, M., Brown, S.J., Bucher, G., 2020. Enhanced genome assembly and a new official gene set for *Tribolium castaneum*. Bmc Genomics 21**,** 47.

Hodek, I., 1973. Biology of Coccinellidae. Springer Science & Business Media, Dordrecht.

Hodek, I., Honěk, A., 2009. Scale insects, mealybugs, whiteflies and psyllids (Hemiptera, Sternorrhyncha) as prey of ladybirds. Biological Control 51**,** 232-243.

Hodek, I., van Emden, H.F., Honěk, A., 2012. Ecology and Behaviour of the Ladybird Beetles (Coccinellidae). Wiley-Blackwell, West Sussex.

Hu, J., Fan, J.P., Sun, Z.Y., Liu, S.L., 2020. NextPolish: a fast and efficient genome polishing tool for long-read assembly. Bioinformatics 36**,** 2253-2255.

Hu, J., Wang, Z., Sun, Z.Y., Hu, B.X., Ayoola, A.O., Liang, F., Li, J.J., Sandoval, J.R., Cooper, D.N., Ye, K., Ruan, J., Xiao, C.-L., Wang, D.-P., Wu, D.-D., Wang, S., 2023. An efficient error correction and accurate assembly tool for noisy long reads. bioRxiv**,** 2023.2003.2009.531669.

Huang, Y.-H., Du, X.-Y., Chen, P.-T., Tang, X.-F., Gong, S.-R., Zhang, P.-F., Yang, H.-Y., De Clercq, P., Li, H.-S., Pang, H., 2022. Is pollinivory in the omnivorous ladybird beetle *Micraspis discolor* (Coleoptera: Coccinellidae) symbiosis-dependent? Biological Control 169**,** 104867.

Huerta-Cepas, J., Serra, F., Bork, P., 2016. ETE 3: Reconstruction, Analysis, and Visualization of Phylogenomic Data. Molecular Biology and Evolution 33**,** 1635-1638.

Huerta-Cepas, J., Szklarczyk, D., Heller, D., Hernandez-Plaza, A., Forslund, S.K., Cook, H., Mende, D.R., Letunic, I., Rattei, T., Jensen, L.J., von Mering, C., Bork, P., 2019. eggNOG 5.0: a hierarchical, functionally and phylogenetically annotated orthology resource based on 5090 organisms and 2502 viruses. Nucleic Acids Research 47**,** D309-D314.

Jasrapuria, S., Arakane, Y., Osman, G., Kramer, K.J., Beeman, R.W., Muthukrishnan, S., 2010. Genes encoding proteins with peritrophin A-type chitin-binding domains in *Tribolium castaneum* are grouped into three distinct families based on phylogeny, expression and function. Insect Biochemistry and Molecular Biology 40**,** 214-227.

Johnson, K.P., Dietrich, C.H., Friedrich, F., Beutel, R.G., Wipfler, B., Peters, R.S., Allen, J.M., Petersen, M., Donath, A., Walden, K.K.O., Kozlov, A.M., Podsiadlowski, L., Mayer, C., Meusemann, K., Vasilikopoulos, A., Waterhouse, R.M., Cameron, S.L., Weirauch, C., Swanson, D.R., Percy, D.M., Hardy, N.B., Terry, I., Liu, S.L., Zhou, X., Misof, B., Robertson, H.M., Yoshizawa, K., 2018. Phylogenomics and the evolution of hemipteroid insects. Proceedings of the National Academy of Sciences of the United States of America 115**,** 12775-12780.

Jones, A.M.E., Winge, P., Bones, A.M., Cole, R., Rossiter, J.T., 2002. Characterization and evolution of a myrosinase from the cabbage aphid *Brevicoryne brassicae*. Insect Biochemistry and Molecular Biology 32**,** 275-284.

Jones, P., Binns, D., Chang, H.Y., Fraser, M., Li, W.Z., McAnulla, C., McWilliam, H., Maslen, J., Mitchell, A., Nuka, G., Pesseat, S., Quinn, A.F., Sangrador-Vegas, A., Scheremetjew, M., Yong, S.Y., Lopez, R., Hunter, S., 2014. InterProScan 5: genome-scale protein function classification. Bioinformatics 30**,** 1236-1240.

Kall, L., Krogh, A., Sonnhammer, E.L.L., 2004. A combined transmembrane topology and signal peptide prediction method. Journal of Molecular Biology 338**,** 1027-1036.

Kanehisa, M., Furumichi, M., Sato, Y., Ishiguro-Watanabe, M., Tanabe, M., 2021. KEGG: integrating viruses and cellular organisms. Nucleic Acids Research 49**,** D545-D551.

Katoh, K., Standley, D.M., 2013. MAFFT Multiple Sequence Alignment Software Version 7: Improvements in Performance and Usability. Molecular Biology and Evolution 30**,** 772-780.

Keeling, C.I., Campbell, E.O., Batista, P.D., Shegelski, V.A., Trevoy, S.A.L., Huber, D.P.W., Janes, J.K., Sperling, F.A.H., 2022. Chromosome-level genome assembly reveals genomic architecture of northern range expansion in the mountain pine beetle, *Dendroctonus ponderosae* Hopkins (Coleoptera: Curculionidae). Molecular Ecology Resources 22**,** 1149-1167.

Keeling, C.I., Yuen, M.M.S., Liao, N.Y., Docking, T.R., Chan, S.K., Taylor, G.A., Palmquist, D.L., Jackman, S.D., Nguyen, A., Li, M., Henderson, H., Janes, J.K., Zhao, Y.J., Pandoh, P., Moore, R., Sperling, F.A.H., Huber, D.P.W., Birol, I., Jones, S.J.M., Bohlmann, J., 2013. Draft genome of the mountain pine beetle, *Dendroctonus ponderosae* Hopkins, a major forest pest. Genome Biology 14**,** R27.

Kent, W.J., 2002. BLAT - The BLAST-like alignment tool. Genome Research 12**,** 656-664.

Kim, D., Paggi, J.M., Park, C., Bennett, C., Salzberg, S.L., 2019. Graph-based genome alignment and genotyping with HISAT2 and HISAT-genotype. Nature Biotechnology 37**,** 907-915.

Kim, S., Chen, J., Cheng, T.J., Gindulyte, A., He, J., He, S.Q., Li, Q.L., Shoemaker, B.A., Thiessen, P.A., Yu, B., Zaslavsky, L., Zhang, J., Bolton, E.E., 2023. PubChem 2023 update. Nucleic Acids Research 51**,** D1373-D1380.

King, R., Buer, B., Davies, T.G.E., Ganko, E., Guest, M., Hassani-Pak, K., Hughes, D., Raming, K., Rawlings, C., Williamson, M., Crossthwaite, A., Nauen, R., Field, L., 2023. The complete genome assemblies of 19 insect pests of worldwide importance to agriculture. Pesticide Biochemistry and Physiology 191**,** 105339.

Kirejtshuk, A.G., Azar, D., 2009. New beetles of Polyphaga (Coleoptera, Polyphaga) from Lower Cretaceous Lebanese amber. Denisia 26**,** 119-130.

Kirejtshuk, A.G., Azar, D., Beaver, R.A., Mandelshtam, M.Y., Nel, A., 2009. The most ancient bark beetle known: a new tribe, genus and species from Lebanese amber (Coleoptera, Curculionidae, Scolytinae). Systematic Entomology 34**,** 101-112.

Kirejtshuk, A.G., Nel, A., 2012. The oldest representatives of the family Coccinellidae (Coleoptera: Polyphaga) from the lowermost Eocene Oise amber (France). Zoosystematica Rossica 21**,** 131-144.

Kirejtshuk, A.G., Poschmann, M., Prokop, J., Garrouste, R., Nel, A., 2014. Evolution of the elytral venation and structural adaptations in the oldest Palaeozoic beetles (Insecta: Coleoptera: Tshekardocoleidae). Journal of Systematic Palaeontology 12**,** 575-600.

Koo, M.S., Park, Y.C., 2002. Gut Luminal Digestive Proteinases of Adult Lady Beetle, *Harmonia axyridis* (Coccinellidae: Coleoptera), Fed an Artificial Diet. Journal of Asia-Pacific Entomology 5**,** 167-173.

Koren, S., Walenz, B.P., Berlin, K., Miller, J.R., Bergman, N.H., Phillippy, A.M., 2017. Canu: scalable and accurate long-read assembly via adaptive *k*-mer weighting and repeat separation. Genome Research 27**,** 722-736.

Korf, I., 2004. Gene finding in novel genomes. BMC Bioinformatics 5**,** 59.

Kovaka, S., Zimin, A.V., Pertea, G.M., Razaghi, R., Salzberg, S.L., Pertea, M., 2019. Transcriptome assembly from long-read RNA-seq alignments with StringTie2. Genome Biology 20**,** 278.

Kriventseva, E.V., Kuznetsov, D., Tegenfeldt, F., Manni, M., Dias, R., Simao, F.A., Zdobnov, E.M., 2019. OrthoDB v10: sampling the diversity of animal, plant, fungal, protist, bacterial and viral genomes for evolutionary and functional annotations of orthologs. Nucleic Acids Research 47**,** D807-D811.

Krzywinski, M., Schein, J., Birol, I., Connors, J., Gascoyne, R., Horsman, D., Jones, S.J., Marra, M.A., 2009. Circos: An information aesthetic for comparative genomics. Genome Research 19**,** 1639-1645.

Laetsch, D.R., Blaxter, M.L., 2017a. BlobTools: Interrogation of genome assemblies [version 1; peer review: 2 approved with reservations]. F1000Research 6**,** 1287.

Laetsch, D.R., Blaxter, M.L., 2017b. KinFin: Software for Taxon-Aware Analysis of Clustered Protein Sequences. G3 Genes|Genomes|Genetics 7**,** 3349-3357.

Langmead, B., Salzberg, S.L., 2012. Fast gapped-read alignment with Bowtie 2. Nature Methods 9**,** 357-U354.

Leal, W.S., 2013. Odorant Reception in Insects: Roles of Receptors, Binding Proteins, and Degrading Enzymes. Annual Review of Entomology 58**,** 373-391.

Leschen, R.A.B., 2000. Beetles feeding on bugs (Coleoptera, Hemiptera): repeated shifts from mycophagous ancestors. Invertebrate Taxonomy 14**,** 917-929.

Li, B., Dewey, C.N., 2011. RSEM: accurate transcript quantification from RNA-Seq data with or without a reference genome. BMC Bioinformatics 12**,** 323.

Li, G.N., Chen, P.-T., Chen, M.-L., Chen, T.-Y., Huang, Y.-H., Lü, X., Li, H.-S., Pang, H., 2024. Effect of *Ephestia kuehniella* Eggs on Development and Transcriptome of the Ladybird Beetle *Propylea japonica*. Insects 15**,** 407.

Li, H.-S., Huang, Y.-H., Chen, M.-L., Ren, Z., Qiu, B.-Y., De Clercq, P., Heckel, G., Pang, H., 2021a. Genomic insight into diet adaptation in the biological control agent *Cryptolaemus montrouzieri*. Bmc Genomics 22**,** 135.

Li, H.-S., Pan, C., De Clercq, P., Ślipiński, A., Pang, H., 2016a. Variation in life history traits and transcriptome associated with adaptation to diet shifts in the ladybird *Cryptolaemus montrouzieri*. Bmc Genomics 17**,** 281.

Li, H.-S., Tang, X.-F., Huang, Y.-H., Xu, Z.-Y., Chen, M.-L., Du, X.-Y., Qiu, B.-Y., Chen, P.-T., Zhang, W., Ślipiński, A., Escalona, H.E., Waterhouse, R.M., Zwick, A., Pang, H., 2021b. Horizontally acquired antibacterial genes associated with adaptive radiation of ladybird beetles. Bmc Biology 19**,** 7.

Li, H., 2018. Minimap2: pairwise alignment for nucleotide sequences. Bioinformatics 34**,** 3094-3100.

Li, H., Durbin, R., 2009. Fast and accurate short read alignment with Burrows-Wheeler transform. Bioinformatics 25**,** 1754-1760.

Li, W., Zhang, Y.-F., Xie, Y.-P., Niu, X.-P., 2016b. Selection response of *Harmonia axyridis* (Pallas) to body volatile of *Ceroplastes japonicus* Green. Journal of Environmental Entomology 38**,** 329-336.

Li, W.J., Laczyński, P., Escalona, H.E., Eberle, J., Huo, L.Z., Chen, X.S., Huang, W.D., Chen, B.X., Ahrens, D., Ślipiński, A., Tomaszewska, W., Wang, X.M., 2020. Combined molecular and morphological data provide insights into the evolution and classification of Chilocorini ladybirds (Coleoptera: Coccinellidae). Systematic Entomology 45**,** 447-463.

Li, W.J., O'Neill, K.R., Haft, D.H., DiCuccio, M., Chetvernin, V., Badretdin, A., Coulouris, G., Chitsaz, F., Derbyshire, M.K., Durkin, A.S., Gonzales, N.R., Gwadz, M., Lanczycki, C.J., Song, J.S., Thanki, N., Wang, J.Y., Yamashita, R.A., Yang, M.Z., Zheng, C.J., Marchler-Bauer, A., Thibaud-Nissen, F., 2021c. RefSeq: expanding the Prokaryotic Genome Annotation Pipeline reach with protein family model curation. Nucleic Acids Research 49**,** D1020-D1028.

Li, X.C., Schuler, M.A., Berenbaum, M.R., 2007. Molecular mechanisms of metabolic resistance to synthetic and natural xenobiotics. Annual Review of Entomology 52**,** 231-253.

Liang, C., Han, S.P., Han, H., Zhao, F., He, Y.Z., 2019. Selection of reference genes for *Harmonia axyridis* (Coleoptera: Coccinellidae) feeding on different diets. Journal of Asia-Pacific Entomology 22**,** 1115-1122.

Lomsadze, A., Burns, P.D., Borodovsky, M., 2014. Integration of mapped RNA-Seq reads into automatic training of eukaryotic gene finding algorithm. Nucleic Acids Research 42**,** e119.

Love, M.I., Huber, W., Anders, S., 2014. Moderated estimation of fold change and dispersion for RNA-seq data with DESeq2. Genome Biology 15**,** 550.

Lundgren, J.G., 2009. Relationships of Natural Enemies and Non-Prey Foods. Springer International, Dordrecht.

Magro, A., Lecompte, E., Magné, F., Hemptinne, J.-L., Crouau-Roy, B., 2010. Phylogeny of ladybirds (Coleoptera: Coccinellidae): Are the subfamilies monophyletic? Molecular Phylogenetics and Evolution 54**,** 833-848.

Majoros, W.H., Pertea, M., Salzberg, S.L., 2004. TigrScan and GlimmerHMM: two open source *ab initio* eukaryotic gene-finders. Bioinformatics 20**,** 2878-2879.

Manni, M., Berkeley, M.R., Seppey, M., Simao, F.A., Zdobnov, E.M., 2021. BUSCO Update: Novel and Streamlined Workflows along with Broader and Deeper Phylogenetic Coverage for Scoring of Eukaryotic, Prokaryotic, and Viral Genomes. Molecular Biology and Evolution 38**,** 4647-4654.

Marks, P., Garcia, S., Barrio, A.M., Belhocine, K., Bernate, J., Bharadwaj, R., Bjornson, K., Catalanotti, C., Delaney, J., Fehr, A., Fiddes, I.T., Galvin, B., Heaton, H., Herschleb, J., Hindson, C., Holt, E., Jabara, C.B., Jett, S., Keivanfar, N., Kyriazopoulou-Panagiotopoulou, S., Lek, M., Lin, B., Lowe, A., Mahamdallie, S., Maheshwari, S., Makarewicz, T., Marshall, J., Meschi, F., O'Keefe, C.J., Ordonez, H., Patel, P., Price, A., Royall, A., Ruark, E., Seal, S., Schnall-Levin, M., Shah, P., Stafford, D., Williams, S., Wu, I., Xu, A.W., Rahman, N., MacArthur, D., Church, D.M., 2019. Resolving the full spectrum of human genome variation using Linked-Reads. Genome Research 29**,** 635-645.

McKenna, D.D., Scully, E.D., Pauchet, Y., Hoover, K., Kirsch, R., Geib, S.M., Mitchell, R.F., Waterhouse, R.M., Ahn, S.-J., Arsala, D., Benoit, J.B., Blackmon, H., Bledsoe, T., Bowsher, J.H., Busch, A., Calla, B., Chao, H., Childers, A.K., Childers, C., Clarke, D.J., Cohen, L., Demuth, J.P., Dinh, H., Doddapaneni, H., Dolan, A., Duan, J.J., Dugan, S., Friedrich, M., Glastad, K.M., Goodisman, M.A.D., Haddad, S., Han, Y., Hughes, D.S.T., Ioannidis, P., Johnston, J.S., Jones, J.W., Kuhn, L.A., Lance, D.R., Lee, C.-Y., Lee, S.L., Lin, H., Lynch, J.A., Moczek, A.P., Murali, S.C., Muzny, D.M., Nelson, D.R., Palli, S.R., Panfilio, K.A., Pers, D., Poelchau, M.F., Quan, H.H., Qu, J.X., Ray, A.M., Rinehart, J.P., Robertson, H.M., Roehrdanz, R., Rosendale, A.J., Shin, S., Silva, C., Torson, A.S., Jentzsch, I.M.V., Werren, J.H., Worley, K.C., Yocum, G., Zdobnov, E.M., Gibbs, R.A., Richards, S., 2016. Genome of the Asian longhorned beetle (*Anoplophora glabripennis*), a globally significant invasive species, reveals key functional and evolutionary innovations at the beetle-plant interface. Genome Biology 17**,** 227.

McKenna, D.D., Shin, S., Ahrens, D., Balke, M., Beza-Beza, C., Clarke, D.J., Donath, A., Escalona, H.E., Friedrich, F., Letsch, H., Liu, S.L., Maddison, D., Mayer, C., Misof, B., Murin, P.J., Niehuis, O., Peters, R.S., Podsiadlowski, L., Pohl, H., Scully, E.D., Yan, E.V., Zhou, X., Ślipiński, A., Beutel, R.G., 2019. The evolution and genomic basis of beetle diversity. Proceedings of the National Academy of Sciences of the United States of America 116**,** 24729-24737.

Mckenna, D.D., Wild, A.L., Kanda, K., Bellamy, C.L., Beutel, R.G., Caterino, M.S., Farnum, C.W., Hawks, D.C., Ivie, M.A., Jameson, M.L., Leschen, R.A.B., Marvaldi, A.E., McHugh, J.V., Newton, A.F., Robertson, J.A., Thayer, M.K., Whiting, M.F., Lawrence, J.F., Ślipiński, A., Maddison, D.R., Farrell, B.D., 2015. The beetle tree of life reveals that Coleoptera survived end-Permian mass extinction to diversify during the Cretaceous terrestrial revolution. Systematic Entomology 40**,** 835-880.

McLean, A.H.C., 2019. Cascading effects of defensive endosymbionts. Current Opinion in Insect Science 32**,** 42-46.

Meekins, D.A., Kanost, M.R., Michel, K., 2017. Serpins in arthropod biology. Seminars in Cell & Developmental Biology 62**,** 105-119.

Mei, Y., Jing, D., Tang, S.Y., Chen, X., Chen, H., Duanmu, H.N., Cong, Y.Y., Chen, M.Y., Ye, X.H., Zhou, H., He, K., Li, F., 2022. InsectBase 2.0: a comprehensive gene resource for insects. Nucleic Acids Research 50**,** D1040-D1045.

Mendes, F.K., Vanderpool, D., Fulton, B., Hahn, M.W., 2020. CAFE 5 models variation in evolutionary rates among gene families. Bioinformatics 36**,** 5516-5518.

Milligan, B.G., 1988. Total DNA isolation. In: Hoelzel, A.R., (Ed.), Molecular genetic analysis of populations. Oxford University Press, Oxford, pp. 29-64.

Mindnich, R.D., Penning, T.M., 2009. Aldo-keto reductase (AKR) superfamily: genomics and annotation. Human Genomics 3**,** 362-370.

Minh, B.Q., Schmidt, H.A., Chernomor, O., Schrempf, D., Woodhams, M.D., von Haeseler, A., Lanfear, R., 2020. IQ-TREE 2: New Models and Efficient Methods for Phylogenetic Inference in the Genomic Era. Molecular Biology and Evolution 37**,** 1530-1534.

Misof, B., Liu, S.L., Meusemann, K., Peters, R.S., Donath, A., Mayer, C., Frandsen, P.B., Ware, J., Flouri, T., Beutel, R.G., Niehuis, O., Petersen, M., Izquierdo-Carrasco, F., Wappler, T., Rust, J., Aberer, A.J., Aspock, U., Aspock, H., Bartel, D., Blanke, A., Berger, S., Bohm, A., Buckley, T.R., Calcott, B., Chen, J.Q., Friedrich, F., Fukui, M., Fujita, M., Greve, C., Grobe, P., Gu, S.C., Huang, Y., Jermiin, L.S., Kawahara, A.Y., Krogmann, L., Kubiak, M., Lanfear, R., Letsch, H., Li, Y.Y., Li, Z.Y., Li, J.G., Lu, H.R., Machida, R., Mashimo, Y., Kapli, P., McKenna, D.D., Meng, G.L., Nakagaki, Y., Navarrete-Heredia, J.L., Ott, M., Ou, Y.X., Pass, G., Podsiadlowski, L., Pohl, H., von Reumont, B.M., Schutte, K., Sekiya, K., Shimizu, S., Ślipiński, A., Stamatakis, A., Song, W.H., Su, X., Szucsich, N.U., Tan, M.H., Tan, X.M., Tang, M., Tang, J.B., Timelthaler, G., Tomizuka, S., Trautwein, M., Tong, X.L., Uchifune, T., Walzl, M.G., Wiegmann, B.M., Wilbrandt, J., Wipfler, B., Wong, T.K.F., Wu, Q., Wu, G.X., Xie, Y.L., Yang, S.Z., Yang, Q., Yeates, D.K., Yoshizawa, K., Zhang, Q., Zhang, R., Zhang, W.W., Zhang, Y.H., Zhao, J., Zhou, C.R., Zhou, L.L., Ziesmann, T., Zou, S.J., Li, Y.R., Xu, X., Zhang, Y., Yang, H.M., Wang, J., Wang, J., Kjer, K.M., Zhou, X., 2014. Phylogenomics resolves the timing and pattern of insect evolution. Science 346**,** 763-767.

Mistry, J., Chuguransky, S., Williams, L., Qureshi, M., Salazar, G.A., Sonnhammer, E.L.L., Tosatto, S.C.E., Paladin, L., Raj, S., Richardson, L.J., Finn, R.D., Bateman, A., 2021. Pfam: The protein families database in 2021. Nucleic Acids Research 49**,** D412-D419.

Mistry, J., Finn, R.D., Eddy, S.R., Bateman, A., Punta, M., 2013. Challenges in homology search: HMMER3 and convergent evolution of coiled-coil regions. Nucleic Acids Research 41**,** e121.

Mitchell, A.L., Attwood, T.K., Babbitt, P.C., Blum, M., Bork, P., Bridge, A., Brown, S.D., Chang, H.Y., El-Gebali, S., Fraser, M.I., Gough, J., Haft, D.R., Huang, H.Z., Letunic, I., Lopez, R., Luciani, A., Madeira, F., Marchler-Bauer, A., Mi, H.Y., Natale, D.A., Necci, M., Nuka, G., Orengo, C., Pandurangan, A.P., Paysan-Lafosse, T., Pesseat, S., Potter, S.C., Qureshi, M.A., Rawlings, N.D., Redaschi, N., Richardson, L.J., Rivoire, C., Salazar, G.A., Sangrador-Vegas, A., Sigrist, C.J.A., Sillitoe, I., Sutton, G.G., Thanki, N., Thomas, P.D., Tosatto, S.C.E., Yong, S.Y., Finn, R.D., 2019. InterPro in 2019: improving coverage, classification and access to protein sequence annotations. Nucleic Acids Research 47**,** D351-D360.

Mitterboeck, T.F., Liu, S.L., Adamowicz, S.J., Fu, J.Z., Zhang, R., Song, W.H., Meusemann, K., Zhou, X., 2017. Positive and relaxed selection associated with flight evolution and loss in insect transcriptomes. Gigascience 6**,** 1-14.

Montgomery, S.H., Capellini, I., Venditti, C., Barton, R.A., Mundy, N.I., 2011. Adaptive Evolution of Four Microcephaly Genes and the Evolution of Brain Size in Anthropoid Primates. Molecular Biology and Evolution 28**,** 625-638.

Musser, R.O., Hum-Musser, S.M., Eichenseer, H., Peiffer, M., Ervin, G., Murphy, J.B., Felton, G.W., 2002. Herbivory: Caterpillar saliva beats plant defences - A new weapon emerges in the evolutionary arms race between plants and herbivores. Nature 416**,** 599-600.

Nadeau, E.A.W., Lecheta, M.C., Obrycki, J.J., Teets, N.M., 2022. Transcriptional Regulation of Reproductive Diapause in the Convergent Lady Beetle, *Hippodamia convergens*. Insects 13**,** 343.

Nagamatsu, Y., Yanagisawa, I., Kimoto, M., Okamoto, E., Koga, D., 1995. Purification of a Chitooligosaccharidolytic *β-N-*Acetylglucosaminidase from *Bombyx mori* Larvae during Metamorphosis and the Nucleotide Sequence of Its cDNA. Bioscience, Biotechnology, and Biochemistry 59**,** 219-225.

Nattier, R., Michel-Salzat, A., Almeida, L.M., Chifflet-Belle, P., Magro, A., Salazar, K., Kergoat, G.J., 2021. Phylogeny and divergence dating of the ladybird beetle tribe Coccinellini Latreille (Coleoptera: Coccinellidae: Coccinellinae). Systematic Entomology 46**,** 632-648.

Nielsen, S.A., Hauge, M.S., Nielsen, F.H., Toft, S., 2000. Activities of glutathione S-transferase and glutathione peroxidases related to diet quality in an aphid predator, the seven-spot ladybird, *Coccinella septempunctata* L. (Coleoptera : Coccinellidae). Alternatives to Laboratory Animals 28**,** 445-449.

Nikolajev, G.V., Wang, B., Liu, Y., Zhang, H.C., 2011. Stag beetles from the Mesozoic of Inner Mongolia, China (Scarabaeoidea: Lucanidae). Acta Palaeontologica Sinica 50**,** 41-47.

Obiero, G.F., Pauli, T., Geuverink, E., Veenendaal, R., Niehuis, O., Große-Wilde, E., 2021. Chemoreceptor Diversity in Apoid Wasps and Its Reduction during the Evolution of the Pollen-Collecting Lifestyle of Bees (Hymenoptera: Apoidea). Genome Biology and Evolution 13**,** evaa269.

Oeyen, J.P., Baa-Puyoulet, P., Benoit, J.B., Beukeboom, L.W., Bornberg-Bauer, E., Buttstedt, A., Calevro, F., Cash, E.I., Chao, H., Charles, H., Chen, M.-J.M., Childers, C., Cridge, A.G., Dearden, P., Dinh, H., Doddapaneni, H.V., Dolan, A., Donath, A., Dowling, D., Dugan, S., Duncan, E., Elpidina, E.N., Friedrich, M., Geuverink, E., Gibson, J.D., Grath, S., Grimmelikhuijzen, C.J.P., Große-Wilde, E., Gudobba, C., Han, Y., Hansson, B.S., Hauser, F., Hughes, D.S.T., Ioannidis, P., Jacquin-Joly, E., Jennings, E.C., Jones, J.W., Klasberg, S., Lee, S.L., Lesný, P., Lovegrove, M., Martin, S., Martynov, A.G., Mayer, C., Montagné, N., Moris, V.C., Munoz-Torres, M., Murali, S.C., Muzny, D.M., Oppert, B., Parisot, N., Pauli, T., Peters, R.S., Petersen, M., Pick, C., Persyn, E., Podsiadlowski, L., Poelchau, M.F., Provataris, P., Qu, J.L., Reijnders, M.J.M.F., von Reumont, B.M., Rosendale, A.J., Simao, F.A., Skelly, J., Sotiropoulos, A.G., Stahl, A.L., Sumitani, M., Szuter, E.M., Tidswell, O., Tsitlakidis, E., Vedder, L., Waterhouse, R.M., Werren, J.H., Wilbrandt, J., Worley, K.C., Yamamoto, D.S., van de Zande, L., Zdobnov, E.M., Ziesmann, T., Gibbs, R.A., Richards, S., Hatakeyama, M., Misof, B., Niehuis, O., 2020. Sawfly Genomes Reveal Evolutionary Acquisitions That Fostered the Mega-Radiation of Parasitoid and Eusocial Hymenoptera. Genome Biology and Evolution 12**,** 1099-1118.

Parisot, N., Vargas-Chávez , C., Goubert, C., Baa-Puyoulet, P., Balmand, S., Beranger, L., Blanc, C., Bonnamour, A., Boulesteix, M., Burlet, N., Calevro, F., Callaerts, P., Chancy, T., Charles, H., Colella, S., Barbosa, A.D., Dell'Aglio, E., Di Genova, A., Febvay, G., Gabaldón, T., Ferrarini, M.G., Gerber, A., Gillet, B., Hubley, R., Hughes, S., Jacquin-Joly, E., Maire, J., Marcet-Houben, M., Masson, F., Meslin, C., Montagné, N., Moya, A., de Vasconcelos, A.T.R., Richard, G., Rosen, J., Sagot, M.-F., Smit, A.F.A., Storer, J.M., Vincent-Monegat, C., Vallier, A., Vigneron, A., Zaidman-Rémy, A., Zamoum, W., Vieira, C., Rebollo, R., Latorre, A., Heddi, A., 2021. The transposable element-rich genome of the cereal pest *Sitophilus oryzae*. Bmc Biology 19**,** 241.

Pauchet, Y., Wilkinson, P., Vogel, H., Nelson, D.R., Reynolds, S.E., Heckel, D.G., Ffrench-Constant, R.H., 2010. Pyrosequencing the *Manduca sexta* larval midgut transcriptome: messages for digestion, detoxification and defence. Insect Molecular Biology 19**,** 61-75.

Pelosi, P., Iovinella, I., Felicioli, A., Dani, F.R., 2014. Soluble proteins of chemical communication: an overview across arthropods. Frontiers in Physiology 5**,** 320.

Pelosi, P., Zhou, J.-J., Ban, L.P., Calvello, M., 2006. Soluble proteins in insect chemical communication. Cellular and Molecular Life Sciences 63**,** 1658-1676.

Peng, Y., Leung, H.C.M., Yiu, S.-M., Lv, M.-J., Zhu, X.-G., Chin, F.Y.L., 2013. IDBA-tran: a more robust de novo de Bruijn graph assembler for transcriptomes with uneven expression levels. Bioinformatics 29**,** 326-334.

Pentzold, S., Jensen, M.K., Matthes, A., Olsen, C.E., Petersen, B.L., Clausen, H., Møller, B.L., Bak, S., Zagrobelny, M., 2017. Spatial separation of the cyanogenic β-glucosidase ZfBGD2 and cyanogenic glucosides in the haemolymph of *Zygaena* larvae facilitates cyanide release. Royal Society Open Science 4**,** 170262.

Pervez, A., Yadav, M., 2018. Foraging Behaviour of Predaceous Ladybird Beetles: A Review. European Journal of Environmental Sciences 8**,** 102-108.

Piersanti, S., Saitta, V., Rebora, M., Salerno, G., 2022. Olfaction in phytophagous ladybird beetles: antennal sensilla and sensitivity to volatiles from host plants in *Chnootriba elaterii*. Arthropod-Plant Interactions 16**,** 617-630.

Qu, C., Wang, R., Che, W.-N., Li, F.-Q., Zhao, H.-P., Wei, Y.-Y., Luo, C., Xue, M., 2021. Identification and tissue distribution of odorant binding protein genes in *Harmonia axyridis* (Coleoptera: Coccinellidae). Journal of Integrative Agriculture 20**,** 2204-2213.

Qu, C., Yang, Z.-K., Wang, S., Zhao, H.-P., Li, F.-Q., Yang, X.-L., Luo, C., 2022a. Binding Affinity Characterization of Four Antennae-Enriched Odorant-Binding Proteins From *Harmonia axyridis* (Coleoptera: Coccinellidae). Frontiers in Physiology 13**,** 829766.

Qu, M.-B., Guo, X.-X., Kong, L., Hou, L.-J., Yang, Q., 2022b. A midgut-specific lytic polysaccharide monooxygenase of *Locusta migratoria* is indispensable for the deconstruction of the peritrophic matrix. Insect Science 29**,** 1287-1298.

Qu, M.B., Guo, X.X., Tian, S., Yang, Q., Kim, M., Mun, S., Noh, M.Y., Kramer, K.J., Muthukrishnan, S., Arakane, Y., 2022c. AA15 lytic polysaccharide monooxygenase is required for efficient chitinous cuticle turnover during insect molting. Communications Biology 5**,** 518.

Rambaut, A., Drummond, A.J., Xie, D., Baele, G., Suchard, M.A., 2018. Posterior Summarization in Bayesian Phylogenetics Using Tracer 1.7. Systematic Biology 67**,** 901-904.

Rao, S.S.P., Huntley, M.H., Durand, N.C., Stamenova, E.K., Bochkov, I.D., Robinson, J.T., Sanborn, A.L., Machol, I., Omer, A.D., Lander, E.S., Aiden, E.L., 2014. A 3D Map of the Human Genome at Kilobase Resolution Reveals Principles of Chromatin Looping. Cell 159**,** 1665-1680.

Rawlings, N.D., Barrett, A.J., Thomas, P.D., Huang, X.D., Bateman, A., Finn, R.D., 2018. The MEROPS database of proteolytic enzymes, their substrates and inhibitors in 2017 and a comparison with peptidases in the PANTHER database. Nucleic Acids Research 46**,** D624-D632.

Ren, S.X., Wang, X.M., Pang, H., Peng, Z.Q., Zeng, T., 2009. Colored Pictorial Handbook of Ladybird Beetles in China. Science Press, Beijing.

Revell, L.J., 2012. phytools: an R package for phylogenetic comparative biology (and other things). Methods in Ecology and Evolution 3**,** 217-223.

Richards, S., Gibbs, R.A., Weinstock, G.M., Brown, S.J., Denell, R., Beeman, R.W., Gibbs, R., Bucher, G., Friedrich, M., Grimmelikhuijzen, C.J.P., Klingler, M., Lorenzen, M.D., Roth, S., Schröder, R., Tautz, D., Zdobnov, E.M., Muzny, D., Attaway, T., Bell, S., Buhay, C.J., Chandrabose, M.N., Chavez, D., Clerk-Blankenburg, K.P., Cree, A., Dao, M., Davis, C., Chacko, J., Dinh, H., Dugan-Rocha, S., Fowler, G., Garner, T.T., Garnes, J., Gnirke, A., Hawes, A., Hernandez, J., Hines, S., Holder, M., Hume, J., Jhangiani, S.N., Joshi, V., Khan, Z.M., Jackson, L., Kovar, C., Kowis, A., Lee, S., Lewis, L.R., Margolis, J., Morgan, M., Nazareth, L.V., Nguyen, N., Okwuonu, G., Parker, D., Ruiz, S.-J., Santibanez, J., Savard, J., Scherer, S.E., Schneider, B., Sodergren, E., Vattahil, S., Villasana, D., White, C.S., Wright, R., Park, Y., Lord, J., Oppert, B., Brown, S., Wang, L.J., Savard, J., Liu, Y., Worley, K., Elsik, C.G., Reese, J.T., Elhaik, E., Landan, G., Graur, D., Arensburger, P., Atkinson, P., Beidler, J., Demuth, J.P., Drury, D.W., Du, Y.-Z., Fujiwara, H., Maselli, V., Osanai, M., Robertson, H.M., Tu, Z.J., Wang, J.-J., Wang, S.Z., Song, H., Zhang, L., Sodergren, E., Werner, D., Stanke, M., Morgenstern, B., Solovyev, V., Kosarev, P., Brown, G., Chen, H.-C., Ermolaeva, O., Hlavina, W., Kapustin, Y., Kiryutin, B., Kitts, P., Maglott, D., Pruitt, K., Sapojnikov, V., Souvorov, A., Mackey, A.J., Waterhouse, R.M., Wyder, S., Zdobnov, E.M., Kriventseva, E.V., Kadowaki, T., Bork, P., Aranda, M., Bao, R.Y., Beermann, A., Berns, N., Bolognesi, R., Bonneton, F., Bopp, D., Butts, T., Chaumot, A., Denell, R.E., Ferrier, D.E.K., Gordon, C.M., Jindra, M., Klingler, M., Lan, Q., Lattorff, H.M.G., Laudet, V., von Levetsow, C., Liu, Z.Y., Lutz, R., Lynch, J.A., da Fonseca, R.N., Posnien, N., Reuter, R., Roth, S., Schinko, J.B., Schmitt, C., Schoppmeier, M., Shippy, T.D., Simonnet, F., Marques-Souza, H., Tomoyasu, Y., Trauner, J., Van der Zee, M., Vervoort, M., Wittkopp, N., Wimmer, E.A., Yang, X.Y., Jones, A.K., Sattelle, D.B., Ebert, P.R., Nelson, D., Scott, J.G., Muthukrishnan, S., Kramer, K.J., Arakane, Y., Zhu, Q.S., Hogenkamp, D., Dixit, R., Jiang, H.B., Zou, Z., Marshall, J., Elpidina, E., Vinokurov, K., Oppert, C., Evans, J., Lu, Z.Q., Zhao, P.C., Sumathipala, N., Altincicek, B., Vilcinskas, A., Williams, M., Hultmark, D., Hetru, C., Hauser, F., Cazzamali, G., Williamson, M., Li, B., Tanaka, Y., Predel, R., Neupert, S., Schachtner, J., Verleyen, P., Raible, F., Walden, K.K.O., Robertson, H.M., Angeli, S., Forêt, S., Schuetz, S., Maleszka, R., Miller, S.C., Grossmann, D., Tribolium Genome Sequencing Consortium, 2008. The genome of the model beetle and pest *Tribolium castaneum*. Nature 452**,** 949-955.

Robertson, G., Schein, J., Chiu, R., Corbett, R., Field, M., Jackman, S.D., Mungall, K., Lee, S., Okada, H.M., Qian, J.Q., Griffith, M., Raymond, A., Thiessen, N., Cezard, T., Butterfield, Y.S., Newsome, R., Chan, S.K., She, R., Varhol, R., Kamoh, B., Prabhu, A.L., Tam, A., Zhao, Y.J., Moore, R.A., Hirst, M., Marra, M.A., Jones, S.J.M., Hoodless, P.A., Birol, I., 2010. *De novo* assembly and analysis of RNA-seq data. Nature Methods 7**,** 909-U962.

Robertson, J.A., Ślipiński, A., Moulton, M., Shockley, F.W., Giorgi, A., Lord, N.P., Mckenna, D.D., Tomaszewska, W., Forrester, J., Miller, K.B., Whiting, M.F., McHugh, J.V., 2015. Phylogeny and classification of Cucujoidea and the recognition of a new superfamily Coccinelloidea (Coleoptera: Cucujiformia). Systematic Entomology 40**,** 745-778.

Rodriguez-de la Noval, C., Rodriguez-Cabrera, L., Izquierdo, L., Espinosa, L.A., Hernandez, D., Ponce, M., Moran-Bertot, I., Tellez-Rodriguez, P., Borras-Hidalgo, O., Huang, S.L., Kan, Y.C., Wright, D.J., Ayra-Pardo, C., 2019. Functional expression of a peritrophin A-like SfPER protein is required for larval development in *Spodoptera frugiperda* (Lepidoptera: Noctuidae). Scientific Reports 9**,** 2630.

Rondoni, G., Roman, A., Meslin, C., Montagné, N., Conti, E., Jacquin-Joly, E., 2021. Antennal Transcriptome Analysis and Identification of Candidate Chemosensory Genes of the Harlequin Ladybird Beetle, *Harmonia axyridis* (Pallas) (Coleoptera: Coccinellidae). Insects 12**,** 209.

Ruan, J., Li, H., 2020. Fast and accurate long-read assembly with wtdbg2. Nature Methods 17**,** 155-158.

Sabbadin, F., Hemsworth, G.R., Ciano, L., Henrissat, B., Dupree, P., Tryfona, T., Marques, R.D.S., Sweeney, S.T., Besser, K., Elias, L., Pesante, G., Li, Y., Dowle, A.A., Bates, R., Gomez, L.D., Simister, R., Davies, G.J., Walton, P.H., Bruce, N.C., McQueen-Mason, S.J., 2018. An ancient family of lytic polysaccharide monooxygenases with roles in arthropod development and biomass digestion. Nature Communications 9**,** 756.

Schoville, S.D., Chen, Y.H., Andersson, M.N., Benoit, J.B., Bhandari, A., Bowsher, J.H., Brevik, K., Cappelle, K., Chen, M.-J.M., Childers, A.K., Childers, C., Christiaens, O., Clements, J., Didion, E.M., Elpidina, E.N., Engsontia, P., Friedrich, M., García-Robles, I., Gibbs, R.A., Goswami, C., Grapputo, A., Gruden, K., Grynberg, M., Henrissat, B., Jennings, E.C., Jones, J.W., Kalsi, M., Khan, S.A., Kumar, A., Li, F., Lombard, V., Ma, X., Martynov, A., Miller, N.J., Mitchell, R.F., Munoz-Torres, M., Muszewska, A., Oppert, B., Palli, S.R., Panfilio, K.A., Pauchet, Y., Perkin, L.C., Petek, M., Poelchau, M.F., Record, E., Rinehart, J.P., Robertson, H.M., Rosendale, A.J., Ruiz-Arroyo, V.M., Smagghe, G., Szendrei, Z., Thomas, G.W.C., Torson, A.S., Jentzsch, I.M.V., Weirauch, M.T., Yates, A.D.T., Yocum, G.D., Yoon, J.-S., Richards, S., 2018. A model species for agricultural pest genomics: the genome of the Colorado potato beetle, *Leptinotarsa decemlineata* (Coleoptera: Chrysomelidae). Scientific Reports 8**,** 1931.

Schulz, M.H., Zerbino, D.R., Vingron, M., Birney, E., 2012. *Oases*: robust *de novo* RNA-seq assembly across the dynamic range of expression levels. Bioinformatics 28**,** 1086-1092.

Seago, A.E., Giorgi, J.A., Li, J.H., Ślipiński, A., 2011. Phylogeny, classification and evolution of ladybird beetles (Coleoptera: Coccinellidae) based on simultaneous analysis of molecular and morphological data. Molecular Phylogenetics and Evolution 60**,** 137-151.

Seagraves, M.P., 2009. Lady beetle oviposition behavior in response to the trophic environment. Biological Control 51**,** 313-322.

Seppey, M., Ioannidis, P., Emerson, B.C., Pitteloud, C., Robinson-Rechavi, M., Roux, J., Escalona, H.E., McKenna, D.D., Misof, B., Shin, S., Zhou, X., Waterhouse, R.M., Alvarez, N., 2019. Genomic signatures accompanying the dietary shift to phytophagy in polyphagan beetles. Genome Biology 20**,** 98.

Servant, N., Varoquaux, N., Lajoie, B.R., Viara, E., Chen, C.J., Vert, J.P., Heard, E., Dekker, J., Barillot, E., 2015. HiC-Pro: an optimized and flexible pipeline for Hi-C data processing. Genome Biology 16**,** 259.

Shakeel, M., Xu, X.X., De Mandal, S., Jin, F.L., 2019. Role of serine protease inhibitors in insect-host-pathogen interactions. Archives of Insect Biochemistry and Physiology 102**,** e21556.

Shen, Y.-Y., Liang, L., Zhu, Z.-H., Zhou, W.-P., Irwin, D.M., Zhang, Y.-P., 2010. Adaptive evolution of energy metabolism genes and the origin of flight in bats. Proceedings of the National Academy of Sciences of the United States of America 107**,** 8666-8671.

Shen, Y.-Y., Shi, P., Sun, Y.-B., Zhang, Y.-P., 2009. Relaxation of selective constraints on avian mitochondrial DNA following the degeneration of flight ability. Genome Research 19**,** 1760-1765.

Shen, Z.H., Zhou, Y., 2023. Genome for *Harmonia axyridis* and immune system analysis. Acta Agriculturae Universitatis Jiangxiensis 45**,** 45-52.

Simao, F.A., Waterhouse, R.M., Ioannidis, P., Kriventseva, E.V., Zdobnov, E.M., 2015. BUSCO: assessing genome assembly and annotation completeness with single-copy orthologs. Bioinformatics 31**,** 3210-3212.

Simion, P., Belkhir, K., Francois, C., Veyssier, J., Rink, J.C., Manuel, M., Philippe, H., Telford, M.J., 2018. A software tool 'CroCo' detects pervasive cross-species contamination in next generation sequencing data. Bmc Biology 16**,** 28.

Slater, G.S., Birney, E., 2005. Automated generation of heuristics for biological sequence comparison. BMC Bioinformatics 6**,** 31.

Ślipiński, A., 2007. Australian Ladybird Beetles (Coleoptera: Coccinellidae): Their biology and classification. Australian Biological Resources Study, Canberra.

Ślipiński, A., Li, J.H., Pang, H., 2020. Ladybird Beetles of the Australo-Pacific Region: Coleoptera: Coccinellidae: Coccinellini. CSIRO Publishing, Melbourne.

Song, N., Li, X.X., Yin, X.M., Li, X.H., Xi, Y.Q., 2020. The mitochondrial genomes of ladybird beetles and implications for evolution and phylogeny. International Journal of Biological Macromolecules 147**,** 1193-1203.

Stanke, M., Diekhans, M., Baertsch, R., Haussler, D., 2008. Using native and syntenically mapped cDNA alignments to improve *de novo* gene finding. Bioinformatics 24**,** 637-644.

Strauss, A.S., Peters, S., Boland, W., Burse, A., 2013. ABC transporter functions as a pacemaker for sequestration of plant glucosides in leaf beetles. Elife 2**,** e01096.

Sun, S.-L., Abudisilimu, N., Yi, H., Li, S.L., Liu, T.-X., Jing, X.F., 2022. Understanding nutritive need in *Harmonia axyridis* larvae: Insights from nutritional geometry. Insect Science 29**,** 1433-1444.

Sutherland, A.M., Parrella, M.P., 2009. Mycophagy in Coccinellidae: Review and synthesis. Biological Control 51**,** 284-293.

Szawaryn, K., 2019. Unexpected diversity of whitefly predators in Eocene Baltic amber-new fossil *Serangium* species (Coleoptera: Coccinellidae). Zootaxa 4571**,** 270-276.

Szawaryn, K., 2021. The first fossil Microweiseini (Coleoptera: Coccinellidae) from the Eocene of Europe and its significance for the reconstruction of the evolution of ladybird beetles. Zoological Journal of the Linnean Society 193**,** 1294-1309.

Szawaryn, K., Bocak, L., Ślipiński, A., Escalona, H.E., Tomaszewska, W., 2015. Phylogeny and evolution of phytophagous ladybird beetles (Coleoptera: Coccinellidae: Epilachnini), with recognition of new genera. Systematic Entomology 40**,** 547-569.

Szawaryn, K., Szwedo, J., 2018. Have ladybird beetles and whiteflies co-existed for at least 40 Mya? Palz 92**,** 593-603.

Szawaryn, K., Tomaszewska, W., 2020a. The First Fossil Sticholotidini Ladybird Beetle (Coleoptera, Coccinellidae) Reveals a Transition Zone through Northern Europe during the Eocene. Papers in Palaeontology 6**,** 651-659.

Szawaryn, K., Tomaszewska, W., 2020b. New and known extinct species of *Rhyzobius* Stephens, 1829 shed light on the phylogeny and biogeography of the genus and the tribe Coccidulini (Coleoptera: Coccinellidae). Journal of Systematic Palaeontology 18**,** 1445-1461.

Tabata, J., De Moraes, C.M., Mescher, M.C., 2011. Olfactory Cues from Plants Infected by Powdery Mildew Guide Foraging by a Mycophagous Ladybird Beetle. Plos One 6**,** e23799.

Takemura, M., Maoka, T., Koyanagi, T., Kawase, N., Nishida, R., Tsuchida, T., Hironaka, M., Ueda, T., Misawa, N., 2021. Elucidation of the whole carotenoid biosynthetic pathway of aphids at the gene level and arthropodal food chain involving aphids and the red dragonfly. Bmc Zoology 6**,** 19.

Tang, H.-Y., Liu, J.-T., Xie, J.-X., Yi, C.-Q., Liu, X.-X., Zhang, Y.-J., Sun, Y., 2022a. Gene cloning and ligand binding characterization of the odorant-binding protein HvarOBP2 in *Hippodamia variegata* (Coleoptera: Coccinellidae). Acta Entomologica Sinica 65**,** 977-985.

Tang, H.Y., Xie, J.X., Liu, J.T., Khashaveh, A., Liu, X.X., Yi, C.Q., Zhao, D.Y., He, L., Sun, Y., Zhang, Y.J., 2023a. Odorant-Binding Protein *HvarOBP*5 in Ladybird *Hippodamia variegata* Regulates the Perception of Semiochemicals from Preys and Habitat Plants. Journal of Agricultural and Food Chemistry 71**,** 1067-1076.

Tang, X.-F., Huang, Y.-H., Li, H.-S., Chen, P.-T., Yang, H.-Y., Liang, Y.-S., Du, X.-Y., Liu, Z.-H., Li, E.-F., Yang, Y.-C., Pang, H., 2022b. Genomic insight into the scale specialization of the biological control agent *Novius pumilus* (Weise, 1892). Bmc Genomics 23**,** 90.

Tang, X.-F., Huang, Y.-H., Sun, Y.-F., Zhang, P.-F., Huo, L.-Z., Li, H.-S., Pang, H., 2023b. The transcriptome of *Icerya aegyptiaca* (Hemiptera: Monophlebidae) and comparison with neococcoids reveal genetic clues of evolution in the scale insects. Bmc Genomics 24**,** 231.

Tetreau, G., Dittmer, N.T., Cao, X.L., Agrawal, S., Chen, Y.R., Muthukrishnan, S., Jiang, H.B., Blissard, G.W., Kanost, M.R., Wang, P., 2015. Analysis of chitin-binding proteins from *Manduca sexta* provides new insights into evolution of peritrophin A-type chitin-binding domains in insects. Insect Biochemistry and Molecular Biology 62**,** 127-141.

Thomas, G.W.C., Dohmen, E., Hughes, D.S.T., Murali, S.C., Poelchau, M., Glastad, K., Anstead, C.A., Ayoub, N.A., Batterham, P., Bellair, M., Binford, G.J., Chao, H., Chen, Y.H., Childers, C., Dinh, H., Doddapaneni, H.V., Duan, J.J., Dugan, S., Esposito, L.A., Friedrich, M., Garb, J., Gasser, R.B., Goodisman, M.A.D., Gundersen-Rindal, D.E., Han, Y., Handler, A.M., Hatakeyama, M., Hering, L., Hunter, W.B., Ioannidis, P., Jayaseelan, J.C., Kalra, D., Khila, A., Korhonen, P.K., Lee, C.E., Lee, S.L., Li, Y.Y., Lindsey, A.R.I., Mayer, G., McGregor, A.P., McKenna, D.D., Misof, B., Munidasa, M., Munoz-Torres, M., Muzny, D.M., Niehuis, O., Osuji-Lacy, N., Palli, S.R., Panfilio, K.A., Pechmann, M., Perry, T., Peters, R.S., Poynton, H.C., Prpic, N.-M., Qu, J.X., Rotenberg, D., Schal, C., Schoville, S.D., Scully, E.D., Skinner, E., Sloan, D.B., Stouthamer, R., Strand, M.R., Szucsich, N.U., Wijeratne, A., Young, N.D., Zattara, E.E., Benoit, J.B., Zdobnov, E.M., Pfrender, M.E., Hackett, K.J., Werren, J.H., Worley, K.C., Gibbs, R.A., Chipman, A.D., Waterhouse, R.M., Bornberg-Bauer, E., Hahn, M.W., Richards, S., 2020. Gene content evolution in the arthropods. Genome Biology 21**,** 15.

Tomaszewska, W., Escalona, H.E., Hartley, D., Li, J.H., Wang, X.M., Li, H.-S., Pang, H., Ślipiński, A., Zwick, A., 2021. Phylogeny of true ladybird beetles (Coccinellidae: Coccinellini) reveals pervasive convergent evolution and a rapid Cenozoic radiation. Systematic Entomology 46**,** 611-631.

Tomaszewska, W., Ślipiński, A., Bai, M., Zhang, W.W., Ren, D., 2018. The oldest representatives of Endomychidae (Coleoptera: Coccinelloidea) from the Upper Cretaceous Burmese amber. Cretaceous Research 91**,** 287-298.

Tong, H.J., Wang, Y., Wang, S.P., Omar, M.A.A., Li, Z.C., Li, Z.H., Ding, S.M., Ao, Y., Wang, Y., Li, F., Jiang, M.X., 2022. Fatty acyl-CoA reductase influences wax biosynthesis in the cotton mealybug, *Phenacoccus solenopsis* Tinsley. Communications Biology 5**,** 1108.

Toussaint, E.F.A., Seidel, M., Arriaga-Varela, E., Hajek, J., Kral, D., Sekerka, L., Short, A.E.Z., Fikacek, M., 2017. The peril of dating beetles. Systematic Entomology 42**,** 1-10.

Tsuji, T., Gotoh, H., Morita, S., Hirata, J., Minakuchi, Y., Yaginuma, T., Toyoda, A., Niimi, T., 2018. Molecular Characterization of Eye Pigmentation-Related ABC Transporter Genes in the Ladybird Beetle *Harmonia axyridis* Reveals Striking Gene Duplication of the *white* Gene. Zoological Science 35**,** 260-267.

Urbina, A., Verdugo, J.A., López, E., Bergmann, J., Zaviezo, T., Flores, M.F., 2018. Searching Behavior of *Cryptolaemus montrouzieri* (Coleoptera: Coccinellidae) in Response to Mealybug Sex Pheromones. Journal of Economic Entomology 111**,** 1996-1999.

Van Dam, M.H., Cabras, A.A., Henderson, J.B., Rominger, A.J., Estrada, C.P., Omer, A.D., Dudchenko, O., Aiden, E.L., Lam, A.W., 2021. The Easter Egg Weevil (*Pachyrhynchus*) genome reveals syntenic patterns in Coleoptera across 200 million years of evolution. PLoS Genetics 17**,** e1009745.

Vaser, R., Sović, I., Nagarajan, N., Šikić, M., 2017. Fast and accurate de novo genome assembly from long uncorrected reads. Genome Research 27**,** 737-746.

Vasimuddin, M., Misra, S., Li, H., Aluru, S., 2019. Efficient Architecture-Aware Acceleration of BWA-MEM for Multicore Systems. 2019 IEEE 33rd International Parallel and Distributed Processing Symposium (IPDPS 2019)**,** 314-324.

Vea, I.M., Grimaldi, D.A., 2016. Putting scales into evolutionary time: the divergence of major scale insect lineages (Hemiptera) predates the radiation of modern angiosperm hosts. Scientific Reports 6**,** 23487.

Verheggen, F., Cherif, A., Martin, C., 2020. The Production of Sex Pheromone in Lady Beetles Is Conditioned by Presence of Aphids and Not by Mating Status. Journal of Chemical Ecology 46**,** 590-596.

Vilcinskas, A., Mukherjee, K., Vogel, H., 2013. Expansion of the antimicrobial peptide repertoire in the invasive ladybird *Harmonia axyridis*. Proceedings of the Royal Society B-Biological Sciences 280**,** 20122113.

Vizán-Rico, H.I., Mayer, C., Petersen, M., McKenna, D.D., Zhou, X., Gómez-Zurita, J., 2019. Patterns and Constraints in the Evolution of Sperm Individualization Genes in Insects, with an Emphasis on Beetles. Genes 10**,** 776.

Wagih, O., 2017. ggseqlogo: a versatile R package for drawing sequence logos. Bioinformatics 33**,** 3645-3647.

Walker, A.J., Ford, L., Majerus, M.E.N., Geoghegan, I.E., Birch, N., Gatehouse, J.A., Gatehouse, A.M.R., 1998. Characterisation of the mid-gut digestive proteinase activity of the two-spot ladybird (*Adalia bipunctata* L.) and its sensitivity to proteinase inhibitors. Insect Biochemistry and Molecular Biology 28**,** 173-180.

Walker, B.J., Abeel, T., Shea, T., Priest, M., Abouelliel, A., Sakthikumar, S., Cuomo, C.A., Zeng, Q.D., Wortman, J., Young, S.K., Earl, A.M., 2014. Pilon: An Integrated Tool for Comprehensive Microbial Variant Detection and Genome Assembly Improvement. Plos One 9**,** e112963.

Wang, B., Ma, J.Y., McKenna, D.D., Yan, E.V., Zhang, H.C., Jarzembowski, E.A., 2014. The earliest known longhorn beetle (Cerambycidae: Prioninae) and implications for the early evolution of Chrysomeloidea. Journal of Systematic Palaeontology 12**,** 565-574.

Wang, Q.Y., Liu, L.W., Zhang, S.J., Wu, H., Huang, J.H., 2022. A chromosome-level genome assembly and intestinal transcriptome of *Trypoxylus dichotomus* (Coleoptera: Scarabaeidae) to understand its lignocellulose digestion ability. Gigascience 11**,** giac059.

Wang, Y.P., Tang, H.B., DeBarry, J.D., Tan, X., Li, J.P., Wang, X.Y., Lee, T.H., Jin, H.Z., Marler, B., Guo, H., Kissinger, J.C., Paterson, A.H., 2012. MCScanX: a toolkit for detection and evolutionary analysis of gene synteny and collinearity. Nucleic Acids Research 40**,** e49.

Waterhouse, R.M., Kriventseva, E.V., Meister, S., Xi, Z.Y., Alvarez, K.S., Bartholomay, L.C., Barillas-Mury, C., Bian, G.W., Blandin, S., Christensen, B.M., Dong, Y.M., Jiang, H.B., Kanost, M.R., Koutsos, A.C., Levashina, E.A., Li, J.Y., Ligoxygakis, P., MacCallum, R.M., Mayhew, G.F., Mendes, A., Michel, K., Osta, M.A., Paskewitz, S., Shin, S.W., Vlachou, D., Wang, L.H., Wei, W.Q., Zheng, L.B., Zou, Z., Severson, D.W., Raikhel, A.S., Kafatos, F.C., Dimopoulos, G., Zdobnov, E.M., Christophides, G.K., 2007. Evolutionary dynamics of immune-related genes and pathways in disease-vector mosquitoes. Science 316**,** 1738-1743.

Weil, T., Rehli, M., Korb, J., 2007. Molecular basis for the reproductive division of labour in a lower termite. Bmc Genomics 8**,** 198.

Wellcome Sanger Institute Tree of Life programme, Wellcome Sanger Institute Scientific Operations: DNA Pipelines collective, Tree of Life Core Informatics collective, Goate, Z., Darwin Tree of Life Consortium, 2022. The genome sequence of the two-spot ladybird, *Adalia bipunctata* (Linnaeus, 1758) [version 1; peer review: 2 approved, 3 approved with reservations]. Wellcome Open Research 7**,** 288.

Weng, Y.-M., Francoeur, C.B., Currie, C.R., Kavanaugh, D.H., Schoville, S.D., 2021. A high-quality carabid genome assembly provides insights into beetle genome evolution and cold adaptation. Molecular Ecology Resources 21**,** 2145-2165.

Woodring, J., 2020. A comparison of ten digestive enzymes reveals a lack of chitinase in a phasmid and the loss of two β-glucanases in a mantid. Physiological Entomology 45**,** 89-94.

Wu, T.D., Watanabe, C.K., 2005. GMAP: a genomic mapping and alignment program for mRNA and EST sequences. Bioinformatics 21**,** 1859-1875.

Xiang, M., Zhang, H.-Z., Jing, X.-Y., Wang, M.-Q., Mao, J.-J., Li, Y.-Y., Zang, L.-S., Zhang, L.-S., 2021. Sequencing, Expression, and Functional Analyses of Four Genes Related to Fatty Acid Biosynthesis During the Diapause Process in the Female Ladybird, *Coccinella septempunctata* L. Frontiers in Physiology 12**,** 706032.

Xie, J.X., Liu, T.H., Yi, C.Q., Liu, X.X., Tang, H.Y., Sun, Y., Shi, W.P., Khashaveh, A., Zhang, Y.J., 2022. Antenna-Biased Odorant Receptor HvarOR25 in *Hippodamia variegata* Tuned to Allelochemicals from Hosts and Habitat Involved in Perceiving Preys. Journal of Agricultural and Food Chemistry 70**,** 1090-1100.

Xie, Y.L., Wu, G.X., Tang, J.B., Luo, R.B., Patterson, J., Liu, S.L., Huang, W.H., He, G.Z., Gu, S.C., Li, S.K., Zhou, X., Lam, T.W., Li, Y.R., Xu, X., Wong, G.K.S., Wang, J., 2014. SOAPdenovo-Trans: *de novo* transcriptome assembly with short RNA-Seq reads. Bioinformatics 30**,** 1660-1666.

Xu, P., Ze, L.-J., Kang, W.-N., Wu, J.-J., Jin, L., Anjum, A.A., Li, G.-Q., 2020. Functional divergence of *white* genes in *Henosepilachna vigintioctopunctata* revealed by RNA interference. Insect Molecular Biology 29**,** 466-476.

Yang, Z.-K., Qu, C., Pan, S.-X., Liu, Y., Shi, Z., Luo, C., Qin, Y.-G., Yang, X.-L., 2023. Aphid-repellent, ladybug-attraction activities, and binding mechanism of methyl salicylate derivatives containing geraniol moiety. Pest Management Science 79**,** 760-770.

Yang, Z.H., 2007. PAML 4: Phylogenetic analysis by maximum likelihood. Molecular Biology and Evolution 24**,** 1586-1591.

Yeo, S., Coombe, L., Warren, R.L., Chu, J., Birol, I., 2018. ARCS: scaffolding genome drafts with linked reads. Bioinformatics 34**,** 725-731.

Yin, Y.B., Mao, X.Z., Yang, J.C., Chen, X., Mao, F.L., Xu, Y., 2012. dbCAN: a web resource for automated carbohydrate-active enzyme annotation. Nucleic Acids Research 40**,** W445-W451.

Yu, G.C., Smith, D.K., Zhu, H.C., Guan, Y., Lam, T.T.Y., 2017. GGTREE: an R package for visualization and annotation of phylogenetic trees with their covariates and other associated data. Methods in Ecology and Evolution 8**,** 28-36.

Yu, G.C., Wang, L.-G., Han, Y.Y., He, Q.-Y., 2012. clusterProfiler: an R Package for Comparing Biological Themes Among Gene Clusters. OMICS: A Journal of Integrative Biology 16**,** 284-287.

Yuan, M.-L., Chen, W.-T., Zhang, Q.-L., Li, M., Zhang, L., Tang, P.-A., 2023. Transcriptomic data recover a new superfamily-level phylogeny of Cucujiformia (Coleoptera, Polyphaga). Molecular Phylogenetics and Evolution 179**,** 107679.

Zdobnov, E.M., Tegenfeldt, F., Kuznetsov, D., Waterhouse, R.M., Simao, F.A., Ioannidis, P., Seppey, M., Loetscher, A., Kriventseva, E.V., 2017. OrthoDB v9.1: cataloging evolutionary and functional annotations for animal, fungal, plant, archaeal, bacterial and viral orthologs. Nucleic Acids Research 45**,** D744-D749.

Zhang, C., Scornavacca, C., Molloy, E.K., Mirarab, S., 2020a. ASTRAL-Pro: Quartet-Based Species-Tree Inference despite Paralogy. Molecular Biology and Evolution 37**,** 3292-3307.

Zhang, J.F., Sun, B., Zhang, X.Y., 1994. Miocene Insects and Spiders from Shanwang, Shandong. Science Press, Beijing.

Zhang, L.J., Li, S., Luo, J.Y., Du, P., Wu, L.K., Li, Y.R., Zhu, X.Z., Wang, L., Zhang, S., Cui, J.J., 2020b. Chromosome-level genome assembly of the predator *Propylea japonica* to understand its tolerance to insecticides and high temperatures. Molecular Ecology Resources 20**,** 292-307.

Zhang, S.-Q., Che, L.-H., Li, Y., Liang, D., Pang, H., Ślipiński, A., Zhang, P., 2018. Evolutionary history of Coleoptera revealed by extensive sampling of genes and species. Nature Communications 9**,** 205.

Zhang, Y.-H., Zheng, J.-H., Mao, R.-Q., Pang, H., 2015. Cloning and characterization analysis of cytochrome P450 *CYP*9Z401 gene from *Cryptolaemus montrouzieri* Mulsant. Journal of Environmental Entomology 37**,** 759-766.

Zhang, Y., Feng, Z.-J., Chen, Z.-S., Wang, X.-X., Cong, H.-S., Fan, Y.-L., Liu, T.-X., 2021. Connection between cuticular hydrocarbons and melanization in *Harmonia axyridis* revealed by RNAi-mediated silencing of the *CYP4G79*. Entomologia Generalis 41**,** 83-96.

Zhang, Z.Q., Pei, P., Zhang, M., Li, F.F., Tang, G.H., 2023. Chromosome-level genome assembly of *Dastarcus helophoroides* provides insights into CYP450 genes expression upon insecticide exposure. Pest Management Science 79**,** 1467-1482.

Zhu, Q.S., Arakane, Y., Banerjee, D., Beeman, R.W., Kramer, K.J., Muthukrishnan, S., 2008a. Domain organization and phylogenetic analysis of the chitinase-like family of proteins in three species of insects. Insect Biochemistry and Molecular Biology 38**,** 452-466.

Zhu, Q.S., Arakane, Y., Beeman, R.W., Kramer, K.J., Muthukrishnan, S., 2008b. Functional specialization among insect chitinase family genes revealed by RNA interference. Proceedings of the National Academy of Sciences of the United States of America 105**,** 6650-6655.

Zhu, W.B., Chi, S.Q., Wang, Y.C., Li, H.R., Wang, Z.K., Gu, S.D., Sun, T., Xiang, H., You, P., Ren, Y.D., 2023. A chromosome-level genome assembly of the *Henosepilachna vigintioctomaculata* provides insights into the evolution of ladybird beetles. DNA Research**,** dsad001.

Zibaee, A., 2020. A Biochemical study on the types of digestive specific proteases in the ladybird, *Cryptolaemus montrouzieri* Mulsant (Col.: Coccinellidae) and the effects of different preys on their activity. Plant Pest Research 10**,** 75-91.
